# Serum Exosomal MicroRNAs as Potential Circulating Biomarkers for Endometriosis

**DOI:** 10.1155/2020/2456340

**Published:** 2020-01-23

**Authors:** Lu Zhang, Huihui Li, Ming Yuan, Dong Li, Chang Sun, Guoyun Wang

**Affiliations:** ^1^Department of Obstetrics and Gynecology, Qilu Hospital of Shandong University, NO.107 Wenhuaxi Road, Jinan, Shandong 250012, China; ^2^Cryomedicine Lab, Qilu Hospital of Shandong University, Jinan, China; ^3^School of Material Science and Engineering, Shandong Jianzhu University, Jinan 250101, China

## Abstract

**Background:**

A reliable noninvasive biomarker is not yet available for endometriosis diagnosis. Novel biomarkers for the diagnosis of endometriosis are urgently needed. The molecular constituents of exosomes, especially exosomal microRNAs (miRNAs), have considerable potential as novel biomarkers for clinical diagnosis. This study is aimed at exploring aberrant exosomal miRNA profiles by using miRNA microarray and at providing more accurate molecular biomarkers of endometriosis.

**Methods:**

Exosomes were isolated from the serum of patients with endometriosis and negative controls and identified by electron microscopy, nanoparticle tracking analysis, and Western blot. Exosomal miRNAs were profiled by miRNA microarrays. The expression of selective serum exosomal miRNA was validated by qRT-PCR. Receiver operating characteristic (ROC) curves were established to explore the diagnostic value of selective miRNAs. Finally, GO annotation and KEGG pathway enrichment analyses were used to display possible functions associated with the two miRNAs.

**Results:**

A total of 24 miRNAs showed differential levels of enrichment with *P* < 0.05 and |log_2_ fold change| > 1 by miRNA microarrays. Among the six selective miRNAs (i.e., miR-134-5p, miR-197-5p, miR-22-3p, miR-320a, miR-494-3p, and miR-939-5p), qRT-PCR analysis revealed that miR-22-3p and miR-320a were significantly upregulated in serum exosomes from patients with endometriosis compared with negative individuals. ROC curve revealed that the serum exosomal miR-22-3p and miR-320a yielded the area under the curve values of 0.855 and 0.827, respectively.

**Conclusion:**

Our results demonstrated that exosomal miR-22-3p and miR-320a were significantly increased in the sera of patients with endometriosis. The two miRNAs may be useful potential biomarkers for endometriosis diagnosis.

## 1. Introduction

Endometriosis, defined as the existence of endometrial tissue outside the uterine cavity, affects approximately 10%–15% of women of reproductive age [1]. The signs and symptoms of patients suffering from endometriosis include pelvic pain, dysmenorrhea, and infertility [2]. Despite the high prevalence of endometriosis, the diagnosis of this disease is often delayed because of the diversity of symptoms and the lack of sensitive biomarkers in the early phase [3]. Especially for pelvic or superficial endometriosis with no ovarian endometriomas or deep infiltrating lesions, which are not easily detected by ultrasound or clinical examination, the need for more timely noninvasive diagnosis is high. Late diagnosis results in delayed treatments, which is the major problem of this disease. The golden standard of endometriosis diagnosis is pathological examination, which indicates that laparoscopic surgery should be performed to provide lesion specimen. However, surgery is a highly invasive procedure with potential risks. Although several studies have found several biomarkers in endometriosis, a definite diagnostic biomarker is not yet available [4, 5].

MicroRNAs (miRNAs) are noncoding functional RNAs that are approximately 22–24 nucleotides in length, which regulate protein expression by targeting the 3′-untranslated region (3′-UTR) of the mRNA [6]. miRNAs play a variety of roles in diverse biological processes, including proliferation, differentiation, growth, and apoptosis [7, 8]. Different miRNA expression profiles between eutopic and ectopic endometria have been reported [9]. miRNAs also play important roles in the pathogenesis, diagnosis, and prognosis of endometriosis [10–12]. Circulating miRNAs also can serve as noninvasive biomarkers for endometriosis [13, 14].

Exosomes are 50–150 nm endosome-derived extracellular vesicles that are widely distributed in many bodily fluids, such as blood, urine, ascites, and amniotic fluid [15]. Exosomes are secreted by various cell types, which envelop various biological molecules, including proteins, RNAs, DNAs, and lipids [16]. Exosomes play an important role in the exchange of biological information between different cells [17]. Considering that exosomes are protected from RNase degradation, stable miRNAs can be detected in exosomes [18]. Many studies have explored exosomal miRNAs as noninvasive biomarkers in some disease [19–21]. However, to the best of our knowledge, the use of exosomal miRNAs as potential indicators for endometriosis has not been reported.

Considering the aberrant expression of circulating miRNAs in endometriosis and their role in the pathogenesis of endometriosis, we hypothesized that exosomal miRNAs would be differentially expressed in the serum of patients with endometriosis. In this study, our goal was to explore aberrant exosomal miRNA profiles by using miRNA microarray and provide several potential molecular biomarkers of endometriosis. We analyzed also their targeted gene through the GO and KEGG pathway, which provides new ideas for our future research.

## 2. Materials and Methods

### 2.1. Patient and Serum Samples

The study was approved by the ethics committee of Qilu Hospital, Shandong University, and informed consent was obtained from all participants. The blood samples were collected at the hospital from the beginning of 2018 until the end of the year. Patients with pelvic endometriosis who were diagnosed by laparoscopy and confirmed by histopathologic examination served as the endometriosis group. Endometriosis patients were recruited when the following criteria were met: aged 20–50 years, no hormone therapy for at least 3 months, nonsmoker, and without history or signs of other inflammatory disease undergoing surgical treatment. The negative control group included patients with a confirmed absence of endometriosis during their surgical procedure, and the diagnosis of this group was infertility caused by tubal factors. Exclusion criteria were malignancy, benign ovarian cyst except endometrioma, severe pelvic inflammation found in surgery, and polycystic ovarian syndrome. During sampling, clinical data from each individual were collected. Endometriosis was classified as minimal/mild (stage I-II) or moderate/severe (stage III-IV) according to the revised American Society for Reproductive Medicine classification. The blood was collected into serum-separating tubes and allowed to stand for 1 h at room temperature prior to centrifugation at 2,000 × g for 15 min. The supernatant was transferred into labeled fresh tubes and stored at −80°C for further analysis. The sera from five pairs of endometriosis patients and negative controls were used to discover exosomal miRNAs with different expression levels. Then, the sera from the additional 20 negative controls and 20 endometriosis patients were used to validate significantly different expression levels of the selective miRNAs.

### 2.2. Serum Exosome Isolation

Exosomes from the serum were prepared by differential ultracentrifugation as previously described [22]. Briefly, the serum samples were collected and centrifuged at 1,500 × g for 15 min at 4°C. Then, 3 ml of the collected supernatant was centrifuged at 12,000 × g for 30 min at 4°C and finally ultracentrifuged at 110,000 × g for 2 h at 4°C. The pellets containing total exosomes were resuspended in 0.5 ml of phosphate-buffered saline (PBS).

### 2.3. Transmission Electron Microscopy (TEM)

The exosomal pellets were resuspended in 50 *μ*l of PBS and adsorbed to a Cu-coated grid for 10 min. The Cu grid was floated on the drop for 2 min at room temperature and subsequently stained with 2% sodium phosphotungstate for 1 min. The sample was allowed to dry for several minutes and examined by TEM (JEOL, Tokyo, Japan).

### 2.4. Nanoparticle Tracking Analysis (NTA)

The isolated exosomes were resuspended in 500 *μ*l of PBS and analyzed using the NanoSight NS300 System (Malvern Instruments, UK). The movement of pellets under Brownian motion was recorded for 60 s to analyze the particle concentrations and size distribution profiles.

### 2.5. Western Blot Analysis

Western blot analysis was used to identify the exosomal markers, namely, CD9 and CD63. The total exosomal proteins were extracted using RIPA lysis buffer (Beyotime, Shanghai, China). The lysates were incubated for 10 min at 0°C. Then, the supernatants were centrifuged at 10,000 × g for 20 min at 4°C. Bicinchoninic Acid Protein Assay (Beyotime, Shanghai, China) was used to identify the protein concentration. Equivalent protein from each sample was separated on a 10% SDS-PAGE gel, transferred to a PVDF membrane, and blotted with an antibody against CD9, CD63, *β*-actin, and calnexin. All primary antibodies were purchased from Abcam (Shanghai, China), and *β*-actin and calnexin were used as the control. After being washed with TBST three times, the membranes were incubated for 1 h at room temperature with horseradish peroxidase-conjugated secondary antibodies (1 : 10,000 dilution; Boster, Wuhan, China). The bands were visualized using a chemiluminescent substrate SuperSignal West Femto trial kit (Thermo Fisher Scientific, MA, USA).

### 2.6. Exosomal RNA Extraction and Microarray Analysis of miRNA Expression

The miRNAs were extracted from exosomes by using the TRIzol reagent (Invitrogen Life Technologies) according to the manufacturer's protocol. The quality and distribution of miRNAs were determined using the Agilent 2100 Bioanalyzer. Human miRNA microarrays (Agilent Human miR v21.0 array) from Agilent Technologies, including 2,549 mature human miRNAs, were used to determine miRNA expression profiling. The hybridized chip was scanned using the G2565BA Microarray Scanner (Agilent Technologies) and analyzed by GenePix Pro software v4.1 (Molecular Devices Corporation, San Jose, CA). Finally, differentially expressed miRNAs were identified using the paired *t*-test with the cutoff criteria of *P* < 0.05 and fold change ≤ 0.5 or ≥2.

### 2.7. Quantitative Reverse Transcription-Polymerase Chain Reaction (qRT-PCR)

Total RNAs were isolated using TRIzol reagent (Invitrogen Life Technologies) according to the manufacturer's instructions. A mirVana™ miRNA isolation kit was used to extract miRNAs from the serum exosome samples according to the manufacturer's protocol (Thermo Fisher Scientific, MA, USA). For the quantification of miRNA expression levels, RT-PCR reactions were performed using the TaqMan miRNA reverse transcription kit (Applied Biosystems, Carlsbad, CA, USA) and TaqMan Universal PCR Master Mix (Applied Biosystems) according to the manufacturer's protocols. All reactions were performed in triplicate, and U6 snRNA served as the internal control. Results were analyzed using the 2^-∆∆Ct^ method.

### 2.8. GO and Pathway Enrichment Analyses

TargetScan was used to analyze the predicted target of these two validated differentially expressed miRNAs (i.e., miR-22-3p and miR-320a). To clarify the biological functions of the target genes and the involved signaling pathways, we annotated each gene on the basis of the Gene Ontology (GO) and KEGG databases. Enrichment calculations were performed using Fisher' exact test. We also conducted GO and pathway enrichment analysis of the target genes. The specific principle is to carry out annotation mapping of differentially expressed genes in GO and KEGG database entries and calculate the number of the target genes in each GO and pathway entry. The hypergeometric test was used for statistics. Then, we selected the GO and KEGG entries that were significantly enriched in the differentially expressed genes. After the calculated *P* value was corrected by multiple hypothesis tests, the *q* value of 0.05 was set as the threshold, and the GO and KEGG terms meeting this condition were defined as the GO and KEGG terms significantly enriched in the target genes.

### 2.9. Statistical Analysis

Statistical analyses were performed using GraphPad Prism 6.0 (GraphPad Software, San Diego, California, USA) and SPSS19.0 (IBM Company, California, USA). We used two-tailed Student's *t*-test or Mann–Whitney *U* test to identify statistically significant differences among endometriosis patients and negative controls. Error bars represented mean ± SEM. The area under the curve (AUC) of the receiver operating characteristic (ROC) curve was measured to determine the diagnostic value of these validated differentially expressed miRNAs. *P* < 0.05 was considered statistically significant.

## 3. Results

### 3.1. Isolated Serum Exosome Characterization

**Figure 1:**
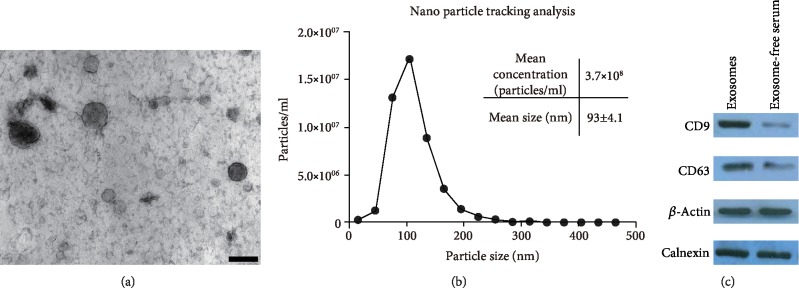
Identification of isolated serum exosomes. (a) Transmission electron microscope images of isolated exosomes. Scale bar = 100 nm. (b) NanoSight analysis for serum exosomes. Horizontal axis, particle size (nm); vertical axis, particle concentration (particles/ml). (c) CD9 and CD63, which are the protein markers of exosomes, were analyzed by Western blot analysis in the exosomes and exosome-free serum. *β*-Actin and calnexin were used as the loading control.

Ultracentrifugation was used to isolate exosomes from the serum. TEM, NTA, and Western blot analysis were used to determine whether we successfully isolated exosomes. TEM showed that isolated exosomes had a round or oval shape (Figure 1(a)). NanoSight analysis demonstrated that the diameter distribution of exosomes ranged from approximately 50 nm to 150 nm in diameter (Figure 1(b)). Nanoparticle calculations on the basis of the NTA experiments revealed that the concentration of exosomes in solution was 3.7 × 10^8^ particles/ml. Western blot analysis was used to confirm the specific exosomal proteins CD63 and CD9. CD9 and CD63 were enriched in the serum exosomes compared with the exosome-depleted serum (Figure 1(c)).

### 3.2. miRNA Microarray Profiling in Serum Exosomes of Patients with Endometriosis and Negative Controls

**Figure 2:**
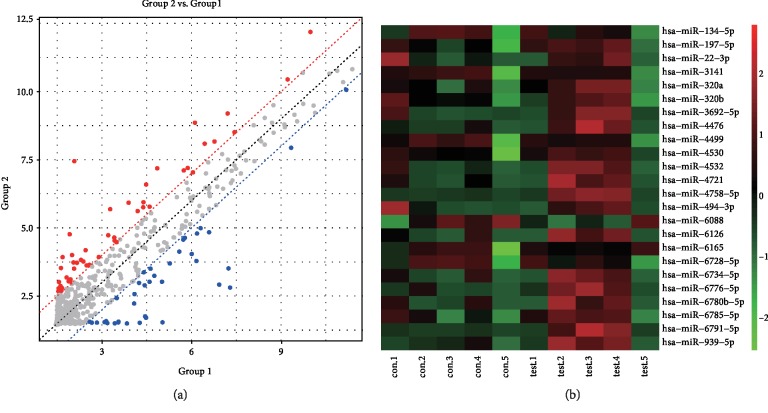
miRNA microarray profiling. (a) Diagram showing 85 differentially expressed miRNAs; blue, downregulated miRNAs; grey, not differentially expressed miRNA; red, upregulated miRNAs. The criteria required a minimum of twofold difference of log_2_ (fold change) in either direction. (b) Significant expression of 24 miRNAs with *P* < 0.05; red, upregulated miRNAs; green, downregulated miRNAs.

We performed miRNA microarray to identify the differential miRNAs in the serum exosomes derived from patients with endometriosis and negative controls. The results are shown in Figure 2(a). A total of 85 differentially expressed miRNAs were identified according to the criteria in the methods section. Among these miRNAs, 24 showed differential levels of enrichment with *P* < 0.05 and |log_2_ fold change| > 1 (Figure 2(b)). As shown in Table 1, 18 serum exosomal miRNAs were upregulated, and 6 were downregulated.

### 3.3. Validation of Significantly Different Expression Levels of Exosomal miRNAs

**Figure 3:**
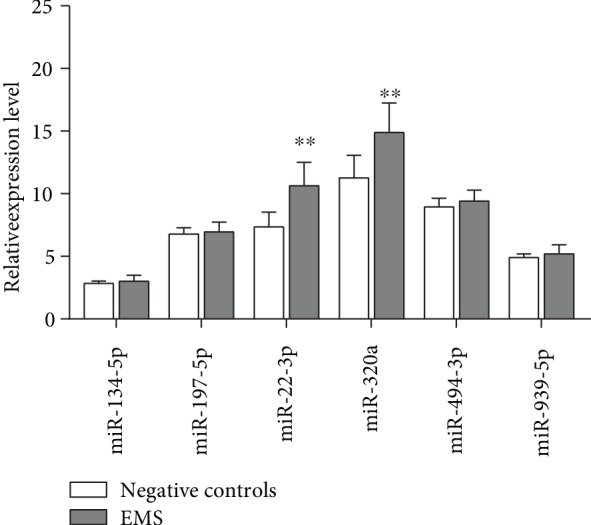
qRT-PCR was used to explore the expression of the six selective miRNAs (i.e., miR-134-5p, miR-197-5p, miR-22-3p, miR-320a, miR-494-3p, and miR-939-5p) in serum exosomes. The obtained values were normalized to U6 snRNA as an internal control. Error bars show the standard error of the mean (SEM). The experiments were repeated three times. ^∗^*P* < 0.05, ^∗∗^*P* < 0.01, compared with negative controls.

On the basis of microarray analysis and the previous literature, we selected miR-134-5p, miR-197-5p, miR-22-3p, miR-320a, miR-494-3p, and miR-939-5p and quantified them through qRT-PCR in serum exosomes from 20 pairs of endometriosis patients and negative individuals. Results revealed that two miRNAs (i.e., miR-22-3p and miR-320a) were significantly upregulated in serum exosomes from endometriosis patients compared with negative individuals (Figure 3). However, the expression levels of the other miRNAs did not differ significantly. The result indicated that serum exosomal miRNAs can serve as noninvasive biomarkers for endometriosis.

### 3.4. Diagnostic Value of Serum Exosomal miRNAs for Patients with Endometriosis

First, we explored the relationship between exosomal miRNA levels and the clinicopathological factors of endometriosis patients. The 20 endometriosis patients were divided into groups of high and low exosomal expression levels of miR-22-3p and miR-320a using the median expression value as the cutoff point. There were no statistically significant differences in parameters such as mean age, pain intensity, or dysmenorrhea between the two groups. The percentage of patients with DIE was higher in the high group of miR-22-3p compared with the low group of miR-22-3p. A higher CA-125 value was associated with higher serum exosomal expression of miR-22-3p and miR-320a in patients with endometriosis (Table 2).

**Figure 4:**
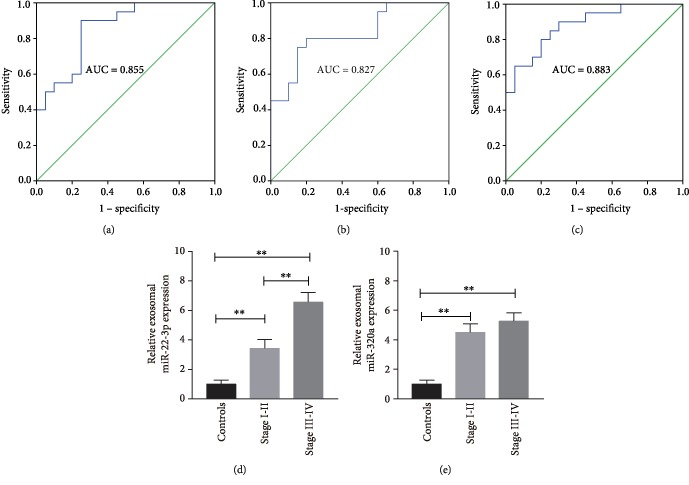
Diagnostic value of serum exosomal miRNAs. ROC curves were produced using each miRNA expression value for miR-22-3p (a) and miR-320a (b) separately and for the combination of miR-22-3p and miR-320a (c). The AUC values with 95% CI were computed for each ROC curve. Wilcoxon–Mann–Whitney test was used to test the null hypothesis that the AUC is 0.5. The expression levels of serum exosomal miR-22-3p (d) and miR-320a (e) were measured in 20 negative controls, 10 endometriosis patients in stage I-II, and 10 endometriosis patients in stage III-IV. ^∗∗^*P* < 0.01, compared with negative controls.

Then ROC curves were established separately for miR-22-3p (Figure 4(a)) and miR-320a (Figure 4(b)) and combined (Figure 4(c)) to explore the diagnostic value of miR-22-3p and miR-320a for endometriosis. The AUC for miR-22-3p was 0.855 (95% CI 0.74–0.97; *P* < 0.01) and for miR-320a was 0.827 (95% CI 0.70-0.96; *P* < 0.01), thereby indicating that the exosomal miR-22-3p had a higher AUC than miR-320a. When miR-22-3p and miR-320a were combined, the AUC increased to 0.883 (95% CI: 0.78 to 0.98; *P* < 0.01). These results indicated that exosomal miRNAs can be found in circulation in patients with endometriosis, and their expression can be used to distinguish endometriosis patients from negative individuals.

Last, we explored the relationship between these two markers and stage of endometriosis. The result showed that miR-22-3p was significantly upregulated in serum exosomes from endometriosis patients in stage III-IV compared with endometriosis patients in stage I-II (Figure 4(d)). No significant difference was detected in the serum levels of exosomal miR-320a at different stages of endometriosis (Figure 4(e)), indicating that miR-320a levels were not correlated with the stage of endometriosis.

### 3.5. Functions and Pathway Analysis of miR-22-3p and miR-320a

**Figure 5:**
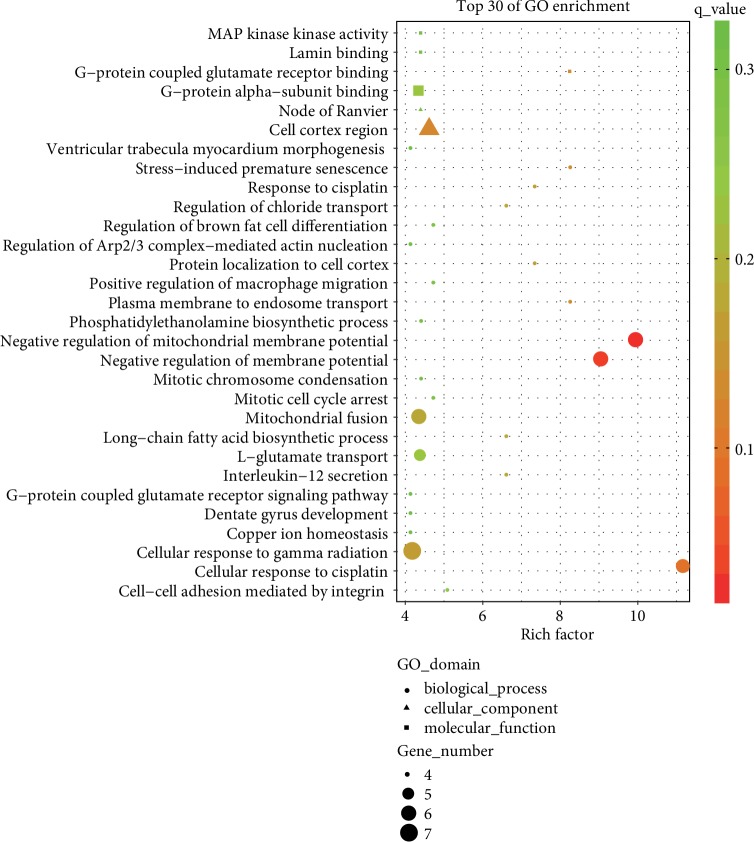
GO analyses of miR-22-3p and miR-320a. The results showed the associated target gene function of the two miRNAs. The enrichment *P* values were calculated by Fisher's exact test. Term/GO in the vertical axis was drawn according to the enrich factor value in descending order. The horizontal axis represents the enrich factor: enrich factor = (dysregulated gene number in a GO term/total dysregulated gene number)/(gene number in a GO term in the database/total gene number in the database). Top 30 GO terms were selected according to the enrich factor value. Selection standards included the gene number in a GO term 4 and *P* < 0.05. Different colors from green to red represent the *q* value. Round, triangular, and square shapes represent the biological process, cellular component, and molecular function, respectively. Different sizes of the shapes represent the gene count number in a GO term.

**Figure 6:**
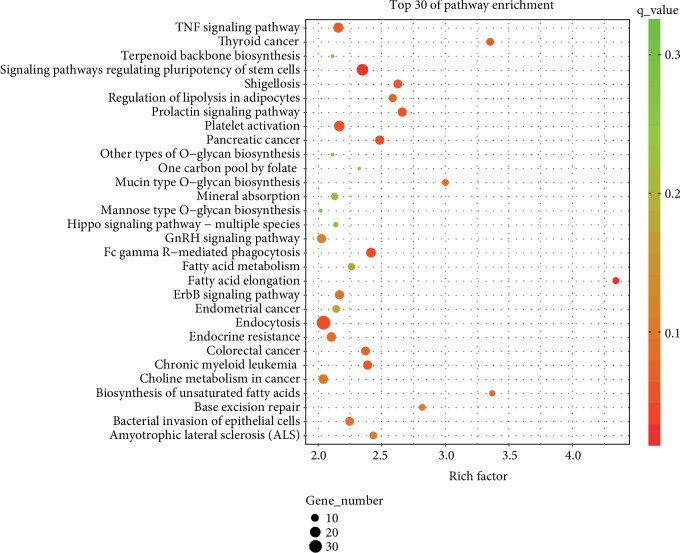
KEGG pathway analyses of miR-22-3p and miR-320a. The results show the associated target gene pathway of the two miRNAs. The enrichment *P* values were calculated by Fisher's exact test. Term/pathway in the vertical axis was drawn according to the enrich factor value of the pathway name in descending order. The horizontal axis represents the enrich factor, as follows: enrich factor = (dysregulated gene number in a pathway/total dysregulated gene number)/(gene number in a pathway in the database/total gene number in the database). Top 30 pathway terms were selected according to the enrich factor value. Selection standards included the gene number in a pathway 4 and *q* < 0.05. Different colors from green to red represent *P* value. Different sizes of the round shape represent gene count number in a pathway.

GO was used to analyze the predictive targets (supplementary [Supplementary-material supplementary-material-1]) of the two miRNAs and showed that a mass of genes was related with MAP kinase activity, lamin binding, G-protein-coupled glutamate receptor binding, G-protein *α*-subunit binding, and node of Ranvier (Figure 5). Pathway enrichment analysis showed that several pathways, including TNF signaling pathway, thyroid cancer, terpenoid backbone biosynthesis, signaling pathways regulating pluripotency of stem cells, and shigellosis, were mostly related to the two significantly increased miRNAs (Figure 6).

## 4. Discussion

Considering that the detection and treatment of endometriosis are often delayed due to the lack of symptoms and sensitive biomarkers, finding an effective noninvasive indicator to diagnose endometriosis in the early stages of the disease is urgently needed. miRNAs play important roles in biological function and endometriosis pathology [12, 23], and circulating miRNAs have been identified for diagnostic application in endometriosis [13, 14]. The stability of circulating free miRNAs and exosomal miRNAs is controversial. Tian et al. reported that there was no significant difference between plasma miRNAs and plasma-derived exosomal miRNAs from healthy people [24]. But more studies support the idea that exosomal RNAs are protected by RNase A treatment and the majority of free microRNAs are concentrated in exosomes [25, 26]. Therefore, free miRNAs in blood are often degraded by RNA enzyme. Therefore, free miRNAs may not accurately reflect the pathological differences. Exosome, which is a new biomarker for many diseases, protects miRNAs from being degraded. Serum exosomal miRNAs may be promising biomarkers for the diagnosis of many diseases, and no relevant research about the function of serum exosomal miRNAs as noninvasive biomarkers for endometriosis has been found. To the best of our knowledge, this report was the first to assess the diagnostic significance of circulating exosomal miRNAs in endometriosis.

In this paper, we first attempted to isolate and identify exosomes in serum. The results showed the exosome morphology, size distribution, and specific markers through TEM, NTA, and Western blot analysis, respectively, which were consistent with previous studies. This result indicated that we have successfully isolated exosomes from the serum. Then, we analyzed the serum exosomal miRNA expression profiles of the five pairs of endometriosis and negative control through miRNA microarray. The results showed that 24 serum exosomal miRNAs were significantly dysregulated. Afterward, we selected five miRNAs (i.e., miR-134-5p, miR-197-5p, miR-22-3p, miR-320a, miR-494-3p, and miR-939-5p) as candidate endometriosis biomarkers and performed qRT-PCR to validate their expression. miR-22-3p and miR-320a were significantly upregulated in the serum exosomes from patients with endometriosis. miR-22-3p, miR-320a, and their combination had high sensitivity and specificity, which increased the diagnostic effectiveness of endometriosis, by ROC analysis. These findings indicated that serum exosomal miR-22-3p and miR-320a are effective in diagnosing endometriosis.

The result of our study was inconsistent with previous studies about circulating miRNAs being biomarkers for endometriosis [27, 28]. Many factors, except that exosome could protect miRNA from being degraded by RNA enzyme, possibly influenced the findings. First, unbalanced distribution of menstrual cycle phases may affect the result. In different menstrual cycle, the activity of ectopic endometrium was different. Second, unbalanced distribution of the stage of endometriosis also may influence the result. Third, the differences of microarray methods and races may also affect the results. In fact, in most diseases, circulating free miRNAs and exosomal miRNAs are inconsistent.

miR-22-3p plays an important role in many important biological processes, including diabetic cardiomyopathy [29] and tumorigenesis [30], and is identified as a noninvasive biomarker for schizophrenia [31] and pancreatic cancer [32]. miR-22-3p affects the chemosensitivity of gastrointestinal stromal tumor cell lines to cisplatin through the PTEN/PI3K/Akt pathway [33], which is associated with cell proliferation in endometriosis in a previous article [34]. Hence, consistent with previous studies, miR-22-3p may play a key role in endometriosis development. Sommariva et al. reported that miR-320a serves as a potential novel circulating biomarker of arrhythmogenic cardiomyopathy [35]. miR-320a also regulates non-small-cell lung cancer metastasis and invasion via the PI3K/Akt pathway [36] and suppresses lung adenocarcinoma cell proliferation and metastasis by targeting STAT3 signal [37]. PI3K/Akt and STAT3 pathways are also involved in the pathogenesis of endometriosis. Our study found miR-22-3p and miR-320a may play an important role in endometriosis occurrence and development, which should be studied further.

The predicted target genes of miR-22-3p and miR-320a were studied through GO analysis. The results showed that specific biological processes, including MAP kinase activity, lamin binding, G-protein-coupled glutamate receptor binding, and G-protein alpha-subunit binding, and node of Ranvier, were enriched. This result was generally consistent with a previous study [38]. Nevertheless, additional research is still needed to determine the mechanisms under which the two miRNAs were increased in the endometriosis serum samples. The two increased miRNAs increased in several pathways, including the TNF signaling pathway, thyroid cancer, terpenoid backbone biosynthesis, signaling pathways regulating pluripotency of stem cells, and shigellosis, by pathway enrichment analysis. Tian et al. reported that miR-191 can inhibit the TNF-*α*-induced apoptosis of ovarian endometriosis by targeting DAPK1 [39]. Kodati et al. reported that shigellosis can trigger the initiation of immunological changes in the pelvic peritoneum, thereby causing endometriosis development [40]. These results are generally consistent with the findings of our pathway enrichment analysis. Meanwhile, thyroid cancer, terpenoid backbone biosynthesis, and signaling pathways regulating the pluripotency of stem cells were the newly identified pathway that may be attributed to the pathogenesis of endometriosis.

Although our study identified some potential exosomal miRNAs as biomarkers for endometriosis, it has several limitations. First, the number of subjects was relatively small. We only performed miRNA array on the five pairs of patients with endometriosis and negative individuals. Therefore, large-scale cohort studies are needed to analyze and confirm these results further. Second, the mechanism of these exosomal miRNAs affecting the diagnosis was not investigated. Finally, this study lacked functional experiments at the cellular and molecular levels to identify the relationship between these miRNAs and endometriosis. We analyzed the enriched signaling pathways with multiple identified miRNAs, but functional research on identified miRNAs and signaling transduction pathways should be carried out in the future.

In summary, through miRNA microarray assay and qRT-PCR analysis, serum exosomes from endometriosis have a unique miRNA expression profile. miR-22-3p and miR-320a in serum exosomes can be used as novel noninvasive diagnostic biomarkers for endometriosis. To the best of our knowledge, this study was the first report to compare the differences between miRNA analyses by using the serum exosomes from endometriosis. However, the mechanisms of these candidate miRNAs in the pathogenesis of endometriosis require further investigation.

## 5. Conclusion

Our results demonstrated that exosomal miR-22-3p and miR-320a were significantly increased in the sera of patients with endometriosis. The two miRNAs may be useful potential biomarkers for endometriosis diagnosis.

## Figures and Tables

**Table 1:** Twenty-four serum exosomal miRNAs were statistically dysregulated.

Upregulated	Fold change	*P* value	Downregulated	Fold change	*P* value
miR-197-5p	18.8110	0.0103	miR-134-5p	0.3552	0.0437
miR-22-3p	6.1246	0.0443	miR-3141	0.4499	0.0424
miR-320a	8.0516	0.0326	miR-4499	0.3828	0.0175
miR-320b	3.0118	0.0337	miR-6088	0.4598	0.0187
miR-3692-5p	3.2835	0.0275	miR-6165	0.4098	0.0309
miR-4476	3.0882	0.0479	miR-6728-5p	0.3894	0.0379
miR-4530	5.4256	0.0050			
miR-4532	2.5511	0.0024			
miR-4721	5.4209	0.0316			
miR-4758-5p	3.9224	0.0013			
miR-494-3p	4.4079	0.0076			
miR-6126	2.4148	0.0379			
miR-6734-5p	2.3407	0.0431			
miR-6776-5p	2.0961	0.0174			
miR-6780b-5p	4.7209	0.0496			
miR-6785-5p	15.4207	0.0302			
miR-6791-5p	2.6535	0.0417			
miR-939-5p	2.1248	0.0088			

**Table 2:** Association of exosomal miR-22-3p and miR-320a expression with the clinicopathological characteristics of endometriosis patients.

Characteristic	Total number	Exosomal miR-22-3p		*P* value	Exosomal miR-320a		*P* value
		Low-expression group	High-expression group		Low-expression group	High-expression group	
Total number	20	10	10		10	10	
Age	20	29.78 ± 4.44	28.96 ± 4.58	0.758	28.71 ± 4.94	28.32 ± 4.70	0.642
Pain intensity (VAS)	20	5.12 ± 2.37	4.98 ± 2.55	0.345	5.33 ± 2.70	5.46 ± 2.91	0.227
CA-125 (IU/ml)	20	73.25 ± 41.75	188.47 ± 78.75	<0.01	68.79 ± 48.47	179.97 ± 83.43	<0.01
Dysmenorrhea	20	5/10 (70%)	7/10 (60%)	0.648	4/10 (60%)	8/10 (70%)	0.169
DIE	20	1/10 (10%)	7/10 (70%)	<0.05	3/10 (30%)	5/10 (50%)	0.648

Data are expressed as mean ± standard deviation or *n* (%). DIE: deeply infiltrating endometriosis; VAS: visual analogue scale.

## Data Availability

The datasets analyzed during this study are available from the corresponding author on reasonable request.

## Supplementary Materials

Supplementary Materials Table 1 is the putative targets of miR-22-3p and miR-320a. The first column is the name of miRNA. The second column is the gene name of the putative targets. The last four columns are the databases that are used to predict the putative targets.
