# Supplementary material for: Serum Exosomal MicroRNAs as Potential Circulating Biomarkers for Endometriosis
Source: Dis Markers. 2020 Jan 23;2020:2456340. doi: 10.1155/2020/2456340 (PMC7008302; doi:10.1155/2020/2456340)
Supplement: Supplementary Materials — Table 1 is the putative targets of miR-22-3p and miR-320a. The first column is the name of miRNA. The second column is the gene name of the putative targets. The last four columns are the databases that are used to predict the putative targets. [file 2456340.f1.docx]

| mirna | gene_symbol | TargetScan | miRDB | miRTarbase | Tarbase |
| --- | --- | --- | --- | --- | --- |
| hsa-miR-320a | A2ML1 | - | - | - | + |
| hsa-miR-320a | AAR2 | - | - | - | + |
| hsa-miR-320a | AASDHPPT | - | - | - | + |
| hsa-miR-320a | ABCA1 | - | - | - | + |
| hsa-miR-320a | ABCA12 | - | - | - | + |
| hsa-miR-320a | ABCA2 | - | - | + | - |
| hsa-miR-320a | ABCA4 | - | - | - | + |
| hsa-miR-320a | ABCA5 | - | - | - | + |
| hsa-miR-320a | ABCB10 | - | - | - | + |
| hsa-miR-320a | ABCD2 | - | - | - | + |
| hsa-miR-320a | ABCD3 | - | - | - | + |
| hsa-miR-320a | ABCE1 | - | - | - | + |
| hsa-miR-320a | ABCF2 | - | - | + | - |
| hsa-miR-320a | ABHD10 | - | - | - | + |
| hsa-miR-320a | ABHD13 | - | + | - | + |
| hsa-miR-320a | ABHD16A | - | - | + | - |
| hsa-miR-320a | ABHD2 | - | - | - | + |
| hsa-miR-320a | ABHD4 | - | - | - | + |
| hsa-miR-320a | ABL2 | - | - | - | + |
| hsa-miR-320a | ABLIM1 | - | - | + | + |
| hsa-miR-320a | ABR | - | - | - | + |
| hsa-miR-320a | AC003682.1 | - | - | - | + |
| hsa-miR-320a | AC005754.1 | - | - | - | + |
| hsa-miR-320a | AC007390.5 | - | - | - | + |
| hsa-miR-320a | AC007405.2 | - | - | - | + |
| hsa-miR-320a | AC012123.1 | + | - | - | - |
| hsa-miR-320a | AC015804.1 | - | - | - | + |
| hsa-miR-320a | AC069547.2 | + | - | - | - |
| hsa-miR-320a | AC084121.16 | - | - | - | + |
| hsa-miR-320a | ACADSB | - | - | - | + |
| hsa-miR-320a | ACAP2 | - | - | - | + |
| hsa-miR-320a | ACBD3 | + | + | - | + |
| hsa-miR-320a | ACBD5 | - | - | - | + |
| hsa-miR-320a | ACER3 | - | - | - | + |
| hsa-miR-320a | ACOT13 | - | - | - | + |
| hsa-miR-320a | ACOT7 | + | - | - | - |
| hsa-miR-320a | ACOX1 | - | - | - | + |
| hsa-miR-320a | ACPP | - | - | + | + |
| hsa-miR-320a | ACSL4 | - | - | - | + |
| hsa-miR-320a | ACSS1 | - | - | - | + |
| hsa-miR-320a | ACTB | - | - | + | - |
| hsa-miR-320a | ACTBL2 | - | - | + | + |
| hsa-miR-320a | ACTC1 | - | - | - | + |
| hsa-miR-320a | ACTG1 | - | - | + | - |
| hsa-miR-320a | ACTN4 | - | - | + | - |
| hsa-miR-320a | ACTR2 | + | - | - | + |
| hsa-miR-320a | ACTR3 | - | - | - | + |
| hsa-miR-320a | ACTR8 | - | - | - | + |
| hsa-miR-320a | ACVR1C | - | - | - | + |
| hsa-miR-320a | ADAL | - | + | - | - |
| hsa-miR-320a | ADAM10 | - | + | - | - |
| hsa-miR-320a | ADAM12 | - | - | - | + |
| hsa-miR-320a | ADAM17 | - | - | - | + |
| hsa-miR-320a | ADAM22 | - | - | - | + |
| hsa-miR-320a | ADAM28 | - | - | - | + |
| hsa-miR-320a | ADAM8 | - | - | - | + |
| hsa-miR-320a | ADAMTS1 | - | - | - | + |
| hsa-miR-320a | ADAMTS12 | - | - | - | + |
| hsa-miR-320a | ADAMTS15 | - | - | - | + |
| hsa-miR-320a | ADAMTS19 | - | - | - | + |
| hsa-miR-320a | ADAMTS3 | - | - | - | + |
| hsa-miR-320a | ADAMTS5 | - | - | - | + |
| hsa-miR-320a | ADAMTSL1 | - | - | - | + |
| hsa-miR-320a | ADAMTSL3 | - | - | - | + |
| hsa-miR-320a | ADCY1 | - | - | + | - |
| hsa-miR-320a | ADCY10 | - | - | - | + |
| hsa-miR-320a | ADCY3 | - | - | - | + |
| hsa-miR-320a | ADCY5 | - | - | - | + |
| hsa-miR-320a | ADCY9 | - | - | - | + |
| hsa-miR-320a | ADCYAP1 | - | - | - | + |
| hsa-miR-320a | ADCYAP1R1 | - | - | - | + |
| hsa-miR-320a | ADGRB3 | - | - | - | + |
| hsa-miR-320a | ADGRG6 | - | - | - | + |
| hsa-miR-320a | ADGRV1 | - | + | - | - |
| hsa-miR-320a | ADH5 | - | - | - | + |
| hsa-miR-320a | ADIPOR1 | - | - | - | + |
| hsa-miR-320a | ADK | - | - | - | + |
| hsa-miR-320a | ADM2 | - | - | - | + |
| hsa-miR-320a | ADNP2 | - | - | - | + |
| hsa-miR-320a | ADO | - | - | - | + |
| hsa-miR-320a | ADRA1D | - | - | - | + |
| hsa-miR-320a | ADRA2A | - | - | - | + |
| hsa-miR-320a | ADSS | - | - | - | + |
| hsa-miR-320a | AEN | - | - | - | + |
| hsa-miR-320a | AFAP1 | - | + | - | - |
| hsa-miR-320a | AFF1 | - | - | - | + |
| hsa-miR-320a | AFF4 | - | + | - | + |
| hsa-miR-320a | AFTPH | - | - | - | + |
| hsa-miR-320a | AGAP1 | - | + | - | + |
| hsa-miR-320a | AGBL5 | - | - | - | + |
| hsa-miR-320a | AGFG1 | - | - | - | + |
| hsa-miR-320a | AGGF1 | - | - | - | + |
| hsa-miR-320a | AGO1 | - | - | + | - |
| hsa-miR-320a | AGO4 | - | - | - | + |
| hsa-miR-320a | AGPAT1 | - | - | - | + |
| hsa-miR-320a | AGPAT6 | - | - | - | + |
| hsa-miR-320a | AGPAT9 | - | - | - | + |
| hsa-miR-320a | AGPS | - | + | - | + |
| hsa-miR-320a | AGRN | - | - | - | + |
| hsa-miR-320a | AGTPBP1 | - | - | - | + |
| hsa-miR-320a | AHCTF1 | - | - | - | + |
| hsa-miR-320a | AHCYL1 | - | - | - | + |
| hsa-miR-320a | AHCYL2 | - | - | - | + |
| hsa-miR-320a | AHDC1 | - | - | - | + |
| hsa-miR-320a | AHI1 | - | - | - | + |
| hsa-miR-320a | AHNAK | - | - | - | + |
| hsa-miR-320a | AHR | - | - | - | + |
| hsa-miR-320a | AHRR | - | - | - | + |
| hsa-miR-320a | AIDA | - | - | - | + |
| hsa-miR-320a | AIM1 | - | + | - | + |
| hsa-miR-320a | AIMP1 | - | - | + | + |
| hsa-miR-320a | AK2 | + | - | + | + |
| hsa-miR-320a | AK4 | - | + | - | + |
| hsa-miR-320a | AK5 | - | - | - | + |
| hsa-miR-320a | AK7 | - | - | - | + |
| hsa-miR-320a | AKAP10 | - | - | - | + |
| hsa-miR-320a | AKAP11 | - | - | - | + |
| hsa-miR-320a | AKAP12 | - | - | - | + |
| hsa-miR-320a | AKAP2 | - | - | - | + |
| hsa-miR-320a | AKIRIN1 | - | - | - | + |
| hsa-miR-320a | AKT2 | - | - | - | + |
| hsa-miR-320a | AKT3 | + | + | - | - |
| hsa-miR-320a | AKTIP | - | - | - | + |
| hsa-miR-320a | AL359392.1 | - | - | - | + |
| hsa-miR-320a | AL360004.1 | - | - | - | + |
| hsa-miR-320a | AL592284.1 | + | - | - | - |
| hsa-miR-320a | ALAD | - | - | - | + |
| hsa-miR-320a | ALCAM | - | - | - | + |
| hsa-miR-320a | ALDH1L2 | - | - | - | + |
| hsa-miR-320a | ALDH5A1 | - | - | - | + |
| hsa-miR-320a | ALDH6A1 | - | - | - | + |
| hsa-miR-320a | ALDH7A1 | - | - | - | + |
| hsa-miR-320a | ALG13 | - | + | - | + |
| hsa-miR-320a | ALG6 | - | - | - | + |
| hsa-miR-320a | ALKBH1 | - | - | - | + |
| hsa-miR-320a | ALKBH5 | - | + | - | + |
| hsa-miR-320a | ALKBH6 | - | - | + | - |
| hsa-miR-320a | ALOX12 | + | + | - | + |
| hsa-miR-320a | ALPK3 | - | - | - | + |
| hsa-miR-320a | ALS2CR8 | - | - | - | + |
| hsa-miR-320a | ALX4 | - | - | - | + |
| hsa-miR-320a | AMACR | - | - | - | + |
| hsa-miR-320a | AMBN | - | - | - | + |
| hsa-miR-320a | AMELX | - | - | + | - |
| hsa-miR-320a | AMER1 | - | - | - | + |
| hsa-miR-320a | AMER2 | - | - | - | + |
| hsa-miR-320a | AMIGO1 | - | - | - | + |
| hsa-miR-320a | AMIGO2 | - | - | - | + |
| hsa-miR-320a | AMOT | - | - | - | + |
| hsa-miR-320a | AMOTL1 | - | - | - | + |
| hsa-miR-320a | AMPD3 | - | - | - | + |
| hsa-miR-320a | ANAPC1 | - | - | - | + |
| hsa-miR-320a | ANAPC16 | - | - | - | + |
| hsa-miR-320a | ANGEL1 | + | - | - | - |
| hsa-miR-320a | ANGEL2 | - | - | - | + |
| hsa-miR-320a | ANGPT2 | - | - | - | + |
| hsa-miR-320a | ANGPTL1 | - | - | - | + |
| hsa-miR-320a | ANK1 | - | - | - | + |
| hsa-miR-320a | ANKFY1 | - | - | - | + |
| hsa-miR-320a | ANKH | - | + | - | + |
| hsa-miR-320a | ANKHD1 | - | - | + | - |
| hsa-miR-320a | ANKIB1 | - | - | - | + |
| hsa-miR-320a | ANKLE2 | - | - | - | + |
| hsa-miR-320a | ANKRD13A | - | + | - | + |
| hsa-miR-320a | ANKRD13C | - | - | - | + |
| hsa-miR-320a | ANKRD17 | - | - | - | + |
| hsa-miR-320a | ANKRD20A2 | - | - | - | + |
| hsa-miR-320a | ANKRD20A4 | - | - | - | + |
| hsa-miR-320a | ANKRD28 | - | - | - | + |
| hsa-miR-320a | ANKRD32 | - | - | - | + |
| hsa-miR-320a | ANKRD34B | - | - | - | + |
| hsa-miR-320a | ANKRD39 | - | - | + | - |
| hsa-miR-320a | ANKRD40 | - | - | - | + |
| hsa-miR-320a | ANKRD42 | - | - | - | + |
| hsa-miR-320a | ANKRD46 | - | - | - | + |
| hsa-miR-320a | ANKRD52 | - | - | + | + |
| hsa-miR-320a | ANKS1A | - | + | - | + |
| hsa-miR-320a | ANO3 | - | - | - | + |
| hsa-miR-320a | ANO5 | - | - | - | + |
| hsa-miR-320a | ANO6 | + | - | - | + |
| hsa-miR-320a | ANOS1 | - | - | - | + |
| hsa-miR-320a | ANP32A | - | - | + | - |
| hsa-miR-320a | ANP32B | - | - | + | + |
| hsa-miR-320a | ANTXR1 | - | - | - | + |
| hsa-miR-320a | ANTXR2 | - | - | - | + |
| hsa-miR-320a | ANXA11 | - | - | + | - |
| hsa-miR-320a | ANXA7 | - | - | - | + |
| hsa-miR-320a | AOC2 | - | - | - | + |
| hsa-miR-320a | AOX1 | - | - | - | + |
| hsa-miR-320a | AP1AR | - | - | - | + |
| hsa-miR-320a | AP1G1 | - | - | - | + |
| hsa-miR-320a | AP3M1 | + | + | - | + |
| hsa-miR-320a | AP4E1 | - | - | - | + |
| hsa-miR-320a | APAF1 | - | - | - | + |
| hsa-miR-320a | APBA1 | - | - | - | + |
| hsa-miR-320a | APBB2 | - | - | - | + |
| hsa-miR-320a | APCDD1L | - | - | - | + |
| hsa-miR-320a | APEX1 | + | - | - | - |
| hsa-miR-320a | APH1B | - | - | - | + |
| hsa-miR-320a | API5 | - | - | - | + |
| hsa-miR-320a | APOBEC4 | - | - | - | + |
| hsa-miR-320a | APPL1 | - | + | - | + |
| hsa-miR-320a | APTX | + | - | - | - |
| hsa-miR-320a | AQP1 | - | - | + | + |
| hsa-miR-320a | AQP11 | - | - | - | + |
| hsa-miR-320a | AQP3 | - | - | - | + |
| hsa-miR-320a | AQP4 | - | - | + | + |
| hsa-miR-320a | AR | - | - | - | + |
| hsa-miR-320a | ARF1 | - | + | + | + |
| hsa-miR-320a | ARF3 | - | - | + | + |
| hsa-miR-320a | ARF6 | - | - | - | + |
| hsa-miR-320a | ARFGEF2 | - | + | - | + |
| hsa-miR-320a | ARFIP1 | + | + | - | + |
| hsa-miR-320a | ARHGAP17 | - | - | + | - |
| hsa-miR-320a | ARHGAP18 | - | - | - | + |
| hsa-miR-320a | ARHGAP20 | - | - | - | + |
| hsa-miR-320a | ARHGAP24 | - | - | - | + |
| hsa-miR-320a | ARHGAP26 | - | - | + | + |
| hsa-miR-320a | ARHGAP28 | - | - | - | + |
| hsa-miR-320a | ARHGAP31 | - | + | - | + |
| hsa-miR-320a | ARHGAP42 | - | - | - | + |
| hsa-miR-320a | ARHGAP5 | - | + | - | - |
| hsa-miR-320a | ARHGEF12 | - | - | - | + |
| hsa-miR-320a | ARHGEF2 | - | - | + | - |
| hsa-miR-320a | ARHGEF26 | - | - | - | + |
| hsa-miR-320a | ARHGEF37 | - | - | - | + |
| hsa-miR-320a | ARHGEF38 | - | + | - | + |
| hsa-miR-320a | ARHGEF6 | - | - | - | + |
| hsa-miR-320a | ARHGEF7 | - | - | - | + |
| hsa-miR-320a | ARHGEF9 | - | - | - | + |
| hsa-miR-320a | ARID4B | - | - | - | + |
| hsa-miR-320a | ARID5B | - | - | + | + |
| hsa-miR-320a | ARL1 | - | - | - | + |
| hsa-miR-320a | ARL10 | + | - | + | - |
| hsa-miR-320a | ARL11 | - | - | - | + |
| hsa-miR-320a | ARL14EPL | + | - | - | - |
| hsa-miR-320a | ARL15 | - | - | - | + |
| hsa-miR-320a | ARL2BP | - | - | - | + |
| hsa-miR-320a | ARL3 | - | - | + | + |
| hsa-miR-320a | ARL4A | + | - | - | + |
| hsa-miR-320a | ARL4C | - | - | - | + |
| hsa-miR-320a | ARL5B | - | - | - | + |
| hsa-miR-320a | ARL6IP1 | - | - | - | + |
| hsa-miR-320a | ARL8B | - | + | - | - |
| hsa-miR-320a | ARL9 | - | - | + | - |
| hsa-miR-320a | ARMC1 | - | + | - | + |
| hsa-miR-320a | ARMCX2 | + | + | - | + |
| hsa-miR-320a | ARMCX3 | - | - | - | + |
| hsa-miR-320a | ARNT | - | - | - | + |
| hsa-miR-320a | ARNT2 | - | - | - | + |
| hsa-miR-320a | ARNTL2 | - | - | - | + |
| hsa-miR-320a | ARPC5 | + | + | - | - |
| hsa-miR-320a | ARPP19 | - | + | + | + |
| hsa-miR-320a | ARRDC2 | - | - | - | + |
| hsa-miR-320a | ARRDC3 | - | - | - | + |
| hsa-miR-320a | ARRDC4 | - | - | - | + |
| hsa-miR-320a | ARRDC5 | - | - | - | + |
| hsa-miR-320a | ARSB | - | - | - | + |
| hsa-miR-320a | ARSI | - | - | - | + |
| hsa-miR-320a | ARSK | - | - | - | + |
| hsa-miR-320a | ARX | - | - | - | + |
| hsa-miR-320a | ASAH2 | - | - | - | + |
| hsa-miR-320a | ASAH2B | - | - | - | + |
| hsa-miR-320a | ASAH2C | + | - | - | - |
| hsa-miR-320a | ASB1 | - | - | - | + |
| hsa-miR-320a | ASB2 | - | - | - | + |
| hsa-miR-320a | ASB5 | + | + | - | + |
| hsa-miR-320a | ASB6 | - | - | + | - |
| hsa-miR-320a | ASB7 | - | - | - | + |
| hsa-miR-320a | ASB8 | - | - | - | + |
| hsa-miR-320a | ASCC1 | - | - | - | + |
| hsa-miR-320a | ASCC2 | - | - | + | - |
| hsa-miR-320a | ASF1A | - | - | - | + |
| hsa-miR-320a | ASF1B | - | - | - | + |
| hsa-miR-320a | ASH2L | + | - | - | - |
| hsa-miR-320a | ASIC2 | - | - | - | + |
| hsa-miR-320a | ASPN | - | - | - | + |
| hsa-miR-320a | ASS1 | - | - | - | + |
| hsa-miR-320a | ASTN1 | - | - | - | + |
| hsa-miR-320a | ASXL1 | - | - | - | + |
| hsa-miR-320a | ASXL2 | - | - | - | + |
| hsa-miR-320a | ASXL3 | - | - | - | + |
| hsa-miR-320a | ATAD2B | - | - | - | + |
| hsa-miR-320a | ATAD5 | - | - | - | + |
| hsa-miR-320a | ATE1 | - | - | - | + |
| hsa-miR-320a | ATF1 | - | - | - | + |
| hsa-miR-320a | ATF2 | - | - | - | + |
| hsa-miR-320a | ATF6 | - | - | - | + |
| hsa-miR-320a | ATF7 | - | - | - | + |
| hsa-miR-320a | ATF7IP | - | - | + | - |
| hsa-miR-320a | ATF7IP2 | - | + | - | - |
| hsa-miR-320a | ATG14 | - | + | - | + |
| hsa-miR-320a | ATG2B | - | + | - | + |
| hsa-miR-320a | ATG4C | - | - | - | + |
| hsa-miR-320a | ATG5 | - | - | - | + |
| hsa-miR-320a | ATG7 | - | + | - | + |
| hsa-miR-320a | ATHL1 | - | + | - | + |
| hsa-miR-320a | ATL1 | - | - | - | + |
| hsa-miR-320a | ATL3 | - | + | - | + |
| hsa-miR-320a | ATM | - | - | - | + |
| hsa-miR-320a | ATMIN | - | - | - | + |
| hsa-miR-320a | ATP11A | - | - | - | + |
| hsa-miR-320a | ATP11B | - | - | - | + |
| hsa-miR-320a | ATP1A1 | - | - | + | - |
| hsa-miR-320a | ATP2A3 | - | + | - | + |
| hsa-miR-320a | ATP2B1 | - | - | - | + |
| hsa-miR-320a | ATP2B3 | - | - | - | + |
| hsa-miR-320a | ATP2B4 | - | - | - | + |
| hsa-miR-320a | ATP2C1 | - | - | - | + |
| hsa-miR-320a | ATP5B | - | - | + | - |
| hsa-miR-320a | ATP5D | - | - | - | + |
| hsa-miR-320a | ATP5G1 | - | - | - | + |
| hsa-miR-320a | ATP5SL | - | - | - | + |
| hsa-miR-320a | ATP6V0A2 | - | - | - | + |
| hsa-miR-320a | ATP6V0D2 | - | - | - | + |
| hsa-miR-320a | ATP6V1A | - | + | - | + |
| hsa-miR-320a | ATP6V1B2 | - | - | + | - |
| hsa-miR-320a | ATP6V1C1 | - | - | - | + |
| hsa-miR-320a | ATP7A | - | - | + | + |
| hsa-miR-320a | ATP8B1 | - | - | - | + |
| hsa-miR-320a | ATP9A | - | - | - | + |
| hsa-miR-320a | ATPAF2 | - | - | + | - |
| hsa-miR-320a | ATPIF1 | - | - | - | + |
| hsa-miR-320a | ATRNL1 | - | - | - | + |
| hsa-miR-320a | ATRX | - | + | - | + |
| hsa-miR-320a | ATXN1 | - | - | + | + |
| hsa-miR-320a | ATXN10 | - | - | - | + |
| hsa-miR-320a | ATXN1L | - | - | - | + |
| hsa-miR-320a | ATXN3 | - | - | - | + |
| hsa-miR-320a | ATXN7 | - | - | - | + |
| hsa-miR-320a | ATXN7L1 | - | - | - | + |
| hsa-miR-320a | ATXN7L3B | - | - | + | - |
| hsa-miR-320a | AUNIP | - | - | - | + |
| hsa-miR-320a | AUP1 | - | - | + | - |
| hsa-miR-320a | AUTS2 | - | - | - | + |
| hsa-miR-320a | AVL9 | - | - | - | + |
| hsa-miR-320a | AWAT2 | - | - | - | + |
| hsa-miR-320a | AXIN1 | - | - | + | - |
| hsa-miR-320a | AZIN1 | - | + | - | + |
| hsa-miR-320a | B3GALT2 | - | - | - | + |
| hsa-miR-320a | B3GALT5 | - | - | - | + |
| hsa-miR-320a | B3GALTL | - | - | - | + |
| hsa-miR-320a | B3GNT5 | - | - | - | + |
| hsa-miR-320a | B4GALT4 | - | + | - | - |
| hsa-miR-320a | B4GALT6 | - | - | - | + |
| hsa-miR-320a | BAALC | - | - | - | + |
| hsa-miR-320a | BACE1 | - | - | - | + |
| hsa-miR-320a | BACH2 | - | - | - | + |
| hsa-miR-320a | BAG4 | - | - | - | + |
| hsa-miR-320a | BAG5 | - | - | - | + |
| hsa-miR-320a | BAHD1 | - | - | - | + |
| hsa-miR-320a | BAIAP3 | - | - | - | + |
| hsa-miR-320a | BAMBI | - | - | - | + |
| hsa-miR-320a | BANF1 | - | - | - | + |
| hsa-miR-320a | BANP | + | + | + | + |
| hsa-miR-320a | BARD1 | - | - | - | + |
| hsa-miR-320a | BASP1 | - | - | - | + |
| hsa-miR-320a | BAZ1A | + | + | - | + |
| hsa-miR-320a | BBIP1 | - | - | - | + |
| hsa-miR-320a | BBS12 | - | - | - | + |
| hsa-miR-320a | BBS5 | - | - | - | + |
| hsa-miR-320a | BBS7 | - | - | - | + |
| hsa-miR-320a | BBX | - | - | - | + |
| hsa-miR-320a | BCAP29 | + | - | - | + |
| hsa-miR-320a | BCAR3 | - | - | - | + |
| hsa-miR-320a | BCAT1 | - | + | - | + |
| hsa-miR-320a | BCL11B | - | - | - | + |
| hsa-miR-320a | BCL2L13 | - | - | - | + |
| hsa-miR-320a | BCL7B | - | - | + | + |
| hsa-miR-320a | BCLAF1 | - | - | - | + |
| hsa-miR-320a | BCOR | - | - | + | - |
| hsa-miR-320a | BCR | - | - | - | + |
| hsa-miR-320a | BECN1 | - | - | - | + |
| hsa-miR-320a | BEND4 | - | + | - | + |
| hsa-miR-320a | BEST4 | - | - | - | + |
| hsa-miR-320a | BET1 | - | - | - | + |
| hsa-miR-320a | BET1L | - | - | + | - |
| hsa-miR-320a | BFAR | - | - | - | + |
| hsa-miR-320a | BHLHE22 | - | - | - | + |
| hsa-miR-320a | BHLHE40 | - | - | - | + |
| hsa-miR-320a | BHLHE41 | - | - | - | + |
| hsa-miR-320a | BHMT | - | - | - | + |
| hsa-miR-320a | BICD1 | - | - | - | + |
| hsa-miR-320a | BICD2 | - | - | - | + |
| hsa-miR-320a | BIN3 | - | - | - | + |
| hsa-miR-320a | BIRC2 | - | - | - | + |
| hsa-miR-320a | BIRC5 | - | - | - | + |
| hsa-miR-320a | BIRC6 | - | - | - | + |
| hsa-miR-320a | BLCAP | - | - | - | + |
| hsa-miR-320a | BLOC1S3 | - | - | - | + |
| hsa-miR-320a | BLOC1S5 | - | + | - | - |
| hsa-miR-320a | BLOC1S6 | - | - | - | + |
| hsa-miR-320a | BMI1 | - | - | + | + |
| hsa-miR-320a | BMP2 | - | - | - | + |
| hsa-miR-320a | BMP3 | - | - | - | + |
| hsa-miR-320a | BMP7 | - | - | - | + |
| hsa-miR-320a | BMPR1A | + | + | - | - |
| hsa-miR-320a | BMPR1B | - | - | - | + |
| hsa-miR-320a | BMS1 | - | - | - | + |
| hsa-miR-320a | BNC1 | - | + | - | + |
| hsa-miR-320a | BNC2 | - | - | - | + |
| hsa-miR-320a | BNIP2 | - | - | - | + |
| hsa-miR-320a | BNIP3 | + | + | - | - |
| hsa-miR-320a | BNIP3L | + | - | - | + |
| hsa-miR-320a | BOD1L1 | - | + | - | - |
| hsa-miR-320a | BOD1L2 | - | - | - | + |
| hsa-miR-320a | BOLL | - | - | - | + |
| hsa-miR-320a | BORA | - | - | - | + |
| hsa-miR-320a | BPNT1 | + | - | - | + |
| hsa-miR-320a | BRD3 | - | - | - | + |
| hsa-miR-320a | BRD4 | - | - | + | + |
| hsa-miR-320a | BRE | - | - | - | + |
| hsa-miR-320a | BRINP3 | - | - | - | + |
| hsa-miR-320a | BRIP1 | - | - | - | + |
| hsa-miR-320a | BROX | + | + | - | + |
| hsa-miR-320a | BRWD3 | - | + | - | + |
| hsa-miR-320a | BSN | - | - | - | + |
| hsa-miR-320a | BSPRY | - | - | - | + |
| hsa-miR-320a | BTAF1 | - | - | - | + |
| hsa-miR-320a | BTBD1 | - | - | - | + |
| hsa-miR-320a | BTBD11 | - | - | - | + |
| hsa-miR-320a | BTBD9 | - | - | - | + |
| hsa-miR-320a | BTF3 | - | - | - | + |
| hsa-miR-320a | BTF3L4 | - | - | - | + |
| hsa-miR-320a | BTG1 | - | - | - | + |
| hsa-miR-320a | BTG3 | - | - | - | + |
| hsa-miR-320a | BTLA | - | - | - | + |
| hsa-miR-320a | BTN1A1 | - | - | - | + |
| hsa-miR-320a | BTRC | - | - | + | + |
| hsa-miR-320a | BVES | - | - | - | + |
| hsa-miR-320a | BX255923.1 | + | - | - | - |
| hsa-miR-320a | BYSL | - | - | + | - |
| hsa-miR-320a | C10orf12 | - | - | - | + |
| hsa-miR-320a | C10orf67 | - | - | - | + |
| hsa-miR-320a | C10orf72 | - | - | - | + |
| hsa-miR-320a | C11orf24 | - | - | - | + |
| hsa-miR-320a | C11orf45 | - | - | - | + |
| hsa-miR-320a | C11orf52 | - | - | - | + |
| hsa-miR-320a | C11orf54 | + | + | - | - |
| hsa-miR-320a | C11orf83 | - | - | - | + |
| hsa-miR-320a | C11orf87 | - | - | - | + |
| hsa-miR-320a | C11orf9 | - | - | - | + |
| hsa-miR-320a | C12orf36 | - | - | - | + |
| hsa-miR-320a | C12orf52 | - | - | + | - |
| hsa-miR-320a | C12orf61 | - | - | - | + |
| hsa-miR-320a | C12orf66 | - | - | - | + |
| hsa-miR-320a | C12orf68 | - | - | - | + |
| hsa-miR-320a | C12orf73 | - | - | - | + |
| hsa-miR-320a | C12orf74 | - | - | - | + |
| hsa-miR-320a | C13orf33 | - | - | - | + |
| hsa-miR-320a | C14orf105 | - | - | - | + |
| hsa-miR-320a | C14orf118 | - | - | - | + |
| hsa-miR-320a | C14orf132 | - | + | - | - |
| hsa-miR-320a | C14orf28 | - | - | - | + |
| hsa-miR-320a | C14orf45 | - | - | - | + |
| hsa-miR-320a | C15orf24 | - | - | - | + |
| hsa-miR-320a | C15orf26 | + | - | - | - |
| hsa-miR-320a | C15orf41 | - | + | - | + |
| hsa-miR-320a | C15orf53 | - | - | - | + |
| hsa-miR-320a | C16orf52 | - | - | - | + |
| hsa-miR-320a | C16orf70 | - | - | - | + |
| hsa-miR-320a | C17orf101 | - | - | - | + |
| hsa-miR-320a | C17orf107 | - | - | - | + |
| hsa-miR-320a | C17orf109 | - | - | - | + |
| hsa-miR-320a | C17orf51 | - | - | - | + |
| hsa-miR-320a | C17orf72 | - | - | - | + |
| hsa-miR-320a | C17orf80 | - | - | - | + |
| hsa-miR-320a | C18orf42 | - | + | - | - |
| hsa-miR-320a | C18orf54 | - | - | - | + |
| hsa-miR-320a | C19orf40 | + | - | - | - |
| hsa-miR-320a | C19orf48 | - | - | - | + |
| hsa-miR-320a | C1GALT1 | - | - | - | + |
| hsa-miR-320a | C1QL3 | - | - | - | + |
| hsa-miR-320a | C1QTNF7 | - | - | - | + |
| hsa-miR-320a | C1QTNF9 | + | - | - | + |
| hsa-miR-320a | C1RL | - | - | - | + |
| hsa-miR-320a | C1orf109 | - | - | - | + |
| hsa-miR-320a | C1orf115 | - | - | - | + |
| hsa-miR-320a | C1orf129 | - | - | - | + |
| hsa-miR-320a | C1orf145 | + | - | - | + |
| hsa-miR-320a | C1orf185 | - | - | - | + |
| hsa-miR-320a | C1orf198 | - | - | - | + |
| hsa-miR-320a | C1orf204 | - | - | - | + |
| hsa-miR-320a | C1orf21 | - | - | - | + |
| hsa-miR-320a | C1orf213 | - | - | - | + |
| hsa-miR-320a | C1orf226 | - | - | - | + |
| hsa-miR-320a | C1orf43 | - | - | - | + |
| hsa-miR-320a | C1orf95 | - | - | - | + |
| hsa-miR-320a | C1orf98 | - | - | - | + |
| hsa-miR-320a | C20orf112 | - | - | - | + |
| hsa-miR-320a | C20orf118 | - | - | - | + |
| hsa-miR-320a | C20orf152 | - | - | - | + |
| hsa-miR-320a | C20orf160 | - | - | - | + |
| hsa-miR-320a | C20orf194 | - | - | - | + |
| hsa-miR-320a | C20orf196 | - | - | + | - |
| hsa-miR-320a | C20orf24 | - | - | + | - |
| hsa-miR-320a | C20orf27 | - | - | + | - |
| hsa-miR-320a | C21orf62 | - | + | - | - |
| hsa-miR-320a | C21orf91 | - | - | - | + |
| hsa-miR-320a | C22orf32 | - | - | - | + |
| hsa-miR-320a | C2CD2 | - | - | + | + |
| hsa-miR-320a | C2orf42 | - | - | - | + |
| hsa-miR-320a | C2orf49 | - | - | - | + |
| hsa-miR-320a | C2orf56 | - | - | - | + |
| hsa-miR-320a | C2orf69 | - | - | - | + |
| hsa-miR-320a | C2orf71 | - | - | - | + |
| hsa-miR-320a | C2orf88 | - | - | - | + |
| hsa-miR-320a | C2orf91 | - | - | - | + |
| hsa-miR-320a | C3orf14 | - | - | - | + |
| hsa-miR-320a | C3orf19 | - | - | - | + |
| hsa-miR-320a | C3orf52 | - | - | - | + |
| hsa-miR-320a | C3orf70 | - | - | - | + |
| hsa-miR-320a | C3orf80 | - | - | - | + |
| hsa-miR-320a | C4BPA | - | - | - | + |
| hsa-miR-320a | C4orf23 | - | - | - | + |
| hsa-miR-320a | C4orf33 | + | - | - | - |
| hsa-miR-320a | C5 | - | - | - | + |
| hsa-miR-320a | C5orf15 | + | - | - | + |
| hsa-miR-320a | C5orf22 | - | + | - | - |
| hsa-miR-320a | C5orf24 | - | - | - | + |
| hsa-miR-320a | C5orf30 | - | - | - | + |
| hsa-miR-320a | C5orf4 | - | - | - | + |
| hsa-miR-320a | C5orf43 | - | - | - | + |
| hsa-miR-320a | C5orf47 | + | - | - | + |
| hsa-miR-320a | C5orf51 | - | + | + | + |
| hsa-miR-320a | C5orf55 | - | - | - | + |
| hsa-miR-320a | C5orf62 | - | - | - | + |
| hsa-miR-320a | C6 | - | - | - | + |
| hsa-miR-320a | C6orf118 | + | + | - | + |
| hsa-miR-320a | C6orf123 | - | - | - | + |
| hsa-miR-320a | C6orf132 | - | - | - | + |
| hsa-miR-320a | C6orf201 | + | - | - | - |
| hsa-miR-320a | C6orf211 | - | - | - | + |
| hsa-miR-320a | C6orf222 | - | - | - | + |
| hsa-miR-320a | C6orf228 | - | - | - | + |
| hsa-miR-320a | C6orf26 | - | - | - | + |
| hsa-miR-320a | C6orf47 | - | - | - | + |
| hsa-miR-320a | C7 | - | - | - | + |
| hsa-miR-320a | C7orf53 | - | - | - | + |
| hsa-miR-320a | C7orf60 | - | - | - | + |
| hsa-miR-320a | C7orf76 | - | + | - | + |
| hsa-miR-320a | C8orf37 | - | - | - | + |
| hsa-miR-320a | C8orf44 | - | - | - | + |
| hsa-miR-320a | C8orf79 | - | - | - | + |
| hsa-miR-320a | C9orf123 | - | - | - | + |
| hsa-miR-320a | C9orf152 | - | - | - | + |
| hsa-miR-320a | C9orf40 | - | - | - | + |
| hsa-miR-320a | C9orf41 | + | - | - | + |
| hsa-miR-320a | C9orf47 | - | - | - | + |
| hsa-miR-320a | C9orf66 | - | - | - | + |
| hsa-miR-320a | C9orf72 | - | - | - | + |
| hsa-miR-320a | C9orf91 | - | - | - | + |
| hsa-miR-320a | CA13 | - | - | - | + |
| hsa-miR-320a | CA5B | - | - | - | + |
| hsa-miR-320a | CA8 | - | - | - | + |
| hsa-miR-320a | CAB39 | - | + | - | + |
| hsa-miR-320a | CABIN1 | - | - | + | - |
| hsa-miR-320a | CABLES1 | - | - | - | + |
| hsa-miR-320a | CABLES2 | - | - | - | + |
| hsa-miR-320a | CACNA1B | - | - | - | + |
| hsa-miR-320a | CACNA1H | - | - | - | + |
| hsa-miR-320a | CACNA1I | - | - | - | + |
| hsa-miR-320a | CACNA2D1 | - | - | - | + |
| hsa-miR-320a | CACNB4 | - | - | - | + |
| hsa-miR-320a | CACNG7 | - | - | - | + |
| hsa-miR-320a | CADM2 | - | - | - | + |
| hsa-miR-320a | CALB1 | - | - | - | + |
| hsa-miR-320a | CALCOCO2 | - | - | - | + |
| hsa-miR-320a | CALCRL | - | - | - | + |
| hsa-miR-320a | CALD1 | - | - | - | + |
| hsa-miR-320a | CALM1 | - | - | - | + |
| hsa-miR-320a | CALM3 | - | - | + | + |
| hsa-miR-320a | CALN1 | - | + | - | + |
| hsa-miR-320a | CALR | - | - | + | - |
| hsa-miR-320a | CALU | - | - | + | - |
| hsa-miR-320a | CAMK2A | - | - | - | + |
| hsa-miR-320a | CAMK2D | - | - | - | + |
| hsa-miR-320a | CAMK2G | - | - | - | + |
| hsa-miR-320a | CAMK2N1 | - | - | - | + |
| hsa-miR-320a | CAMKK2 | - | - | - | + |
| hsa-miR-320a | CAMKV | - | - | - | + |
| hsa-miR-320a | CAMSAP1 | - | - | - | + |
| hsa-miR-320a | CAMSAP2 | - | + | - | + |
| hsa-miR-320a | CAMTA1 | - | - | - | + |
| hsa-miR-320a | CANT1 | - | - | + | + |
| hsa-miR-320a | CANX | - | + | + | + |
| hsa-miR-320a | CAP1 | - | - | - | + |
| hsa-miR-320a | CAP2 | - | - | - | + |
| hsa-miR-320a | CAPN1 | - | - | - | + |
| hsa-miR-320a | CAPN2 | - | - | - | + |
| hsa-miR-320a | CAPN7 | - | - | - | + |
| hsa-miR-320a | CAPNS1 | - | - | + | + |
| hsa-miR-320a | CAPRIN1 | - | + | - | - |
| hsa-miR-320a | CARD18 | - | - | - | + |
| hsa-miR-320a | CARKD | - | - | - | + |
| hsa-miR-320a | CASD1 | - | - | - | + |
| hsa-miR-320a | CASP10 | - | - | - | + |
| hsa-miR-320a | CASP2 | - | - | - | + |
| hsa-miR-320a | CASP3 | - | + | - | - |
| hsa-miR-320a | CASQ2 | - | - | - | + |
| hsa-miR-320a | CAST | - | - | - | + |
| hsa-miR-320a | CASZ1 | - | - | - | + |
| hsa-miR-320a | CAV2 | - | - | - | + |
| hsa-miR-320a | CBFB | - | - | - | + |
| hsa-miR-320a | CBL | - | - | - | + |
| hsa-miR-320a | CBLN1 | - | + | - | - |
| hsa-miR-320a | CBR1 | - | + | - | + |
| hsa-miR-320a | CBWD5 | - | + | - | - |
| hsa-miR-320a | CBX2 | - | - | - | + |
| hsa-miR-320a | CBX3 | + | - | - | - |
| hsa-miR-320a | CBX5 | - | - | + | + |
| hsa-miR-320a | CBX7 | - | - | - | + |
| hsa-miR-320a | CBY1 | - | - | - | + |
| hsa-miR-320a | CC2D1B | - | - | - | + |
| hsa-miR-320a | CCDC113 | - | - | - | + |
| hsa-miR-320a | CCDC117 | - | - | - | + |
| hsa-miR-320a | CCDC121 | - | - | - | + |
| hsa-miR-320a | CCDC126 | - | - | - | + |
| hsa-miR-320a | CCDC144NL | - | - | - | + |
| hsa-miR-320a | CCDC146 | - | - | - | + |
| hsa-miR-320a | CCDC149 | - | + | - | + |
| hsa-miR-320a | CCDC152 | + | - | - | + |
| hsa-miR-320a | CCDC17 | + | + | - | - |
| hsa-miR-320a | CCDC172 | - | - | - | + |
| hsa-miR-320a | CCDC186 | - | - | - | + |
| hsa-miR-320a | CCDC3 | - | - | - | + |
| hsa-miR-320a | CCDC47 | - | - | - | + |
| hsa-miR-320a | CCDC50 | - | - | - | + |
| hsa-miR-320a | CCDC6 | - | - | - | + |
| hsa-miR-320a | CCDC69 | - | - | - | + |
| hsa-miR-320a | CCDC71L | - | - | - | + |
| hsa-miR-320a | CCDC75 | - | - | - | + |
| hsa-miR-320a | CCDC77 | - | - | - | + |
| hsa-miR-320a | CCDC82 | - | - | - | + |
| hsa-miR-320a | CCDC85A | - | - | - | + |
| hsa-miR-320a | CCDC88A | - | - | - | + |
| hsa-miR-320a | CCER1 | - | - | - | + |
| hsa-miR-320a | CCL11 | - | - | - | + |
| hsa-miR-320a | CCL28 | - | - | - | + |
| hsa-miR-320a | CCNA2 | - | - | - | + |
| hsa-miR-320a | CCND2 | - | - | + | + |
| hsa-miR-320a | CCND3 | - | - | - | + |
| hsa-miR-320a | CCNE1 | - | - | - | + |
| hsa-miR-320a | CCNE2 | - | - | - | + |
| hsa-miR-320a | CCNG2 | - | - | - | + |
| hsa-miR-320a | CCNI | - | - | + | - |
| hsa-miR-320a | CCNJ | - | - | - | + |
| hsa-miR-320a | CCNT1 | - | - | - | + |
| hsa-miR-320a | CCNT2 | - | - | - | + |
| hsa-miR-320a | CCNY | - | - | - | + |
| hsa-miR-320a | CCNYL1 | - | - | - | + |
| hsa-miR-320a | CCP110 | - | - | - | + |
| hsa-miR-320a | CCPG1 | - | - | - | + |
| hsa-miR-320a | CCR3 | - | - | - | + |
| hsa-miR-320a | CCR7 | + | - | - | + |
| hsa-miR-320a | CCR9 | - | - | - | + |
| hsa-miR-320a | CCSER2 | + | - | - | - |
| hsa-miR-320a | CCT3 | - | - | + | - |
| hsa-miR-320a | CCT5 | - | - | - | + |
| hsa-miR-320a | CD109 | - | - | - | + |
| hsa-miR-320a | CD163 | - | - | - | + |
| hsa-miR-320a | CD1D | - | - | - | + |
| hsa-miR-320a | CD2 | - | - | - | + |
| hsa-miR-320a | CD244 | - | - | - | + |
| hsa-miR-320a | CD274 | - | + | - | + |
| hsa-miR-320a | CD2AP | - | - | - | + |
| hsa-miR-320a | CD302 | - | - | - | + |
| hsa-miR-320a | CD34 | - | - | - | + |
| hsa-miR-320a | CD3G | + | - | - | - |
| hsa-miR-320a | CD44 | - | - | + | - |
| hsa-miR-320a | CD46 | - | - | - | + |
| hsa-miR-320a | CD47 | - | - | - | + |
| hsa-miR-320a | CD5 | - | - | - | + |
| hsa-miR-320a | CD84 | - | - | - | + |
| hsa-miR-320a | CD93 | - | - | - | + |
| hsa-miR-320a | CD97 | - | - | - | + |
| hsa-miR-320a | CD99L2 | - | - | - | + |
| hsa-miR-320a | CDADC1 | - | - | - | + |
| hsa-miR-320a | CDC14B | - | - | - | + |
| hsa-miR-320a | CDC23 | - | - | - | + |
| hsa-miR-320a | CDC25A | - | - | - | + |
| hsa-miR-320a | CDC37L1 | - | - | - | + |
| hsa-miR-320a | CDC42BPA | - | - | - | + |
| hsa-miR-320a | CDC42EP3 | - | - | - | + |
| hsa-miR-320a | CDC42SE1 | - | - | + | - |
| hsa-miR-320a | CDCA3 | + | - | + | - |
| hsa-miR-320a | CDCA7 | - | - | - | + |
| hsa-miR-320a | CDCA7L | - | - | - | + |
| hsa-miR-320a | CDH19 | - | - | - | + |
| hsa-miR-320a | CDH2 | - | + | - | + |
| hsa-miR-320a | CDH20 | + | + | - | + |
| hsa-miR-320a | CDH24 | - | - | - | + |
| hsa-miR-320a | CDH6 | - | - | - | + |
| hsa-miR-320a | CDH9 | - | - | - | + |
| hsa-miR-320a | CDK1 | - | - | - | + |
| hsa-miR-320a | CDK12 | - | - | - | + |
| hsa-miR-320a | CDK13 | + | + | - | + |
| hsa-miR-320a | CDK14 | - | - | - | + |
| hsa-miR-320a | CDK17 | - | - | - | + |
| hsa-miR-320a | CDK19 | - | - | - | + |
| hsa-miR-320a | CDK2AP2 | - | - | + | - |
| hsa-miR-320a | CDK4 | - | - | + | - |
| hsa-miR-320a | CDK5R1 | - | - | - | + |
| hsa-miR-320a | CDK5R2 | - | - | - | + |
| hsa-miR-320a | CDK5RAP1 | + | - | - | - |
| hsa-miR-320a | CDK6 | - | + | - | + |
| hsa-miR-320a | CDKN1B | - | - | - | + |
| hsa-miR-320a | CDKN1C | - | - | - | + |
| hsa-miR-320a | CDKN2A | - | - | + | - |
| hsa-miR-320a | CDKN2AIPNL | - | - | - | + |
| hsa-miR-320a | CDKN2B | - | - | - | + |
| hsa-miR-320a | CDKN2C | - | - | - | + |
| hsa-miR-320a | CDO1 | - | - | - | + |
| hsa-miR-320a | CDON | - | - | - | + |
| hsa-miR-320a | CDR2L | - | - | - | + |
| hsa-miR-320a | CDRT15L2 | - | - | - | + |
| hsa-miR-320a | CDS2 | - | - | + | - |
| hsa-miR-320a | CDT1 | - | - | - | + |
| hsa-miR-320a | CDV3 | - | - | - | + |
| hsa-miR-320a | CDYL | - | - | - | + |
| hsa-miR-320a | CEACAM6 | + | - | - | + |
| hsa-miR-320a | CEBPG | - | - | - | + |
| hsa-miR-320a | CECR5 | - | - | - | + |
| hsa-miR-320a | CECR6 | - | + | - | + |
| hsa-miR-320a | CELA1 | - | - | - | + |
| hsa-miR-320a | CELF1 | - | - | - | + |
| hsa-miR-320a | CELSR2 | - | - | - | + |
| hsa-miR-320a | CELSR3 | - | + | - | - |
| hsa-miR-320a | CENPA | - | - | - | + |
| hsa-miR-320a | CENPF | - | - | - | + |
| hsa-miR-320a | CENPJ | - | - | - | + |
| hsa-miR-320a | CEP126 | - | - | - | + |
| hsa-miR-320a | CEP350 | - | - | - | + |
| hsa-miR-320a | CEP41 | - | + | - | - |
| hsa-miR-320a | CEP85 | + | + | - | + |
| hsa-miR-320a | CEP97 | - | - | - | + |
| hsa-miR-320a | CEPT1 | - | - | - | + |
| hsa-miR-320a | CERS2 | - | - | - | + |
| hsa-miR-320a | CERS3 | - | - | - | + |
| hsa-miR-320a | CERS6 | - | - | - | + |
| hsa-miR-320a | CFHR3 | + | - | - | + |
| hsa-miR-320a | CFL2 | - | - | - | + |
| hsa-miR-320a | CGA | + | - | - | + |
| hsa-miR-320a | CGGBP1 | - | + | - | + |
| hsa-miR-320a | CGN | - | - | - | + |
| hsa-miR-320a | CGNL1 | - | - | - | + |
| hsa-miR-320a | CHAMP1 | - | - | - | + |
| hsa-miR-320a | CHCHD7 | - | - | - | + |
| hsa-miR-320a | CHD1L | - | - | + | - |
| hsa-miR-320a | CHD5 | - | - | - | + |
| hsa-miR-320a | CHD6 | - | - | - | + |
| hsa-miR-320a | CHD9 | - | - | - | + |
| hsa-miR-320a | CHEK1 | - | + | - | + |
| hsa-miR-320a | CHIT1 | - | - | - | + |
| hsa-miR-320a | CHL1 | - | - | - | + |
| hsa-miR-320a | CHM | - | - | - | + |
| hsa-miR-320a | CHML | - | - | - | + |
| hsa-miR-320a | CHMP2B | - | - | - | + |
| hsa-miR-320a | CHMP4B | - | - | - | + |
| hsa-miR-320a | CHMP4C | - | - | - | + |
| hsa-miR-320a | CHN1 | - | - | - | + |
| hsa-miR-320a | CHP1 | - | - | - | + |
| hsa-miR-320a | CHP2 | - | - | - | + |
| hsa-miR-320a | CHRAC1 | - | - | - | + |
| hsa-miR-320a | CHRFAM7A | - | - | - | + |
| hsa-miR-320a | CHRNA3 | - | - | - | + |
| hsa-miR-320a | CHRNA7 | - | - | - | + |
| hsa-miR-320a | CHRNB2 | - | - | - | + |
| hsa-miR-320a | CHST11 | - | - | - | + |
| hsa-miR-320a | CHST7 | - | - | - | + |
| hsa-miR-320a | CHST8 | - | - | - | + |
| hsa-miR-320a | CHST9 | - | - | - | + |
| hsa-miR-320a | CHSY1 | - | - | - | + |
| hsa-miR-320a | CHSY3 | - | - | - | + |
| hsa-miR-320a | CHTF8 | - | - | - | + |
| hsa-miR-320a | CHUK | - | - | - | + |
| hsa-miR-320a | CIAPIN1 | - | - | - | + |
| hsa-miR-320a | CIB4 | - | - | - | + |
| hsa-miR-320a | CIC | - | - | - | + |
| hsa-miR-320a | CIPC | - | - | - | + |
| hsa-miR-320a | CISD1 | - | - | - | + |
| hsa-miR-320a | CISD2 | + | - | - | - |
| hsa-miR-320a | CKAP2 | - | - | - | + |
| hsa-miR-320a | CKAP4 | - | - | - | + |
| hsa-miR-320a | CKAP5 | - | + | - | + |
| hsa-miR-320a | CKB | - | - | + | - |
| hsa-miR-320a | CKS1B | - | - | + | - |
| hsa-miR-320a | CLASP1 | - | + | - | + |
| hsa-miR-320a | CLCA2 | - | - | - | + |
| hsa-miR-320a | CLCC1 | - | - | - | + |
| hsa-miR-320a | CLCN3 | - | - | - | + |
| hsa-miR-320a | CLCN4 | - | - | - | + |
| hsa-miR-320a | CLCN5 | - | - | - | + |
| hsa-miR-320a | CLDN1 | - | - | - | + |
| hsa-miR-320a | CLDN12 | - | - | + | - |
| hsa-miR-320a | CLDN22 | - | - | - | + |
| hsa-miR-320a | CLDN7 | - | - | - | + |
| hsa-miR-320a | CLDN8 | - | - | - | + |
| hsa-miR-320a | CLEC2A | - | - | - | + |
| hsa-miR-320a | CLEC3A | - | - | - | + |
| hsa-miR-320a | CLHC1 | - | + | - | - |
| hsa-miR-320a | CLIC4 | - | - | - | + |
| hsa-miR-320a | CLIC6 | - | - | - | + |
| hsa-miR-320a | CLINT1 | - | - | - | + |
| hsa-miR-320a | CLK2 | - | - | - | + |
| hsa-miR-320a | CLLU1OS | - | - | - | + |
| hsa-miR-320a | CLMN | - | - | - | + |
| hsa-miR-320a | CLN6 | - | - | + | - |
| hsa-miR-320a | CLOCK | - | + | - | + |
| hsa-miR-320a | CLSPN | - | + | - | - |
| hsa-miR-320a | CLTC | - | - | - | + |
| hsa-miR-320a | CLU | - | - | - | + |
| hsa-miR-320a | CLUH | - | - | + | - |
| hsa-miR-320a | CLUL1 | - | - | - | + |
| hsa-miR-320a | CLVS1 | - | - | - | + |
| hsa-miR-320a | CMAS | - | - | + | - |
| hsa-miR-320a | CMPK1 | - | + | - | + |
| hsa-miR-320a | CMTM4 | - | + | - | + |
| hsa-miR-320a | CMTR2 | - | + | - | - |
| hsa-miR-320a | CNGB3 | - | - | - | + |
| hsa-miR-320a | CNKSR2 | - | + | - | + |
| hsa-miR-320a | CNNM1 | - | - | - | + |
| hsa-miR-320a | CNOT6 | - | - | - | + |
| hsa-miR-320a | CNOT7 | - | + | - | + |
| hsa-miR-320a | CNPY4 | - | - | - | + |
| hsa-miR-320a | CNTN1 | - | - | - | + |
| hsa-miR-320a | CNTN2 | - | - | - | + |
| hsa-miR-320a | CNTN3 | - | - | - | + |
| hsa-miR-320a | CNTN5 | - | - | - | + |
| hsa-miR-320a | CNTNAP2 | - | - | - | + |
| hsa-miR-320a | CNTNAP5 | - | + | - | - |
| hsa-miR-320a | COA5 | - | - | - | + |
| hsa-miR-320a | COBLL1 | - | - | - | + |
| hsa-miR-320a | COG5 | - | - | + | + |
| hsa-miR-320a | COG6 | - | - | - | + |
| hsa-miR-320a | COL10A1 | - | - | - | + |
| hsa-miR-320a | COL12A1 | - | - | - | + |
| hsa-miR-320a | COL1A2 | - | - | - | + |
| hsa-miR-320a | COL24A1 | - | + | - | - |
| hsa-miR-320a | COL3A1 | - | - | - | + |
| hsa-miR-320a | COL4A3BP | - | - | - | + |
| hsa-miR-320a | COL4A4 | - | - | - | + |
| hsa-miR-320a | COL4A6 | - | - | - | + |
| hsa-miR-320a | COMMD2 | - | - | - | + |
| hsa-miR-320a | COMMD3-BMI1 | - | + | - | - |
| hsa-miR-320a | COMMD4 | - | - | + | - |
| hsa-miR-320a | COPA | - | - | - | + |
| hsa-miR-320a | COPG2 | - | + | - | + |
| hsa-miR-320a | COPS2 | + | + | - | - |
| hsa-miR-320a | COQ10A | - | - | - | + |
| hsa-miR-320a | COQ10B | - | - | - | + |
| hsa-miR-320a | COQ5 | - | - | - | + |
| hsa-miR-320a | CORO1C | - | - | - | + |
| hsa-miR-320a | CORO2A | - | - | - | + |
| hsa-miR-320a | CORO2B | - | - | - | + |
| hsa-miR-320a | COTL1 | - | - | + | - |
| hsa-miR-320a | COX1 | - | - | + | - |
| hsa-miR-320a | COX10 | - | - | - | + |
| hsa-miR-320a | COX11 | - | - | - | + |
| hsa-miR-320a | COX2 | - | - | + | - |
| hsa-miR-320a | COX3 | - | - | + | - |
| hsa-miR-320a | COX6B1 | - | - | + | - |
| hsa-miR-320a | CPA3 | - | - | - | + |
| hsa-miR-320a | CPA5 | - | - | - | + |
| hsa-miR-320a | CPD | - | + | + | + |
| hsa-miR-320a | CPEB1 | - | + | - | - |
| hsa-miR-320a | CPEB3 | - | - | + | - |
| hsa-miR-320a | CPEB4 | - | - | - | + |
| hsa-miR-320a | CPED1 | + | + | - | + |
| hsa-miR-320a | CPM | - | - | - | + |
| hsa-miR-320a | CPNE8 | - | - | - | + |
| hsa-miR-320a | CPS1 | - | - | - | + |
| hsa-miR-320a | CPSF3L | - | - | + | - |
| hsa-miR-320a | CPSF6 | - | - | - | + |
| hsa-miR-320a | CPSF7 | - | - | - | + |
| hsa-miR-320a | CPVL | - | - | - | + |
| hsa-miR-320a | CPXCR1 | - | - | - | + |
| hsa-miR-320a | CRBN | - | - | - | + |
| hsa-miR-320a | CREB1 | - | - | - | + |
| hsa-miR-320a | CREB3L2 | - | - | - | + |
| hsa-miR-320a | CREB5 | - | + | - | + |
| hsa-miR-320a | CREBRF | - | - | + | - |
| hsa-miR-320a | CREG1 | + | + | - | + |
| hsa-miR-320a | CRELD1 | - | - | - | + |
| hsa-miR-320a | CREM | - | - | - | + |
| hsa-miR-320a | CRIM1 | - | - | - | + |
| hsa-miR-320a | CRIPAK | - | - | - | + |
| hsa-miR-320a | CRIPT | - | + | - | - |
| hsa-miR-320a | CRISP2 | + | - | - | + |
| hsa-miR-320a | CRISPLD1 | - | - | - | + |
| hsa-miR-320a | CRISPLD2 | - | - | - | + |
| hsa-miR-320a | CRK | - | - | + | - |
| hsa-miR-320a | CRKL | - | + | - | + |
| hsa-miR-320a | CRLS1 | - | - | - | + |
| hsa-miR-320a | CROT | - | - | - | + |
| hsa-miR-320a | CRTC3 | - | - | - | + |
| hsa-miR-320a | CRYBG3 | - | - | - | + |
| hsa-miR-320a | CSDA | - | - | - | + |
| hsa-miR-320a | CSDE1 | - | - | + | + |
| hsa-miR-320a | CSE1L | - | - | + | + |
| hsa-miR-320a | CSF1 | - | - | - | + |
| hsa-miR-320a | CSF2RA | - | - | - | + |
| hsa-miR-320a | CSF2RB | - | - | - | + |
| hsa-miR-320a | CSMD3 | - | - | - | + |
| hsa-miR-320a | CSNK1A1L | - | - | - | + |
| hsa-miR-320a | CSNK1E | - | - | - | + |
| hsa-miR-320a | CSNK1G1 | - | - | - | + |
| hsa-miR-320a | CSNK1G2 | - | - | - | + |
| hsa-miR-320a | CSNK1G3 | - | - | - | + |
| hsa-miR-320a | CSRNP2 | - | - | - | + |
| hsa-miR-320a | CSRNP3 | - | - | - | + |
| hsa-miR-320a | CST9L | - | - | - | + |
| hsa-miR-320a | CSTF2T | - | - | - | + |
| hsa-miR-320a | CTAG2 | - | - | - | + |
| hsa-miR-320a | CTAGE1 | - | - | - | + |
| hsa-miR-320a | CTAGE4 | - | - | - | + |
| hsa-miR-320a | CTAGE5 | - | - | - | + |
| hsa-miR-320a | CTAGE8 | - | - | - | + |
| hsa-miR-320a | CTAGE9 | - | - | - | + |
| hsa-miR-320a | CTC1 | - | - | - | + |
| hsa-miR-320a | CTCF | - | - | - | + |
| hsa-miR-320a | CTD-2117L12.1 | - | - | - | + |
| hsa-miR-320a | CTD-2228K2.5 | - | - | - | + |
| hsa-miR-320a | CTDNEP1 | + | - | - | + |
| hsa-miR-320a | CTDSP2 | - | - | - | + |
| hsa-miR-320a | CTDSPL | - | - | - | + |
| hsa-miR-320a | CTLA4 | - | - | - | + |
| hsa-miR-320a | CTNNAL1 | - | - | + | + |
| hsa-miR-320a | CTNNB1 | - | - | - | + |
| hsa-miR-320a | CTNNBL1 | - | - | + | - |
| hsa-miR-320a | CTNS | - | - | + | - |
| hsa-miR-320a | CTPS | - | - | - | + |
| hsa-miR-320a | CTPS1 | - | - | + | - |
| hsa-miR-320a | CTPS2 | - | - | - | + |
| hsa-miR-320a | CTRC | - | - | + | - |
| hsa-miR-320a | CTSC | - | - | - | + |
| hsa-miR-320a | CTSV | + | + | - | - |
| hsa-miR-320a | CTTN | - | - | - | + |
| hsa-miR-320a | CTTNBP2NL | - | - | - | + |
| hsa-miR-320a | CTXN2 | + | - | - | + |
| hsa-miR-320a | CUL3 | - | - | - | + |
| hsa-miR-320a | CUL5 | - | - | - | + |
| hsa-miR-320a | CUL7 | - | - | - | + |
| hsa-miR-320a | CUTC | + | - | - | + |
| hsa-miR-320a | CWF19L1 | - | - | - | + |
| hsa-miR-320a | CX3CR1 | - | - | - | + |
| hsa-miR-320a | CXCL14 | - | - | - | + |
| hsa-miR-320a | CXCL16 | - | - | - | + |
| hsa-miR-320a | CXCL2 | + | - | - | - |
| hsa-miR-320a | CXCL3 | - | - | - | + |
| hsa-miR-320a | CXCL5 | - | - | - | + |
| hsa-miR-320a | CXCL9 | - | - | - | + |
| hsa-miR-320a | CXCR2 | - | - | - | + |
| hsa-miR-320a | CXXC4 | - | - | - | + |
| hsa-miR-320a | CXorf23 | - | - | - | + |
| hsa-miR-320a | CXorf31 | - | - | - | + |
| hsa-miR-320a | CXorf38 | + | - | - | - |
| hsa-miR-320a | CXorf57 | - | - | - | + |
| hsa-miR-320a | CXorf64 | - | - | - | + |
| hsa-miR-320a | CYB561D1 | - | - | - | + |
| hsa-miR-320a | CYB5A | - | - | - | + |
| hsa-miR-320a | CYB5D2 | - | - | - | + |
| hsa-miR-320a | CYBA | - | - | + | - |
| hsa-miR-320a | CYBB | - | - | - | + |
| hsa-miR-320a | CYBRD1 | - | - | - | + |
| hsa-miR-320a | CYGB | - | - | - | + |
| hsa-miR-320a | CYLD | - | - | + | - |
| hsa-miR-320a | CYP1A2 | - | + | - | + |
| hsa-miR-320a | CYP27B1 | - | - | - | + |
| hsa-miR-320a | CYP2J2 | - | - | - | + |
| hsa-miR-320a | CYP2U1 | - | - | - | + |
| hsa-miR-320a | CYP3A7 | - | - | - | + |
| hsa-miR-320a | CYP4A11 | - | - | - | + |
| hsa-miR-320a | CYP4F22 | - | - | - | + |
| hsa-miR-320a | CYP4Z1 | - | - | - | + |
| hsa-miR-320a | CYP7A1 | - | - | - | + |
| hsa-miR-320a | CYP8B1 | - | - | - | + |
| hsa-miR-320a | CYR61 | - | - | - | + |
| hsa-miR-320a | CYSLTR1 | - | - | - | + |
| hsa-miR-320a | CYTH1 | - | + | - | + |
| hsa-miR-320a | CYTH3 | - | - | + | + |
| hsa-miR-320a | CYTIP | - | + | - | + |
| hsa-miR-320a | CYYR1 | - | - | - | + |
| hsa-miR-320a | DAAM1 | - | - | - | + |
| hsa-miR-320a | DACT1 | - | - | - | + |
| hsa-miR-320a | DAG1 | - | + | - | + |
| hsa-miR-320a | DAK | - | - | - | + |
| hsa-miR-320a | DAP | - | - | - | + |
| hsa-miR-320a | DAPL1 | - | - | - | + |
| hsa-miR-320a | DARS2 | - | - | - | + |
| hsa-miR-320a | DAZ1 | - | - | - | + |
| hsa-miR-320a | DAZ3 | - | - | - | + |
| hsa-miR-320a | DAZAP1 | + | + | - | - |
| hsa-miR-320a | DAZL | - | - | - | + |
| hsa-miR-320a | DBN1 | - | + | - | + |
| hsa-miR-320a | DBNDD1 | - | - | - | + |
| hsa-miR-320a | DBR1 | - | + | - | + |
| hsa-miR-320a | DCAF10 | - | + | - | + |
| hsa-miR-320a | DCAF11 | + | - | + | - |
| hsa-miR-320a | DCAF12 | - | - | - | + |
| hsa-miR-320a | DCAF12L1 | - | - | - | + |
| hsa-miR-320a | DCAF16 | + | - | - | + |
| hsa-miR-320a | DCAF17 | - | - | - | + |
| hsa-miR-320a | DCAF4L1 | - | - | - | + |
| hsa-miR-320a | DCAF4L2 | - | - | - | + |
| hsa-miR-320a | DCAF5 | - | - | - | + |
| hsa-miR-320a | DCAF8L1 | - | - | - | + |
| hsa-miR-320a | DCBLD2 | - | - | - | + |
| hsa-miR-320a | DCDC2 | - | - | - | + |
| hsa-miR-320a | DCHS1 | - | - | - | + |
| hsa-miR-320a | DCK | + | + | - | - |
| hsa-miR-320a | DCLK3 | - | - | - | + |
| hsa-miR-320a | DCLRE1B | - | - | + | - |
| hsa-miR-320a | DCP1A | - | + | - | - |
| hsa-miR-320a | DCP2 | - | - | + | + |
| hsa-miR-320a | DCTN4 | - | - | - | + |
| hsa-miR-320a | DCTN5 | - | + | + | - |
| hsa-miR-320a | DCX | - | - | - | + |
| hsa-miR-320a | DDA1 | - | - | - | + |
| hsa-miR-320a | DDAH1 | - | - | - | + |
| hsa-miR-320a | DDHD2 | - | - | - | + |
| hsa-miR-320a | DDIT4L | - | - | - | + |
| hsa-miR-320a | DDX20 | - | - | - | + |
| hsa-miR-320a | DDX21 | - | - | - | + |
| hsa-miR-320a | DDX26B | - | - | - | + |
| hsa-miR-320a | DDX3X | - | - | - | + |
| hsa-miR-320a | DDX3Y | - | - | - | + |
| hsa-miR-320a | DDX42 | + | - | - | - |
| hsa-miR-320a | DDX54 | - | + | - | + |
| hsa-miR-320a | DDX60 | - | - | - | + |
| hsa-miR-320a | DEFB118 | + | - | - | + |
| hsa-miR-320a | DEK | - | - | - | + |
| hsa-miR-320a | DENND1B | - | - | - | + |
| hsa-miR-320a | DENND4A | - | - | - | + |
| hsa-miR-320a | DENND4B | - | - | + | - |
| hsa-miR-320a | DENND5A | - | - | - | + |
| hsa-miR-320a | DENND6A | - | - | - | + |
| hsa-miR-320a | DEPDC1 | - | - | - | + |
| hsa-miR-320a | DEPTOR | - | - | - | + |
| hsa-miR-320a | DERL1 | - | + | - | + |
| hsa-miR-320a | DERL2 | - | - | - | + |
| hsa-miR-320a | DESI1 | - | - | - | + |
| hsa-miR-320a | DESI2 | - | + | - | + |
| hsa-miR-320a | DGCR8 | - | - | - | + |
| hsa-miR-320a | DGKB | - | + | - | + |
| hsa-miR-320a | DGKE | - | + | - | + |
| hsa-miR-320a | DHDDS | - | + | - | - |
| hsa-miR-320a | DHRSX | - | - | + | - |
| hsa-miR-320a | DHX15 | - | + | - | + |
| hsa-miR-320a | DHX33 | - | - | - | + |
| hsa-miR-320a | DHX40 | - | - | - | + |
| hsa-miR-320a | DHX57 | - | - | - | + |
| hsa-miR-320a | DHX8 | - | - | - | + |
| hsa-miR-320a | DIAPH1 | - | - | + | + |
| hsa-miR-320a | DIAPH3 | - | - | - | + |
| hsa-miR-320a | DICER1 | - | - | - | + |
| hsa-miR-320a | DIDO1 | - | - | - | + |
| hsa-miR-320a | DIO2 | - | + | - | + |
| hsa-miR-320a | DIP2B | - | - | - | + |
| hsa-miR-320a | DIP2C | - | + | - | + |
| hsa-miR-320a | DIRC2 | - | - | + | + |
| hsa-miR-320a | DISC1 | - | + | - | + |
| hsa-miR-320a | DKK1 | - | - | - | + |
| hsa-miR-320a | DKK2 | - | - | - | + |
| hsa-miR-320a | DLC1 | - | - | - | + |
| hsa-miR-320a | DLG1 | + | - | - | + |
| hsa-miR-320a | DLG2 | - | - | - | + |
| hsa-miR-320a | DLG4 | - | - | - | + |
| hsa-miR-320a | DLGAP2 | - | - | - | + |
| hsa-miR-320a | DLGAP4 | - | + | - | + |
| hsa-miR-320a | DLST | - | - | - | + |
| hsa-miR-320a | DLX1 | - | + | - | + |
| hsa-miR-320a | DLX5 | - | - | - | + |
| hsa-miR-320a | DMD | - | - | - | + |
| hsa-miR-320a | DMP1 | - | - | - | + |
| hsa-miR-320a | DMRT3 | - | - | - | + |
| hsa-miR-320a | DMRTC2 | - | - | - | + |
| hsa-miR-320a | DMXL1 | - | - | - | + |
| hsa-miR-320a | DNAH5 | - | - | - | + |
| hsa-miR-320a | DNAH6 | - | - | - | + |
| hsa-miR-320a | DNAH7 | + | - | - | - |
| hsa-miR-320a | DNAI1 | + | - | - | - |
| hsa-miR-320a | DNAJA2 | - | - | - | + |
| hsa-miR-320a | DNAJA4 | - | - | - | + |
| hsa-miR-320a | DNAJB14 | - | + | - | + |
| hsa-miR-320a | DNAJB5 | - | - | + | - |
| hsa-miR-320a | DNAJB7 | - | - | - | + |
| hsa-miR-320a | DNAJB9 | - | - | + | + |
| hsa-miR-320a | DNAJC11 | - | - | - | + |
| hsa-miR-320a | DNAJC12 | - | - | - | + |
| hsa-miR-320a | DNAJC13 | - | - | - | + |
| hsa-miR-320a | DNAJC14 | - | - | + | - |
| hsa-miR-320a | DNAJC18 | - | - | - | + |
| hsa-miR-320a | DNAJC21 | - | - | - | + |
| hsa-miR-320a | DNAJC27 | - | - | - | + |
| hsa-miR-320a | DNAJC3 | - | + | - | - |
| hsa-miR-320a | DNAJC30 | + | - | - | - |
| hsa-miR-320a | DNAJC6 | - | - | - | + |
| hsa-miR-320a | DNAJC8 | - | - | - | + |
| hsa-miR-320a | DNALI1 | - | - | - | + |
| hsa-miR-320a | DNER | + | + | - | + |
| hsa-miR-320a | DOCK3 | - | - | - | + |
| hsa-miR-320a | DOK3 | - | - | + | + |
| hsa-miR-320a | DOK6 | - | - | - | + |
| hsa-miR-320a | DOT1L | - | - | - | + |
| hsa-miR-320a | DPF2 | - | - | - | + |
| hsa-miR-320a | DPM2 | - | - | - | + |
| hsa-miR-320a | DPP10 | - | - | - | + |
| hsa-miR-320a | DPP3 | - | - | + | - |
| hsa-miR-320a | DPP8 | - | - | - | + |
| hsa-miR-320a | DPPA4 | - | - | - | + |
| hsa-miR-320a | DPT | - | - | - | + |
| hsa-miR-320a | DPY19L1 | - | - | - | + |
| hsa-miR-320a | DPY19L4 | - | - | - | + |
| hsa-miR-320a | DPY30 | + | + | - | - |
| hsa-miR-320a | DPYSL2 | - | - | + | + |
| hsa-miR-320a | DPYSL5 | - | - | - | + |
| hsa-miR-320a | DR1 | - | - | + | - |
| hsa-miR-320a | DRD5 | - | - | - | + |
| hsa-miR-320a | DROSHA | - | - | - | + |
| hsa-miR-320a | DSC2 | - | - | - | + |
| hsa-miR-320a | DSC3 | - | - | - | + |
| hsa-miR-320a | DSCC1 | - | + | - | + |
| hsa-miR-320a | DSCR4 | - | - | - | + |
| hsa-miR-320a | DSCR6 | - | - | - | + |
| hsa-miR-320a | DST | - | - | - | + |
| hsa-miR-320a | DTHD1 | - | - | - | + |
| hsa-miR-320a | DTL | - | - | - | + |
| hsa-miR-320a | DTNA | - | + | - | + |
| hsa-miR-320a | DTX3 | - | - | + | + |
| hsa-miR-320a | DTX3L | - | - | - | + |
| hsa-miR-320a | DUSP16 | - | + | - | + |
| hsa-miR-320a | DUSP18 | - | - | - | + |
| hsa-miR-320a | DUSP19 | - | - | - | + |
| hsa-miR-320a | DUSP22 | - | - | - | + |
| hsa-miR-320a | DUSP3 | - | - | - | + |
| hsa-miR-320a | DUSP4 | - | - | - | + |
| hsa-miR-320a | DUSP8 | - | - | + | - |
| hsa-miR-320a | DVL2 | - | - | - | + |
| hsa-miR-320a | DVL3 | - | - | + | - |
| hsa-miR-320a | DYNC1I1 | - | + | - | + |
| hsa-miR-320a | DYNLT3 | - | - | - | + |
| hsa-miR-320a | DYRK1A | - | - | - | + |
| hsa-miR-320a | DYRK1B | - | - | + | + |
| hsa-miR-320a | Drosophila | - | + | - | - |
| hsa-miR-320a | E2F1 | - | - | - | + |
| hsa-miR-320a | E2F2 | - | - | - | + |
| hsa-miR-320a | E2F3 | - | - | - | + |
| hsa-miR-320a | EAF1 | - | - | + | - |
| hsa-miR-320a | EARS2 | - | - | + | + |
| hsa-miR-320a | EBAG9 | - | - | - | + |
| hsa-miR-320a | EBF2 | - | + | - | + |
| hsa-miR-320a | ECE1 | - | - | - | + |
| hsa-miR-320a | ECH1 | - | - | + | - |
| hsa-miR-320a | ECHDC1 | - | - | - | + |
| hsa-miR-320a | ECT2L | - | - | - | + |
| hsa-miR-320a | EDA2R | - | - | - | + |
| hsa-miR-320a | EDC3 | - | - | + | - |
| hsa-miR-320a | EDC4 | - | - | - | + |
| hsa-miR-320a | EDEM1 | - | - | - | + |
| hsa-miR-320a | EDEM3 | - | - | - | + |
| hsa-miR-320a | EDIL3 | - | - | - | + |
| hsa-miR-320a | EDN2 | - | - | - | + |
| hsa-miR-320a | EDNRB | - | - | - | + |
| hsa-miR-320a | EEA1 | - | - | - | + |
| hsa-miR-320a | EEF2 | - | - | + | - |
| hsa-miR-320a | EEF2K | - | - | - | + |
| hsa-miR-320a | EEPD1 | - | - | - | + |
| hsa-miR-320a | EFCAB14 | - | - | - | + |
| hsa-miR-320a | EFCAB3 | - | - | - | + |
| hsa-miR-320a | EFHC1 | - | - | - | + |
| hsa-miR-320a | EFHD2 | - | - | - | + |
| hsa-miR-320a | EFNA5 | - | - | - | + |
| hsa-miR-320a | EFNB1 | - | - | - | + |
| hsa-miR-320a | EFNB2 | - | - | - | + |
| hsa-miR-320a | EFR3A | - | - | - | + |
| hsa-miR-320a | EFS | + | - | - | + |
| hsa-miR-320a | EGF | - | - | - | + |
| hsa-miR-320a | EGLN1 | - | - | - | + |
| hsa-miR-320a | EGR3 | - | - | - | + |
| hsa-miR-320a | EIF1AX | - | - | - | + |
| hsa-miR-320a | EIF2A | - | - | + | - |
| hsa-miR-320a | EIF2AK3 | + | - | - | + |
| hsa-miR-320a | EIF2B1 | + | - | - | - |
| hsa-miR-320a | EIF2B3 | - | - | + | - |
| hsa-miR-320a | EIF2B5 | - | - | + | - |
| hsa-miR-320a | EIF2D | + | + | - | + |
| hsa-miR-320a | EIF2S2 | - | - | - | + |
| hsa-miR-320a | EIF3C | - | - | + | - |
| hsa-miR-320a | EIF3F | - | - | + | - |
| hsa-miR-320a | EIF3J | - | - | + | + |
| hsa-miR-320a | EIF3L | - | - | + | - |
| hsa-miR-320a | EIF4B | - | - | - | + |
| hsa-miR-320a | EIF4E | - | + | - | - |
| hsa-miR-320a | EIF4EBP2 | - | - | - | + |
| hsa-miR-320a | EIF4G1 | - | - | + | - |
| hsa-miR-320a | EIF4H | - | - | - | + |
| hsa-miR-320a | EIF5 | - | - | - | + |
| hsa-miR-320a | EIF5A2 | - | - | - | + |
| hsa-miR-320a | EIF5B | - | - | - | + |
| hsa-miR-320a | ELAC1 | - | - | - | + |
| hsa-miR-320a | ELAVL1 | - | - | - | + |
| hsa-miR-320a | ELAVL2 | - | - | - | + |
| hsa-miR-320a | ELAVL4 | - | - | - | + |
| hsa-miR-320a | ELF3 | - | - | - | + |
| hsa-miR-320a | ELF4 | - | - | - | + |
| hsa-miR-320a | ELK1 | - | - | - | + |
| hsa-miR-320a | ELK3 | - | - | - | + |
| hsa-miR-320a | ELK4 | - | - | - | + |
| hsa-miR-320a | ELL2 | - | + | - | + |
| hsa-miR-320a | ELL3 | - | - | - | + |
| hsa-miR-320a | ELMOD2 | - | - | - | + |
| hsa-miR-320a | ELMSAN1 | - | - | - | + |
| hsa-miR-320a | ELOVL2 | - | + | - | - |
| hsa-miR-320a | ELOVL7 | - | - | - | + |
| hsa-miR-320a | EMB | - | - | - | + |
| hsa-miR-320a | EMC1 | - | - | - | + |
| hsa-miR-320a | EMC7 | + | - | - | - |
| hsa-miR-320a | EMILIN3 | - | - | - | + |
| hsa-miR-320a | EML6 | - | - | - | + |
| hsa-miR-320a | EMP2 | - | - | - | + |
| hsa-miR-320a | ENAH | - | + | - | + |
| hsa-miR-320a | ENAM | - | - | - | + |
| hsa-miR-320a | ENDOD1 | - | - | - | + |
| hsa-miR-320a | ENOX2 | - | - | - | + |
| hsa-miR-320a | ENPEP | - | - | + | - |
| hsa-miR-320a | ENPP1 | - | - | - | + |
| hsa-miR-320a | ENPP4 | - | - | - | + |
| hsa-miR-320a | ENPP6 | - | - | - | + |
| hsa-miR-320a | ENSA | - | - | - | + |
| hsa-miR-320a | ENTHD1 | - | - | - | + |
| hsa-miR-320a | ENTPD1 | - | - | - | + |
| hsa-miR-320a | ENTPD4 | - | - | - | + |
| hsa-miR-320a | EOGT | + | + | - | + |
| hsa-miR-320a | EOMES | - | - | - | + |
| hsa-miR-320a | EP400 | - | - | + | - |
| hsa-miR-320a | EPAS1 | - | - | - | + |
| hsa-miR-320a | EPB41 | - | - | + | - |
| hsa-miR-320a | EPB41L1 | - | - | - | + |
| hsa-miR-320a | EPB41L4B | - | - | - | + |
| hsa-miR-320a | EPB41L5 | - | - | - | + |
| hsa-miR-320a | EPC1 | - | - | - | + |
| hsa-miR-320a | EPHA3 | - | - | - | + |
| hsa-miR-320a | EPHA4 | - | - | + | + |
| hsa-miR-320a | EPHA7 | - | - | - | + |
| hsa-miR-320a | EPM2AIP1 | - | - | + | + |
| hsa-miR-320a | EPN2 | - | - | - | + |
| hsa-miR-320a | EPO | - | - | - | + |
| hsa-miR-320a | EPPIN | - | - | - | + |
| hsa-miR-320a | EPRS | - | - | - | + |
| hsa-miR-320a | EPS15L1 | + | - | - | - |
| hsa-miR-320a | EPSTI1 | - | - | - | + |
| hsa-miR-320a | EPT1 | - | - | - | + |
| hsa-miR-320a | ERAP1 | - | - | - | + |
| hsa-miR-320a | ERBB2IP | - | - | - | + |
| hsa-miR-320a | ERBB4 | - | - | - | + |
| hsa-miR-320a | ERC1 | - | - | + | - |
| hsa-miR-320a | ERCC1 | - | - | - | + |
| hsa-miR-320a | ERCC4 | - | - | - | + |
| hsa-miR-320a | ERCC6L2 | - | - | - | + |
| hsa-miR-320a | EREG | + | - | - | + |
| hsa-miR-320a | ERG | - | - | - | + |
| hsa-miR-320a | ERICH2 | - | + | - | - |
| hsa-miR-320a | ERMAP | - | - | - | + |
| hsa-miR-320a | ERMN | + | - | - | + |
| hsa-miR-320a | ERMP1 | - | - | - | + |
| hsa-miR-320a | ERO1LB | - | - | - | + |
| hsa-miR-320a | ERP44 | - | - | - | + |
| hsa-miR-320a | ESF1 | - | - | - | + |
| hsa-miR-320a | ESM1 | + | - | - | + |
| hsa-miR-320a | ESRRA | - | - | - | + |
| hsa-miR-320a | ESRRG | - | + | - | + |
| hsa-miR-320a | ESYT1 | - | - | - | + |
| hsa-miR-320a | ETFA | + | - | - | - |
| hsa-miR-320a | ETS1 | - | - | - | + |
| hsa-miR-320a | ETV1 | - | + | - | + |
| hsa-miR-320a | ETV3 | - | - | - | + |
| hsa-miR-320a | ETV5 | - | - | - | + |
| hsa-miR-320a | ETV6 | - | - | - | + |
| hsa-miR-320a | EVI5 | - | - | - | + |
| hsa-miR-320a | EVX2 | - | - | - | + |
| hsa-miR-320a | EWSR1 | - | - | + | - |
| hsa-miR-320a | EXO1 | - | + | - | + |
| hsa-miR-320a | EXOC1 | - | - | - | + |
| hsa-miR-320a | EXOC4 | - | - | - | + |
| hsa-miR-320a | EXOC6B | - | - | - | + |
| hsa-miR-320a | EXOC7 | - | - | - | + |
| hsa-miR-320a | EXPH5 | - | - | - | + |
| hsa-miR-320a | EXT1 | - | - | + | - |
| hsa-miR-320a | EXT2 | - | - | - | + |
| hsa-miR-320a | EYA1 | + | - | - | - |
| hsa-miR-320a | EYA3 | - | + | - | + |
| hsa-miR-320a | EYA4 | - | - | - | + |
| hsa-miR-320a | EYS | - | - | - | + |
| hsa-miR-320a | EZH2 | - | - | + | - |
| hsa-miR-320a | EZR | - | - | - | + |
| hsa-miR-320a | F11R | - | - | - | + |
| hsa-miR-320a | F13A1 | - | - | - | + |
| hsa-miR-320a | F2RL2 | - | - | - | + |
| hsa-miR-320a | F3 | - | - | - | + |
| hsa-miR-320a | F9 | - | - | - | + |
| hsa-miR-320a | FAAH2 | - | - | - | + |
| hsa-miR-320a | FADD | - | + | - | + |
| hsa-miR-320a | FADS2 | - | - | - | + |
| hsa-miR-320a | FAF2 | - | - | - | + |
| hsa-miR-320a | FAIM2 | - | - | - | + |
| hsa-miR-320a | FAM101B | - | - | - | + |
| hsa-miR-320a | FAM102A | - | - | + | + |
| hsa-miR-320a | FAM102B | - | - | - | + |
| hsa-miR-320a | FAM103A1 | - | - | - | + |
| hsa-miR-320a | FAM104A | - | - | - | + |
| hsa-miR-320a | FAM105B | - | - | - | + |
| hsa-miR-320a | FAM106A | - | - | - | + |
| hsa-miR-320a | FAM107A | - | - | - | + |
| hsa-miR-320a | FAM108B1 | - | - | - | + |
| hsa-miR-320a | FAM109B | - | - | - | + |
| hsa-miR-320a | FAM110B | - | - | - | + |
| hsa-miR-320a | FAM110C | - | - | - | + |
| hsa-miR-320a | FAM115C | - | - | - | + |
| hsa-miR-320a | FAM117B | - | + | + | + |
| hsa-miR-320a | FAM118B | - | - | - | + |
| hsa-miR-320a | FAM120B | - | - | - | + |
| hsa-miR-320a | FAM120C | - | + | - | + |
| hsa-miR-320a | FAM122B | - | - | - | + |
| hsa-miR-320a | FAM122C | - | - | - | + |
| hsa-miR-320a | FAM124A | - | - | - | + |
| hsa-miR-320a | FAM124B | - | - | - | + |
| hsa-miR-320a | FAM126B | - | + | - | - |
| hsa-miR-320a | FAM127B | - | - | + | - |
| hsa-miR-320a | FAM127C | - | - | - | + |
| hsa-miR-320a | FAM129A | - | - | - | + |
| hsa-miR-320a | FAM131B | - | + | - | - |
| hsa-miR-320a | FAM133A | - | - | - | + |
| hsa-miR-320a | FAM134B | - | - | - | + |
| hsa-miR-320a | FAM134C | - | + | - | - |
| hsa-miR-320a | FAM13A | - | - | - | + |
| hsa-miR-320a | FAM13B | - | - | - | + |
| hsa-miR-320a | FAM155B | - | - | - | + |
| hsa-miR-320a | FAM160B1 | - | + | - | + |
| hsa-miR-320a | FAM161B | - | - | - | + |
| hsa-miR-320a | FAM163A | - | - | - | + |
| hsa-miR-320a | FAM167A | - | - | - | + |
| hsa-miR-320a | FAM168A | - | - | - | + |
| hsa-miR-320a | FAM171B | - | - | - | + |
| hsa-miR-320a | FAM172A | - | - | - | + |
| hsa-miR-320a | FAM177A1 | - | - | - | + |
| hsa-miR-320a | FAM178A | - | - | - | + |
| hsa-miR-320a | FAM18B2-CDRT4 | - | - | - | + |
| hsa-miR-320a | FAM190A | - | - | - | + |
| hsa-miR-320a | FAM190B | - | - | - | + |
| hsa-miR-320a | FAM192A | - | - | - | + |
| hsa-miR-320a | FAM198B | - | - | - | + |
| hsa-miR-320a | FAM199X | - | + | - | + |
| hsa-miR-320a | FAM19A1 | - | - | - | + |
| hsa-miR-320a | FAM19A5 | - | - | - | + |
| hsa-miR-320a | FAM208A | - | - | - | + |
| hsa-miR-320a | FAM208B | - | + | + | + |
| hsa-miR-320a | FAM20B | - | - | - | + |
| hsa-miR-320a | FAM210B | - | - | - | + |
| hsa-miR-320a | FAM211A | - | - | - | + |
| hsa-miR-320a | FAM216B | - | - | - | + |
| hsa-miR-320a | FAM217B | - | - | - | + |
| hsa-miR-320a | FAM218A | - | - | - | + |
| hsa-miR-320a | FAM26D | - | - | - | + |
| hsa-miR-320a | FAM40B | - | - | - | + |
| hsa-miR-320a | FAM46A | - | - | - | + |
| hsa-miR-320a | FAM46B | - | - | + | - |
| hsa-miR-320a | FAM46C | - | - | - | + |
| hsa-miR-320a | FAM48A | - | - | - | + |
| hsa-miR-320a | FAM49B | - | + | - | + |
| hsa-miR-320a | FAM53B | - | + | - | + |
| hsa-miR-320a | FAM60A | - | - | + | - |
| hsa-miR-320a | FAM63B | - | + | - | + |
| hsa-miR-320a | FAM65B | - | - | - | + |
| hsa-miR-320a | FAM69A | - | - | - | + |
| hsa-miR-320a | FAM69B | + | - | - | - |
| hsa-miR-320a | FAM71B | - | - | - | + |
| hsa-miR-320a | FAM73A | - | - | - | + |
| hsa-miR-320a | FAM78A | - | - | - | + |
| hsa-miR-320a | FAM81A | - | - | - | + |
| hsa-miR-320a | FAM82B | - | - | - | + |
| hsa-miR-320a | FAM83A | - | - | - | + |
| hsa-miR-320a | FAM83B | + | - | - | - |
| hsa-miR-320a | FAM83G | - | - | + | - |
| hsa-miR-320a | FAM84A | - | - | - | + |
| hsa-miR-320a | FAM84B | - | + | - | + |
| hsa-miR-320a | FAM86C1 | - | - | - | + |
| hsa-miR-320a | FAM89A | + | + | - | + |
| hsa-miR-320a | FAM8A1 | - | - | - | + |
| hsa-miR-320a | FAR1 | - | - | - | + |
| hsa-miR-320a | FAT1 | - | - | - | + |
| hsa-miR-320a | FAT4 | - | - | - | + |
| hsa-miR-320a | FBN2 | - | - | - | + |
| hsa-miR-320a | FBN3 | - | - | - | + |
| hsa-miR-320a | FBXL13 | - | - | + | - |
| hsa-miR-320a | FBXL14 | - | - | - | + |
| hsa-miR-320a | FBXL22 | - | - | - | + |
| hsa-miR-320a | FBXL3 | - | - | - | + |
| hsa-miR-320a | FBXO11 | - | + | - | + |
| hsa-miR-320a | FBXO17 | - | - | - | + |
| hsa-miR-320a | FBXO28 | + | - | + | + |
| hsa-miR-320a | FBXO31 | - | - | + | - |
| hsa-miR-320a | FBXO32 | - | + | - | + |
| hsa-miR-320a | FBXO34 | - | - | - | + |
| hsa-miR-320a | FBXO38 | - | - | - | + |
| hsa-miR-320a | FBXO40 | - | - | - | + |
| hsa-miR-320a | FBXO41 | - | - | - | + |
| hsa-miR-320a | FBXO42 | - | - | - | + |
| hsa-miR-320a | FBXO44 | - | - | - | + |
| hsa-miR-320a | FBXO45 | + | - | - | + |
| hsa-miR-320a | FBXO5 | - | - | - | + |
| hsa-miR-320a | FBXO9 | - | - | - | + |
| hsa-miR-320a | FBXW11 | - | - | - | + |
| hsa-miR-320a | FBXW7 | - | - | - | + |
| hsa-miR-320a | FBXW8 | - | - | - | + |
| hsa-miR-320a | FCGR2A | - | - | - | + |
| hsa-miR-320a | FCGR3A | - | - | - | + |
| hsa-miR-320a | FCGR3B | - | - | - | + |
| hsa-miR-320a | FCHO2 | - | - | - | + |
| hsa-miR-320a | FCRL1 | - | - | - | + |
| hsa-miR-320a | FCRL4 | - | - | - | + |
| hsa-miR-320a | FDX1 | - | - | - | + |
| hsa-miR-320a | FECH | - | - | - | + |
| hsa-miR-320a | FEM1B | - | + | - | + |
| hsa-miR-320a | FEM1C | - | - | - | + |
| hsa-miR-320a | FEN1 | - | - | - | + |
| hsa-miR-320a | FERMT1 | - | - | - | + |
| hsa-miR-320a | FERMT2 | - | - | - | + |
| hsa-miR-320a | FGD1 | - | - | - | + |
| hsa-miR-320a | FGD4 | - | + | - | + |
| hsa-miR-320a | FGD5 | - | - | - | + |
| hsa-miR-320a | FGD6 | - | - | - | + |
| hsa-miR-320a | FGF1 | - | - | - | + |
| hsa-miR-320a | FGF19 | - | - | - | + |
| hsa-miR-320a | FGF2 | - | - | - | + |
| hsa-miR-320a | FGF23 | - | - | - | + |
| hsa-miR-320a | FGF5 | - | - | - | + |
| hsa-miR-320a | FGF9 | - | - | - | + |
| hsa-miR-320a | FGL1 | + | - | - | - |
| hsa-miR-320a | FGL2 | - | - | - | + |
| hsa-miR-320a | FHDC1 | - | - | - | + |
| hsa-miR-320a | FHL3 | - | - | + | - |
| hsa-miR-320a | FJX1 | - | - | - | + |
| hsa-miR-320a | FKBP14 | - | - | - | + |
| hsa-miR-320a | FKBP1A | - | + | + | + |
| hsa-miR-320a | FKBP5 | - | + | - | + |
| hsa-miR-320a | FKBP7 | - | - | - | + |
| hsa-miR-320a | FKSG79 | - | + | - | - |
| hsa-miR-320a | FLAD1 | - | - | + | - |
| hsa-miR-320a | FLNC | - | - | - | + |
| hsa-miR-320a | FLOT1 | - | - | + | - |
| hsa-miR-320a | FLRT3 | - | + | - | - |
| hsa-miR-320a | FLT1 | - | - | - | + |
| hsa-miR-320a | FMN1 | - | - | - | + |
| hsa-miR-320a | FMO1 | - | - | + | - |
| hsa-miR-320a | FMOD | - | - | - | + |
| hsa-miR-320a | FMR1 | - | - | - | + |
| hsa-miR-320a | FNBP1 | - | - | - | + |
| hsa-miR-320a | FNBP1L | - | - | - | + |
| hsa-miR-320a | FNBP4 | - | - | - | + |
| hsa-miR-320a | FNDC3B | - | - | - | + |
| hsa-miR-320a | FNIP1 | - | - | - | + |
| hsa-miR-320a | FNIP2 | - | - | - | + |
| hsa-miR-320a | FOSL1 | - | - | - | + |
| hsa-miR-320a | FOXA1 | - | - | - | + |
| hsa-miR-320a | FOXC1 | - | - | - | + |
| hsa-miR-320a | FOXE1 | - | - | - | + |
| hsa-miR-320a | FOXJ2 | - | - | - | + |
| hsa-miR-320a | FOXJ3 | - | - | - | + |
| hsa-miR-320a | FOXK1 | - | - | - | + |
| hsa-miR-320a | FOXK2 | - | - | - | + |
| hsa-miR-320a | FOXL1 | - | - | - | + |
| hsa-miR-320a | FOXL2 | - | - | - | + |
| hsa-miR-320a | FOXM1 | - | + | - | + |
| hsa-miR-320a | FOXN2 | - | - | - | + |
| hsa-miR-320a | FOXN3 | - | - | - | + |
| hsa-miR-320a | FOXO1 | - | - | - | + |
| hsa-miR-320a | FOXO3 | - | - | - | + |
| hsa-miR-320a | FOXO4 | - | - | - | + |
| hsa-miR-320a | FOXP1 | - | - | + | + |
| hsa-miR-320a | FOXQ1 | + | + | - | - |
| hsa-miR-320a | FRA10AC1 | - | - | - | + |
| hsa-miR-320a | FRAS1 | - | - | - | + |
| hsa-miR-320a | FRAT2 | - | - | + | + |
| hsa-miR-320a | FREM2 | - | - | - | + |
| hsa-miR-320a | FRMD3 | - | - | - | + |
| hsa-miR-320a | FRMD5 | - | - | - | + |
| hsa-miR-320a | FRMD6 | - | - | - | + |
| hsa-miR-320a | FRMPD4 | - | - | - | + |
| hsa-miR-320a | FRRS1L | - | + | - | - |
| hsa-miR-320a | FRS2 | - | - | - | + |
| hsa-miR-320a | FSCB | - | - | - | + |
| hsa-miR-320a | FSD1L | - | - | - | + |
| hsa-miR-320a | FSD2 | - | - | - | + |
| hsa-miR-320a | FST | - | - | - | + |
| hsa-miR-320a | FTL | - | - | - | + |
| hsa-miR-320a | FUS | + | - | - | - |
| hsa-miR-320a | FUT10 | - | - | - | + |
| hsa-miR-320a | FUT4 | - | - | - | + |
| hsa-miR-320a | FUT8 | - | - | - | + |
| hsa-miR-320a | FXC1 | - | - | - | + |
| hsa-miR-320a | FYB | - | - | - | + |
| hsa-miR-320a | FYN | - | - | - | + |
| hsa-miR-320a | FZD3 | - | - | - | + |
| hsa-miR-320a | FZD4 | - | - | - | + |
| hsa-miR-320a | FZD5 | - | - | - | + |
| hsa-miR-320a | FZD7 | - | - | - | + |
| hsa-miR-320a | G0S2 | - | - | - | + |
| hsa-miR-320a | G3BP1 | - | - | + | + |
| hsa-miR-320a | GAB1 | - | - | - | + |
| hsa-miR-320a | GAB3 | - | - | - | + |
| hsa-miR-320a | GABBR2 | - | - | - | + |
| hsa-miR-320a | GABPA | - | - | - | + |
| hsa-miR-320a | GABPB1 | - | + | - | - |
| hsa-miR-320a | GABRA1 | - | - | - | + |
| hsa-miR-320a | GABRA4 | - | - | - | + |
| hsa-miR-320a | GABRA5 | + | + | - | + |
| hsa-miR-320a | GABRB2 | - | + | - | - |
| hsa-miR-320a | GABRB3 | - | - | - | + |
| hsa-miR-320a | GABRG1 | - | + | - | + |
| hsa-miR-320a | GABRG2 | - | - | - | + |
| hsa-miR-320a | GABRP | - | - | - | + |
| hsa-miR-320a | GADD45G | - | - | - | + |
| hsa-miR-320a | GAGE1 | + | - | - | - |
| hsa-miR-320a | GAGE10 | + | - | - | - |
| hsa-miR-320a | GAGE12B | + | - | - | - |
| hsa-miR-320a | GAGE12C | + | - | - | + |
| hsa-miR-320a | GAGE12D | + | - | - | + |
| hsa-miR-320a | GAGE12E | + | - | - | + |
| hsa-miR-320a | GAGE12F | + | - | - | + |
| hsa-miR-320a | GAGE12G | + | - | - | + |
| hsa-miR-320a | GAGE12H | + | - | - | + |
| hsa-miR-320a | GAGE12I | + | - | - | - |
| hsa-miR-320a | GAGE12J | + | - | - | + |
| hsa-miR-320a | GAGE13 | + | - | - | - |
| hsa-miR-320a | GAGE2A | + | - | - | + |
| hsa-miR-320a | GAGE2C | + | - | - | - |
| hsa-miR-320a | GAGE2D | + | - | - | - |
| hsa-miR-320a | GAGE2E | + | - | - | - |
| hsa-miR-320a | GALC | - | - | - | + |
| hsa-miR-320a | GALE | - | - | - | + |
| hsa-miR-320a | GALM | - | - | - | + |
| hsa-miR-320a | GALNT1 | - | - | - | + |
| hsa-miR-320a | GALNT10 | - | - | - | + |
| hsa-miR-320a | GALNT12 | - | - | - | + |
| hsa-miR-320a | GALNT2 | - | - | - | + |
| hsa-miR-320a | GALNT3 | - | - | - | + |
| hsa-miR-320a | GALNT4 | - | - | - | + |
| hsa-miR-320a | GALNT7 | - | - | - | + |
| hsa-miR-320a | GALNTL4 | - | - | - | + |
| hsa-miR-320a | GAPDH | - | - | + | - |
| hsa-miR-320a | GART | - | - | + | - |
| hsa-miR-320a | GATA1 | - | - | - | + |
| hsa-miR-320a | GATA2 | - | - | - | + |
| hsa-miR-320a | GATA6 | - | - | - | + |
| hsa-miR-320a | GATAD2B | - | - | - | + |
| hsa-miR-320a | GBE1 | - | - | - | + |
| hsa-miR-320a | GBP4 | - | - | - | + |
| hsa-miR-320a | GBX2 | - | - | - | + |
| hsa-miR-320a | GCG | + | + | - | + |
| hsa-miR-320a | GCLC | - | - | - | + |
| hsa-miR-320a | GCM1 | - | - | - | + |
| hsa-miR-320a | GCM2 | + | - | - | + |
| hsa-miR-320a | GCN1L1 | - | - | - | + |
| hsa-miR-320a | GCNT2 | - | - | - | + |
| hsa-miR-320a | GCNT4 | - | + | - | - |
| hsa-miR-320a | GCOM1 | - | - | - | + |
| hsa-miR-320a | GCSAML | - | - | - | + |
| hsa-miR-320a | GDA | - | - | - | + |
| hsa-miR-320a | GDAP1 | - | - | - | + |
| hsa-miR-320a | GDE1 | - | - | - | + |
| hsa-miR-320a | GDF5 | - | - | - | + |
| hsa-miR-320a | GDF6 | - | - | - | + |
| hsa-miR-320a | GDI2 | - | - | - | + |
| hsa-miR-320a | GEMIN5 | - | - | - | + |
| hsa-miR-320a | GFPT1 | - | - | - | + |
| hsa-miR-320a | GFPT2 | - | - | - | + |
| hsa-miR-320a | GFRA1 | - | - | - | + |
| hsa-miR-320a | GGA1 | - | - | + | - |
| hsa-miR-320a | GGA2 | - | - | - | + |
| hsa-miR-320a | GGT6 | - | - | - | + |
| hsa-miR-320a | GHITM | - | + | - | + |
| hsa-miR-320a | GHR | - | - | - | + |
| hsa-miR-320a | GHSR | - | - | + | - |
| hsa-miR-320a | GIMAP1 | - | - | - | + |
| hsa-miR-320a | GIMAP5 | - | - | - | + |
| hsa-miR-320a | GIMAP6 | - | - | - | + |
| hsa-miR-320a | GIMAP7 | + | - | - | - |
| hsa-miR-320a | GINS3 | - | - | - | + |
| hsa-miR-320a | GIPC2 | - | - | - | + |
| hsa-miR-320a | GIPC3 | - | - | - | + |
| hsa-miR-320a | GIT2 | - | - | - | + |
| hsa-miR-320a | GJA1 | - | - | - | + |
| hsa-miR-320a | GJA5 | - | + | - | - |
| hsa-miR-320a | GJB1 | - | - | - | + |
| hsa-miR-320a | GJB2 | - | - | - | + |
| hsa-miR-320a | GJB4 | + | - | - | + |
| hsa-miR-320a | GJC1 | - | - | - | + |
| hsa-miR-320a | GLCCI1 | - | - | - | + |
| hsa-miR-320a | GLDN | - | - | - | + |
| hsa-miR-320a | GLI3 | - | + | - | + |
| hsa-miR-320a | GLIPR2 | - | - | - | + |
| hsa-miR-320a | GLIS3 | - | - | - | + |
| hsa-miR-320a | GLOD4 | - | - | - | + |
| hsa-miR-320a | GLP2R | - | - | - | + |
| hsa-miR-320a | GLRX | + | - | - | + |
| hsa-miR-320a | GLS | - | - | - | + |
| hsa-miR-320a | GLTSCR1L | - | - | - | + |
| hsa-miR-320a | GLUD1 | - | - | - | + |
| hsa-miR-320a | GLUL | - | - | + | - |
| hsa-miR-320a | GLYATL2 | - | - | - | + |
| hsa-miR-320a | GMCL1 | - | - | - | + |
| hsa-miR-320a | GMNC | - | - | - | + |
| hsa-miR-320a | GNA12 | - | - | - | + |
| hsa-miR-320a | GNA14 | - | - | - | + |
| hsa-miR-320a | GNAI1 | - | + | + | + |
| hsa-miR-320a | GNAI3 | - | + | - | - |
| hsa-miR-320a | GNAL | - | - | - | + |
| hsa-miR-320a | GNAO1 | - | - | - | + |
| hsa-miR-320a | GNAQ | - | - | - | + |
| hsa-miR-320a | GNAS | - | - | + | - |
| hsa-miR-320a | GNB4 | - | - | - | + |
| hsa-miR-320a | GNE | - | + | - | - |
| hsa-miR-320a | GNG12 | - | - | - | + |
| hsa-miR-320a | GNG2 | - | - | - | + |
| hsa-miR-320a | GNL1 | - | - | - | + |
| hsa-miR-320a | GNPDA1 | - | - | - | + |
| hsa-miR-320a | GNPNAT1 | - | - | - | + |
| hsa-miR-320a | GOLGA1 | - | + | - | + |
| hsa-miR-320a | GOLGA2 | - | - | - | + |
| hsa-miR-320a | GOLGA3 | - | - | - | + |
| hsa-miR-320a | GOLGA4 | - | - | - | + |
| hsa-miR-320a | GOLGA6A | - | - | - | + |
| hsa-miR-320a | GOLGA8B | - | - | - | + |
| hsa-miR-320a | GOLM1 | + | - | + | + |
| hsa-miR-320a | GOLPH3 | - | - | - | + |
| hsa-miR-320a | GOLPH3L | - | - | - | + |
| hsa-miR-320a | GOLT1B | - | + | - | + |
| hsa-miR-320a | GOPC | - | + | - | - |
| hsa-miR-320a | GORASP2 | - | - | + | - |
| hsa-miR-320a | GOSR1 | - | - | - | + |
| hsa-miR-320a | GOSR2 | - | - | - | + |
| hsa-miR-320a | GOT2 | - | - | - | + |
| hsa-miR-320a | GP2 | - | - | - | + |
| hsa-miR-320a | GPALPP1 | + | - | - | - |
| hsa-miR-320a | GPAM | - | - | - | + |
| hsa-miR-320a | GPBP1L1 | - | - | - | + |
| hsa-miR-320a | GPC1 | - | - | - | + |
| hsa-miR-320a | GPC6 | - | - | - | + |
| hsa-miR-320a | GPCPD1 | - | + | - | + |
| hsa-miR-320a | GPD2 | - | + | - | + |
| hsa-miR-320a | GPHA2 | - | - | - | + |
| hsa-miR-320a | GPHN | - | - | - | + |
| hsa-miR-320a | GPM6A | - | - | - | + |
| hsa-miR-320a | GPN1 | - | - | + | - |
| hsa-miR-320a | GPNMB | - | + | - | - |
| hsa-miR-320a | GPR107 | - | - | - | + |
| hsa-miR-320a | GPR110 | - | - | - | + |
| hsa-miR-320a | GPR111 | - | - | - | + |
| hsa-miR-320a | GPR137B | - | - | - | + |
| hsa-miR-320a | GPR137C | - | - | - | + |
| hsa-miR-320a | GPR149 | - | - | - | + |
| hsa-miR-320a | GPR155 | - | - | - | + |
| hsa-miR-320a | GPR158 | - | - | - | + |
| hsa-miR-320a | GPR17 | - | - | - | + |
| hsa-miR-320a | GPR171 | + | - | - | + |
| hsa-miR-320a | GPR173 | - | - | - | + |
| hsa-miR-320a | GPR174 | + | - | - | + |
| hsa-miR-320a | GPR176 | - | - | - | + |
| hsa-miR-320a | GPR180 | - | - | - | + |
| hsa-miR-320a | GPR55 | - | - | - | + |
| hsa-miR-320a | GPR84 | - | - | - | + |
| hsa-miR-320a | GPR88 | - | - | - | + |
| hsa-miR-320a | GPR89B | - | - | - | + |
| hsa-miR-320a | GPRASP1 | - | + | - | + |
| hsa-miR-320a | GPT2 | - | - | + | + |
| hsa-miR-320a | GPX5 | - | - | - | + |
| hsa-miR-320a | GRAMD1B | - | - | - | + |
| hsa-miR-320a | GRAMD4 | - | - | - | + |
| hsa-miR-320a | GRAP2 | - | - | - | + |
| hsa-miR-320a | GRASP | + | + | - | + |
| hsa-miR-320a | GRB10 | - | - | - | + |
| hsa-miR-320a | GRB2 | - | + | - | - |
| hsa-miR-320a | GREB1 | - | - | - | + |
| hsa-miR-320a | GRHL2 | - | - | - | + |
| hsa-miR-320a | GRIA1 | - | - | - | + |
| hsa-miR-320a | GRIA2 | - | - | - | + |
| hsa-miR-320a | GRIA4 | + | - | - | + |
| hsa-miR-320a | GRID1 | - | - | - | + |
| hsa-miR-320a | GRID2 | - | - | - | + |
| hsa-miR-320a | GRIN2A | - | - | - | + |
| hsa-miR-320a | GRIN2C | - | - | - | + |
| hsa-miR-320a | GRIN2D | - | - | - | + |
| hsa-miR-320a | GRIN3A | - | - | - | + |
| hsa-miR-320a | GRIP1 | - | - | - | + |
| hsa-miR-320a | GRIPAP1 | - | - | - | + |
| hsa-miR-320a | GRK6 | - | - | + | - |
| hsa-miR-320a | GRM1 | - | - | - | + |
| hsa-miR-320a | GRM5 | - | - | - | + |
| hsa-miR-320a | GRM6 | - | - | - | + |
| hsa-miR-320a | GRSF1 | - | - | - | + |
| hsa-miR-320a | GRTP1 | + | - | - | - |
| hsa-miR-320a | GRWD1 | - | - | + | - |
| hsa-miR-320a | GSG2 | - | + | - | + |
| hsa-miR-320a | GSK3A | - | - | - | + |
| hsa-miR-320a | GSK3B | - | - | - | + |
| hsa-miR-320a | GSKIP | + | + | - | + |
| hsa-miR-320a | GSPT1 | - | + | - | + |
| hsa-miR-320a | GSPT2 | + | + | - | + |
| hsa-miR-320a | GSS | - | - | - | + |
| hsa-miR-320a | GTF2A1 | + | - | - | + |
| hsa-miR-320a | GTF2A2 | - | - | - | + |
| hsa-miR-320a | GTF2F2 | - | - | - | + |
| hsa-miR-320a | GTF2H1 | - | - | - | + |
| hsa-miR-320a | GTF2H5 | + | - | - | - |
| hsa-miR-320a | GTF3C3 | - | - | - | + |
| hsa-miR-320a | GTF3C4 | - | - | + | - |
| hsa-miR-320a | GTPBP2 | - | + | + | - |
| hsa-miR-320a | GTPBP8 | + | - | - | - |
| hsa-miR-320a | GUCA1A | - | - | - | + |
| hsa-miR-320a | GUCY1A2 | - | - | - | + |
| hsa-miR-320a | GXYLT1 | - | + | + | + |
| hsa-miR-320a | GYG1 | - | - | - | + |
| hsa-miR-320a | GYS1 | - | - | - | + |
| hsa-miR-320a | GZF1 | - | - | - | + |
| hsa-miR-320a | H2AFV | - | - | - | + |
| hsa-miR-320a | H2AFX | - | - | + | - |
| hsa-miR-320a | H2AFY2 | - | - | - | + |
| hsa-miR-320a | H3F3B | - | - | - | + |
| hsa-miR-320a | H6PD | - | - | - | + |
| hsa-miR-320a | HACD3 | - | - | - | + |
| hsa-miR-320a | HADH | + | + | - | - |
| hsa-miR-320a | HADHB | - | - | - | + |
| hsa-miR-320a | HAO1 | - | - | - | + |
| hsa-miR-320a | HAS2 | - | - | - | + |
| hsa-miR-320a | HAS3 | - | + | - | + |
| hsa-miR-320a | HAUS2 | - | - | - | + |
| hsa-miR-320a | HAUS5 | - | - | - | + |
| hsa-miR-320a | HAUS6 | - | - | + | - |
| hsa-miR-320a | HBP1 | - | - | - | + |
| hsa-miR-320a | HBS1L | - | - | - | + |
| hsa-miR-320a | HCAR2 | - | - | - | + |
| hsa-miR-320a | HCAR3 | - | - | - | + |
| hsa-miR-320a | HCCS | - | - | - | + |
| hsa-miR-320a | HCFC2 | - | - | - | + |
| hsa-miR-320a | HCN4 | - | - | - | + |
| hsa-miR-320a | HDAC4 | - | - | - | + |
| hsa-miR-320a | HDGF | - | - | - | + |
| hsa-miR-320a | HDLBP | - | - | - | + |
| hsa-miR-320a | HDX | - | + | - | + |
| hsa-miR-320a | HEATR1 | - | - | - | + |
| hsa-miR-320a | HEATR5B | - | - | - | + |
| hsa-miR-320a | HEBP1 | + | - | - | - |
| hsa-miR-320a | HECA | - | - | - | + |
| hsa-miR-320a | HECTD2 | + | + | - | - |
| hsa-miR-320a | HEL-S-1a | - | + | - | - |
| hsa-miR-320a | HELZ | - | + | - | + |
| hsa-miR-320a | HEPACAM2 | - | - | - | + |
| hsa-miR-320a | HEPHL1 | - | - | - | + |
| hsa-miR-320a | HEPN1 | - | - | - | + |
| hsa-miR-320a | HERC1 | - | - | - | + |
| hsa-miR-320a | HERC3 | - | - | - | + |
| hsa-miR-320a | HERC5 | - | - | - | + |
| hsa-miR-320a | HERPUD1 | - | - | - | + |
| hsa-miR-320a | HES1 | - | - | - | + |
| hsa-miR-320a | HES6 | - | - | + | - |
| hsa-miR-320a | HEY1 | - | + | - | + |
| hsa-miR-320a | HGF | - | - | - | + |
| hsa-miR-320a | HGSNAT | - | - | - | + |
| hsa-miR-320a | HHIPL1 | - | - | - | + |
| hsa-miR-320a | HHLA2 | + | - | - | - |
| hsa-miR-320a | HIAT1 | - | - | - | + |
| hsa-miR-320a | HIC1 | - | - | - | + |
| hsa-miR-320a | HIC2 | - | - | - | + |
| hsa-miR-320a | HIF1A | - | - | - | + |
| hsa-miR-320a | HIPK1 | - | + | - | + |
| hsa-miR-320a | HIPK2 | - | - | - | + |
| hsa-miR-320a | HIPK3 | - | - | - | + |
| hsa-miR-320a | HIST1H1A | - | - | + | - |
| hsa-miR-320a | HIST1H1E | - | - | + | - |
| hsa-miR-320a | HIST1H2BC | + | - | + | - |
| hsa-miR-320a | HIST1H2BH | - | - | + | - |
| hsa-miR-320a | HIST1H2BJ | - | - | + | - |
| hsa-miR-320a | HIST1H2BK | - | - | + | - |
| hsa-miR-320a | HIST1H2BL | - | - | + | - |
| hsa-miR-320a | HIST1H2BO | - | - | + | - |
| hsa-miR-320a | HIST1H3B | - | - | + | - |
| hsa-miR-320a | HIST1H3H | - | - | + | - |
| hsa-miR-320a | HIST2H2AA3 | - | - | + | - |
| hsa-miR-320a | HIST2H2BE | - | - | - | + |
| hsa-miR-320a | HIST2H3A | - | - | + | - |
| hsa-miR-320a | HIST2H4B | - | - | + | - |
| hsa-miR-320a | HIVEP2 | - | + | - | - |
| hsa-miR-320a | HK2 | - | - | - | + |
| hsa-miR-320a | HKDC1 | - | - | - | + |
| hsa-miR-320a | HLA-DOA | - | - | - | + |
| hsa-miR-320a | HLA-DPA1 | + | + | - | - |
| hsa-miR-320a | HLA-DQB1 | - | - | - | + |
| hsa-miR-320a | HLA-DRB5 | - | - | - | + |
| hsa-miR-320a | HLCS | - | + | - | + |
| hsa-miR-320a | HLF | - | - | - | + |
| hsa-miR-320a | HLTF | + | + | - | + |
| hsa-miR-320a | HM13 | - | - | - | + |
| hsa-miR-320a | HMG20A | - | - | - | + |
| hsa-miR-320a | HMG20B | - | - | - | + |
| hsa-miR-320a | HMGA1 | - | - | - | + |
| hsa-miR-320a | HMGB3 | - | - | - | + |
| hsa-miR-320a | HMGCR | - | - | - | + |
| hsa-miR-320a | HMGN3 | - | - | - | + |
| hsa-miR-320a | HMX3 | - | - | - | + |
| hsa-miR-320a | HN1L | - | + | - | + |
| hsa-miR-320a | HNF1A | - | - | + | + |
| hsa-miR-320a | HNMT | - | - | - | + |
| hsa-miR-320a | HNRNPA2B1 | - | - | - | + |
| hsa-miR-320a | HNRNPA3 | - | - | - | + |
| hsa-miR-320a | HNRNPC | - | - | - | + |
| hsa-miR-320a | HNRNPF | - | + | - | + |
| hsa-miR-320a | HNRNPH2 | - | - | - | + |
| hsa-miR-320a | HNRNPH3 | - | - | - | + |
| hsa-miR-320a | HNRNPM | - | - | - | + |
| hsa-miR-320a | HNRNPR | - | - | - | + |
| hsa-miR-320a | HNRNPU | - | - | - | + |
| hsa-miR-320a | HNRNPUL1 | - | - | + | + |
| hsa-miR-320a | HNRPLL | - | - | - | + |
| hsa-miR-320a | HOPX | - | - | - | + |
| hsa-miR-320a | HOXA10 | + | - | + | + |
| hsa-miR-320a | HOXA3 | - | - | - | + |
| hsa-miR-320a | HOXA5 | + | + | - | + |
| hsa-miR-320a | HOXA9 | - | - | - | + |
| hsa-miR-320a | HOXB5 | - | - | - | + |
| hsa-miR-320a | HOXB8 | - | - | + | - |
| hsa-miR-320a | HOXC10 | - | - | - | + |
| hsa-miR-320a | HOXC13 | - | - | - | + |
| hsa-miR-320a | HOXC8 | - | - | - | + |
| hsa-miR-320a | HOXD1 | - | + | - | + |
| hsa-miR-320a | HOXD3 | - | - | - | + |
| hsa-miR-320a | HP1BP3 | - | - | - | + |
| hsa-miR-320a | HPCAL4 | - | - | - | + |
| hsa-miR-320a | HPGD | - | - | - | + |
| hsa-miR-320a | HPRT1 | - | - | - | + |
| hsa-miR-320a | HPS3 | - | - | - | + |
| hsa-miR-320a | HPSE | - | - | - | + |
| hsa-miR-320a | HRH1 | - | - | - | + |
| hsa-miR-320a | HRNR | - | - | - | + |
| hsa-miR-320a | HS2ST1 | - | + | - | + |
| hsa-miR-320a | HS3ST3B1 | - | - | - | + |
| hsa-miR-320a | HS6ST1 | - | - | - | + |
| hsa-miR-320a | HSD17B10 | - | - | + | - |
| hsa-miR-320a | HSD17B12 | - | - | - | + |
| hsa-miR-320a | HSD17B13 | - | - | - | + |
| hsa-miR-320a | HSDL1 | - | - | - | + |
| hsa-miR-320a | HSF2 | - | - | - | + |
| hsa-miR-320a | HSF5 | - | - | - | + |
| hsa-miR-320a | HSP90AB1 | - | - | + | - |
| hsa-miR-320a | HSPA12A | - | - | - | + |
| hsa-miR-320a | HSPA4 | - | - | + | - |
| hsa-miR-320a | HSPA4L | - | - | + | - |
| hsa-miR-320a | HSPA5 | - | - | - | + |
| hsa-miR-320a | HSPA8 | - | - | + | - |
| hsa-miR-320a | HSPA9 | - | - | + | - |
| hsa-miR-320a | HSPB6 | - | - | + | - |
| hsa-miR-320a | HSPH1 | - | - | - | + |
| hsa-miR-320a | HTR2C | - | - | - | + |
| hsa-miR-320a | HTR7 | - | - | - | + |
| hsa-miR-320a | HTRA1 | - | - | - | + |
| hsa-miR-320a | HUNK | - | - | - | + |
| hsa-miR-320a | HUWE1 | - | - | + | - |
| hsa-miR-320a | ICAM1 | - | - | - | + |
| hsa-miR-320a | ICK | - | - | - | + |
| hsa-miR-320a | ICMT | - | - | - | + |
| hsa-miR-320a | IDE | - | + | - | + |
| hsa-miR-320a | IDH3G | - | - | + | - |
| hsa-miR-320a | IDI2 | + | - | - | - |
| hsa-miR-320a | IFFO2 | - | - | + | - |
| hsa-miR-320a | IFI16 | - | - | - | + |
| hsa-miR-320a | IFIT2 | - | - | - | + |
| hsa-miR-320a | IFIT5 | - | - | - | + |
| hsa-miR-320a | IFNAR1 | - | - | - | + |
| hsa-miR-320a | IFNGR1 | - | - | - | + |
| hsa-miR-320a | IFNW1 | + | - | - | - |
| hsa-miR-320a | IFT57 | - | - | - | + |
| hsa-miR-320a | IGF1 | - | - | - | + |
| hsa-miR-320a | IGF1R | - | - | + | + |
| hsa-miR-320a | IGF2 | - | - | + | + |
| hsa-miR-320a | IGF2AS | - | - | - | + |
| hsa-miR-320a | IGF2BP1 | - | - | + | + |
| hsa-miR-320a | IGF2BP2 | - | - | - | + |
| hsa-miR-320a | IGF2BP3 | - | + | + | - |
| hsa-miR-320a | IGFBP3 | - | - | - | + |
| hsa-miR-320a | IGFBP5 | - | - | - | + |
| hsa-miR-320a | IGFL3 | - | - | - | + |
| hsa-miR-320a | IGHMBP2 | - | - | - | + |
| hsa-miR-320a | IGSF1 | - | - | - | + |
| hsa-miR-320a | IGSF3 | - | - | - | + |
| hsa-miR-320a | IGSF5 | - | - | - | + |
| hsa-miR-320a | IKBIP | - | - | + | - |
| hsa-miR-320a | IKZF1 | - | - | - | + |
| hsa-miR-320a | IKZF2 | - | - | - | + |
| hsa-miR-320a | IKZF5 | - | - | - | + |
| hsa-miR-320a | IL12B | - | + | - | + |
| hsa-miR-320a | IL17D | - | - | - | + |
| hsa-miR-320a | IL17RA | - | - | - | + |
| hsa-miR-320a | IL17RD | - | - | - | + |
| hsa-miR-320a | IL1RAP | - | + | - | - |
| hsa-miR-320a | IL1RL1 | - | - | - | + |
| hsa-miR-320a | IL21 | - | - | - | + |
| hsa-miR-320a | IL22RA2 | - | - | - | + |
| hsa-miR-320a | IL4 | - | - | - | + |
| hsa-miR-320a | IL5RA | + | - | - | - |
| hsa-miR-320a | IL6R | - | - | - | + |
| hsa-miR-320a | IL6ST | - | - | - | + |
| hsa-miR-320a | ILF3 | - | - | + | + |
| hsa-miR-320a | ILK | - | - | - | + |
| hsa-miR-320a | IMP4 | - | - | + | - |
| hsa-miR-320a | IMPACT | - | - | - | + |
| hsa-miR-320a | IMPAD1 | - | - | - | + |
| hsa-miR-320a | IMPG2 | - | - | - | + |
| hsa-miR-320a | INA | - | + | - | + |
| hsa-miR-320a | INADL | - | - | - | + |
| hsa-miR-320a | ING5 | - | + | - | + |
| hsa-miR-320a | INHBB | - | - | - | + |
| hsa-miR-320a | INHBE | - | - | - | + |
| hsa-miR-320a | INO80D | - | - | + | + |
| hsa-miR-320a | INPP5F | - | - | - | + |
| hsa-miR-320a | INPP5K | - | - | - | + |
| hsa-miR-320a | INSC | - | - | - | + |
| hsa-miR-320a | INSIG2 | - | - | - | + |
| hsa-miR-320a | INSL5 | - | - | - | + |
| hsa-miR-320a | INSM1 | - | - | - | + |
| hsa-miR-320a | INSM2 | + | - | - | + |
| hsa-miR-320a | INSR | - | - | - | + |
| hsa-miR-320a | INTS2 | - | - | - | + |
| hsa-miR-320a | INTS3 | - | - | + | - |
| hsa-miR-320a | INTS7 | - | - | - | + |
| hsa-miR-320a | IP6K1 | - | - | - | + |
| hsa-miR-320a | IPCEF1 | - | - | - | + |
| hsa-miR-320a | IPMK | - | - | - | + |
| hsa-miR-320a | IPO5 | - | + | - | + |
| hsa-miR-320a | IPO7 | - | + | - | + |
| hsa-miR-320a | IPO8 | - | - | - | + |
| hsa-miR-320a | IPO9 | - | - | - | + |
| hsa-miR-320a | IPPK | - | - | - | + |
| hsa-miR-320a | IQCE | - | - | - | + |
| hsa-miR-320a | IQGAP1 | - | - | - | + |
| hsa-miR-320a | IQSEC1 | - | - | - | + |
| hsa-miR-320a | IRAK2 | - | - | - | + |
| hsa-miR-320a | IRAK3 | - | - | - | + |
| hsa-miR-320a | IRAK4 | - | - | - | + |
| hsa-miR-320a | IREB2 | - | - | - | + |
| hsa-miR-320a | IRF2 | - | - | - | + |
| hsa-miR-320a | IRF2BP2 | - | - | + | + |
| hsa-miR-320a | IRF2BPL | - | - | - | + |
| hsa-miR-320a | IRF4 | - | - | - | + |
| hsa-miR-320a | IRF6 | + | + | - | + |
| hsa-miR-320a | ISCA1 | - | - | - | + |
| hsa-miR-320a | ISOC1 | - | - | - | + |
| hsa-miR-320a | ISX | - | - | - | + |
| hsa-miR-320a | ISYNA1 | - | - | + | - |
| hsa-miR-320a | ITCH | - | - | - | + |
| hsa-miR-320a | ITFG3 | - | - | + | - |
| hsa-miR-320a | ITGA10 | - | - | - | + |
| hsa-miR-320a | ITGA2 | - | - | - | + |
| hsa-miR-320a | ITGAM | - | - | - | + |
| hsa-miR-320a | ITGAV | - | - | - | + |
| hsa-miR-320a | ITGB1 | + | - | - | + |
| hsa-miR-320a | ITGB3 | - | - | + | - |
| hsa-miR-320a | ITGB5 | - | - | - | + |
| hsa-miR-320a | ITGB8 | - | - | - | + |
| hsa-miR-320a | ITIH4 | - | - | - | + |
| hsa-miR-320a | ITIH6 | - | - | - | + |
| hsa-miR-320a | ITK | - | - | - | + |
| hsa-miR-320a | ITM2B | - | - | + | - |
| hsa-miR-320a | ITPK1 | - | + | - | - |
| hsa-miR-320a | ITPKB | - | - | - | + |
| hsa-miR-320a | ITPRIPL2 | - | - | + | + |
| hsa-miR-320a | ITSN2 | + | - | - | - |
| hsa-miR-320a | IVNS1ABP | - | - | + | + |
| hsa-miR-320a | IYD | - | - | - | + |
| hsa-miR-320a | JAK2 | - | + | - | + |
| hsa-miR-320a | JAK3 | - | - | - | + |
| hsa-miR-320a | JAKMIP3 | - | - | - | + |
| hsa-miR-320a | JAM2 | - | - | - | + |
| hsa-miR-320a | JAM3 | - | - | - | + |
| hsa-miR-320a | JARID2 | - | - | - | + |
| hsa-miR-320a | JMJD4 | - | - | - | + |
| hsa-miR-320a | JMJD6 | - | - | - | + |
| hsa-miR-320a | JMJD7 | - | - | - | + |
| hsa-miR-320a | JMY | - | + | - | + |
| hsa-miR-320a | JOSD1 | - | - | - | + |
| hsa-miR-320a | JPH1 | - | - | - | + |
| hsa-miR-320a | JPH2 | - | - | - | + |
| hsa-miR-320a | JPH4 | - | - | - | + |
| hsa-miR-320a | KANK2 | - | - | - | + |
| hsa-miR-320a | KANSL1 | - | - | - | + |
| hsa-miR-320a | KANSL1L | - | - | - | + |
| hsa-miR-320a | KANSL3 | - | - | - | + |
| hsa-miR-320a | KAT2B | - | - | - | + |
| hsa-miR-320a | KAT6B | - | + | - | + |
| hsa-miR-320a | KATNAL1 | - | + | - | + |
| hsa-miR-320a | KATNB1 | - | - | - | + |
| hsa-miR-320a | KAZN | - | - | - | + |
| hsa-miR-320a | KBTBD11 | - | - | - | + |
| hsa-miR-320a | KBTBD12 | - | - | - | + |
| hsa-miR-320a | KBTBD2 | - | - | - | + |
| hsa-miR-320a | KBTBD3 | + | - | - | - |
| hsa-miR-320a | KBTBD6 | - | + | - | + |
| hsa-miR-320a | KBTBD8 | - | - | - | + |
| hsa-miR-320a | KCMF1 | - | - | - | + |
| hsa-miR-320a | KCNA1 | - | - | - | + |
| hsa-miR-320a | KCNAB1 | - | - | - | + |
| hsa-miR-320a | KCNAB2 | - | - | - | + |
| hsa-miR-320a | KCNC3 | - | - | - | + |
| hsa-miR-320a | KCND2 | - | - | - | + |
| hsa-miR-320a | KCNG3 | - | - | - | + |
| hsa-miR-320a | KCNH1 | - | - | - | + |
| hsa-miR-320a | KCNH7 | - | - | - | + |
| hsa-miR-320a | KCNIP3 | - | - | - | + |
| hsa-miR-320a | KCNJ10 | - | - | - | + |
| hsa-miR-320a | KCNJ12 | - | - | - | + |
| hsa-miR-320a | KCNJ16 | - | - | - | + |
| hsa-miR-320a | KCNJ3 | - | - | - | + |
| hsa-miR-320a | KCNK10 | - | - | - | + |
| hsa-miR-320a | KCNK2 | - | - | - | + |
| hsa-miR-320a | KCNMB4 | - | - | - | + |
| hsa-miR-320a | KCNQ1 | - | - | - | + |
| hsa-miR-320a | KCNRG | - | - | - | + |
| hsa-miR-320a | KCNS3 | + | + | - | + |
| hsa-miR-320a | KCNV1 | - | - | - | + |
| hsa-miR-320a | KCTD10 | - | - | - | + |
| hsa-miR-320a | KCTD18 | - | - | - | + |
| hsa-miR-320a | KCTD20 | - | - | - | + |
| hsa-miR-320a | KCTD3 | - | - | - | + |
| hsa-miR-320a | KCTD7 | - | - | + | - |
| hsa-miR-320a | KCTD8 | - | - | - | + |
| hsa-miR-320a | KCTD9 | - | - | - | + |
| hsa-miR-320a | KDELR1 | - | - | + | - |
| hsa-miR-320a | KDELR2 | - | - | - | + |
| hsa-miR-320a | KDM5A | - | + | - | + |
| hsa-miR-320a | KDM5C | - | - | + | + |
| hsa-miR-320a | KDM5D | - | - | - | + |
| hsa-miR-320a | KDM6A | - | - | - | + |
| hsa-miR-320a | KDSR | - | + | - | + |
| hsa-miR-320a | KERA | - | - | - | + |
| hsa-miR-320a | KHDRBS1 | - | - | - | + |
| hsa-miR-320a | KHSRP | - | - | - | + |
| hsa-miR-320a | KIAA0020 | - | - | - | + |
| hsa-miR-320a | KIAA0087 | - | - | - | + |
| hsa-miR-320a | KIAA0100 | - | - | + | - |
| hsa-miR-320a | KIAA0141 | - | - | - | + |
| hsa-miR-320a | KIAA0182 | - | - | - | + |
| hsa-miR-320a | KIAA0226 | - | - | - | + |
| hsa-miR-320a | KIAA0232 | - | - | - | + |
| hsa-miR-320a | KIAA0319 | - | - | - | + |
| hsa-miR-320a | KIAA0355 | - | - | - | + |
| hsa-miR-320a | KIAA0368 | - | - | + | + |
| hsa-miR-320a | KIAA0430 | - | - | - | + |
| hsa-miR-320a | KIAA0513 | - | - | - | + |
| hsa-miR-320a | KIAA0754 | - | - | - | + |
| hsa-miR-320a | KIAA0895 | - | + | - | + |
| hsa-miR-320a | KIAA0947 | - | - | - | + |
| hsa-miR-320a | KIAA1024 | - | - | - | + |
| hsa-miR-320a | KIAA1045 | - | - | - | + |
| hsa-miR-320a | KIAA1109 | - | - | - | + |
| hsa-miR-320a | KIAA1147 | - | - | - | + |
| hsa-miR-320a | KIAA1161 | - | - | - | + |
| hsa-miR-320a | KIAA1199 | - | - | - | + |
| hsa-miR-320a | KIAA1210 | - | - | - | + |
| hsa-miR-320a | KIAA1217 | - | - | - | + |
| hsa-miR-320a | KIAA1244 | - | - | - | + |
| hsa-miR-320a | KIAA1324 | - | + | - | - |
| hsa-miR-320a | KIAA1324L | - | - | - | + |
| hsa-miR-320a | KIAA1429 | - | + | - | - |
| hsa-miR-320a | KIAA1430 | - | - | - | + |
| hsa-miR-320a | KIAA1432 | - | - | - | + |
| hsa-miR-320a | KIAA1468 | - | - | - | + |
| hsa-miR-320a | KIAA1549 | - | + | - | + |
| hsa-miR-320a | KIAA1551 | - | - | - | + |
| hsa-miR-320a | KIAA1598 | - | - | - | + |
| hsa-miR-320a | KIAA1632 | - | - | - | + |
| hsa-miR-320a | KIAA1715 | - | - | - | + |
| hsa-miR-320a | KIAA1804 | - | - | - | + |
| hsa-miR-320a | KIAA2013 | - | - | - | + |
| hsa-miR-320a | KIAA2018 | - | - | - | + |
| hsa-miR-320a | KIF13A | - | - | - | + |
| hsa-miR-320a | KIF13B | - | - | - | + |
| hsa-miR-320a | KIF14 | - | - | - | + |
| hsa-miR-320a | KIF16B | - | - | - | + |
| hsa-miR-320a | KIF17 | - | - | - | + |
| hsa-miR-320a | KIF18B | - | - | - | + |
| hsa-miR-320a | KIF1A | - | - | - | + |
| hsa-miR-320a | KIF1B | - | - | - | + |
| hsa-miR-320a | KIF1C | - | - | - | + |
| hsa-miR-320a | KIF26A | - | + | - | - |
| hsa-miR-320a | KIF26B | - | - | - | + |
| hsa-miR-320a | KIF2C | - | - | - | + |
| hsa-miR-320a | KIF3B | - | - | - | + |
| hsa-miR-320a | KIF5B | - | - | - | + |
| hsa-miR-320a | KIN | - | - | - | + |
| hsa-miR-320a | KIT | - | - | - | + |
| hsa-miR-320a | KITLG | + | + | - | - |
| hsa-miR-320a | KL | - | - | - | + |
| hsa-miR-320a | KLB | - | - | - | + |
| hsa-miR-320a | KLF10 | - | - | - | + |
| hsa-miR-320a | KLF12 | - | - | - | + |
| hsa-miR-320a | KLF13 | + | - | - | + |
| hsa-miR-320a | KLF3 | - | - | - | + |
| hsa-miR-320a | KLF5 | + | + | - | + |
| hsa-miR-320a | KLF7 | - | - | - | + |
| hsa-miR-320a | KLF8 | - | - | - | + |
| hsa-miR-320a | KLF9 | - | - | - | + |
| hsa-miR-320a | KLHDC10 | - | - | - | + |
| hsa-miR-320a | KLHDC9 | - | - | - | + |
| hsa-miR-320a | KLHL1 | - | - | - | + |
| hsa-miR-320a | KLHL14 | - | + | - | + |
| hsa-miR-320a | KLHL15 | - | - | + | + |
| hsa-miR-320a | KLHL20 | - | - | - | + |
| hsa-miR-320a | KLHL23 | - | + | - | + |
| hsa-miR-320a | KLHL25 | - | - | - | + |
| hsa-miR-320a | KLHL28 | - | - | - | + |
| hsa-miR-320a | KLHL3 | - | - | - | + |
| hsa-miR-320a | KLHL30 | - | - | - | + |
| hsa-miR-320a | KLHL36 | + | + | - | - |
| hsa-miR-320a | KLHL4 | - | - | - | + |
| hsa-miR-320a | KLHL42 | - | - | - | + |
| hsa-miR-320a | KLHL6 | - | - | - | + |
| hsa-miR-320a | KLLN | + | - | - | - |
| hsa-miR-320a | KMT2A | - | - | + | - |
| hsa-miR-320a | KMT2C | - | - | + | - |
| hsa-miR-320a | KMT2D | - | - | + | - |
| hsa-miR-320a | KPNA1 | - | - | - | + |
| hsa-miR-320a | KPNA3 | - | + | - | + |
| hsa-miR-320a | KPNA4 | - | - | - | + |
| hsa-miR-320a | KPNA6 | - | - | - | + |
| hsa-miR-320a | KPNA7 | - | - | - | + |
| hsa-miR-320a | KPNB1 | - | - | + | - |
| hsa-miR-320a | KRAS | - | - | - | + |
| hsa-miR-320a | KREMEN1 | - | - | - | + |
| hsa-miR-320a | KRI1 | - | - | - | + |
| hsa-miR-320a | KRIT1 | + | - | - | + |
| hsa-miR-320a | KRT12 | - | - | - | + |
| hsa-miR-320a | KRT27 | - | - | - | + |
| hsa-miR-320a | KRT5 | - | - | - | + |
| hsa-miR-320a | KRT71 | - | - | - | + |
| hsa-miR-320a | KRT78 | - | - | - | + |
| hsa-miR-320a | KRT80 | - | - | - | + |
| hsa-miR-320a | KRTAP11-1 | - | - | - | + |
| hsa-miR-320a | KRTAP12-1 | + | - | - | + |
| hsa-miR-320a | KRTAP12-2 | - | - | - | + |
| hsa-miR-320a | KRTAP17-1 | + | - | - | + |
| hsa-miR-320a | KRTAP19-3 | - | - | - | + |
| hsa-miR-320a | KRTAP2-3 | - | - | - | + |
| hsa-miR-320a | KRTAP24-1 | - | - | - | + |
| hsa-miR-320a | KRTAP3-3 | + | - | - | + |
| hsa-miR-320a | KRTAP4-1 | + | + | - | - |
| hsa-miR-320a | KRTAP9-3 | + | - | - | + |
| hsa-miR-320a | KRTAP9-8 | - | - | - | + |
| hsa-miR-320a | KRTAP9-9 | - | - | - | + |
| hsa-miR-320a | KSR1 | - | - | - | + |
| hsa-miR-320a | KSR2 | - | - | - | + |
| hsa-miR-320a | L1CAM | - | - | - | + |
| hsa-miR-320a | LACE1 | - | - | - | + |
| hsa-miR-320a | LACTB | - | - | - | + |
| hsa-miR-320a | LAIR1 | - | - | - | + |
| hsa-miR-320a | LAMA1 | - | - | - | + |
| hsa-miR-320a | LAMA5 | - | - | + | - |
| hsa-miR-320a | LAMB4 | - | - | - | + |
| hsa-miR-320a | LAMC1 | - | - | - | + |
| hsa-miR-320a | LAMP1 | - | - | - | + |
| hsa-miR-320a | LAMP2 | + | - | - | + |
| hsa-miR-320a | LAMP3 | - | - | - | + |
| hsa-miR-320a | LAMP5 | - | - | - | + |
| hsa-miR-320a | LANCL1 | - | - | - | + |
| hsa-miR-320a | LAPTM4A | + | - | - | + |
| hsa-miR-320a | LARP1 | - | - | - | + |
| hsa-miR-320a | LARP1B | - | - | - | + |
| hsa-miR-320a | LARP4 | - | - | - | + |
| hsa-miR-320a | LARS | - | + | - | + |
| hsa-miR-320a | LATS1 | - | - | - | + |
| hsa-miR-320a | LATS2 | - | - | - | + |
| hsa-miR-320a | LBH | - | + | - | + |
| hsa-miR-320a | LBR | - | - | - | + |
| hsa-miR-320a | LCA10 | + | - | - | - |
| hsa-miR-320a | LCA5 | - | - | - | + |
| hsa-miR-320a | LCE5A | - | - | - | + |
| hsa-miR-320a | LCP1 | - | - | - | + |
| hsa-miR-320a | LDB2 | - | - | - | + |
| hsa-miR-320a | LDB3 | - | - | - | + |
| hsa-miR-320a | LDHAL6A | - | - | - | + |
| hsa-miR-320a | LDLR | - | - | - | + |
| hsa-miR-320a | LDLRAD3 | - | - | - | + |
| hsa-miR-320a | LDLRAD4 | - | - | - | + |
| hsa-miR-320a | LDLRAP1 | - | - | - | + |
| hsa-miR-320a | LDOC1L | - | - | - | + |
| hsa-miR-320a | LEF1 | + | - | - | + |
| hsa-miR-320a | LEMD3 | - | - | - | + |
| hsa-miR-320a | LEO1 | - | - | - | + |
| hsa-miR-320a | LEPR | + | - | - | - |
| hsa-miR-320a | LEPREL1 | - | - | - | + |
| hsa-miR-320a | LEPROTL1 | - | - | - | + |
| hsa-miR-320a | LETM1 | - | - | - | + |
| hsa-miR-320a | LGI2 | - | - | - | + |
| hsa-miR-320a | LGR5 | - | - | - | + |
| hsa-miR-320a | LGSN | - | - | - | + |
| hsa-miR-320a | LHFPL3 | + | + | - | - |
| hsa-miR-320a | LHX6 | - | - | - | + |
| hsa-miR-320a | LIAS | - | - | - | + |
| hsa-miR-320a | LIFR | - | - | - | + |
| hsa-miR-320a | LILRA4 | - | - | - | + |
| hsa-miR-320a | LILRB4 | - | - | - | + |
| hsa-miR-320a | LIMCH1 | - | - | - | + |
| hsa-miR-320a | LIN28A | - | - | - | + |
| hsa-miR-320a | LIN28B | - | - | - | + |
| hsa-miR-320a | LIN7C | - | - | - | + |
| hsa-miR-320a | LINC00636 | - | - | - | + |
| hsa-miR-320a | LINGO2 | - | - | - | + |
| hsa-miR-320a | LIPH | - | - | - | + |
| hsa-miR-320a | LIPJ | - | - | - | + |
| hsa-miR-320a | LITAF | - | - | + | - |
| hsa-miR-320a | LIX1 | - | - | - | + |
| hsa-miR-320a | LMAN1 | - | - | - | + |
| hsa-miR-320a | LMAN1L | - | - | - | + |
| hsa-miR-320a | LMBRD2 | - | - | - | + |
| hsa-miR-320a | LMCD1 | - | + | - | - |
| hsa-miR-320a | LMLN | - | - | - | + |
| hsa-miR-320a | LMNA | - | - | - | + |
| hsa-miR-320a | LMNB1 | - | - | + | - |
| hsa-miR-320a | LMO3 | + | + | - | - |
| hsa-miR-320a | LMO4 | - | - | - | + |
| hsa-miR-320a | LMO7 | - | - | - | + |
| hsa-miR-320a | LMTK2 | - | - | - | + |
| hsa-miR-320a | LOC100130522 | - | - | - | + |
| hsa-miR-320a | LONP2 | - | - | + | - |
| hsa-miR-320a | LONRF2 | - | - | - | + |
| hsa-miR-320a | LOX | - | - | - | + |
| hsa-miR-320a | LOXL3 | - | - | - | + |
| hsa-miR-320a | LPAR5 | - | - | - | + |
| hsa-miR-320a | LPCAT2 | - | - | - | + |
| hsa-miR-320a | LPGAT1 | - | - | - | + |
| hsa-miR-320a | LPHN2 | - | - | - | + |
| hsa-miR-320a | LPHN3 | - | - | - | + |
| hsa-miR-320a | LPIN2 | - | - | - | + |
| hsa-miR-320a | LPIN3 | - | - | - | + |
| hsa-miR-320a | LPL | - | - | - | + |
| hsa-miR-320a | LPPR5 | - | - | - | + |
| hsa-miR-320a | LRCH2 | - | - | - | + |
| hsa-miR-320a | LRCH3 | - | - | + | - |
| hsa-miR-320a | LRGUK | - | - | - | + |
| hsa-miR-320a | LRIF1 | - | - | - | + |
| hsa-miR-320a | LRIT1 | - | - | - | + |
| hsa-miR-320a | LRP10 | - | - | - | + |
| hsa-miR-320a | LRP11 | - | - | - | + |
| hsa-miR-320a | LRP12 | - | - | - | + |
| hsa-miR-320a | LRP1B | - | - | - | + |
| hsa-miR-320a | LRP2 | - | - | - | + |
| hsa-miR-320a | LRP4 | - | - | - | + |
| hsa-miR-320a | LRP6 | - | - | - | + |
| hsa-miR-320a | LRRC1 | - | - | - | + |
| hsa-miR-320a | LRRC16A | - | - | - | + |
| hsa-miR-320a | LRRC17 | - | - | - | + |
| hsa-miR-320a | LRRC19 | - | - | - | + |
| hsa-miR-320a | LRRC31 | - | - | - | + |
| hsa-miR-320a | LRRC33 | - | - | - | + |
| hsa-miR-320a | LRRC38 | - | - | - | + |
| hsa-miR-320a | LRRC4 | - | - | - | + |
| hsa-miR-320a | LRRC40 | - | - | - | + |
| hsa-miR-320a | LRRC66 | + | - | - | + |
| hsa-miR-320a | LRRC8B | - | - | - | + |
| hsa-miR-320a | LRRC8C | - | - | - | + |
| hsa-miR-320a | LRRC8D | - | - | - | + |
| hsa-miR-320a | LRRC8E | - | - | - | + |
| hsa-miR-320a | LRRC9 | - | - | - | + |
| hsa-miR-320a | LRRN1 | - | - | - | + |
| hsa-miR-320a | LRRTM4 | - | - | - | + |
| hsa-miR-320a | LSM11 | - | - | - | + |
| hsa-miR-320a | LSM12 | - | - | + | + |
| hsa-miR-320a | LSM5 | - | - | - | + |
| hsa-miR-320a | LTB4R | - | - | - | + |
| hsa-miR-320a | LTBP2 | - | - | - | + |
| hsa-miR-320a | LTK | - | - | - | + |
| hsa-miR-320a | LUZP1 | - | - | - | + |
| hsa-miR-320a | LUZP2 | - | - | - | + |
| hsa-miR-320a | LYG2 | + | - | - | - |
| hsa-miR-320a | LYPD3 | - | - | - | + |
| hsa-miR-320a | LYPD4 | - | - | - | + |
| hsa-miR-320a | LYPD6 | - | - | - | + |
| hsa-miR-320a | LYPD6B | - | - | - | + |
| hsa-miR-320a | LYRM7 | - | - | - | + |
| hsa-miR-320a | LYSMD2 | - | - | - | + |
| hsa-miR-320a | LYZL4 | - | - | - | + |
| hsa-miR-320a | LZTFL1 | - | - | - | + |
| hsa-miR-320a | LZTS1 | - | - | - | + |
| hsa-miR-320a | MACROD2 | - | - | - | + |
| hsa-miR-320a | MAEL | - | - | - | + |
| hsa-miR-320a | MAF | - | - | - | + |
| hsa-miR-320a | MAFB | - | - | - | + |
| hsa-miR-320a | MAFG | - | - | - | + |
| hsa-miR-320a | MAFK | - | - | - | + |
| hsa-miR-320a | MAGEA8 | - | - | - | + |
| hsa-miR-320a | MAGEB1 | - | - | - | + |
| hsa-miR-320a | MAGEC1 | - | - | - | + |
| hsa-miR-320a | MAGEC2 | - | - | - | + |
| hsa-miR-320a | MAGED4 | - | - | - | + |
| hsa-miR-320a | MAGEL2 | - | - | - | + |
| hsa-miR-320a | MAGI2 | - | - | - | + |
| hsa-miR-320a | MAK | - | - | - | + |
| hsa-miR-320a | MAK16 | - | - | + | + |
| hsa-miR-320a | MAMDC2 | - | - | - | + |
| hsa-miR-320a | MAML1 | - | + | - | + |
| hsa-miR-320a | MAN1A1 | - | - | - | + |
| hsa-miR-320a | MAN1C1 | - | - | - | + |
| hsa-miR-320a | MAN2A1 | - | - | + | - |
| hsa-miR-320a | MANBA | + | + | - | - |
| hsa-miR-320a | MANEA | - | - | - | + |
| hsa-miR-320a | MAP1B | - | + | - | + |
| hsa-miR-320a | MAP1LC3B2 | + | - | - | - |
| hsa-miR-320a | MAP2 | - | - | - | + |
| hsa-miR-320a | MAP2K4 | - | - | - | + |
| hsa-miR-320a | MAP3K13 | - | - | - | + |
| hsa-miR-320a | MAP3K2 | - | - | - | + |
| hsa-miR-320a | MAP3K7 | - | - | - | + |
| hsa-miR-320a | MAP3K9 | - | - | - | + |
| hsa-miR-320a | MAP4K3 | - | - | - | + |
| hsa-miR-320a | MAP6 | - | - | - | + |
| hsa-miR-320a | MAP7 | - | + | - | + |
| hsa-miR-320a | MAP7D2 | - | - | - | + |
| hsa-miR-320a | MAP9 | - | - | - | + |
| hsa-miR-320a | MAPK1 | - | - | + | - |
| hsa-miR-320a | MAPK14 | - | - | - | + |
| hsa-miR-320a | MAPK1IP1L | - | + | + | + |
| hsa-miR-320a | MAPK8 | - | - | - | + |
| hsa-miR-320a | MAPK8IP3 | - | + | + | + |
| hsa-miR-320a | MAPK9 | - | + | - | + |
| hsa-miR-320a | MAPRE1 | - | - | - | + |
| hsa-miR-320a | MAPRE2 | - | - | - | + |
| hsa-miR-320a | MAPRE3 | - | - | - | + |
| hsa-miR-320a | MAPT | - | - | - | + |
| hsa-miR-320a | 4-Mar | - | - | + | - |
| hsa-miR-320a | 5-Mar | - | + | - | + |
| hsa-miR-320a | 6-Mar | - | - | - | + |
| hsa-miR-320a | 9-Mar | - | - | - | + |
| hsa-miR-320a | MARK1 | - | + | - | + |
| hsa-miR-320a | MARK2 | - | - | + | - |
| hsa-miR-320a | MARVELD1 | - | - | - | + |
| hsa-miR-320a | MARVELD3 | - | - | - | + |
| hsa-miR-320a | MAST3 | - | - | - | + |
| hsa-miR-320a | MAST4 | - | + | - | - |
| hsa-miR-320a | MASTL | - | + | - | + |
| hsa-miR-320a | MAT1A | - | + | - | - |
| hsa-miR-320a | MAT2A | - | - | - | + |
| hsa-miR-320a | MAVS | - | - | - | + |
| hsa-miR-320a | MAX | - | - | + | - |
| hsa-miR-320a | MB21D2 | - | - | - | + |
| hsa-miR-320a | MBD1 | - | - | - | + |
| hsa-miR-320a | MBD2 | - | - | - | + |
| hsa-miR-320a | MBIP | + | - | - | - |
| hsa-miR-320a | MBL2 | + | - | - | + |
| hsa-miR-320a | MBLAC2 | - | - | - | + |
| hsa-miR-320a | MBNL2 | - | - | - | + |
| hsa-miR-320a | MBNL3 | - | - | - | + |
| hsa-miR-320a | MBTD1 | - | - | - | + |
| hsa-miR-320a | MC2R | - | - | - | + |
| hsa-miR-320a | MCC | - | - | - | + |
| hsa-miR-320a | MCF2L | - | - | - | + |
| hsa-miR-320a | MCFD2 | - | - | - | + |
| hsa-miR-320a | MCL1 | - | + | + | + |
| hsa-miR-320a | MCM3AP | - | - | - | + |
| hsa-miR-320a | MCM4 | - | - | + | + |
| hsa-miR-320a | MCM6 | - | - | - | + |
| hsa-miR-320a | MCMBP | - | - | - | + |
| hsa-miR-320a | MCMDC2 | - | + | - | + |
| hsa-miR-320a | MCOLN2 | + | - | - | - |
| hsa-miR-320a | MCRS1 | - | - | + | - |
| hsa-miR-320a | MCTP1 | - | - | - | + |
| hsa-miR-320a | MDC1 | - | - | - | + |
| hsa-miR-320a | MDGA2 | - | + | - | + |
| hsa-miR-320a | MDH2 | - | - | + | + |
| hsa-miR-320a | MDK | + | - | - | - |
| hsa-miR-320a | MDM1 | - | - | - | + |
| hsa-miR-320a | MDM2 | - | - | - | + |
| hsa-miR-320a | MDM4 | - | - | - | + |
| hsa-miR-320a | MDN1 | - | - | - | + |
| hsa-miR-320a | MECOM | - | - | - | + |
| hsa-miR-320a | MECP2 | - | - | - | + |
| hsa-miR-320a | MED1 | - | + | - | - |
| hsa-miR-320a | MED12 | - | - | + | - |
| hsa-miR-320a | MED12L | - | - | - | + |
| hsa-miR-320a | MED13 | - | - | - | + |
| hsa-miR-320a | MED13L | - | - | - | + |
| hsa-miR-320a | MED14 | - | - | - | + |
| hsa-miR-320a | MED19 | - | - | - | + |
| hsa-miR-320a | MED21 | + | - | - | - |
| hsa-miR-320a | MED23 | - | - | + | + |
| hsa-miR-320a | MED24 | - | - | + | - |
| hsa-miR-320a | MED26 | - | - | + | - |
| hsa-miR-320a | MED6 | - | - | - | + |
| hsa-miR-320a | MED7 | + | + | - | - |
| hsa-miR-320a | MED8 | - | - | - | + |
| hsa-miR-320a | MEF2A | - | - | - | + |
| hsa-miR-320a | MEF2C | - | - | - | + |
| hsa-miR-320a | MEF2D | - | - | + | + |
| hsa-miR-320a | MEGF10 | - | - | - | + |
| hsa-miR-320a | MEGF9 | - | - | - | + |
| hsa-miR-320a | MEOX2 | - | - | + | + |
| hsa-miR-320a | MEPCE | - | - | - | + |
| hsa-miR-320a | MESDC2 | - | - | - | + |
| hsa-miR-320a | MET | - | - | + | - |
| hsa-miR-320a | METAP1D | - | - | - | + |
| hsa-miR-320a | METAP2 | - | - | - | + |
| hsa-miR-320a | METTL13 | - | + | - | - |
| hsa-miR-320a | METTL15 | - | - | - | + |
| hsa-miR-320a | METTL17 | - | - | + | - |
| hsa-miR-320a | METTL21A | - | - | - | + |
| hsa-miR-320a | METTL21C | - | - | - | + |
| hsa-miR-320a | METTL6 | - | - | - | + |
| hsa-miR-320a | METTL7A | + | + | + | + |
| hsa-miR-320a | METTL9 | - | - | - | + |
| hsa-miR-320a | MEX3B | - | + | - | - |
| hsa-miR-320a | MEX3D | - | - | - | + |
| hsa-miR-320a | MFAP3L | - | - | - | + |
| hsa-miR-320a | MFAP5 | + | - | - | + |
| hsa-miR-320a | MFHAS1 | - | - | - | + |
| hsa-miR-320a | MFN2 | - | - | - | + |
| hsa-miR-320a | MFSD6 | - | - | - | + |
| hsa-miR-320a | MFSD6L | - | - | - | + |
| hsa-miR-320a | MFSD8 | - | - | - | + |
| hsa-miR-320a | MGA | - | - | - | + |
| hsa-miR-320a | MGARP | - | - | - | + |
| hsa-miR-320a | MGAT4A | - | - | - | + |
| hsa-miR-320a | MGAT4B | - | - | - | + |
| hsa-miR-320a | MGAT5 | - | - | - | + |
| hsa-miR-320a | MGEA5 | - | - | - | + |
| hsa-miR-320a | MGRN1 | - | - | - | + |
| hsa-miR-320a | MGST2 | + | - | - | - |
| hsa-miR-320a | MIB1 | - | - | - | + |
| hsa-miR-320a | MICALCL | - | - | - | + |
| hsa-miR-320a | MICB | - | - | - | + |
| hsa-miR-320a | MICU3 | - | - | - | + |
| hsa-miR-320a | MID1 | - | - | - | + |
| hsa-miR-320a | MIER1 | - | + | - | + |
| hsa-miR-320a | MIER3 | - | + | - | - |
| hsa-miR-320a | MIF | - | - | + | - |
| hsa-miR-320a | MIF4GD | - | - | - | + |
| hsa-miR-320a | MINOS1 | - | - | - | + |
| hsa-miR-320a | MINPP1 | - | - | - | + |
| hsa-miR-320a | MIOS | - | - | - | + |
| hsa-miR-320a | MIP | - | - | - | + |
| hsa-miR-320a | MIS18BP1 | - | - | - | + |
| hsa-miR-320a | MITF | - | - | - | + |
| hsa-miR-320a | MKI67 | - | - | - | + |
| hsa-miR-320a | MKL2 | - | - | - | + |
| hsa-miR-320a | MKLN1 | + | - | - | + |
| hsa-miR-320a | MKX | - | - | - | + |
| hsa-miR-320a | MLEC | - | - | - | + |
| hsa-miR-320a | MLF1 | - | + | - | + |
| hsa-miR-320a | MLH3 | - | - | - | + |
| hsa-miR-320a | MLIP | + | - | - | - |
| hsa-miR-320a | MLK4 | + | - | - | - |
| hsa-miR-320a | MLL | - | - | - | + |
| hsa-miR-320a | MLLT10 | - | - | - | + |
| hsa-miR-320a | MLLT3 | - | + | - | + |
| hsa-miR-320a | MLLT4 | - | - | - | + |
| hsa-miR-320a | MLX | - | - | + | - |
| hsa-miR-320a | MMAA | - | - | - | + |
| hsa-miR-320a | MMAB | - | - | - | + |
| hsa-miR-320a | MME | - | - | - | + |
| hsa-miR-320a | MMP16 | - | + | - | + |
| hsa-miR-320a | MMS22L | - | + | + | + |
| hsa-miR-320a | MN1 | - | - | - | + |
| hsa-miR-320a | MNF1 | - | - | + | - |
| hsa-miR-320a | MOB1B | - | + | - | + |
| hsa-miR-320a | MOB3B | - | - | - | + |
| hsa-miR-320a | MOB3C | - | - | - | + |
| hsa-miR-320a | MOB4 | - | - | - | + |
| hsa-miR-320a | MOBP | - | - | - | + |
| hsa-miR-320a | MOCS1 | - | - | + | - |
| hsa-miR-320a | MOCS2 | - | - | - | + |
| hsa-miR-320a | MORC3 | - | - | - | + |
| hsa-miR-320a | MORF4L1 | - | - | - | + |
| hsa-miR-320a | MOV10 | - | - | - | + |
| hsa-miR-320a | MPC2 | - | - | - | + |
| hsa-miR-320a | MPI | - | - | + | - |
| hsa-miR-320a | MPP6 | - | - | - | + |
| hsa-miR-320a | MPPED2 | + | + | - | + |
| hsa-miR-320a | MPZ | - | - | - | + |
| hsa-miR-320a | MPZL1 | - | - | - | + |
| hsa-miR-320a | MPZL2 | - | - | - | + |
| hsa-miR-320a | MPZL3 | - | - | - | + |
| hsa-miR-320a | MRAP2 | - | + | - | + |
| hsa-miR-320a | MRAS | - | - | - | + |
| hsa-miR-320a | MRFAP1 | - | + | - | - |
| hsa-miR-320a | MRFAP1L1 | - | - | - | + |
| hsa-miR-320a | MRO | - | + | - | - |
| hsa-miR-320a | MRP63 | - | - | - | + |
| hsa-miR-320a | MRPL14 | - | - | - | + |
| hsa-miR-320a | MRPL19 | - | + | - | - |
| hsa-miR-320a | MRPL35 | + | + | - | + |
| hsa-miR-320a | MRPL49 | - | - | - | + |
| hsa-miR-320a | MRPL9 | - | - | + | - |
| hsa-miR-320a | MRPS10 | - | - | - | + |
| hsa-miR-320a | MRPS16 | - | - | - | + |
| hsa-miR-320a | MRPS18B | + | + | + | - |
| hsa-miR-320a | MRPS25 | - | - | - | + |
| hsa-miR-320a | MRPS35 | - | - | - | + |
| hsa-miR-320a | MRPS5 | - | - | + | - |
| hsa-miR-320a | MRS2 | - | - | - | + |
| hsa-miR-320a | MRTO4 | - | - | - | + |
| hsa-miR-320a | MS4A7 | - | - | - | + |
| hsa-miR-320a | MSANTD4 | - | - | - | + |
| hsa-miR-320a | MSH4 | - | - | - | + |
| hsa-miR-320a | MSI2 | + | + | - | + |
| hsa-miR-320a | MSL2 | - | - | - | + |
| hsa-miR-320a | MSL3 | - | - | - | + |
| hsa-miR-320a | MSMO1 | - | - | - | + |
| hsa-miR-320a | MSRB3 | + | - | - | + |
| hsa-miR-320a | MST4 | + | - | - | - |
| hsa-miR-320a | MT2A | + | - | - | - |
| hsa-miR-320a | MTA3 | - | - | - | + |
| hsa-miR-320a | MTAP | - | - | - | + |
| hsa-miR-320a | MTCH2 | - | - | - | + |
| hsa-miR-320a | MTDH | - | - | - | + |
| hsa-miR-320a | MTERFD1 | - | - | - | + |
| hsa-miR-320a | MTERFD3 | - | - | - | + |
| hsa-miR-320a | MTF1 | - | + | - | - |
| hsa-miR-320a | MTF2 | - | - | - | + |
| hsa-miR-320a | MTFMT | + | - | - | - |
| hsa-miR-320a | MTHFSD | - | - | - | + |
| hsa-miR-320a | MTM1 | - | - | - | + |
| hsa-miR-320a | MTMR1 | - | - | - | + |
| hsa-miR-320a | MTMR11 | - | + | - | - |
| hsa-miR-320a | MTMR12 | - | - | - | + |
| hsa-miR-320a | MTMR2 | - | - | - | + |
| hsa-miR-320a | MTMR3 | - | - | - | + |
| hsa-miR-320a | MTMR4 | - | - | - | + |
| hsa-miR-320a | MTMR6 | - | + | - | + |
| hsa-miR-320a | MTMR7 | - | - | - | + |
| hsa-miR-320a | MTMR9 | - | - | - | + |
| hsa-miR-320a | MTPN | - | - | - | + |
| hsa-miR-320a | MTRF1 | + | + | - | - |
| hsa-miR-320a | MTRF1L | - | - | - | + |
| hsa-miR-320a | MTRNR2L1 | + | - | - | + |
| hsa-miR-320a | MTRNR2L13 | + | - | - | - |
| hsa-miR-320a | MTRNR2L3 | - | - | - | + |
| hsa-miR-320a | MTRNR2L6 | - | - | + | - |
| hsa-miR-320a | MTRNR2L7 | - | - | - | + |
| hsa-miR-320a | MTRNR2L8 | + | - | - | + |
| hsa-miR-320a | MTRR | - | + | - | - |
| hsa-miR-320a | MTURN | - | - | - | + |
| hsa-miR-320a | MUC13 | - | + | - | - |
| hsa-miR-320a | MUL1 | - | - | - | + |
| hsa-miR-320a | MUM1L1 | - | - | - | + |
| hsa-miR-320a | MURC | + | - | - | + |
| hsa-miR-320a | MXD1 | - | - | - | + |
| hsa-miR-320a | MXI1 | - | + | - | + |
| hsa-miR-320a | MYBL1 | - | - | - | + |
| hsa-miR-320a | MYC | - | - | + | - |
| hsa-miR-320a | MYCN | - | - | - | + |
| hsa-miR-320a | MYEF2 | - | + | - | - |
| hsa-miR-320a | MYF6 | - | - | - | + |
| hsa-miR-320a | MYH10 | - | - | - | + |
| hsa-miR-320a | MYH15 | - | - | - | + |
| hsa-miR-320a | MYL12A | - | - | - | + |
| hsa-miR-320a | MYL12B | + | - | - | + |
| hsa-miR-320a | MYLK3 | - | - | - | + |
| hsa-miR-320a | MYNN | - | - | - | + |
| hsa-miR-320a | MYO10 | - | - | - | + |
| hsa-miR-320a | MYO1B | - | - | - | + |
| hsa-miR-320a | MYO1D | - | - | + | - |
| hsa-miR-320a | MYO5A | - | - | - | + |
| hsa-miR-320a | MYO5B | - | - | - | + |
| hsa-miR-320a | MYO5C | - | - | + | - |
| hsa-miR-320a | MYO6 | - | - | + | + |
| hsa-miR-320a | MYO9A | - | - | - | + |
| hsa-miR-320a | MYOC | - | - | - | + |
| hsa-miR-320a | MYOCD | - | - | - | + |
| hsa-miR-320a | MYOD1 | - | - | - | + |
| hsa-miR-320a | MYOM2 | - | - | - | + |
| hsa-miR-320a | MYOZ2 | - | - | - | + |
| hsa-miR-320a | MYOZ3 | - | - | - | + |
| hsa-miR-320a | MYRF | + | - | - | - |
| hsa-miR-320a | MYSM1 | - | - | - | + |
| hsa-miR-320a | MYT1L | - | - | - | + |
| hsa-miR-320a | MZT1 | + | - | - | + |
| hsa-miR-320a | N4BP1 | - | + | - | + |
| hsa-miR-320a | N4BP2L1 | - | - | - | + |
| hsa-miR-320a | NAA15 | - | + | - | + |
| hsa-miR-320a | NAA16 | - | - | - | + |
| hsa-miR-320a | NAA20 | + | - | - | + |
| hsa-miR-320a | NAA25 | - | - | - | + |
| hsa-miR-320a | NAA40 | - | - | - | + |
| hsa-miR-320a | NAA50 | - | - | - | + |
| hsa-miR-320a | NAALAD2 | - | - | - | + |
| hsa-miR-320a | NAALADL2 | - | - | - | + |
| hsa-miR-320a | NAB1 | - | - | - | + |
| hsa-miR-320a | NACC2 | - | - | - | + |
| hsa-miR-320a | NADK | - | - | - | + |
| hsa-miR-320a | NADKD1 | - | - | - | + |
| hsa-miR-320a | NAGA | - | - | - | + |
| hsa-miR-320a | NALCN | - | - | - | + |
| hsa-miR-320a | NAMPT | - | - | - | + |
| hsa-miR-320a | NANOG | - | - | + | - |
| hsa-miR-320a | NANOS1 | - | - | - | + |
| hsa-miR-320a | NANOS2 | - | - | - | + |
| hsa-miR-320a | NANP | - | - | - | + |
| hsa-miR-320a | NAP1L1 | - | - | - | + |
| hsa-miR-320a | NAP1L5 | - | + | - | + |
| hsa-miR-320a | NAP1L6 | - | - | - | + |
| hsa-miR-320a | NAPB | - | - | - | + |
| hsa-miR-320a | NAPEPLD | - | - | - | + |
| hsa-miR-320a | NAPG | - | - | - | + |
| hsa-miR-320a | NAT1 | + | - | - | + |
| hsa-miR-320a | NAT10 | - | - | - | + |
| hsa-miR-320a | NAT6 | + | - | - | + |
| hsa-miR-320a | NAT9 | - | - | - | + |
| hsa-miR-320a | NBEA | - | - | - | + |
| hsa-miR-320a | NBEAL1 | - | - | - | + |
| hsa-miR-320a | NBPF4 | + | - | - | - |
| hsa-miR-320a | NBPF6 | + | - | - | - |
| hsa-miR-320a | NCAM1 | - | - | - | + |
| hsa-miR-320a | NCAN | - | - | - | + |
| hsa-miR-320a | NCAPD3 | - | + | + | - |
| hsa-miR-320a | NCBP1 | - | - | - | + |
| hsa-miR-320a | NCDN | - | - | - | + |
| hsa-miR-320a | NCK2 | - | - | - | + |
| hsa-miR-320a | NCKAP1 | - | - | - | + |
| hsa-miR-320a | NCOA1 | - | - | - | + |
| hsa-miR-320a | NCOR1 | - | + | + | - |
| hsa-miR-320a | NCR3 | - | - | - | + |
| hsa-miR-320a | NCR3LG1 | - | - | - | + |
| hsa-miR-320a | NCS1 | - | - | - | + |
| hsa-miR-320a | ND1 | - | - | + | - |
| hsa-miR-320a | ND4 | - | - | + | - |
| hsa-miR-320a | ND5 | - | - | + | - |
| hsa-miR-320a | NDEL1 | - | - | - | + |
| hsa-miR-320a | NDN | - | - | - | + |
| hsa-miR-320a | NDP | - | - | - | + |
| hsa-miR-320a | NDRG3 | - | - | - | + |
| hsa-miR-320a | NDST1 | - | - | - | + |
| hsa-miR-320a | NDUFA10 | - | - | - | + |
| hsa-miR-320a | NDUFA3 | - | - | + | - |
| hsa-miR-320a | NDUFA5 | - | - | - | + |
| hsa-miR-320a | NEBL | - | - | - | + |
| hsa-miR-320a | NECAB1 | - | - | - | + |
| hsa-miR-320a | NECAP2 | - | - | - | + |
| hsa-miR-320a | NEDD1 | - | - | - | + |
| hsa-miR-320a | NEGR1 | - | + | - | - |
| hsa-miR-320a | NEK2 | - | - | - | + |
| hsa-miR-320a | NEK7 | - | - | - | + |
| hsa-miR-320a | NELL2 | - | - | - | + |
| hsa-miR-320a | NEO1 | - | - | + | + |
| hsa-miR-320a | NET1 | - | - | + | + |
| hsa-miR-320a | NETO1 | - | + | - | - |
| hsa-miR-320a | NEURL1B | - | - | - | + |
| hsa-miR-320a | NEUROG1 | - | - | - | + |
| hsa-miR-320a | NEXN | - | - | - | + |
| hsa-miR-320a | NFATC2 | - | + | - | + |
| hsa-miR-320a | NFATC2IP | - | - | - | + |
| hsa-miR-320a | NFATC3 | - | - | + | + |
| hsa-miR-320a | NFE2L3 | - | - | - | + |
| hsa-miR-320a | NFIA | - | - | - | + |
| hsa-miR-320a | NFIB | - | - | - | + |
| hsa-miR-320a | NFIC | - | - | - | + |
| hsa-miR-320a | NFKB1 | - | - | - | + |
| hsa-miR-320a | NFKBIZ | - | - | - | + |
| hsa-miR-320a | NFYB | - | - | - | + |
| hsa-miR-320a | NGB | - | - | - | + |
| hsa-miR-320a | NHP2L1 | - | - | - | + |
| hsa-miR-320a | NHS | - | - | - | + |
| hsa-miR-320a | NHSL1 | - | - | - | + |
| hsa-miR-320a | NIN | + | + | - | - |
| hsa-miR-320a | NIP7 | - | + | - | + |
| hsa-miR-320a | NIPA1 | - | - | - | + |
| hsa-miR-320a | NIPA2 | - | - | - | + |
| hsa-miR-320a | NIPAL3 | - | - | - | + |
| hsa-miR-320a | NIPBL | - | - | - | + |
| hsa-miR-320a | NIT2 | - | - | + | - |
| hsa-miR-320a | NKAIN1 | - | - | - | + |
| hsa-miR-320a | NKAIN2 | - | - | - | + |
| hsa-miR-320a | NKAIN4 | - | - | - | + |
| hsa-miR-320a | NKIRAS2 | - | - | - | + |
| hsa-miR-320a | NKRF | - | - | - | + |
| hsa-miR-320a | NKX2-1 | - | - | - | + |
| hsa-miR-320a | NKX2-2 | - | - | - | + |
| hsa-miR-320a | NKX3-1 | + | - | - | + |
| hsa-miR-320a | NLGN1 | - | - | - | + |
| hsa-miR-320a | NLGN3 | - | - | - | + |
| hsa-miR-320a | NLGN4X | - | - | - | + |
| hsa-miR-320a | NLK | - | - | - | + |
| hsa-miR-320a | NLN | - | - | - | + |
| hsa-miR-320a | NLRP8 | - | - | - | + |
| hsa-miR-320a | NMD3 | - | - | - | + |
| hsa-miR-320a | NMI | - | - | - | + |
| hsa-miR-320a | NMNAT1 | - | - | - | + |
| hsa-miR-320a | NMNAT2 | - | - | - | + |
| hsa-miR-320a | NOC3L | - | - | - | + |
| hsa-miR-320a | NOC4L | - | - | + | - |
| hsa-miR-320a | NOL4 | - | - | - | + |
| hsa-miR-320a | NOL9 | - | - | - | + |
| hsa-miR-320a | NOLC1 | - | - | - | + |
| hsa-miR-320a | NOM1 | - | - | - | + |
| hsa-miR-320a | NONO | - | - | - | + |
| hsa-miR-320a | NOP14 | - | - | - | + |
| hsa-miR-320a | NOS1 | - | - | - | + |
| hsa-miR-320a | NOS1AP | - | - | + | + |
| hsa-miR-320a | NOX4 | - | - | - | + |
| hsa-miR-320a | NPAP1 | - | - | - | + |
| hsa-miR-320a | NPAS2 | + | + | - | + |
| hsa-miR-320a | NPAS3 | - | - | - | + |
| hsa-miR-320a | NPAT | - | - | - | + |
| hsa-miR-320a | NPC1L1 | - | - | - | + |
| hsa-miR-320a | NPEPPS | - | - | + | - |
| hsa-miR-320a | NPHS2 | - | - | - | + |
| hsa-miR-320a | NPLOC4 | - | - | + | - |
| hsa-miR-320a | NPM1 | - | - | + | - |
| hsa-miR-320a | NPM3 | - | - | + | - |
| hsa-miR-320a | NPNT | - | - | - | + |
| hsa-miR-320a | NPPB | - | - | - | + |
| hsa-miR-320a | NPR1 | - | - | + | - |
| hsa-miR-320a | NPR3 | - | - | - | + |
| hsa-miR-320a | NPTX1 | - | - | - | + |
| hsa-miR-320a | NPTX2 | - | - | - | + |
| hsa-miR-320a | NPY1R | - | - | - | + |
| hsa-miR-320a | NQO1 | - | - | - | + |
| hsa-miR-320a | NQO2 | - | - | + | - |
| hsa-miR-320a | NR1H2 | - | - | - | + |
| hsa-miR-320a | NR2C2 | - | + | - | - |
| hsa-miR-320a | NR2E1 | - | - | - | + |
| hsa-miR-320a | NR2E3 | - | + | - | - |
| hsa-miR-320a | NR2F2 | - | - | - | + |
| hsa-miR-320a | NR3C1 | - | + | - | + |
| hsa-miR-320a | NR3C2 | - | + | - | - |
| hsa-miR-320a | NR4A2 | - | - | - | + |
| hsa-miR-320a | NR6A1 | - | - | - | + |
| hsa-miR-320a | NRBP1 | - | - | + | - |
| hsa-miR-320a | NRCAM | - | - | - | + |
| hsa-miR-320a | NRF1 | - | - | + | - |
| hsa-miR-320a | NRIP2 | - | - | - | + |
| hsa-miR-320a | NRIP3 | - | - | - | + |
| hsa-miR-320a | NRK | - | - | - | + |
| hsa-miR-320a | NRL | - | - | - | + |
| hsa-miR-320a | NRN1 | + | - | - | - |
| hsa-miR-320a | NRP1 | - | + | + | + |
| hsa-miR-320a | NRP2 | - | - | - | + |
| hsa-miR-320a | NRXN3 | - | - | - | + |
| hsa-miR-320a | NSD1 | - | - | - | + |
| hsa-miR-320a | NSFL1C | - | - | + | - |
| hsa-miR-320a | NSMCE1 | + | - | - | - |
| hsa-miR-320a | NT5C | - | - | + | - |
| hsa-miR-320a | NT5C2 | - | - | - | + |
| hsa-miR-320a | NT5C3A | - | - | + | - |
| hsa-miR-320a | NT5DC3 | - | - | - | + |
| hsa-miR-320a | NTF3 | - | - | - | + |
| hsa-miR-320a | NTN4 | - | - | - | + |
| hsa-miR-320a | NTRK2 | - | - | - | + |
| hsa-miR-320a | NUCKS1 | - | - | - | + |
| hsa-miR-320a | NUDCD1 | - | - | - | + |
| hsa-miR-320a | NUDT10 | - | - | - | + |
| hsa-miR-320a | NUDT11 | - | - | - | + |
| hsa-miR-320a | NUDT15 | - | - | - | + |
| hsa-miR-320a | NUDT21 | - | - | + | + |
| hsa-miR-320a | NUDT5 | - | - | - | + |
| hsa-miR-320a | NUF2 | - | - | - | + |
| hsa-miR-320a | NUFIP1 | - | - | - | + |
| hsa-miR-320a | NUFIP2 | - | + | - | + |
| hsa-miR-320a | NUGGC | - | - | - | + |
| hsa-miR-320a | NUMB | - | - | - | + |
| hsa-miR-320a | NUP153 | - | - | - | + |
| hsa-miR-320a | NUP160 | - | - | - | + |
| hsa-miR-320a | NUP214 | - | - | - | + |
| hsa-miR-320a | NUP50 | - | + | - | + |
| hsa-miR-320a | NUS1 | - | + | - | + |
| hsa-miR-320a | NXF1 | - | - | - | + |
| hsa-miR-320a | NXT1 | - | - | + | - |
| hsa-miR-320a | NXT2 | - | - | + | + |
| hsa-miR-320a | NYAP2 | - | - | - | + |
| hsa-miR-320a | NYNRIN | - | - | - | + |
| hsa-miR-320a | OAZ1 | - | - | + | - |
| hsa-miR-320a | OCEL1 | + | - | - | + |
| hsa-miR-320a | OCIAD1 | - | - | - | + |
| hsa-miR-320a | OCLN | - | - | - | + |
| hsa-miR-320a | OCRL | - | - | + | - |
| hsa-miR-320a | ODF2 | - | - | - | + |
| hsa-miR-320a | ODZ3 | - | - | - | + |
| hsa-miR-320a | ODZ4 | - | - | - | + |
| hsa-miR-320a | OGDH | - | - | - | + |
| hsa-miR-320a | OGDHL | - | - | - | + |
| hsa-miR-320a | OGN | + | - | - | + |
| hsa-miR-320a | OGT | - | - | - | + |
| hsa-miR-320a | OLA1 | - | - | - | + |
| hsa-miR-320a | OLFM2 | - | - | - | + |
| hsa-miR-320a | OLFML2A | - | - | - | + |
| hsa-miR-320a | OLIG3 | + | - | - | + |
| hsa-miR-320a | OLR1 | - | - | - | + |
| hsa-miR-320a | ONECUT1 | + | - | - | + |
| hsa-miR-320a | ONECUT2 | - | + | - | + |
| hsa-miR-320a | OPA1 | - | - | + | + |
| hsa-miR-320a | OPALIN | - | - | - | + |
| hsa-miR-320a | OPHN1 | - | - | - | + |
| hsa-miR-320a | OPRL1 | - | - | - | + |
| hsa-miR-320a | OR2A4 | - | + | - | - |
| hsa-miR-320a | OR2C3 | - | - | - | + |
| hsa-miR-320a | OR2F1 | + | - | - | - |
| hsa-miR-320a | OR2F2 | - | - | - | + |
| hsa-miR-320a | OR4K2 | - | - | - | + |
| hsa-miR-320a | OR4K5 | - | - | - | + |
| hsa-miR-320a | OR7D2 | - | - | - | + |
| hsa-miR-320a | OR9Q1 | - | - | - | + |
| hsa-miR-320a | ORAI2 | - | - | + | + |
| hsa-miR-320a | ORC1 | - | - | + | + |
| hsa-miR-320a | ORM1 | + | - | - | - |
| hsa-miR-320a | ORMDL2 | - | - | + | - |
| hsa-miR-320a | ORMDL3 | - | - | - | + |
| hsa-miR-320a | OSBP | - | - | - | + |
| hsa-miR-320a | OSBP2 | - | - | - | + |
| hsa-miR-320a | OSBPL11 | - | - | - | + |
| hsa-miR-320a | OSBPL3 | - | - | - | + |
| hsa-miR-320a | OSBPL8 | - | - | - | + |
| hsa-miR-320a | OSGIN2 | - | - | - | + |
| hsa-miR-320a | OSTC | + | - | - | + |
| hsa-miR-320a | OSTF1 | - | - | - | + |
| hsa-miR-320a | OSTM1 | - | - | - | + |
| hsa-miR-320a | OTUD1 | - | - | + | + |
| hsa-miR-320a | OTUD3 | - | - | - | + |
| hsa-miR-320a | OTUD4 | - | - | - | + |
| hsa-miR-320a | OTUD6B | - | - | - | + |
| hsa-miR-320a | OXSR1 | - | - | - | + |
| hsa-miR-320a | P2RY8 | - | - | - | + |
| hsa-miR-320a | P4HA1 | - | - | + | - |
| hsa-miR-320a | P4HA2 | - | - | - | + |
| hsa-miR-320a | PAAF1 | - | - | - | + |
| hsa-miR-320a | PABPC1L2A | - | - | - | + |
| hsa-miR-320a | PABPC1L2B | - | - | - | + |
| hsa-miR-320a | PACRG | + | - | - | - |
| hsa-miR-320a | PACS1 | - | - | + | + |
| hsa-miR-320a | PACS2 | - | - | - | + |
| hsa-miR-320a | PAF1 | - | - | + | - |
| hsa-miR-320a | PAFAH1B1 | - | - | - | + |
| hsa-miR-320a | PAFAH1B2 | - | - | - | + |
| hsa-miR-320a | PAG1 | - | - | - | + |
| hsa-miR-320a | PAGE1 | + | - | - | + |
| hsa-miR-320a | PAICS | - | - | + | + |
| hsa-miR-320a | PAIP1 | - | - | - | + |
| hsa-miR-320a | PAIP2B | - | - | - | + |
| hsa-miR-320a | PAK1 | - | - | + | + |
| hsa-miR-320a | PAK2 | - | - | - | + |
| hsa-miR-320a | PAK7 | - | + | - | + |
| hsa-miR-320a | PALD1 | - | - | - | + |
| hsa-miR-320a | PALLD | - | - | - | + |
| hsa-miR-320a | PALM2-AKAP2 | - | - | - | + |
| hsa-miR-320a | PALMD | - | - | - | + |
| hsa-miR-320a | PAN3 | - | + | - | - |
| hsa-miR-320a | PANK3 | - | - | + | - |
| hsa-miR-320a | PANX1 | - | - | - | + |
| hsa-miR-320a | PAPD4 | - | - | - | + |
| hsa-miR-320a | PAPD5 | - | + | - | - |
| hsa-miR-320a | PAPD7 | - | - | - | + |
| hsa-miR-320a | PAPOLA | + | - | + | + |
| hsa-miR-320a | PAPOLB | - | - | - | + |
| hsa-miR-320a | PAPPA | - | - | - | + |
| hsa-miR-320a | PAQR5 | - | - | - | + |
| hsa-miR-320a | PAQR8 | - | - | - | + |
| hsa-miR-320a | PARD3 | - | - | - | + |
| hsa-miR-320a | PARD3B | - | - | - | + |
| hsa-miR-320a | PARD6B | - | - | - | + |
| hsa-miR-320a | PARD6G | - | - | - | + |
| hsa-miR-320a | PARK2 | - | + | - | + |
| hsa-miR-320a | PARP4 | - | - | - | + |
| hsa-miR-320a | PARP9 | - | - | - | + |
| hsa-miR-320a | PASD1 | - | - | - | + |
| hsa-miR-320a | PASK | - | - | - | + |
| hsa-miR-320a | PATE3 | + | - | - | + |
| hsa-miR-320a | PATL1 | - | - | - | + |
| hsa-miR-320a | PAX3 | - | - | - | + |
| hsa-miR-320a | PAX5 | - | - | - | + |
| hsa-miR-320a | PAX6 | - | - | - | + |
| hsa-miR-320a | PAX7 | - | - | - | + |
| hsa-miR-320a | PBRM1 | - | - | - | + |
| hsa-miR-320a | PBX1 | - | + | - | + |
| hsa-miR-320a | PBX3 | + | + | - | + |
| hsa-miR-320a | PCBP1 | - | - | - | + |
| hsa-miR-320a | PCBP2 | - | - | + | - |
| hsa-miR-320a | PCDH-alpha13 | - | + | - | - |
| hsa-miR-320a | PCDH-alpha3 | - | + | - | - |
| hsa-miR-320a | PCDH-alpha5 | - | + | - | - |
| hsa-miR-320a | PCDH-alpha7 | - | + | - | - |
| hsa-miR-320a | PCDH-alpha8 | - | + | - | - |
| hsa-miR-320a | PCDH10 | - | - | - | + |
| hsa-miR-320a | PCDH11X | - | - | - | + |
| hsa-miR-320a | PCDH11Y | - | - | - | + |
| hsa-miR-320a | PCDH12 | - | + | - | + |
| hsa-miR-320a | PCDH17 | - | - | - | + |
| hsa-miR-320a | PCDH18 | - | - | - | + |
| hsa-miR-320a | PCDH19 | - | + | - | + |
| hsa-miR-320a | PCDH20 | - | - | - | + |
| hsa-miR-320a | PCDH9 | - | - | - | + |
| hsa-miR-320a | PCDHA1 | - | + | - | + |
| hsa-miR-320a | PCDHA10 | - | + | - | + |
| hsa-miR-320a | PCDHA11 | - | + | - | + |
| hsa-miR-320a | PCDHA12 | - | + | - | + |
| hsa-miR-320a | PCDHA13 | - | - | - | + |
| hsa-miR-320a | PCDHA2 | - | + | - | + |
| hsa-miR-320a | PCDHA3 | - | - | - | + |
| hsa-miR-320a | PCDHA4 | - | + | - | + |
| hsa-miR-320a | PCDHA5 | - | - | - | + |
| hsa-miR-320a | PCDHA6 | - | + | - | + |
| hsa-miR-320a | PCDHA7 | - | - | - | + |
| hsa-miR-320a | PCDHA8 | - | - | - | + |
| hsa-miR-320a | PCDHA9 | - | + | - | - |
| hsa-miR-320a | PCDHAC1 | - | + | - | + |
| hsa-miR-320a | PCDHAC2 | - | + | - | + |
| hsa-miR-320a | PCDHB3 | - | - | - | + |
| hsa-miR-320a | PCDHB5 | - | - | - | + |
| hsa-miR-320a | PCDHB6 | - | - | - | + |
| hsa-miR-320a | PCDHGA1 | - | - | - | + |
| hsa-miR-320a | PCDHGA10 | - | - | - | + |
| hsa-miR-320a | PCDHGA11 | - | - | - | + |
| hsa-miR-320a | PCDHGA12 | - | - | - | + |
| hsa-miR-320a | PCDHGA2 | - | - | - | + |
| hsa-miR-320a | PCDHGA3 | - | - | - | + |
| hsa-miR-320a | PCDHGA4 | - | - | - | + |
| hsa-miR-320a | PCDHGA5 | - | - | - | + |
| hsa-miR-320a | PCDHGA6 | - | - | - | + |
| hsa-miR-320a | PCDHGA7 | - | - | - | + |
| hsa-miR-320a | PCDHGA8 | - | - | - | + |
| hsa-miR-320a | PCDHGA9 | - | - | - | + |
| hsa-miR-320a | PCDHGB1 | - | - | - | + |
| hsa-miR-320a | PCDHGB2 | - | - | - | + |
| hsa-miR-320a | PCDHGB3 | - | - | - | + |
| hsa-miR-320a | PCDHGB4 | - | - | - | + |
| hsa-miR-320a | PCDHGB6 | - | - | - | + |
| hsa-miR-320a | PCDHGB7 | - | - | - | + |
| hsa-miR-320a | PCDHGC3 | - | - | - | + |
| hsa-miR-320a | PCDHGC4 | - | - | - | + |
| hsa-miR-320a | PCDHGC5 | - | - | - | + |
| hsa-miR-320a | PCGF1 | + | - | - | - |
| hsa-miR-320a | PCGF3 | - | - | - | + |
| hsa-miR-320a | PCGF5 | - | - | - | + |
| hsa-miR-320a | PCLO | - | - | - | + |
| hsa-miR-320a | PCMT1 | - | - | - | + |
| hsa-miR-320a | PCMTD1 | - | - | + | + |
| hsa-miR-320a | PCMTD2 | - | - | - | + |
| hsa-miR-320a | PCNX | - | - | - | + |
| hsa-miR-320a | PCSK1 | - | - | - | + |
| hsa-miR-320a | PCSK2 | - | - | - | + |
| hsa-miR-320a | PCSK5 | - | - | - | + |
| hsa-miR-320a | PCSK7 | + | + | - | + |
| hsa-miR-320a | PCSK9 | - | - | - | + |
| hsa-miR-320a | PCYOX1 | - | - | - | + |
| hsa-miR-320a | PCYT1B | - | - | - | + |
| hsa-miR-320a | PDCD11 | - | - | - | + |
| hsa-miR-320a | PDCD2 | - | - | - | + |
| hsa-miR-320a | PDCD4 | - | - | - | + |
| hsa-miR-320a | PDCD6IP | - | - | - | + |
| hsa-miR-320a | PDCL | - | - | - | + |
| hsa-miR-320a | PDE10A | - | + | - | + |
| hsa-miR-320a | PDE11A | - | - | - | + |
| hsa-miR-320a | PDE1A | - | - | - | + |
| hsa-miR-320a | PDE1C | - | - | - | + |
| hsa-miR-320a | PDE3A | - | - | - | + |
| hsa-miR-320a | PDE3B | - | - | - | + |
| hsa-miR-320a | PDE4B | - | - | - | + |
| hsa-miR-320a | PDE4C | - | - | - | + |
| hsa-miR-320a | PDE4D | - | - | - | + |
| hsa-miR-320a | PDE5A | - | - | - | + |
| hsa-miR-320a | PDE6A | - | + | - | + |
| hsa-miR-320a | PDGFA | - | - | - | + |
| hsa-miR-320a | PDGFD | - | - | - | + |
| hsa-miR-320a | PDGFRA | - | - | - | + |
| hsa-miR-320a | PDHA1 | - | - | - | + |
| hsa-miR-320a | PDHX | - | - | + | - |
| hsa-miR-320a | PDIA3 | - | - | - | + |
| hsa-miR-320a | PDIA4 | - | - | - | + |
| hsa-miR-320a | PDIA6 | - | - | - | + |
| hsa-miR-320a | PDIK1L | + | + | - | + |
| hsa-miR-320a | PDK1 | - | + | - | - |
| hsa-miR-320a | PDK4 | - | - | - | + |
| hsa-miR-320a | PDLIM1 | - | - | + | + |
| hsa-miR-320a | PDLIM2 | - | - | - | + |
| hsa-miR-320a | PDLIM5 | - | - | - | + |
| hsa-miR-320a | PDP1 | - | - | - | + |
| hsa-miR-320a | PDP2 | - | - | - | + |
| hsa-miR-320a | PDPK1 | - | - | - | + |
| hsa-miR-320a | PDS5A | - | - | - | + |
| hsa-miR-320a | PDS5B | - | - | - | + |
| hsa-miR-320a | PDSS1 | - | - | + | - |
| hsa-miR-320a | PDXDC1 | - | - | - | + |
| hsa-miR-320a | PDZD2 | - | - | - | + |
| hsa-miR-320a | PDZK1 | - | - | - | + |
| hsa-miR-320a | PEA15 | - | - | - | + |
| hsa-miR-320a | PEAK1 | - | - | - | + |
| hsa-miR-320a | PEG10 | - | - | - | + |
| hsa-miR-320a | PEG3 | - | - | - | + |
| hsa-miR-320a | PELI2 | - | - | - | + |
| hsa-miR-320a | PER3 | - | - | - | + |
| hsa-miR-320a | PEX11B | - | - | - | + |
| hsa-miR-320a | PEX12 | - | + | - | - |
| hsa-miR-320a | PEX5L | - | - | - | + |
| hsa-miR-320a | PFDN4 | - | - | - | + |
| hsa-miR-320a | PFDN6 | - | - | + | - |
| hsa-miR-320a | PFKFB2 | - | + | - | + |
| hsa-miR-320a | PFKFB3 | - | - | - | + |
| hsa-miR-320a | PFKM | - | - | + | - |
| hsa-miR-320a | PFN1 | + | - | - | + |
| hsa-miR-320a | PGM2L1 | - | - | - | + |
| hsa-miR-320a | PGM3 | - | - | - | + |
| hsa-miR-320a | PGM5 | - | - | - | + |
| hsa-miR-320a | PGPEP1 | - | - | - | + |
| hsa-miR-320a | PGR | - | - | - | + |
| hsa-miR-320a | PHACTR2 | - | - | - | + |
| hsa-miR-320a | PHACTR4 | - | - | - | + |
| hsa-miR-320a | PHAX | - | - | - | + |
| hsa-miR-320a | PHB | - | - | - | + |
| hsa-miR-320a | PHC1 | - | + | - | - |
| hsa-miR-320a | PHC3 | - | + | - | - |
| hsa-miR-320a | PHEX | - | - | - | + |
| hsa-miR-320a | PHF1 | - | + | - | + |
| hsa-miR-320a | PHF13 | - | - | - | + |
| hsa-miR-320a | PHF17 | - | - | - | + |
| hsa-miR-320a | PHF19 | - | - | - | + |
| hsa-miR-320a | PHF3 | - | + | - | - |
| hsa-miR-320a | PHF6 | - | - | - | + |
| hsa-miR-320a | PHF8 | - | - | - | + |
| hsa-miR-320a | PHLPP1 | - | - | - | + |
| hsa-miR-320a | PHOSPHO1 | + | - | - | + |
| hsa-miR-320a | PHOX2B | - | - | - | + |
| hsa-miR-320a | PHTF2 | - | + | - | + |
| hsa-miR-320a | PHYHIPL | - | - | - | + |
| hsa-miR-320a | PI15 | - | - | - | + |
| hsa-miR-320a | PIBF1 | - | - | - | + |
| hsa-miR-320a | PICALM | - | + | - | + |
| hsa-miR-320a | PID1 | - | - | - | + |
| hsa-miR-320a | PIF1 | - | - | - | + |
| hsa-miR-320a | PIGA | - | - | - | + |
| hsa-miR-320a | PIGM | - | - | - | + |
| hsa-miR-320a | PIGN | - | + | - | + |
| hsa-miR-320a | PIGP | - | - | + | - |
| hsa-miR-320a | PIH1D3 | - | - | - | + |
| hsa-miR-320a | PIK3C2A | - | - | - | + |
| hsa-miR-320a | PIK3CA | - | - | - | + |
| hsa-miR-320a | PIK3R1 | - | - | - | + |
| hsa-miR-320a | PIKFYVE | - | - | - | + |
| hsa-miR-320a | PIM2 | - | - | - | + |
| hsa-miR-320a | PIP4K2A | - | - | - | + |
| hsa-miR-320a | PIP4K2B | - | - | + | - |
| hsa-miR-320a | PIP5K1A | - | - | - | + |
| hsa-miR-320a | PITHD1 | - | - | - | + |
| hsa-miR-320a | PITPNA | - | - | - | + |
| hsa-miR-320a | PITPNB | - | - | - | + |
| hsa-miR-320a | PITPNC1 | - | - | - | + |
| hsa-miR-320a | PITRM1 | - | - | - | + |
| hsa-miR-320a | PITX1 | - | - | + | - |
| hsa-miR-320a | PIWIL1 | - | - | - | + |
| hsa-miR-320a | PIWIL2 | - | - | - | + |
| hsa-miR-320a | PJA2 | - | - | - | + |
| hsa-miR-320a | PKD2 | - | - | - | + |
| hsa-miR-320a | PKDREJ | - | - | - | + |
| hsa-miR-320a | PKHD1 | - | - | - | + |
| hsa-miR-320a | PKHD1L1 | - | - | - | + |
| hsa-miR-320a | PKIA | - | - | - | + |
| hsa-miR-320a | PKM | - | - | + | - |
| hsa-miR-320a | PKN2 | - | - | - | + |
| hsa-miR-320a | PKNOX1 | - | - | - | + |
| hsa-miR-320a | PKNOX2 | - | - | - | + |
| hsa-miR-320a | PKP1 | - | - | - | + |
| hsa-miR-320a | PL-5283 | - | - | - | + |
| hsa-miR-320a | PLAG1 | - | - | - | + |
| hsa-miR-320a | PLAGL2 | - | - | - | + |
| hsa-miR-320a | PLCD3 | - | - | + | - |
| hsa-miR-320a | PLCXD3 | - | - | - | + |
| hsa-miR-320a | PLD3 | - | - | + | - |
| hsa-miR-320a | PLEC | - | - | - | + |
| hsa-miR-320a | PLEK | - | - | - | + |
| hsa-miR-320a | PLEKHA1 | - | - | - | + |
| hsa-miR-320a | PLEKHA5 | + | + | - | - |
| hsa-miR-320a | PLEKHA7 | - | - | - | + |
| hsa-miR-320a | PLEKHB2 | - | - | - | + |
| hsa-miR-320a | PLEKHF2 | - | - | - | + |
| hsa-miR-320a | PLEKHG1 | - | - | - | + |
| hsa-miR-320a | PLEKHG2 | - | - | - | + |
| hsa-miR-320a | PLEKHG5 | - | - | - | + |
| hsa-miR-320a | PLEKHH1 | - | - | - | + |
| hsa-miR-320a | PLEKHH2 | - | - | - | + |
| hsa-miR-320a | PLEKHM3 | - | - | - | + |
| hsa-miR-320a | PLEKHO1 | - | - | - | + |
| hsa-miR-320a | PLK2 | - | - | - | + |
| hsa-miR-320a | PLK3 | - | - | - | + |
| hsa-miR-320a | PLK4 | - | - | - | + |
| hsa-miR-320a | PLPPR1 | - | + | - | - |
| hsa-miR-320a | PLS1 | - | + | + | + |
| hsa-miR-320a | PLS3 | - | - | + | - |
| hsa-miR-320a | PLSCR4 | - | - | - | + |
| hsa-miR-320a | PLXDC2 | - | - | - | + |
| hsa-miR-320a | PLXNA2 | - | - | - | + |
| hsa-miR-320a | PLXNA4 | - | - | - | + |
| hsa-miR-320a | PLXNC1 | - | + | - | + |
| hsa-miR-320a | PM20D2 | - | - | - | + |
| hsa-miR-320a | PMP2 | - | - | - | + |
| hsa-miR-320a | PMP22 | - | - | - | + |
| hsa-miR-320a | PNMA2 | - | - | - | + |
| hsa-miR-320a | PNMAL1 | - | - | - | + |
| hsa-miR-320a | PNN | - | + | + | + |
| hsa-miR-320a | PNO1 | - | - | - | + |
| hsa-miR-320a | PNPLA8 | - | - | - | + |
| hsa-miR-320a | PNPO | + | + | - | - |
| hsa-miR-320a | PNRC2 | + | - | - | + |
| hsa-miR-320a | POC1B-GALNT4 | - | - | - | + |
| hsa-miR-320a | PODN | - | - | - | + |
| hsa-miR-320a | POFUT2 | - | - | - | + |
| hsa-miR-320a | POGZ | - | + | - | + |
| hsa-miR-320a | POLDIP3 | - | - | - | + |
| hsa-miR-320a | POLE3 | - | + | - | + |
| hsa-miR-320a | POLE4 | + | - | - | - |
| hsa-miR-320a | POLR1C | + | - | - | - |
| hsa-miR-320a | POLR2A | - | - | + | - |
| hsa-miR-320a | POLR2F | - | - | + | - |
| hsa-miR-320a | POLR2L | - | - | - | + |
| hsa-miR-320a | POLR2M | - | - | - | + |
| hsa-miR-320a | POLR3B | - | - | - | + |
| hsa-miR-320a | POLR3D | - | - | + | - |
| hsa-miR-320a | POLR3E | - | - | + | - |
| hsa-miR-320a | POM121 | - | - | + | + |
| hsa-miR-320a | POMT2 | - | - | - | + |
| hsa-miR-320a | POP1 | - | - | + | + |
| hsa-miR-320a | POT1 | - | - | - | + |
| hsa-miR-320a | POU1F1 | - | - | - | + |
| hsa-miR-320a | POU2F1 | + | - | - | - |
| hsa-miR-320a | POU2F2 | - | - | - | + |
| hsa-miR-320a | POU3F1 | - | - | - | + |
| hsa-miR-320a | POU3F2 | - | - | - | + |
| hsa-miR-320a | POU4F1 | - | - | - | + |
| hsa-miR-320a | POU4F2 | - | - | - | + |
| hsa-miR-320a | POU6F2 | - | - | - | + |
| hsa-miR-320a | PP13439 | - | - | + | - |
| hsa-miR-320a | PPA1 | + | - | - | + |
| hsa-miR-320a | PPAP2B | - | - | - | + |
| hsa-miR-320a | PPAPDC2 | - | - | - | + |
| hsa-miR-320a | PPARA | - | - | - | + |
| hsa-miR-320a | PPARGC1A | - | - | - | + |
| hsa-miR-320a | PPARGC1B | - | - | - | + |
| hsa-miR-320a | PPAT | - | - | - | + |
| hsa-miR-320a | PPCDC | - | - | - | + |
| hsa-miR-320a | PPCS | + | - | - | - |
| hsa-miR-320a | PPHLN1 | - | - | - | + |
| hsa-miR-320a | PPIA | - | - | + | - |
| hsa-miR-320a | PPIAL4G | - | - | + | - |
| hsa-miR-320a | PPIC | - | - | - | + |
| hsa-miR-320a | PPIF | - | - | + | - |
| hsa-miR-320a | PPIG | - | - | - | + |
| hsa-miR-320a | PPIL1 | - | - | + | - |
| hsa-miR-320a | PPIL3 | + | - | - | - |
| hsa-miR-320a | PPIL4 | - | - | + | - |
| hsa-miR-320a | PPIL6 | - | - | - | + |
| hsa-miR-320a | PPIP5K1 | - | - | - | + |
| hsa-miR-320a | PPM1A | - | + | - | + |
| hsa-miR-320a | PPM1B | - | + | - | + |
| hsa-miR-320a | PPM1F | - | - | - | + |
| hsa-miR-320a | PPM1H | - | - | - | + |
| hsa-miR-320a | PPP1CB | - | - | - | + |
| hsa-miR-320a | PPP1CC | - | - | - | + |
| hsa-miR-320a | PPP1R11 | - | + | - | - |
| hsa-miR-320a | PPP1R12A | - | - | - | + |
| hsa-miR-320a | PPP1R14A | - | - | + | - |
| hsa-miR-320a | PPP1R14C | - | - | - | + |
| hsa-miR-320a | PPP1R15B | - | - | - | + |
| hsa-miR-320a | PPP1R1C | - | - | - | + |
| hsa-miR-320a | PPP1R26 | - | - | - | + |
| hsa-miR-320a | PPP1R37 | - | - | - | + |
| hsa-miR-320a | PPP1R3B | - | - | - | + |
| hsa-miR-320a | PPP1R3C | - | - | - | + |
| hsa-miR-320a | PPP1R3D | - | - | - | + |
| hsa-miR-320a | PPP1R3E | - | - | - | + |
| hsa-miR-320a | PPP1R3F | - | - | - | + |
| hsa-miR-320a | PPP1R7 | - | - | + | + |
| hsa-miR-320a | PPP1R8 | - | - | - | + |
| hsa-miR-320a | PPP1R9A | - | - | - | + |
| hsa-miR-320a | PPP2CB | - | - | - | + |
| hsa-miR-320a | PPP2R1B | - | - | - | + |
| hsa-miR-320a | PPP2R2B | - | - | - | + |
| hsa-miR-320a | PPP2R2C | - | + | - | + |
| hsa-miR-320a | PPP2R3A | - | - | - | + |
| hsa-miR-320a | PPP2R5A | - | - | - | + |
| hsa-miR-320a | PPP2R5C | - | - | - | + |
| hsa-miR-320a | PPP3R1 | - | - | - | + |
| hsa-miR-320a | PPP3R2 | - | - | - | + |
| hsa-miR-320a | PPP4C | - | - | + | - |
| hsa-miR-320a | PPP4R3B | - | + | - | - |
| hsa-miR-320a | PPP6C | - | - | - | + |
| hsa-miR-320a | PPT1 | - | - | + | - |
| hsa-miR-320a | PPTC7 | - | - | - | + |
| hsa-miR-320a | PQBP1 | - | - | - | + |
| hsa-miR-320a | PRAMEF18 | + | - | - | - |
| hsa-miR-320a | PRAMEF19 | + | - | - | - |
| hsa-miR-320a | PRDM1 | - | - | - | + |
| hsa-miR-320a | PRDM10 | - | - | - | + |
| hsa-miR-320a | PRDM16 | - | - | - | + |
| hsa-miR-320a | PRDM6 | - | - | - | + |
| hsa-miR-320a | PRDX3 | - | - | + | - |
| hsa-miR-320a | PRDX6 | - | - | + | + |
| hsa-miR-320a | PRELP | - | - | - | + |
| hsa-miR-320a | PREPL | - | - | - | + |
| hsa-miR-320a | PRICKLE1 | - | - | - | + |
| hsa-miR-320a | PRICKLE2 | - | - | - | + |
| hsa-miR-320a | PRKAA1 | - | - | - | + |
| hsa-miR-320a | PRKAA2 | - | - | - | + |
| hsa-miR-320a | PRKAB1 | - | - | - | + |
| hsa-miR-320a | PRKAB2 | - | - | - | + |
| hsa-miR-320a | PRKACA | - | - | - | + |
| hsa-miR-320a | PRKAG2 | + | - | - | + |
| hsa-miR-320a | PRKAR2A | - | - | - | + |
| hsa-miR-320a | PRKAR2B | - | - | - | + |
| hsa-miR-320a | PRKCA | - | - | - | + |
| hsa-miR-320a | PRKCB | - | - | - | + |
| hsa-miR-320a | PRKCE | - | - | - | + |
| hsa-miR-320a | PRKCG | - | + | - | + |
| hsa-miR-320a | PRKD1 | - | - | - | + |
| hsa-miR-320a | PRKG1 | - | - | - | + |
| hsa-miR-320a | PRMT5 | - | - | + | - |
| hsa-miR-320a | PRMT8 | - | - | - | + |
| hsa-miR-320a | PRND | - | - | - | + |
| hsa-miR-320a | PROK1 | + | - | - | + |
| hsa-miR-320a | PROK2 | + | - | - | + |
| hsa-miR-320a | PROS1 | - | - | - | + |
| hsa-miR-320a | PROSC | - | - | - | + |
| hsa-miR-320a | PROSER1 | - | - | + | - |
| hsa-miR-320a | PROSER2 | - | - | - | + |
| hsa-miR-320a | PROX1 | - | - | - | + |
| hsa-miR-320a | PROX2 | - | - | - | + |
| hsa-miR-320a | PRPF40A | - | - | - | + |
| hsa-miR-320a | PRPF4B | - | - | - | + |
| hsa-miR-320a | PRPS1 | - | + | - | + |
| hsa-miR-320a | PRR12 | - | - | + | - |
| hsa-miR-320a | PRR14L | - | + | + | - |
| hsa-miR-320a | PRR15 | - | - | - | + |
| hsa-miR-320a | PRR16 | - | - | - | + |
| hsa-miR-320a | PRR23B | - | - | - | + |
| hsa-miR-320a | PRR26 | + | - | - | - |
| hsa-miR-320a | PRR7 | - | - | - | + |
| hsa-miR-320a | PRRC2C | - | - | + | + |
| hsa-miR-320a | PRRG1 | - | - | - | + |
| hsa-miR-320a | PRRT2 | - | - | - | + |
| hsa-miR-320a | PRSS12 | - | - | - | + |
| hsa-miR-320a | PRSS16 | - | + | - | + |
| hsa-miR-320a | PRSS23 | - | - | - | + |
| hsa-miR-320a | PRSS35 | - | - | - | + |
| hsa-miR-320a | PRSS46 | - | - | - | + |
| hsa-miR-320a | PRSS53 | - | + | - | + |
| hsa-miR-320a | PRTFDC1 | - | - | - | + |
| hsa-miR-320a | PRTG | - | - | - | + |
| hsa-miR-320a | PSAT1 | - | - | - | + |
| hsa-miR-320a | PSD3 | - | - | - | + |
| hsa-miR-320a | PSEN1 | - | - | - | + |
| hsa-miR-320a | PSG3 | - | - | - | + |
| hsa-miR-320a | PSIP1 | - | - | - | + |
| hsa-miR-320a | PSMA5 | - | + | - | + |
| hsa-miR-320a | PSMB1 | - | - | - | + |
| hsa-miR-320a | PSMB11 | - | - | - | + |
| hsa-miR-320a | PSMD10 | - | - | + | - |
| hsa-miR-320a | PSMD11 | - | - | + | + |
| hsa-miR-320a | PSME3 | - | - | - | + |
| hsa-miR-320a | PSMF1 | - | - | + | + |
| hsa-miR-320a | PSMG4 | + | - | - | - |
| hsa-miR-320a | PSRC1 | - | - | + | - |
| hsa-miR-320a | PTAR1 | - | - | - | + |
| hsa-miR-320a | PTBP3 | - | - | - | + |
| hsa-miR-320a | PTCD3 | - | - | - | + |
| hsa-miR-320a | PTCHD2 | - | - | - | + |
| hsa-miR-320a | PTEN | - | + | + | + |
| hsa-miR-320a | PTGER3 | - | - | - | + |
| hsa-miR-320a | PTGER4 | - | - | - | + |
| hsa-miR-320a | PTGES3 | - | - | - | + |
| hsa-miR-320a | PTGFRN | - | + | - | + |
| hsa-miR-320a | PTGIS | - | - | - | + |
| hsa-miR-320a | PTGS1 | - | - | - | + |
| hsa-miR-320a | PTP4A2 | - | - | + | - |
| hsa-miR-320a | PTPN1 | - | - | - | + |
| hsa-miR-320a | PTPN11 | - | - | + | + |
| hsa-miR-320a | PTPN12 | - | - | - | + |
| hsa-miR-320a | PTPN13 | - | - | - | + |
| hsa-miR-320a | PTPN3 | - | - | - | + |
| hsa-miR-320a | PTPN4 | - | - | - | + |
| hsa-miR-320a | PTPN9 | - | - | + | - |
| hsa-miR-320a | PTPRA | - | - | - | + |
| hsa-miR-320a | PTPRD | - | - | - | + |
| hsa-miR-320a | PTPRE | - | - | - | + |
| hsa-miR-320a | PTPRG | - | - | - | + |
| hsa-miR-320a | PTPRM | - | - | - | + |
| hsa-miR-320a | PTPRN | - | - | - | + |
| hsa-miR-320a | PTPRS | - | - | - | + |
| hsa-miR-320a | PTPRT | - | - | - | + |
| hsa-miR-320a | PTPRU | - | - | - | + |
| hsa-miR-320a | PTRF | - | - | - | + |
| hsa-miR-320a | PTTG1 | - | - | + | - |
| hsa-miR-320a | PTTG1IP | - | - | - | + |
| hsa-miR-320a | PTX3 | - | - | - | + |
| hsa-miR-320a | PUM1 | - | - | - | + |
| hsa-miR-320a | PUM2 | - | - | - | + |
| hsa-miR-320a | PURB | - | - | - | + |
| hsa-miR-320a | PUS7 | - | + | - | + |
| hsa-miR-320a | PUS7L | - | - | - | + |
| hsa-miR-320a | PUSL1 | + | - | - | + |
| hsa-miR-320a | PVRL2 | - | - | + | - |
| hsa-miR-320a | PXDC1 | - | - | - | + |
| hsa-miR-320a | PXN | - | - | - | + |
| hsa-miR-320a | PXYLP1 | - | - | - | + |
| hsa-miR-320a | PYGO1 | - | - | - | + |
| hsa-miR-320a | PYGO2 | + | - | + | + |
| hsa-miR-320a | PYROXD1 | - | - | - | + |
| hsa-miR-320a | PYURF | - | - | - | + |
| hsa-miR-320a | QKI | - | - | - | + |
| hsa-miR-320a | QSER1 | - | - | - | + |
| hsa-miR-320a | QSOX2 | - | + | - | + |
| hsa-miR-320a | QTRTD1 | - | - | - | + |
| hsa-miR-320a | R3HDM1 | - | - | + | + |
| hsa-miR-320a | RAB10 | - | - | - | + |
| hsa-miR-320a | RAB11A | + | - | - | + |
| hsa-miR-320a | RAB11FIP1 | - | - | - | + |
| hsa-miR-320a | RAB11FIP2 | + | + | - | + |
| hsa-miR-320a | RAB11FIP3 | - | - | - | + |
| hsa-miR-320a | RAB11FIP5 | - | - | - | + |
| hsa-miR-320a | RAB12 | - | - | - | + |
| hsa-miR-320a | RAB14 | - | - | - | + |
| hsa-miR-320a | RAB15 | - | - | - | + |
| hsa-miR-320a | RAB18 | - | + | - | + |
| hsa-miR-320a | RAB1B | - | - | - | + |
| hsa-miR-320a | RAB22A | - | - | - | + |
| hsa-miR-320a | RAB24 | - | - | - | + |
| hsa-miR-320a | RAB27A | - | + | - | + |
| hsa-miR-320a | RAB27B | - | - | - | + |
| hsa-miR-320a | RAB29 | - | - | - | + |
| hsa-miR-320a | RAB2A | - | - | + | + |
| hsa-miR-320a | RAB30 | - | - | - | + |
| hsa-miR-320a | RAB32 | - | - | - | + |
| hsa-miR-320a | RAB33B | - | - | - | + |
| hsa-miR-320a | RAB35 | - | - | - | + |
| hsa-miR-320a | RAB36 | - | - | - | + |
| hsa-miR-320a | RAB39B | - | + | - | + |
| hsa-miR-320a | RAB3C | - | - | - | + |
| hsa-miR-320a | RAB3D | - | + | - | + |
| hsa-miR-320a | RAB3GAP1 | - | - | - | + |
| hsa-miR-320a | RAB3GAP2 | - | + | - | + |
| hsa-miR-320a | RAB3IP | - | - | - | + |
| hsa-miR-320a | RAB4A | - | - | - | + |
| hsa-miR-320a | RAB5A | - | - | - | + |
| hsa-miR-320a | RAB6A | - | - | - | + |
| hsa-miR-320a | RAB6B | - | - | - | + |
| hsa-miR-320a | RAB6C | - | - | - | + |
| hsa-miR-320a | RAB7A | - | - | - | + |
| hsa-miR-320a | RAB8B | - | - | - | + |
| hsa-miR-320a | RAB9B | - | - | - | + |
| hsa-miR-320a | RABGAP1L | - | - | - | + |
| hsa-miR-320a | RABL3 | - | - | - | + |
| hsa-miR-320a | RAC1 | + | - | + | + |
| hsa-miR-320a | RACGAP1 | - | - | - | + |
| hsa-miR-320a | RAD17 | - | - | - | + |
| hsa-miR-320a | RAD18 | - | + | - | + |
| hsa-miR-320a | RAD21 | - | + | - | + |
| hsa-miR-320a | RAD21-AS1 | - | - | - | + |
| hsa-miR-320a | RAD23A | - | - | + | - |
| hsa-miR-320a | RAD23B | - | - | - | + |
| hsa-miR-320a | RAD51 | + | + | - | + |
| hsa-miR-320a | RAD51B | - | - | - | + |
| hsa-miR-320a | RAD9A | - | - | - | + |
| hsa-miR-320a | RAG1 | - | - | - | + |
| hsa-miR-320a | RAI14 | - | - | - | + |
| hsa-miR-320a | RAI2 | - | + | - | + |
| hsa-miR-320a | RALA | - | - | - | + |
| hsa-miR-320a | RALBP1 | - | - | - | + |
| hsa-miR-320a | RALGPS1 | - | + | - | - |
| hsa-miR-320a | RALY | - | - | + | - |
| hsa-miR-320a | RAN | - | - | + | - |
| hsa-miR-320a | RANBP10 | - | + | - | - |
| hsa-miR-320a | RANBP2 | - | - | - | + |
| hsa-miR-320a | RANBP6 | - | - | + | + |
| hsa-miR-320a | RAP1A | + | + | - | + |
| hsa-miR-320a | RAP1GAP2 | - | - | - | + |
| hsa-miR-320a | RAP1GDS1 | - | - | - | + |
| hsa-miR-320a | RAP2C | - | - | - | + |
| hsa-miR-320a | RAPGEF3 | - | - | - | + |
| hsa-miR-320a | RAPGEF5 | - | - | - | + |
| hsa-miR-320a | RAPGEF6 | - | - | - | + |
| hsa-miR-320a | RAPH1 | - | + | - | + |
| hsa-miR-320a | RARG | - | - | - | + |
| hsa-miR-320a | RASA1 | + | + | - | - |
| hsa-miR-320a | RASA2 | - | - | - | + |
| hsa-miR-320a | RASAL1 | - | - | - | + |
| hsa-miR-320a | RASD2 | - | - | - | + |
| hsa-miR-320a | RASGRF1 | - | - | - | + |
| hsa-miR-320a | RASGRF2 | - | - | - | + |
| hsa-miR-320a | RASGRP1 | - | - | - | + |
| hsa-miR-320a | RASGRP3 | - | - | - | + |
| hsa-miR-320a | RASSF2 | - | - | - | + |
| hsa-miR-320a | RASSF3 | - | - | - | + |
| hsa-miR-320a | RASSF6 | - | - | - | + |
| hsa-miR-320a | RASSF8 | - | - | - | + |
| hsa-miR-320a | RAVER2 | - | - | - | + |
| hsa-miR-320a | RB1 | - | - | - | + |
| hsa-miR-320a | RBAK | - | - | - | + |
| hsa-miR-320a | RBBP5 | - | - | - | + |
| hsa-miR-320a | RBFOX1 | + | - | - | + |
| hsa-miR-320a | RBFOX2 | - | - | - | + |
| hsa-miR-320a | RBFOX3 | - | - | - | + |
| hsa-miR-320a | RBL1 | - | - | - | + |
| hsa-miR-320a | RBL2 | - | - | - | + |
| hsa-miR-320a | RBM12 | - | - | - | + |
| hsa-miR-320a | RBM20 | - | - | - | + |
| hsa-miR-320a | RBM24 | + | + | - | + |
| hsa-miR-320a | RBM27 | - | - | - | + |
| hsa-miR-320a | RBM38 | - | - | + | - |
| hsa-miR-320a | RBM41 | - | + | - | - |
| hsa-miR-320a | RBM45 | - | + | - | - |
| hsa-miR-320a | RBMS1 | - | - | - | + |
| hsa-miR-320a | RBMS2 | - | - | - | + |
| hsa-miR-320a | RBMXL1 | - | + | - | + |
| hsa-miR-320a | RBMXL2 | - | - | - | + |
| hsa-miR-320a | RBMXL3 | - | - | - | + |
| hsa-miR-320a | RBP7 | + | - | - | + |
| hsa-miR-320a | RBPMS | - | - | - | + |
| hsa-miR-320a | RC3H1 | - | + | - | + |
| hsa-miR-320a | RCAN1 | - | - | - | + |
| hsa-miR-320a | RCAN2 | - | - | - | + |
| hsa-miR-320a | RCBTB2 | - | - | - | + |
| hsa-miR-320a | RCC2 | - | - | - | + |
| hsa-miR-320a | RCCD1 | - | - | - | + |
| hsa-miR-320a | RCN2 | + | + | - | - |
| hsa-miR-320a | RCOR3 | - | - | - | + |
| hsa-miR-320a | RCSD1 | - | - | - | + |
| hsa-miR-320a | RD3L | - | - | - | + |
| hsa-miR-320a | RDX | - | - | - | + |
| hsa-miR-320a | RECK | - | - | - | + |
| hsa-miR-320a | REEP1 | - | - | - | + |
| hsa-miR-320a | REEP3 | - | + | - | + |
| hsa-miR-320a | RELA | - | - | + | - |
| hsa-miR-320a | RELT | - | - | - | + |
| hsa-miR-320a | REPIN1 | - | - | + | - |
| hsa-miR-320a | REPS2 | - | - | - | + |
| hsa-miR-320a | RERE | - | - | + | + |
| hsa-miR-320a | REST | - | - | - | + |
| hsa-miR-320a | RFESD | + | + | - | - |
| hsa-miR-320a | RFNG | - | - | - | + |
| hsa-miR-320a | RFPL1 | - | - | - | + |
| hsa-miR-320a | RFT1 | - | - | - | + |
| hsa-miR-320a | RFTN1 | - | - | - | + |
| hsa-miR-320a | RFX1 | - | - | - | + |
| hsa-miR-320a | RFX3 | - | - | - | + |
| hsa-miR-320a | RFX7 | - | - | + | - |
| hsa-miR-320a | RGCC | - | - | - | + |
| hsa-miR-320a | RGL1 | - | - | - | + |
| hsa-miR-320a | RGMB | - | - | - | + |
| hsa-miR-320a | RGPD3 | - | - | - | + |
| hsa-miR-320a | RGPD4 | - | - | - | + |
| hsa-miR-320a | RGS10 | + | - | - | + |
| hsa-miR-320a | RGS18 | - | - | - | + |
| hsa-miR-320a | RGS4 | - | - | - | + |
| hsa-miR-320a | RGS5 | - | - | - | + |
| hsa-miR-320a | RGS7BP | - | - | - | + |
| hsa-miR-320a | RGS9BP | - | + | - | + |
| hsa-miR-320a | RHAG | - | - | - | + |
| hsa-miR-320a | RHBDD1 | - | - | - | + |
| hsa-miR-320a | RHD | - | - | - | + |
| hsa-miR-320a | RHEB | - | - | - | + |
| hsa-miR-320a | RHOBTB1 | - | + | - | + |
| hsa-miR-320a | RHOBTB2 | - | - | - | + |
| hsa-miR-320a | RHOBTB3 | - | - | - | + |
| hsa-miR-320a | RHOG | - | + | - | + |
| hsa-miR-320a | RHOU | - | - | - | + |
| hsa-miR-320a | RIC8B | - | - | - | + |
| hsa-miR-320a | RIMBP2 | - | - | - | + |
| hsa-miR-320a | RIMS1 | - | - | - | + |
| hsa-miR-320a | RIMS4 | - | - | - | + |
| hsa-miR-320a | RIN2 | - | - | - | + |
| hsa-miR-320a | RING1 | - | - | - | + |
| hsa-miR-320a | RIOK2 | - | - | - | + |
| hsa-miR-320a | RIOK3 | - | + | - | + |
| hsa-miR-320a | RIT1 | - | + | - | + |
| hsa-miR-320a | RIT2 | - | - | - | + |
| hsa-miR-320a | RMDN3 | - | + | - | - |
| hsa-miR-320a | RMND5A | - | - | - | + |
| hsa-miR-320a | RNASE2 | + | - | - | + |
| hsa-miR-320a | RNASE3 | + | - | - | + |
| hsa-miR-320a | RNASET2 | + | - | - | - |
| hsa-miR-320a | RND3 | - | - | - | + |
| hsa-miR-320a | RNF10 | - | - | + | - |
| hsa-miR-320a | RNF103 | - | - | + | + |
| hsa-miR-320a | RNF111 | - | - | - | + |
| hsa-miR-320a | RNF114 | - | - | - | + |
| hsa-miR-320a | RNF125 | - | - | + | + |
| hsa-miR-320a | RNF128 | - | - | - | + |
| hsa-miR-320a | RNF130 | - | - | + | - |
| hsa-miR-320a | RNF138 | - | + | - | + |
| hsa-miR-320a | RNF14 | - | - | - | + |
| hsa-miR-320a | RNF141 | - | - | - | + |
| hsa-miR-320a | RNF144A | - | - | - | + |
| hsa-miR-320a | RNF144B | - | - | - | + |
| hsa-miR-320a | RNF145 | - | - | - | + |
| hsa-miR-320a | RNF149 | - | - | - | + |
| hsa-miR-320a | RNF157 | - | - | - | + |
| hsa-miR-320a | RNF168 | - | - | - | + |
| hsa-miR-320a | RNF169 | - | - | - | + |
| hsa-miR-320a | RNF180 | - | - | - | + |
| hsa-miR-320a | RNF185 | + | + | - | + |
| hsa-miR-320a | RNF2 | - | - | - | + |
| hsa-miR-320a | RNF20 | - | - | - | + |
| hsa-miR-320a | RNF212 | - | - | - | + |
| hsa-miR-320a | RNF213 | - | - | - | + |
| hsa-miR-320a | RNF216 | - | - | + | - |
| hsa-miR-320a | RNF32 | - | - | - | + |
| hsa-miR-320a | RNF34 | - | - | - | + |
| hsa-miR-320a | RNF38 | - | - | - | + |
| hsa-miR-320a | RNF41 | - | - | + | - |
| hsa-miR-320a | RNF6 | - | - | - | + |
| hsa-miR-320a | RNF8 | - | - | - | + |
| hsa-miR-320a | RNGTT | - | - | - | + |
| hsa-miR-320a | RNPS1 | - | - | - | + |
| hsa-miR-320a | ROCK2 | - | - | - | + |
| hsa-miR-320a | ROR2 | - | - | - | + |
| hsa-miR-320a | RORA | - | - | - | + |
| hsa-miR-320a | RORC | - | - | - | + |
| hsa-miR-320a | RP1-127H14.3 | + | - | - | - |
| hsa-miR-320a | RP11-1118M6.1 | - | - | - | + |
| hsa-miR-320a | RP11-111M22.2 | - | - | - | + |
| hsa-miR-320a | RP11-114H20.1 | - | - | - | + |
| hsa-miR-320a | RP11-122A3.2 | - | - | - | + |
| hsa-miR-320a | RP11-17A1.2 | - | - | - | + |
| hsa-miR-320a | RP11-322L20.1 | - | - | - | + |
| hsa-miR-320a | RP11-382J12.1 | - | - | - | + |
| hsa-miR-320a | RP11-422N16.3 | - | - | - | + |
| hsa-miR-320a | RP11-625H11.1 | + | - | - | + |
| hsa-miR-320a | RP11-65D24.2 | + | - | - | - |
| hsa-miR-320a | RP11-664D7.4 | - | - | - | + |
| hsa-miR-320a | RP11-676J12.7 | - | - | - | + |
| hsa-miR-320a | RP11-758M4.1 | - | - | - | + |
| hsa-miR-320a | RP11-762I7.5 | + | - | - | - |
| hsa-miR-320a | RP11-796G6.2 | + | - | - | + |
| hsa-miR-320a | RP11-863K10.7 | - | - | - | + |
| hsa-miR-320a | RP1L1 | - | - | - | + |
| hsa-miR-320a | RP2 | - | - | - | + |
| hsa-miR-320a | RP3-324N14.2 | - | - | - | + |
| hsa-miR-320a | RP5-1187M17.10 | - | - | - | + |
| hsa-miR-320a | RPA3-AS1 | + | - | - | - |
| hsa-miR-320a | RPAP1 | - | - | + | - |
| hsa-miR-320a | RPAP3 | - | - | - | + |
| hsa-miR-320a | RPL10 | - | - | - | + |
| hsa-miR-320a | RPL13A | - | - | + | + |
| hsa-miR-320a | RPL15 | - | + | - | - |
| hsa-miR-320a | RPL27 | - | - | + | - |
| hsa-miR-320a | RPL27A | - | - | - | + |
| hsa-miR-320a | RPL28 | - | - | - | + |
| hsa-miR-320a | RPL30 | - | - | + | - |
| hsa-miR-320a | RPL36 | - | - | + | - |
| hsa-miR-320a | RPL39L | - | - | - | + |
| hsa-miR-320a | RPL7A | - | - | + | - |
| hsa-miR-320a | RPL8 | - | - | + | - |
| hsa-miR-320a | RPL9 | - | - | + | - |
| hsa-miR-320a | RPLP1 | - | - | + | - |
| hsa-miR-320a | RPRD2 | - | - | + | + |
| hsa-miR-320a | RPS12 | - | - | + | - |
| hsa-miR-320a | RPS16 | - | - | + | - |
| hsa-miR-320a | RPS17 | - | - | + | - |
| hsa-miR-320a | RPS24 | - | - | - | + |
| hsa-miR-320a | RPS27 | - | - | + | - |
| hsa-miR-320a | RPS4X | - | - | + | - |
| hsa-miR-320a | RPS6KA1 | - | - | + | - |
| hsa-miR-320a | RPS6KA2 | - | - | - | + |
| hsa-miR-320a | RPS6KA3 | - | - | + | + |
| hsa-miR-320a | RPS6KB1 | - | - | - | + |
| hsa-miR-320a | RPTN | - | - | - | + |
| hsa-miR-320a | RRAGC | - | - | - | + |
| hsa-miR-320a | RRAGD | - | - | - | + |
| hsa-miR-320a | RRAS2 | - | - | - | + |
| hsa-miR-320a | RRM2B | - | - | - | + |
| hsa-miR-320a | RRN3 | - | + | - | + |
| hsa-miR-320a | RRP15 | - | - | - | + |
| hsa-miR-320a | RRP1B | - | + | - | + |
| hsa-miR-320a | RS1 | - | - | - | + |
| hsa-miR-320a | RSAD2 | - | - | - | + |
| hsa-miR-320a | RSBN1 | - | - | - | + |
| hsa-miR-320a | RSF1 | - | - | - | + |
| hsa-miR-320a | RSL1D1 | - | - | - | + |
| hsa-miR-320a | RTEL1-TNFRSF6B | - | - | - | + |
| hsa-miR-320a | RTF1 | - | - | - | + |
| hsa-miR-320a | RTKN | - | - | - | + |
| hsa-miR-320a | RTKN2 | - | - | - | + |
| hsa-miR-320a | RTN3 | - | - | - | + |
| hsa-miR-320a | RTN4 | - | - | + | - |
| hsa-miR-320a | RTP1 | - | - | - | + |
| hsa-miR-320a | RUFY2 | - | + | - | + |
| hsa-miR-320a | RUNDC1 | - | - | - | + |
| hsa-miR-320a | RUNDC3B | - | - | - | + |
| hsa-miR-320a | RUNX1T1 | - | - | - | + |
| hsa-miR-320a | RUNX2 | - | + | - | + |
| hsa-miR-320a | RWDD2B | - | - | - | + |
| hsa-miR-320a | RWDD4 | - | - | - | + |
| hsa-miR-320a | RXFP2 | - | - | - | + |
| hsa-miR-320a | RYR3 | - | - | - | + |
| hsa-miR-320a | S100A10 | - | - | - | + |
| hsa-miR-320a | S100PBP | - | - | - | + |
| hsa-miR-320a | S100Z | - | - | - | + |
| hsa-miR-320a | S1PR1 | - | - | - | + |
| hsa-miR-320a | SALL1 | - | - | + | + |
| hsa-miR-320a | SAMD12 | - | - | - | + |
| hsa-miR-320a | SAMD4A | - | - | - | + |
| hsa-miR-320a | SAMD4B | - | - | + | + |
| hsa-miR-320a | SAMD5 | - | - | - | + |
| hsa-miR-320a | SAMD8 | - | - | - | + |
| hsa-miR-320a | SAMD9L | - | - | - | + |
| hsa-miR-320a | SAP30 | - | - | - | + |
| hsa-miR-320a | SAR1B | - | - | - | + |
| hsa-miR-320a | SARNP | - | + | - | - |
| hsa-miR-320a | SASH1 | - | + | - | + |
| hsa-miR-320a | SASS6 | - | - | - | + |
| hsa-miR-320a | SAT1 | - | - | - | + |
| hsa-miR-320a | SATB2 | - | - | + | + |
| hsa-miR-320a | SAXO1 | - | + | - | + |
| hsa-miR-320a | SAYSD1 | - | - | - | + |
| hsa-miR-320a | SBF1 | - | - | + | - |
| hsa-miR-320a | SBNO1 | - | - | - | + |
| hsa-miR-320a | SCAF1 | - | - | + | - |
| hsa-miR-320a | SCD | - | - | - | + |
| hsa-miR-320a | SCFD2 | - | - | - | + |
| hsa-miR-320a | SCG3 | - | - | - | + |
| hsa-miR-320a | SCG5 | - | - | - | + |
| hsa-miR-320a | SCLT1 | - | - | - | + |
| hsa-miR-320a | SCLY | + | - | - | - |
| hsa-miR-320a | SCN10A | - | - | - | + |
| hsa-miR-320a | SCN1A | - | - | - | + |
| hsa-miR-320a | SCN2A | - | - | - | + |
| hsa-miR-320a | SCN2B | - | - | - | + |
| hsa-miR-320a | SCN3A | - | - | - | + |
| hsa-miR-320a | SCN3B | - | - | - | + |
| hsa-miR-320a | SCN4B | - | - | - | + |
| hsa-miR-320a | SCN9A | - | - | - | + |
| hsa-miR-320a | SCNM1 | - | - | - | + |
| hsa-miR-320a | SCNN1G | - | - | - | + |
| hsa-miR-320a | SCOC | + | + | - | - |
| hsa-miR-320a | SCRN1 | - | - | - | + |
| hsa-miR-320a | SCUBE3 | - | - | - | + |
| hsa-miR-320a | SCYL2 | - | - | - | + |
| hsa-miR-320a | SCYL3 | - | - | - | + |
| hsa-miR-320a | SDC1 | - | - | - | + |
| hsa-miR-320a | SDC2 | - | - | - | + |
| hsa-miR-320a | SDC4 | - | - | - | + |
| hsa-miR-320a | SDCBP | - | - | - | + |
| hsa-miR-320a | SDCBP2 | - | - | - | + |
| hsa-miR-320a | SDCCAG3 | - | - | - | + |
| hsa-miR-320a | SDF2 | - | - | - | + |
| hsa-miR-320a | SDHAF3 | - | - | - | + |
| hsa-miR-320a | SDHC | - | - | + | - |
| hsa-miR-320a | SDHD | - | + | - | + |
| hsa-miR-320a | SDK1 | - | - | - | + |
| hsa-miR-320a | SDK2 | - | - | - | + |
| hsa-miR-320a | SDR9C7 | - | - | - | + |
| hsa-miR-320a | SEC14L1 | - | + | + | - |
| hsa-miR-320a | SEC14L5 | - | - | - | + |
| hsa-miR-320a | SEC22C | - | + | - | - |
| hsa-miR-320a | SEC23B | - | - | - | + |
| hsa-miR-320a | SEC24A | - | - | + | + |
| hsa-miR-320a | SEC61A1 | - | - | - | + |
| hsa-miR-320a | SEC62 | - | - | - | + |
| hsa-miR-320a | SEC63 | + | + | - | + |
| hsa-miR-320a | SECISBP2 | - | - | - | + |
| hsa-miR-320a | SECISBP2L | - | - | - | + |
| hsa-miR-320a | SEH1L | - | - | - | + |
| hsa-miR-320a | SEL1L | - | + | - | + |
| hsa-miR-320a | SELL | - | - | - | + |
| hsa-miR-320a | SELT | - | - | - | + |
| hsa-miR-320a | SEMA3A | - | + | - | + |
| hsa-miR-320a | SEMA3C | - | - | - | + |
| hsa-miR-320a | SEMA3D | - | - | - | + |
| hsa-miR-320a | SEMA5A | - | - | - | + |
| hsa-miR-320a | SEMA6A | - | - | - | + |
| hsa-miR-320a | SEMA6D | - | + | - | + |
| hsa-miR-320a | SEMA7A | - | - | + | + |
| hsa-miR-320a | SENP3 | - | - | + | - |
| hsa-miR-320a | SENP5 | - | - | - | + |
| hsa-miR-320a | SENP7 | - | - | - | + |
| hsa-miR-320a | 15-Sep | - | + | - | + |
| hsa-miR-320a | SEPN1 | - | - | - | + |
| hsa-miR-320a | SEPP1 | - | - | - | + |
| hsa-miR-320a | SEPSECS | - | + | - | - |
| hsa-miR-320a | 1-Sep | - | - | + | - |
| hsa-miR-320a | 10-Sep | - | - | - | + |
| hsa-miR-320a | 2-Sep | - | - | - | + |
| hsa-miR-320a | 6-Sep | - | - | - | + |
| hsa-miR-320a | 8-Sep | - | - | - | + |
| hsa-miR-320a | SERBP1 | - | - | - | + |
| hsa-miR-320a | SERF1A | - | + | - | - |
| hsa-miR-320a | SERF1B | - | + | - | - |
| hsa-miR-320a | SERINC1 | - | - | - | + |
| hsa-miR-320a | SERINC3 | - | + | - | - |
| hsa-miR-320a | SERP1 | - | - | - | + |
| hsa-miR-320a | SERPINB5 | - | - | - | + |
| hsa-miR-320a | SERPINB9 | - | - | - | + |
| hsa-miR-320a | SERPINE1 | - | - | - | + |
| hsa-miR-320a | SERPINE2 | - | - | - | + |
| hsa-miR-320a | SERPINF1 | + | - | - | - |
| hsa-miR-320a | SERPING1 | - | + | - | - |
| hsa-miR-320a | SERTAD2 | - | - | - | + |
| hsa-miR-320a | SERTM1 | - | - | - | + |
| hsa-miR-320a | SESN1 | - | - | - | + |
| hsa-miR-320a | SESN3 | - | - | - | + |
| hsa-miR-320a | SESTD1 | - | - | - | + |
| hsa-miR-320a | SET | - | - | - | + |
| hsa-miR-320a | SETBP1 | - | - | - | + |
| hsa-miR-320a | SETD1A | - | - | + | - |
| hsa-miR-320a | SETD3 | - | - | + | - |
| hsa-miR-320a | SETD8 | - | - | - | + |
| hsa-miR-320a | SETDB2 | - | - | - | + |
| hsa-miR-320a | SEZ6L | - | - | - | + |
| hsa-miR-320a | SF1 | - | - | + | - |
| hsa-miR-320a | SF3A3 | - | - | + | + |
| hsa-miR-320a | SF3B3 | - | - | + | + |
| hsa-miR-320a | SF3B6 | - | - | - | + |
| hsa-miR-320a | SFMBT1 | - | - | - | + |
| hsa-miR-320a | SFRP1 | - | - | - | + |
| hsa-miR-320a | SFRS14 | - | - | - | + |
| hsa-miR-320a | SFT2D1 | - | - | - | + |
| hsa-miR-320a | SFTA3 | + | - | - | - |
| hsa-miR-320a | SFXN1 | - | - | - | + |
| hsa-miR-320a | SGCB | - | + | - | + |
| hsa-miR-320a | SGCD | - | - | - | + |
| hsa-miR-320a | SGMS1 | - | - | - | + |
| hsa-miR-320a | SGMS2 | - | - | - | + |
| hsa-miR-320a | SGPL1 | - | - | - | + |
| hsa-miR-320a | SGPP1 | - | - | - | + |
| hsa-miR-320a | SGTB | - | - | - | + |
| hsa-miR-320a | SH2B3 | - | + | - | + |
| hsa-miR-320a | SH2D1A | - | - | - | + |
| hsa-miR-320a | SH2D1B | - | - | - | + |
| hsa-miR-320a | SH2D4B | - | - | - | + |
| hsa-miR-320a | SH3BGRL | - | - | - | + |
| hsa-miR-320a | SH3BGRL2 | - | + | - | + |
| hsa-miR-320a | SH3BP4 | - | - | - | + |
| hsa-miR-320a | SH3D19 | - | - | - | + |
| hsa-miR-320a | SH3GL1 | - | + | + | + |
| hsa-miR-320a | SH3GL2 | - | - | - | + |
| hsa-miR-320a | SH3KBP1 | - | - | - | + |
| hsa-miR-320a | SH3PXD2A | - | - | - | + |
| hsa-miR-320a | SH3PXD2B | - | - | - | + |
| hsa-miR-320a | SH3RF1 | - | - | - | + |
| hsa-miR-320a | SHC4 | - | - | - | + |
| hsa-miR-320a | SHCBP1 | + | + | - | + |
| hsa-miR-320a | SHISA2 | - | - | - | + |
| hsa-miR-320a | SHISA7 | - | + | - | + |
| hsa-miR-320a | SHISA9 | - | - | - | + |
| hsa-miR-320a | SHOC2 | - | - | - | + |
| hsa-miR-320a | SHPK | - | - | - | + |
| hsa-miR-320a | SHROOM2 | - | - | - | + |
| hsa-miR-320a | SHROOM3 | - | - | - | + |
| hsa-miR-320a | SHROOM4 | - | - | - | + |
| hsa-miR-320a | SHTN1 | - | + | - | - |
| hsa-miR-320a | SI | - | - | - | + |
| hsa-miR-320a | SIAH1 | - | - | - | + |
| hsa-miR-320a | SIAH2 | - | - | - | + |
| hsa-miR-320a | SIDT1 | - | - | - | + |
| hsa-miR-320a | SIGLECL1 | - | - | - | + |
| hsa-miR-320a | SIK1 | - | - | - | + |
| hsa-miR-320a | SIKE1 | - | - | - | + |
| hsa-miR-320a | SIM1 | - | - | - | + |
| hsa-miR-320a | SIPA1L1 | - | - | - | + |
| hsa-miR-320a | SIPA1L2 | - | - | - | + |
| hsa-miR-320a | SIRPA | - | - | - | + |
| hsa-miR-320a | SIRPD | - | - | - | + |
| hsa-miR-320a | SIRT4 | - | - | - | + |
| hsa-miR-320a | SIRT5 | - | - | - | + |
| hsa-miR-320a | SIRT6 | - | - | - | + |
| hsa-miR-320a | SIX2 | - | - | - | + |
| hsa-miR-320a | SIX3 | - | - | - | + |
| hsa-miR-320a | SIX4 | - | - | + | - |
| hsa-miR-320a | SKA1 | - | - | - | + |
| hsa-miR-320a | SKA2 | - | - | - | + |
| hsa-miR-320a | SKAP2 | - | - | - | + |
| hsa-miR-320a | SKI | - | - | - | + |
| hsa-miR-320a | SKIDA1 | - | - | - | + |
| hsa-miR-320a | SKP2 | - | - | - | + |
| hsa-miR-320a | SLC10A2 | - | - | - | + |
| hsa-miR-320a | SLC10A3 | + | + | - | + |
| hsa-miR-320a | SLC10A7 | + | - | - | + |
| hsa-miR-320a | SLC11A2 | - | - | - | + |
| hsa-miR-320a | SLC12A1 | - | - | - | + |
| hsa-miR-320a | SLC12A2 | - | - | - | + |
| hsa-miR-320a | SLC12A3 | - | - | - | + |
| hsa-miR-320a | SLC12A4 | - | - | - | + |
| hsa-miR-320a | SLC15A2 | - | - | - | + |
| hsa-miR-320a | SLC15A5 | - | - | - | + |
| hsa-miR-320a | SLC16A1 | - | + | + | + |
| hsa-miR-320a | SLC16A10 | - | - | - | + |
| hsa-miR-320a | SLC16A14 | - | + | - | + |
| hsa-miR-320a | SLC16A2 | - | - | - | + |
| hsa-miR-320a | SLC16A6 | - | - | - | + |
| hsa-miR-320a | SLC17A4 | - | - | - | + |
| hsa-miR-320a | SLC17A5 | - | - | - | + |
| hsa-miR-320a | SLC17A6 | - | - | - | + |
| hsa-miR-320a | SLC17A7 | - | - | - | + |
| hsa-miR-320a | SLC18A1 | - | - | - | + |
| hsa-miR-320a | SLC18A2 | - | - | - | + |
| hsa-miR-320a | SLC18B1 | - | - | - | + |
| hsa-miR-320a | SLC1A2 | - | - | - | + |
| hsa-miR-320a | SLC1A4 | - | - | - | + |
| hsa-miR-320a | SLC20A1 | - | - | - | + |
| hsa-miR-320a | SLC20A2 | - | - | - | + |
| hsa-miR-320a | SLC22A12 | - | - | - | + |
| hsa-miR-320a | SLC22A15 | - | - | - | + |
| hsa-miR-320a | SLC22A23 | - | - | - | + |
| hsa-miR-320a | SLC22A7 | - | - | - | + |
| hsa-miR-320a | SLC24A3 | - | - | - | + |
| hsa-miR-320a | SLC25A12 | - | - | + | - |
| hsa-miR-320a | SLC25A13 | - | - | - | + |
| hsa-miR-320a | SLC25A14 | - | - | - | + |
| hsa-miR-320a | SLC25A30 | - | - | - | + |
| hsa-miR-320a | SLC25A36 | - | + | - | + |
| hsa-miR-320a | SLC25A38 | - | - | - | + |
| hsa-miR-320a | SLC25A40 | - | - | - | + |
| hsa-miR-320a | SLC25A44 | - | - | - | + |
| hsa-miR-320a | SLC25A51 | + | - | - | - |
| hsa-miR-320a | SLC25A53 | - | + | - | - |
| hsa-miR-320a | SLC26A2 | - | - | - | + |
| hsa-miR-320a | SLC26A3 | + | - | - | - |
| hsa-miR-320a | SLC26A7 | - | - | - | + |
| hsa-miR-320a | SLC26A9 | - | - | - | + |
| hsa-miR-320a | SLC28A3 | - | - | - | + |
| hsa-miR-320a | SLC2A1 | - | - | + | - |
| hsa-miR-320a | SLC2A10 | - | - | - | + |
| hsa-miR-320a | SLC2A12 | - | + | - | - |
| hsa-miR-320a | SLC2A13 | - | - | - | + |
| hsa-miR-320a | SLC2A14 | - | - | - | + |
| hsa-miR-320a | SLC2A3 | - | - | - | + |
| hsa-miR-320a | SLC30A6 | - | - | - | + |
| hsa-miR-320a | SLC30A7 | - | - | - | + |
| hsa-miR-320a | SLC30A8 | - | - | - | + |
| hsa-miR-320a | SLC31A1 | - | - | - | + |
| hsa-miR-320a | SLC35A3 | - | - | - | + |
| hsa-miR-320a | SLC35B4 | - | - | - | + |
| hsa-miR-320a | SLC35C1 | - | - | - | + |
| hsa-miR-320a | SLC35C2 | - | - | - | + |
| hsa-miR-320a | SLC35D1 | - | - | - | + |
| hsa-miR-320a | SLC35F1 | - | - | - | + |
| hsa-miR-320a | SLC35F3 | - | - | - | + |
| hsa-miR-320a | SLC36A1 | - | - | - | + |
| hsa-miR-320a | SLC36A4 | - | - | - | + |
| hsa-miR-320a | SLC38A2 | - | - | + | - |
| hsa-miR-320a | SLC39A14 | - | - | - | + |
| hsa-miR-320a | SLC39A6 | - | - | - | + |
| hsa-miR-320a | SLC39A8 | - | + | - | - |
| hsa-miR-320a | SLC41A2 | - | - | - | + |
| hsa-miR-320a | SLC44A1 | - | - | - | + |
| hsa-miR-320a | SLC45A4 | - | - | - | + |
| hsa-miR-320a | SLC46A2 | + | - | - | + |
| hsa-miR-320a | SLC46A3 | - | - | - | + |
| hsa-miR-320a | SLC47A1 | - | - | - | + |
| hsa-miR-320a | SLC48A1 | - | - | - | + |
| hsa-miR-320a | SLC4A4 | - | - | - | + |
| hsa-miR-320a | SLC4A7 | - | - | - | + |
| hsa-miR-320a | SLC50A1 | + | - | - | + |
| hsa-miR-320a | SLC5A12 | - | - | - | + |
| hsa-miR-320a | SLC5A3 | - | + | - | + |
| hsa-miR-320a | SLC6A1 | - | - | - | + |
| hsa-miR-320a | SLC6A17 | - | - | - | + |
| hsa-miR-320a | SLC6A2 | - | - | - | + |
| hsa-miR-320a | SLC6A20 | - | - | - | + |
| hsa-miR-320a | SLC6A8 | - | - | + | + |
| hsa-miR-320a | SLC7A11 | - | + | - | + |
| hsa-miR-320a | SLC7A2 | - | - | - | + |
| hsa-miR-320a | SLC7A6 | - | - | - | + |
| hsa-miR-320a | SLC9A2 | - | + | - | + |
| hsa-miR-320a | SLC9A4 | - | - | - | + |
| hsa-miR-320a | SLC9A6 | - | - | - | + |
| hsa-miR-320a | SLC9A9 | - | - | - | + |
| hsa-miR-320a | SLCO1A2 | - | - | - | + |
| hsa-miR-320a | SLCO4C1 | - | - | - | + |
| hsa-miR-320a | SLFN11 | - | - | - | + |
| hsa-miR-320a | SLIT3 | - | - | - | + |
| hsa-miR-320a | SLITRK1 | - | - | - | + |
| hsa-miR-320a | SLITRK3 | - | - | - | + |
| hsa-miR-320a | SLITRK6 | - | - | - | + |
| hsa-miR-320a | SLK | - | - | - | + |
| hsa-miR-320a | SLMO2 | - | - | - | + |
| hsa-miR-320a | SLU7 | - | - | - | + |
| hsa-miR-320a | SMAD2 | - | - | - | + |
| hsa-miR-320a | SMAD3 | - | - | - | + |
| hsa-miR-320a | SMAD5 | - | - | - | + |
| hsa-miR-320a | SMAD7 | - | - | - | + |
| hsa-miR-320a | SMAD9 | + | + | - | - |
| hsa-miR-320a | SMAP1 | - | - | - | + |
| hsa-miR-320a | SMAP2 | - | - | - | + |
| hsa-miR-320a | SMARCA1 | - | - | - | + |
| hsa-miR-320a | SMARCA2 | - | - | - | + |
| hsa-miR-320a | SMARCA5 | - | - | - | + |
| hsa-miR-320a | SMARCAD1 | - | - | + | - |
| hsa-miR-320a | SMARCAL1 | - | - | - | + |
| hsa-miR-320a | SMARCC1 | - | - | - | + |
| hsa-miR-320a | SMARCC2 | - | - | + | - |
| hsa-miR-320a | SMARCD2 | - | + | - | + |
| hsa-miR-320a | SMC1A | - | - | + | + |
| hsa-miR-320a | SMC1B | - | - | - | + |
| hsa-miR-320a | SMC3 | - | - | - | + |
| hsa-miR-320a | SMC5 | - | - | - | + |
| hsa-miR-320a | SMC6 | - | - | - | + |
| hsa-miR-320a | SMCR8 | - | + | - | + |
| hsa-miR-320a | SMG1 | - | - | - | + |
| hsa-miR-320a | SMG7 | - | + | - | + |
| hsa-miR-320a | SMIM19 | + | - | - | - |
| hsa-miR-320a | SMNDC1 | + | + | - | - |
| hsa-miR-320a | SMOC1 | - | - | - | + |
| hsa-miR-320a | SMOX | - | - | - | + |
| hsa-miR-320a | SMPD3 | - | - | - | + |
| hsa-miR-320a | SMPDL3A | - | - | - | + |
| hsa-miR-320a | SMS | - | - | - | + |
| hsa-miR-320a | SMU1 | - | - | - | + |
| hsa-miR-320a | SMUG1 | - | - | + | - |
| hsa-miR-320a | SMURF1 | - | - | - | + |
| hsa-miR-320a | SNAP23 | - | - | - | + |
| hsa-miR-320a | SNAP25 | - | - | - | + |
| hsa-miR-320a | SND1 | - | - | - | + |
| hsa-miR-320a | SNED1 | - | - | - | + |
| hsa-miR-320a | SNIP1 | - | - | - | + |
| hsa-miR-320a | SNRNP48 | - | - | + | + |
| hsa-miR-320a | SNRPB | - | - | + | - |
| hsa-miR-320a | SNRPD2 | - | - | + | - |
| hsa-miR-320a | SNRPG | - | - | + | - |
| hsa-miR-320a | SNRPN | - | - | - | + |
| hsa-miR-320a | SNTB1 | + | + | - | + |
| hsa-miR-320a | SNTB2 | - | - | - | + |
| hsa-miR-320a | SNTG1 | - | - | - | + |
| hsa-miR-320a | SNX11 | - | - | - | + |
| hsa-miR-320a | SNX12 | - | - | - | + |
| hsa-miR-320a | SNX16 | - | + | - | + |
| hsa-miR-320a | SNX18 | - | - | - | + |
| hsa-miR-320a | SNX20 | - | - | - | + |
| hsa-miR-320a | SNX24 | - | - | - | + |
| hsa-miR-320a | SNX3 | - | - | - | + |
| hsa-miR-320a | SNX30 | - | - | - | + |
| hsa-miR-320a | SNX31 | - | - | - | + |
| hsa-miR-320a | SNX4 | + | - | - | - |
| hsa-miR-320a | SOAT1 | + | + | - | + |
| hsa-miR-320a | SOBP | - | - | - | + |
| hsa-miR-320a | SOCS4 | - | - | - | + |
| hsa-miR-320a | SOCS5 | - | - | - | + |
| hsa-miR-320a | SOCS6 | - | - | - | + |
| hsa-miR-320a | SOGA1 | - | + | - | - |
| hsa-miR-320a | SOGA3 | - | - | - | + |
| hsa-miR-320a | SOLH | - | - | + | - |
| hsa-miR-320a | SON | - | - | - | + |
| hsa-miR-320a | SORBS1 | - | - | + | - |
| hsa-miR-320a | SORBS2 | - | - | - | + |
| hsa-miR-320a | SORCS2 | - | + | - | + |
| hsa-miR-320a | SORCS3 | - | - | - | + |
| hsa-miR-320a | SORL1 | - | - | - | + |
| hsa-miR-320a | SORT1 | - | - | - | + |
| hsa-miR-320a | SOS2 | - | - | - | + |
| hsa-miR-320a | SOSTDC1 | - | - | - | + |
| hsa-miR-320a | SOWAHA | - | + | - | + |
| hsa-miR-320a | SOX11 | - | - | - | + |
| hsa-miR-320a | SOX12 | - | - | - | + |
| hsa-miR-320a | SOX2 | - | - | - | + |
| hsa-miR-320a | SOX3 | - | - | - | + |
| hsa-miR-320a | SOX4 | - | - | - | + |
| hsa-miR-320a | SOX6 | - | - | - | + |
| hsa-miR-320a | SOX8 | - | - | - | + |
| hsa-miR-320a | SOX9 | - | - | - | + |
| hsa-miR-320a | SP1 | - | - | - | + |
| hsa-miR-320a | SP100 | - | + | - | - |
| hsa-miR-320a | SP2 | - | - | - | + |
| hsa-miR-320a | SP5 | - | - | - | + |
| hsa-miR-320a | SPAG1 | - | - | - | + |
| hsa-miR-320a | SPAG9 | - | - | - | + |
| hsa-miR-320a | SPARC | - | - | - | + |
| hsa-miR-320a | SPATA13 | - | - | - | + |
| hsa-miR-320a | SPATA18 | - | + | - | + |
| hsa-miR-320a | SPATA5 | - | - | - | + |
| hsa-miR-320a | SPATA6 | - | + | - | + |
| hsa-miR-320a | SPATS2L | - | - | - | + |
| hsa-miR-320a | SPC25 | - | - | - | + |
| hsa-miR-320a | SPCS2 | - | - | - | + |
| hsa-miR-320a | SPCS3 | - | - | - | + |
| hsa-miR-320a | SPEN | - | - | + | - |
| hsa-miR-320a | SPG11 | - | - | - | + |
| hsa-miR-320a | SPG20 | - | - | - | + |
| hsa-miR-320a | SPIC | - | - | - | + |
| hsa-miR-320a | SPIN4 | - | - | - | + |
| hsa-miR-320a | SPINK6 | + | - | - | - |
| hsa-miR-320a | SPINT2 | - | - | - | + |
| hsa-miR-320a | SPIRE1 | - | - | - | + |
| hsa-miR-320a | SPOCK1 | - | - | - | + |
| hsa-miR-320a | SPOCK2 | - | - | - | + |
| hsa-miR-320a | SPOP | - | - | - | + |
| hsa-miR-320a | SPOPL | + | + | - | + |
| hsa-miR-320a | SPPL2B | - | + | - | - |
| hsa-miR-320a | SPRED1 | - | - | - | + |
| hsa-miR-320a | SPRED2 | - | - | - | + |
| hsa-miR-320a | SPRR1B | - | - | - | + |
| hsa-miR-320a | SPRR3 | - | - | - | + |
| hsa-miR-320a | SPRY1 | - | - | - | + |
| hsa-miR-320a | SPRY2 | - | - | - | + |
| hsa-miR-320a | SPRY3 | - | - | + | + |
| hsa-miR-320a | SPRY4 | - | - | - | + |
| hsa-miR-320a | SPRYD4 | + | + | + | - |
| hsa-miR-320a | SPTBN1 | - | - | - | + |
| hsa-miR-320a | SPTLC2 | - | - | - | + |
| hsa-miR-320a | SPTSSA | - | + | - | + |
| hsa-miR-320a | SRCAP | - | - | + | - |
| hsa-miR-320a | SREK1 | - | + | - | + |
| hsa-miR-320a | SREK1IP1 | - | - | - | + |
| hsa-miR-320a | SRF | - | - | + | + |
| hsa-miR-320a | SRGAP2 | - | + | - | - |
| hsa-miR-320a | SRI | - | - | - | + |
| hsa-miR-320a | SRM | - | - | + | - |
| hsa-miR-320a | SRP19 | - | + | - | - |
| hsa-miR-320a | SRP68 | - | - | + | - |
| hsa-miR-320a | SRPK1 | - | - | - | + |
| hsa-miR-320a | SRPK2 | - | - | - | + |
| hsa-miR-320a | SRPR | - | - | - | + |
| hsa-miR-320a | SRPX | - | - | - | + |
| hsa-miR-320a | SRRM1 | - | - | - | + |
| hsa-miR-320a | SRRM2 | - | - | + | - |
| hsa-miR-320a | SRRM4 | - | - | - | + |
| hsa-miR-320a | SRSF2 | - | - | - | + |
| hsa-miR-320a | SRSF7 | - | - | + | - |
| hsa-miR-320a | SRXN1 | - | - | - | + |
| hsa-miR-320a | SS18 | - | - | - | + |
| hsa-miR-320a | SSH2 | - | - | - | + |
| hsa-miR-320a | SSR1 | - | - | - | + |
| hsa-miR-320a | SSSCA1 | - | - | + | - |
| hsa-miR-320a | SSTR1 | - | - | - | + |
| hsa-miR-320a | SSX2IP | - | - | - | + |
| hsa-miR-320a | SSX7 | - | - | - | + |
| hsa-miR-320a | ST13 | - | - | - | + |
| hsa-miR-320a | ST3GAL1 | - | + | - | + |
| hsa-miR-320a | ST5 | - | - | - | + |
| hsa-miR-320a | ST6GAL2 | - | - | - | + |
| hsa-miR-320a | ST6GALNAC2 | - | - | - | + |
| hsa-miR-320a | ST6GALNAC3 | - | - | - | + |
| hsa-miR-320a | ST7-OT4 | + | - | - | - |
| hsa-miR-320a | STAG2 | - | + | - | + |
| hsa-miR-320a | STAMBP | - | - | - | + |
| hsa-miR-320a | STARD4 | + | - | - | + |
| hsa-miR-320a | STARD7 | - | - | - | + |
| hsa-miR-320a | STAT1 | - | - | - | + |
| hsa-miR-320a | STAT3 | - | - | - | + |
| hsa-miR-320a | STAT4 | + | + | - | - |
| hsa-miR-320a | STAT5B | - | - | - | + |
| hsa-miR-320a | STAU1 | - | - | - | + |
| hsa-miR-320a | STC1 | - | - | - | + |
| hsa-miR-320a | STEAP2 | - | - | - | + |
| hsa-miR-320a | STIL | - | - | - | + |
| hsa-miR-320a | STIM2 | - | - | - | + |
| hsa-miR-320a | STK17A | - | - | - | + |
| hsa-miR-320a | STK17B | - | - | - | + |
| hsa-miR-320a | STK26 | - | + | - | + |
| hsa-miR-320a | STK32A | - | - | - | + |
| hsa-miR-320a | STK32B | - | + | - | + |
| hsa-miR-320a | STK35 | - | - | - | + |
| hsa-miR-320a | STK38 | - | - | - | + |
| hsa-miR-320a | STK38L | - | - | - | + |
| hsa-miR-320a | STK4 | - | - | - | + |
| hsa-miR-320a | STMN4 | - | - | - | + |
| hsa-miR-320a | STOM | - | - | - | + |
| hsa-miR-320a | STOML2 | - | - | + | - |
| hsa-miR-320a | STOX1 | - | - | - | + |
| hsa-miR-320a | STRADB | - | - | - | + |
| hsa-miR-320a | STRBP | - | - | - | + |
| hsa-miR-320a | STRN | - | - | - | + |
| hsa-miR-320a | STRN3 | - | - | - | + |
| hsa-miR-320a | STS | - | - | - | + |
| hsa-miR-320a | STUB1 | - | - | - | + |
| hsa-miR-320a | STX12 | - | - | - | + |
| hsa-miR-320a | STX16 | - | - | - | + |
| hsa-miR-320a | STX2 | - | - | - | + |
| hsa-miR-320a | STX6 | - | - | - | + |
| hsa-miR-320a | STYK1 | - | - | - | + |
| hsa-miR-320a | STYX | - | - | - | + |
| hsa-miR-320a | SUB1 | - | + | - | + |
| hsa-miR-320a | SUCLA2 | - | - | - | + |
| hsa-miR-320a | SUFU | - | - | - | + |
| hsa-miR-320a | SULF1 | - | - | - | + |
| hsa-miR-320a | SULT1C2 | - | - | - | + |
| hsa-miR-320a | SUMO3 | - | - | - | + |
| hsa-miR-320a | SUPT16H | - | - | - | + |
| hsa-miR-320a | SUPT7L | - | - | - | + |
| hsa-miR-320a | SUPV3L1 | - | - | + | - |
| hsa-miR-320a | SURF4 | - | - | - | + |
| hsa-miR-320a | SUSD6 | - | + | - | + |
| hsa-miR-320a | SUV420H1 | - | + | - | + |
| hsa-miR-320a | SUZ12 | - | - | - | + |
| hsa-miR-320a | SV2A | - | - | - | + |
| hsa-miR-320a | SV2B | - | + | - | + |
| hsa-miR-320a | SWAP70 | - | - | + | + |
| hsa-miR-320a | SWSAP1 | - | - | - | + |
| hsa-miR-320a | SWT1 | - | - | - | + |
| hsa-miR-320a | SYCP2 | - | - | - | + |
| hsa-miR-320a | SYDE1 | - | - | - | + |
| hsa-miR-320a | SYDE2 | - | - | - | + |
| hsa-miR-320a | SYF2 | - | - | + | - |
| hsa-miR-320a | SYK | - | - | - | + |
| hsa-miR-320a | SYN3 | - | - | - | + |
| hsa-miR-320a | SYNCRIP | - | + | + | + |
| hsa-miR-320a | SYNDIG1 | - | - | - | + |
| hsa-miR-320a | SYNGR2 | + | + | + | - |
| hsa-miR-320a | SYNGR3 | - | - | - | + |
| hsa-miR-320a | SYNJ1 | - | - | - | + |
| hsa-miR-320a | SYNJ2BP | - | - | + | - |
| hsa-miR-320a | SYNM | - | - | + | - |
| hsa-miR-320a | SYPL1 | - | - | - | + |
| hsa-miR-320a | SYS1 | - | - | - | + |
| hsa-miR-320a | SYT1 | - | - | - | + |
| hsa-miR-320a | SYT11 | - | - | - | + |
| hsa-miR-320a | SYT13 | - | - | - | + |
| hsa-miR-320a | SYT2 | - | - | - | + |
| hsa-miR-320a | SYT4 | - | - | - | + |
| hsa-miR-320a | SYT6 | - | - | - | + |
| hsa-miR-320a | SYTL4 | + | - | - | - |
| hsa-miR-320a | TAC1 | - | - | + | - |
| hsa-miR-320a | TACC1 | - | + | - | - |
| hsa-miR-320a | TADA1 | - | - | - | + |
| hsa-miR-320a | TAF1 | - | - | - | + |
| hsa-miR-320a | TAF12 | - | - | - | + |
| hsa-miR-320a | TAF15 | - | - | + | - |
| hsa-miR-320a | TAF2 | - | - | - | + |
| hsa-miR-320a | TAF5 | - | + | - | + |
| hsa-miR-320a | TAF5L | - | - | - | + |
| hsa-miR-320a | TAF7 | - | - | - | + |
| hsa-miR-320a | TAF7L | - | - | - | + |
| hsa-miR-320a | TAF9B | - | - | - | + |
| hsa-miR-320a | TAGAP | - | - | - | + |
| hsa-miR-320a | TAL1 | - | - | + | + |
| hsa-miR-320a | TANC1 | - | - | - | + |
| hsa-miR-320a | TANC2 | - | + | - | + |
| hsa-miR-320a | TAOK1 | - | - | - | + |
| hsa-miR-320a | TAOK2 | - | - | - | + |
| hsa-miR-320a | TAOK3 | - | - | - | + |
| hsa-miR-320a | TAP2 | - | - | - | + |
| hsa-miR-320a | TAPBP | - | - | - | + |
| hsa-miR-320a | TASP1 | - | - | - | + |
| hsa-miR-320a | TAX1BP3 | - | - | - | + |
| hsa-miR-320a | TBC1D1 | - | - | - | + |
| hsa-miR-320a | TBC1D12 | - | - | - | + |
| hsa-miR-320a | TBC1D14 | - | - | + | - |
| hsa-miR-320a | TBC1D15 | - | - | - | + |
| hsa-miR-320a | TBC1D20 | - | - | - | + |
| hsa-miR-320a | TBC1D22B | - | - | - | + |
| hsa-miR-320a | TBC1D24 | - | - | - | + |
| hsa-miR-320a | TBC1D26 | - | - | - | + |
| hsa-miR-320a | TBC1D30 | - | - | - | + |
| hsa-miR-320a | TBC1D4 | - | - | - | + |
| hsa-miR-320a | TBC1D9 | - | - | - | + |
| hsa-miR-320a | TBCA | - | - | - | + |
| hsa-miR-320a | TBCCD1 | - | - | - | + |
| hsa-miR-320a | TBL1X | - | - | - | + |
| hsa-miR-320a | TBL1XR1 | - | + | - | + |
| hsa-miR-320a | TBX15 | - | - | - | + |
| hsa-miR-320a | TBX18 | - | + | - | + |
| hsa-miR-320a | TBX21 | - | - | - | + |
| hsa-miR-320a | TBX3 | - | - | - | + |
| hsa-miR-320a | TBX4 | - | - | - | + |
| hsa-miR-320a | TBX5 | - | - | - | + |
| hsa-miR-320a | TC2N | - | - | - | + |
| hsa-miR-320a | TCEA1 | - | - | - | + |
| hsa-miR-320a | TCEAL8 | + | - | - | + |
| hsa-miR-320a | TCEB1 | - | - | - | + |
| hsa-miR-320a | TCERG1 | - | - | - | + |
| hsa-miR-320a | TCF12 | - | - | - | + |
| hsa-miR-320a | TCF19 | - | - | - | + |
| hsa-miR-320a | TCF3 | - | - | + | + |
| hsa-miR-320a | TCF7L1 | - | - | - | + |
| hsa-miR-320a | TCF7L2 | - | - | - | + |
| hsa-miR-320a | TCHHL1 | - | - | - | + |
| hsa-miR-320a | TCP1 | - | - | + | - |
| hsa-miR-320a | TCP10L2 | - | - | - | + |
| hsa-miR-320a | TCTEX1D1 | - | - | - | + |
| hsa-miR-320a | TDG | + | + | - | + |
| hsa-miR-320a | TDP1 | - | + | + | + |
| hsa-miR-320a | TDRD6 | - | - | - | + |
| hsa-miR-320a | TEAD1 | - | - | + | - |
| hsa-miR-320a | TECPR2 | - | - | - | + |
| hsa-miR-320a | TECTB | - | - | - | + |
| hsa-miR-320a | TEKT1 | - | - | - | + |
| hsa-miR-320a | TENM1 | - | - | - | + |
| hsa-miR-320a | TES | - | - | - | + |
| hsa-miR-320a | TESPA1 | - | - | - | + |
| hsa-miR-320a | TET1 | - | - | - | + |
| hsa-miR-320a | TET3 | - | - | - | + |
| hsa-miR-320a | TEX11 | - | - | - | + |
| hsa-miR-320a | TEX2 | - | - | - | + |
| hsa-miR-320a | TEX261 | - | - | - | + |
| hsa-miR-320a | TFAP2A | - | - | + | - |
| hsa-miR-320a | TFAP2C | - | - | - | + |
| hsa-miR-320a | TFB2M | - | - | - | + |
| hsa-miR-320a | TFCP2 | - | - | - | + |
| hsa-miR-320a | TFCP2L1 | - | + | - | + |
| hsa-miR-320a | TFDP2 | - | - | - | + |
| hsa-miR-320a | TFE3 | - | - | - | + |
| hsa-miR-320a | TFEC | - | - | - | + |
| hsa-miR-320a | TFF3 | + | - | - | + |
| hsa-miR-320a | TFPI | - | + | - | + |
| hsa-miR-320a | TFRC | + | - | + | - |
| hsa-miR-320a | TGDS | - | - | - | + |
| hsa-miR-320a | TGFA | - | - | - | + |
| hsa-miR-320a | TGFB2 | - | - | - | + |
| hsa-miR-320a | TGFBR1 | - | - | - | + |
| hsa-miR-320a | TGFBR2 | - | - | - | + |
| hsa-miR-320a | TGM4 | - | - | - | + |
| hsa-miR-320a | TGOLN2 | + | + | - | + |
| hsa-miR-320a | THAP2 | - | - | - | + |
| hsa-miR-320a | THAP5 | - | - | - | + |
| hsa-miR-320a | THAP6 | - | - | - | + |
| hsa-miR-320a | THBD | - | - | - | + |
| hsa-miR-320a | THBS2 | - | - | - | + |
| hsa-miR-320a | THBS4 | - | - | - | + |
| hsa-miR-320a | THEG | - | - | - | + |
| hsa-miR-320a | THRB | - | - | - | + |
| hsa-miR-320a | THRSP | - | - | - | + |
| hsa-miR-320a | THSD4 | - | + | - | + |
| hsa-miR-320a | THSD7B | - | - | - | + |
| hsa-miR-320a | THUMPD1 | - | - | - | + |
| hsa-miR-320a | THUMPD3 | - | - | - | + |
| hsa-miR-320a | THYN1 | - | - | - | + |
| hsa-miR-320a | TIAM1 | - | - | - | + |
| hsa-miR-320a | TIGAR | - | + | - | - |
| hsa-miR-320a | TIGD4 | - | - | - | + |
| hsa-miR-320a | TIMELESS | - | - | - | + |
| hsa-miR-320a | TIMM8B | + | - | - | - |
| hsa-miR-320a | TIPARP | - | - | - | + |
| hsa-miR-320a | TJAP1 | - | - | + | - |
| hsa-miR-320a | TJP1 | - | - | - | + |
| hsa-miR-320a | TJP2 | - | - | - | + |
| hsa-miR-320a | TK2 | - | + | - | + |
| hsa-miR-320a | TKTL2 | - | - | - | + |
| hsa-miR-320a | TLE3 | - | - | - | + |
| hsa-miR-320a | TLK2 | - | + | - | - |
| hsa-miR-320a | TLL2 | - | - | - | + |
| hsa-miR-320a | TLN2 | - | - | - | + |
| hsa-miR-320a | TLR5 | - | - | - | + |
| hsa-miR-320a | TLR6 | - | - | - | + |
| hsa-miR-320a | TLR8 | - | - | - | + |
| hsa-miR-320a | TLX3 | - | - | - | + |
| hsa-miR-320a | TM4SF4 | + | - | - | - |
| hsa-miR-320a | TM9SF2 | - | - | - | + |
| hsa-miR-320a | TM9SF3 | - | - | - | + |
| hsa-miR-320a | TMA16 | - | - | - | + |
| hsa-miR-320a | TMBIM6 | - | - | - | + |
| hsa-miR-320a | TMC1 | - | - | - | + |
| hsa-miR-320a | TMC7 | - | - | - | + |
| hsa-miR-320a | TMCC3 | - | - | - | + |
| hsa-miR-320a | TMCO3 | - | - | - | + |
| hsa-miR-320a | TMCO6 | - | - | + | - |
| hsa-miR-320a | TMED10 | - | - | - | + |
| hsa-miR-320a | TMED5 | - | - | - | + |
| hsa-miR-320a | TMED6 | + | - | - | + |
| hsa-miR-320a | TMED7 | - | - | - | + |
| hsa-miR-320a | TMEM100 | + | + | - | + |
| hsa-miR-320a | TMEM101 | - | - | + | - |
| hsa-miR-320a | TMEM106B | + | + | - | - |
| hsa-miR-320a | TMEM106C | - | - | - | + |
| hsa-miR-320a | TMEM108 | - | + | - | - |
| hsa-miR-320a | TMEM110 | - | - | - | + |
| hsa-miR-320a | TMEM120B | - | - | - | + |
| hsa-miR-320a | TMEM123 | - | + | - | - |
| hsa-miR-320a | TMEM128 | - | + | - | + |
| hsa-miR-320a | TMEM131 | - | - | - | + |
| hsa-miR-320a | TMEM132B | - | - | - | + |
| hsa-miR-320a | TMEM132C | - | - | - | + |
| hsa-miR-320a | TMEM136 | - | + | - | + |
| hsa-miR-320a | TMEM139 | - | - | - | + |
| hsa-miR-320a | TMEM144 | + | - | - | - |
| hsa-miR-320a | TMEM14A | - | - | - | + |
| hsa-miR-320a | TMEM14C | - | - | - | + |
| hsa-miR-320a | TMEM150C | - | - | - | + |
| hsa-miR-320a | TMEM158 | - | - | - | + |
| hsa-miR-320a | TMEM163 | - | - | - | + |
| hsa-miR-320a | TMEM167A | - | - | - | + |
| hsa-miR-320a | TMEM170A | - | - | - | + |
| hsa-miR-320a | TMEM170B | - | - | - | + |
| hsa-miR-320a | TMEM178B | - | - | - | + |
| hsa-miR-320a | TMEM182 | - | - | - | + |
| hsa-miR-320a | TMEM189-UBE2V1 | - | - | - | + |
| hsa-miR-320a | TMEM19 | - | - | - | + |
| hsa-miR-320a | TMEM194A | - | - | - | + |
| hsa-miR-320a | TMEM199 | - | - | - | + |
| hsa-miR-320a | TMEM2 | - | + | - | - |
| hsa-miR-320a | TMEM204 | - | - | - | + |
| hsa-miR-320a | TMEM220 | - | - | - | + |
| hsa-miR-320a | TMEM229B | - | - | - | + |
| hsa-miR-320a | TMEM230 | + | - | - | + |
| hsa-miR-320a | TMEM231 | - | - | - | + |
| hsa-miR-320a | TMEM236 | - | - | - | + |
| hsa-miR-320a | TMEM237 | - | - | - | + |
| hsa-miR-320a | TMEM245 | - | + | - | + |
| hsa-miR-320a | TMEM246 | - | - | - | + |
| hsa-miR-320a | TMEM248 | - | - | - | + |
| hsa-miR-320a | TMEM254 | - | + | - | + |
| hsa-miR-320a | TMEM255A | - | + | - | + |
| hsa-miR-320a | TMEM26 | - | - | - | + |
| hsa-miR-320a | TMEM263 | - | - | - | + |
| hsa-miR-320a | TMEM30A | - | - | - | + |
| hsa-miR-320a | TMEM30B | - | - | - | + |
| hsa-miR-320a | TMEM33 | - | - | - | + |
| hsa-miR-320a | TMEM35 | - | - | - | + |
| hsa-miR-320a | TMEM38B | + | - | - | + |
| hsa-miR-320a | TMEM43 | - | - | + | + |
| hsa-miR-320a | TMEM45B | - | - | - | + |
| hsa-miR-320a | TMEM47 | - | + | - | + |
| hsa-miR-320a | TMEM51 | - | - | + | + |
| hsa-miR-320a | TMEM55A | - | - | - | + |
| hsa-miR-320a | TMEM56 | - | - | - | + |
| hsa-miR-320a | TMEM63A | - | - | - | + |
| hsa-miR-320a | TMEM64 | + | - | + | + |
| hsa-miR-320a | TMEM67 | - | - | - | + |
| hsa-miR-320a | TMEM70 | - | - | - | + |
| hsa-miR-320a | TMEM72 | - | - | - | + |
| hsa-miR-320a | TMEM80 | + | - | - | - |
| hsa-miR-320a | TMEM81 | - | - | - | + |
| hsa-miR-320a | TMEM87B | - | - | - | + |
| hsa-miR-320a | TMEM97 | - | - | - | + |
| hsa-miR-320a | TMEM98 | + | - | + | - |
| hsa-miR-320a | TMEM9B | - | - | - | + |
| hsa-miR-320a | TMF1 | - | - | - | + |
| hsa-miR-320a | TMOD2 | - | - | - | + |
| hsa-miR-320a | TMPO | - | - | + | + |
| hsa-miR-320a | TMPPE | - | - | - | + |
| hsa-miR-320a | TMPRSS11F | - | - | - | + |
| hsa-miR-320a | TMPRSS13 | - | - | - | + |
| hsa-miR-320a | TMPRSS2 | - | - | - | + |
| hsa-miR-320a | TMSB15A | + | - | - | + |
| hsa-miR-320a | TMTC1 | - | + | + | + |
| hsa-miR-320a | TMTC3 | - | - | - | + |
| hsa-miR-320a | TMTC4 | - | - | - | + |
| hsa-miR-320a | TMX3 | - | - | - | + |
| hsa-miR-320a | TMX4 | - | - | - | + |
| hsa-miR-320a | TNF | - | - | - | + |
| hsa-miR-320a | TNFAIP2 | - | - | - | + |
| hsa-miR-320a | TNFRSF11B | - | - | - | + |
| hsa-miR-320a | TNFRSF17 | + | - | - | + |
| hsa-miR-320a | TNFRSF19 | - | - | - | + |
| hsa-miR-320a | TNFRSF21 | - | - | - | + |
| hsa-miR-320a | TNFRSF9 | - | + | - | - |
| hsa-miR-320a | TNFSF13B | - | - | - | + |
| hsa-miR-320a | TNFSF4 | - | - | - | + |
| hsa-miR-320a | TNIK | - | - | - | + |
| hsa-miR-320a | TNKS | - | - | - | + |
| hsa-miR-320a | TNKS2 | - | - | - | + |
| hsa-miR-320a | TNRC6A | - | - | + | - |
| hsa-miR-320a | TNRC6B | - | - | + | + |
| hsa-miR-320a | TNRC6C | - | + | + | + |
| hsa-miR-320a | TNS1 | - | + | - | + |
| hsa-miR-320a | TOB1 | - | - | - | + |
| hsa-miR-320a | TOB2 | - | - | + | - |
| hsa-miR-320a | TOE1 | - | - | + | - |
| hsa-miR-320a | TOMM34 | - | - | - | + |
| hsa-miR-320a | TOMM70A | - | + | - | + |
| hsa-miR-320a | TONSL | - | - | + | - |
| hsa-miR-320a | TOP1 | - | - | - | + |
| hsa-miR-320a | TOP2A | - | - | - | + |
| hsa-miR-320a | TOP3A | - | - | + | - |
| hsa-miR-320a | TOR1AIP2 | - | + | - | + |
| hsa-miR-320a | TOX | - | - | - | + |
| hsa-miR-320a | TOX3 | - | - | - | + |
| hsa-miR-320a | TP53INP1 | - | - | - | + |
| hsa-miR-320a | TP53RK | - | - | - | + |
| hsa-miR-320a | TP63 | - | - | - | + |
| hsa-miR-320a | TP73 | - | - | + | + |
| hsa-miR-320a | TPD52 | - | - | + | + |
| hsa-miR-320a | TPD52L2 | - | + | - | + |
| hsa-miR-320a | TPD52L3 | - | - | - | + |
| hsa-miR-320a | TPI1 | - | - | + | - |
| hsa-miR-320a | TPM1 | - | + | - | - |
| hsa-miR-320a | TPM3 | + | - | - | + |
| hsa-miR-320a | TPM4 | - | - | - | + |
| hsa-miR-320a | TPMT | - | - | - | + |
| hsa-miR-320a | TPR | - | + | - | - |
| hsa-miR-320a | TPRXL | - | - | - | + |
| hsa-miR-320a | TPST1 | - | - | - | + |
| hsa-miR-320a | TRAF3IP1 | - | - | - | + |
| hsa-miR-320a | TRAF3IP2 | - | - | - | + |
| hsa-miR-320a | TRAF4 | - | - | + | - |
| hsa-miR-320a | TRAF7 | - | - | - | + |
| hsa-miR-320a | TRAFD1 | - | - | - | + |
| hsa-miR-320a | TRAK1 | - | - | - | + |
| hsa-miR-320a | TRAK2 | - | - | - | + |
| hsa-miR-320a | TRAM1 | - | - | - | + |
| hsa-miR-320a | TRAM2 | - | - | - | + |
| hsa-miR-320a | TRAP1 | + | - | - | - |
| hsa-miR-320a | TRAPPC2 | - | - | - | + |
| hsa-miR-320a | TRAPPC6B | - | - | - | + |
| hsa-miR-320a | TRDN | - | + | - | - |
| hsa-miR-320a | TRERF1 | - | - | - | + |
| hsa-miR-320a | TRIAP1 | - | + | - | + |
| hsa-miR-320a | TRIB1 | - | - | - | + |
| hsa-miR-320a | TRIB2 | - | - | - | + |
| hsa-miR-320a | TRIM13 | - | - | - | + |
| hsa-miR-320a | TRIM14 | - | - | + | + |
| hsa-miR-320a | TRIM2 | - | - | - | + |
| hsa-miR-320a | TRIM23 | - | - | - | + |
| hsa-miR-320a | TRIM24 | - | + | - | - |
| hsa-miR-320a | TRIM25 | - | - | - | + |
| hsa-miR-320a | TRIM32 | - | - | - | + |
| hsa-miR-320a | TRIM33 | - | - | - | + |
| hsa-miR-320a | TRIM35 | - | - | - | + |
| hsa-miR-320a | TRIM36 | - | - | - | + |
| hsa-miR-320a | TRIM4 | - | - | - | + |
| hsa-miR-320a | TRIM41 | - | + | - | + |
| hsa-miR-320a | TRIM42 | - | - | - | + |
| hsa-miR-320a | TRIM5 | - | - | - | + |
| hsa-miR-320a | TRIM6 | - | - | - | + |
| hsa-miR-320a | TRIM6-TRIM34 | - | - | - | + |
| hsa-miR-320a | TRIM62 | - | - | - | + |
| hsa-miR-320a | TRIM63 | - | - | - | + |
| hsa-miR-320a | TRIM65 | - | - | - | + |
| hsa-miR-320a | TRIM67 | - | - | - | + |
| hsa-miR-320a | TRIM9 | - | - | - | + |
| hsa-miR-320a | TRIP11 | - | - | - | + |
| hsa-miR-320a | TRIP13 | - | - | - | + |
| hsa-miR-320a | TRMT10A | - | + | - | + |
| hsa-miR-320a | TRMT13 | - | - | - | + |
| hsa-miR-320a | TRMT5 | - | - | - | + |
| hsa-miR-320a | TRMT6 | - | - | - | + |
| hsa-miR-320a | TRMT61B | - | - | - | + |
| hsa-miR-320a | TROVE2 | + | + | - | + |
| hsa-miR-320a | TRPC1 | - | - | - | + |
| hsa-miR-320a | TRPC5 | - | - | + | + |
| hsa-miR-320a | TRPM7 | - | - | - | + |
| hsa-miR-320a | TRPS1 | - | - | - | + |
| hsa-miR-320a | TRRAP | + | - | - | - |
| hsa-miR-320a | TRUB1 | - | - | - | + |
| hsa-miR-320a | TSC1 | - | + | + | + |
| hsa-miR-320a | TSC22D2 | - | - | + | - |
| hsa-miR-320a | TSC22D3 | - | - | - | + |
| hsa-miR-320a | TSC22D4 | + | - | - | + |
| hsa-miR-320a | TSEN2 | - | - | - | + |
| hsa-miR-320a | TSFM | - | - | - | + |
| hsa-miR-320a | TSHZ1 | - | - | - | + |
| hsa-miR-320a | TSHZ2 | - | - | - | + |
| hsa-miR-320a | TSHZ3 | - | + | - | + |
| hsa-miR-320a | TSN | - | - | - | + |
| hsa-miR-320a | TSNAX | - | - | - | + |
| hsa-miR-320a | TSPAN11 | - | - | - | + |
| hsa-miR-320a | TSPAN12 | - | - | - | + |
| hsa-miR-320a | TSPAN18 | - | - | - | + |
| hsa-miR-320a | TSPAN2 | - | - | - | + |
| hsa-miR-320a | TSPAN9 | - | - | + | + |
| hsa-miR-320a | TSPYL2 | - | - | + | - |
| hsa-miR-320a | TSPYL5 | - | + | - | + |
| hsa-miR-320a | TSR1 | - | - | - | + |
| hsa-miR-320a | TSSK1B | - | - | - | + |
| hsa-miR-320a | TTBK2 | - | - | - | + |
| hsa-miR-320a | TTC14 | + | + | - | - |
| hsa-miR-320a | TTC17 | - | - | - | + |
| hsa-miR-320a | TTC21B | - | - | - | + |
| hsa-miR-320a | TTC26 | - | - | - | + |
| hsa-miR-320a | TTC28 | - | - | - | + |
| hsa-miR-320a | TTC3 | - | - | - | + |
| hsa-miR-320a | TTC8 | + | - | - | - |
| hsa-miR-320a | TTC9 | - | - | - | + |
| hsa-miR-320a | TTPA | - | - | - | + |
| hsa-miR-320a | TTPAL | - | - | - | + |
| hsa-miR-320a | TTYH3 | - | - | - | + |
| hsa-miR-320a | TUB | - | - | + | + |
| hsa-miR-320a | TUBA1B | - | - | + | - |
| hsa-miR-320a | TUBA4A | - | - | - | + |
| hsa-miR-320a | TUBA8 | - | - | - | + |
| hsa-miR-320a | TUBB | - | - | + | + |
| hsa-miR-320a | TUBB1 | - | - | - | + |
| hsa-miR-320a | TUBGCP2 | - | - | + | - |
| hsa-miR-320a | TULP4 | - | - | - | + |
| hsa-miR-320a | TUSC3 | + | + | - | + |
| hsa-miR-320a | TWF1 | - | + | - | - |
| hsa-miR-320a | TXLNA | - | - | - | + |
| hsa-miR-320a | TXLNG | - | - | - | + |
| hsa-miR-320a | TXNDC16 | - | - | - | + |
| hsa-miR-320a | TXNDC5 | - | + | - | - |
| hsa-miR-320a | TYR | - | - | - | + |
| hsa-miR-320a | UACA | - | - | - | + |
| hsa-miR-320a | UAP1 | - | - | - | + |
| hsa-miR-320a | UBA5 | - | - | - | + |
| hsa-miR-320a | UBA52 | - | - | + | - |
| hsa-miR-320a | UBAP2 | - | - | + | - |
| hsa-miR-320a | UBAP2L | - | - | + | - |
| hsa-miR-320a | UBE2C | + | - | - | - |
| hsa-miR-320a | UBE2D1 | - | - | - | + |
| hsa-miR-320a | UBE2D2 | - | - | - | + |
| hsa-miR-320a | UBE2D3 | - | + | - | + |
| hsa-miR-320a | UBE2E3 | - | - | - | + |
| hsa-miR-320a | UBE2G1 | - | - | + | + |
| hsa-miR-320a | UBE2H | - | - | - | + |
| hsa-miR-320a | UBE2I | - | - | - | + |
| hsa-miR-320a | UBE2K | - | - | - | + |
| hsa-miR-320a | UBE2O | - | - | - | + |
| hsa-miR-320a | UBE2Q1 | - | - | - | + |
| hsa-miR-320a | UBE2Q2 | - | - | - | + |
| hsa-miR-320a | UBE2W | - | - | - | + |
| hsa-miR-320a | UBE2Z | - | - | - | + |
| hsa-miR-320a | UBE3A | - | - | - | + |
| hsa-miR-320a | UBE3C | - | - | - | + |
| hsa-miR-320a | UBE4A | - | - | - | + |
| hsa-miR-320a | UBFD1 | - | - | - | + |
| hsa-miR-320a | UBIAD1 | - | - | - | + |
| hsa-miR-320a | UBL3 | - | - | - | + |
| hsa-miR-320a | UBLCP1 | - | - | - | + |
| hsa-miR-320a | UBP1 | - | - | - | + |
| hsa-miR-320a | UBQLN1 | - | - | - | + |
| hsa-miR-320a | UBQLN4 | - | - | + | - |
| hsa-miR-320a | UBR2 | - | - | - | + |
| hsa-miR-320a | UBR7 | - | - | - | + |
| hsa-miR-320a | UBTD2 | - | - | - | + |
| hsa-miR-320a | UBXN10 | - | - | - | + |
| hsa-miR-320a | UBXN2B | - | - | - | + |
| hsa-miR-320a | UBXN4 | - | - | - | + |
| hsa-miR-320a | UCKL1 | + | - | - | - |
| hsa-miR-320a | UFD1L | - | - | - | + |
| hsa-miR-320a | UGCG | - | - | - | + |
| hsa-miR-320a | UGT2A3 | - | - | - | + |
| hsa-miR-320a | UGT8 | - | - | - | + |
| hsa-miR-320a | UHMK1 | - | - | - | + |
| hsa-miR-320a | UHRF1BP1 | - | - | - | + |
| hsa-miR-320a | UHRF1BP1L | - | - | - | + |
| hsa-miR-320a | ULK1 | - | - | + | + |
| hsa-miR-320a | UNC119B | - | + | - | + |
| hsa-miR-320a | UNC13A | - | - | - | + |
| hsa-miR-320a | UNC13D | - | - | + | - |
| hsa-miR-320a | UNC5A | - | - | + | - |
| hsa-miR-320a | UNC5C | - | - | - | + |
| hsa-miR-320a | UNC5D | - | - | - | + |
| hsa-miR-320a | UNC79 | - | - | - | + |
| hsa-miR-320a | UNC80 | - | - | - | + |
| hsa-miR-320a | UPF1 | - | - | - | + |
| hsa-miR-320a | UPK1B | - | - | - | + |
| hsa-miR-320a | URB1 | - | - | - | + |
| hsa-miR-320a | URB2 | - | - | - | + |
| hsa-miR-320a | UROS | - | - | - | + |
| hsa-miR-320a | USB1 | - | - | - | + |
| hsa-miR-320a | USF2 | - | - | + | - |
| hsa-miR-320a | USF3 | - | + | - | - |
| hsa-miR-320a | USP1 | - | - | - | + |
| hsa-miR-320a | USP12 | + | + | - | + |
| hsa-miR-320a | USP16 | - | - | + | + |
| hsa-miR-320a | USP2 | - | - | - | + |
| hsa-miR-320a | USP20 | - | - | - | + |
| hsa-miR-320a | USP22 | - | - | + | - |
| hsa-miR-320a | USP24 | - | - | - | + |
| hsa-miR-320a | USP25 | + | + | - | + |
| hsa-miR-320a | USP30 | - | - | - | + |
| hsa-miR-320a | USP31 | - | - | - | + |
| hsa-miR-320a | USP37 | - | - | - | + |
| hsa-miR-320a | USP40 | - | - | - | + |
| hsa-miR-320a | USP42 | - | - | + | - |
| hsa-miR-320a | USP43 | - | - | - | + |
| hsa-miR-320a | USP44 | - | + | - | + |
| hsa-miR-320a | USP46 | - | + | - | + |
| hsa-miR-320a | USP47 | - | - | - | + |
| hsa-miR-320a | USP51 | - | - | - | + |
| hsa-miR-320a | USP9Y | - | - | - | + |
| hsa-miR-320a | USPL1 | - | - | + | - |
| hsa-miR-320a | UST | - | - | - | + |
| hsa-miR-320a | UTP14C | - | + | - | + |
| hsa-miR-320a | UTP18 | + | - | - | - |
| hsa-miR-320a | UTP23 | - | - | - | + |
| hsa-miR-320a | UTRN | - | - | - | + |
| hsa-miR-320a | UTS2D | - | - | - | + |
| hsa-miR-320a | UTY | - | + | - | + |
| hsa-miR-320a | UXS1 | - | - | - | + |
| hsa-miR-320a | VAMP4 | - | - | - | + |
| hsa-miR-320a | VAMP7 | - | - | - | + |
| hsa-miR-320a | VANGL1 | - | - | - | + |
| hsa-miR-320a | VAPB | - | - | - | + |
| hsa-miR-320a | VASH1 | - | - | - | + |
| hsa-miR-320a | VASH2 | - | - | - | + |
| hsa-miR-320a | VASN | - | - | - | + |
| hsa-miR-320a | VAT1 | - | - | - | + |
| hsa-miR-320a | VAT1L | - | - | - | + |
| hsa-miR-320a | VAV2 | - | - | + | - |
| hsa-miR-320a | VBP1 | - | - | - | + |
| hsa-miR-320a | VCAM1 | - | - | - | + |
| hsa-miR-320a | VCAN | - | - | - | + |
| hsa-miR-320a | VCL | - | - | + | - |
| hsa-miR-320a | VCPIP1 | - | - | - | + |
| hsa-miR-320a | VDAC1 | - | - | + | + |
| hsa-miR-320a | VDAC2 | - | - | + | - |
| hsa-miR-320a | VENTX | - | - | - | + |
| hsa-miR-320a | VEPH1 | - | + | - | - |
| hsa-miR-320a | VEZT | - | + | - | - |
| hsa-miR-320a | VGLL3 | - | - | - | + |
| hsa-miR-320a | VGLL4 | - | - | - | + |
| hsa-miR-320a | VHL | - | + | - | - |
| hsa-miR-320a | VIM | + | - | - | - |
| hsa-miR-320a | VIPAS39 | - | - | - | + |
| hsa-miR-320a | VIPR1 | - | - | - | + |
| hsa-miR-320a | VIPR2 | - | - | - | + |
| hsa-miR-320a | VIT | - | - | - | + |
| hsa-miR-320a | VKORC1 | - | - | + | - |
| hsa-miR-320a | VLDLR | - | + | - | - |
| hsa-miR-320a | VMA21 | + | - | - | + |
| hsa-miR-320a | VMP1 | - | - | - | + |
| hsa-miR-320a | VOPP1 | - | - | + | - |
| hsa-miR-320a | VPS13D | - | - | + | - |
| hsa-miR-320a | VPS18 | - | - | - | + |
| hsa-miR-320a | VPS36 | - | - | - | + |
| hsa-miR-320a | VPS37B | + | + | - | + |
| hsa-miR-320a | VPS39 | - | + | - | + |
| hsa-miR-320a | VPS41 | - | - | - | + |
| hsa-miR-320a | VPS45 | - | - | + | + |
| hsa-miR-320a | VPS4B | - | - | - | + |
| hsa-miR-320a | VPS53 | - | - | - | + |
| hsa-miR-320a | VPS8 | - | + | - | - |
| hsa-miR-320a | VRTN | - | - | - | + |
| hsa-miR-320a | VSIG1 | - | - | - | + |
| hsa-miR-320a | VSIG10 | - | + | - | + |
| hsa-miR-320a | VSIG4 | - | - | - | + |
| hsa-miR-320a | VSTM2A | - | - | - | + |
| hsa-miR-320a | VTCN1 | - | - | - | + |
| hsa-miR-320a | VTI1A | - | - | - | + |
| hsa-miR-320a | VWA5A | - | - | - | + |
| hsa-miR-320a | VWC2L | - | - | - | + |
| hsa-miR-320a | WAC | - | - | - | + |
| hsa-miR-320a | WAPAL | - | - | - | + |
| hsa-miR-320a | WARS | + | + | + | + |
| hsa-miR-320a | WARS2 | - | - | - | + |
| hsa-miR-320a | WASF3 | - | - | - | + |
| hsa-miR-320a | WASL | - | - | - | + |
| hsa-miR-320a | WBP1L | - | - | + | - |
| hsa-miR-320a | WDFY1 | - | - | - | + |
| hsa-miR-320a | WDHD1 | - | - | - | + |
| hsa-miR-320a | WDR1 | - | - | - | + |
| hsa-miR-320a | WDR11 | - | - | - | + |
| hsa-miR-320a | WDR13 | - | - | + | - |
| hsa-miR-320a | WDR19 | + | + | - | - |
| hsa-miR-320a | WDR26 | - | - | - | + |
| hsa-miR-320a | WDR35 | - | - | - | + |
| hsa-miR-320a | WDR4 | - | - | - | + |
| hsa-miR-320a | WDR43 | - | - | - | + |
| hsa-miR-320a | WDR47 | - | - | - | + |
| hsa-miR-320a | WDR5B | - | - | - | + |
| hsa-miR-320a | WDR62 | + | - | - | - |
| hsa-miR-320a | WDR76 | - | - | - | + |
| hsa-miR-320a | WDR77 | - | - | + | + |
| hsa-miR-320a | WDR82 | - | - | - | + |
| hsa-miR-320a | WDTC1 | - | - | - | + |
| hsa-miR-320a | WFDC13 | - | - | - | + |
| hsa-miR-320a | WFDC9 | - | - | - | + |
| hsa-miR-320a | WIPF1 | - | - | - | + |
| hsa-miR-320a | WIPF2 | - | - | - | + |
| hsa-miR-320a | WIPI2 | - | - | - | + |
| hsa-miR-320a | WLS | - | + | - | + |
| hsa-miR-320a | WNK1 | - | - | + | + |
| hsa-miR-320a | WNT11 | - | - | - | + |
| hsa-miR-320a | WNT8A | - | - | - | + |
| hsa-miR-320a | WRNIP1 | - | + | - | - |
| hsa-miR-320a | WSB2 | - | - | - | + |
| hsa-miR-320a | WTAP | - | - | - | + |
| hsa-miR-320a | WWC2 | - | + | - | - |
| hsa-miR-320a | WWP1 | - | - | - | + |
| hsa-miR-320a | WWTR1 | - | - | - | + |
| hsa-miR-320a | XAF1 | - | - | - | + |
| hsa-miR-320a | XAGE1A | + | - | - | - |
| hsa-miR-320a | XAGE1B | + | - | - | - |
| hsa-miR-320a | XAGE1C | + | - | - | - |
| hsa-miR-320a | XAGE1D | + | - | - | - |
| hsa-miR-320a | XAGE1E | + | - | - | - |
| hsa-miR-320a | XAGE2B | - | - | - | + |
| hsa-miR-320a | XAGE3 | + | - | - | + |
| hsa-miR-320a | XBP1 | - | + | + | - |
| hsa-miR-320a | XCL1 | - | - | - | + |
| hsa-miR-320a | XIAP | - | + | - | + |
| hsa-miR-320a | XIRP1 | - | - | - | + |
| hsa-miR-320a | XK | - | - | - | + |
| hsa-miR-320a | XKR3 | - | - | - | + |
| hsa-miR-320a | XKR9 | - | - | - | + |
| hsa-miR-320a | XKRX | - | + | - | - |
| hsa-miR-320a | XPNPEP3 | - | - | - | + |
| hsa-miR-320a | XPO6 | - | - | + | - |
| hsa-miR-320a | XPO7 | - | - | - | + |
| hsa-miR-320a | XPR1 | - | - | - | + |
| hsa-miR-320a | XRCC1 | - | - | - | + |
| hsa-miR-320a | XRCC2 | - | - | - | + |
| hsa-miR-320a | XRCC4 | + | - | - | + |
| hsa-miR-320a | XRN1 | - | - | - | + |
| hsa-miR-320a | YAF2 | - | - | - | + |
| hsa-miR-320a | YAP1 | - | - | - | + |
| hsa-miR-320a | YBX1 | - | - | - | + |
| hsa-miR-320a | YBX2 | + | - | - | + |
| hsa-miR-320a | YIPF5 | - | - | - | + |
| hsa-miR-320a | YIPF6 | - | - | + | + |
| hsa-miR-320a | YIPF7 | - | - | - | + |
| hsa-miR-320a | YOD1 | - | + | + | + |
| hsa-miR-320a | YPEL1 | - | - | - | + |
| hsa-miR-320a | YPEL2 | - | - | + | + |
| hsa-miR-320a | YPEL5 | - | - | - | + |
| hsa-miR-320a | YRDC | - | - | + | - |
| hsa-miR-320a | YTHDC1 | - | - | - | + |
| hsa-miR-320a | YTHDC2 | - | - | - | + |
| hsa-miR-320a | YTHDF1 | - | - | - | + |
| hsa-miR-320a | YTHDF2 | - | - | - | + |
| hsa-miR-320a | YTHDF3 | - | + | - | - |
| hsa-miR-320a | YWHAE | - | + | + | + |
| hsa-miR-320a | YWHAG | - | - | - | + |
| hsa-miR-320a | YWHAH | + | + | - | - |
| hsa-miR-320a | YWHAQ | - | + | - | + |
| hsa-miR-320a | YWHAZ | - | + | - | - |
| hsa-miR-320a | ZAK | - | - | - | + |
| hsa-miR-320a | ZAR1 | - | - | - | + |
| hsa-miR-320a | ZAR1L | - | - | - | + |
| hsa-miR-320a | ZBED1 | - | - | - | + |
| hsa-miR-320a | ZBED4 | - | - | - | + |
| hsa-miR-320a | ZBTB1 | - | - | - | + |
| hsa-miR-320a | ZBTB10 | - | + | - | + |
| hsa-miR-320a | ZBTB11 | - | - | - | + |
| hsa-miR-320a | ZBTB18 | - | - | - | + |
| hsa-miR-320a | ZBTB24 | - | - | - | + |
| hsa-miR-320a | ZBTB33 | - | - | + | + |
| hsa-miR-320a | ZBTB34 | - | - | - | + |
| hsa-miR-320a | ZBTB39 | - | - | - | + |
| hsa-miR-320a | ZBTB4 | - | - | - | + |
| hsa-miR-320a | ZBTB40 | - | - | - | + |
| hsa-miR-320a | ZBTB43 | - | - | - | + |
| hsa-miR-320a | ZBTB44 | - | + | - | + |
| hsa-miR-320a | ZBTB46 | - | - | - | + |
| hsa-miR-320a | ZBTB7A | - | - | - | + |
| hsa-miR-320a | ZBTB8OS | + | - | - | - |
| hsa-miR-320a | ZBTB9 | - | - | - | + |
| hsa-miR-320a | ZC3H12B | - | - | - | + |
| hsa-miR-320a | ZC3H12C | - | + | - | + |
| hsa-miR-320a | ZC3H13 | - | - | - | + |
| hsa-miR-320a | ZC3H4 | - | - | + | + |
| hsa-miR-320a | ZC3H7B | - | + | - | + |
| hsa-miR-320a | ZCCHC10 | - | - | - | + |
| hsa-miR-320a | ZCCHC12 | - | - | - | + |
| hsa-miR-320a | ZCCHC16 | - | - | - | + |
| hsa-miR-320a | ZCCHC24 | - | - | - | + |
| hsa-miR-320a | ZDBF2 | - | - | - | + |
| hsa-miR-320a | ZDHHC1 | - | - | - | + |
| hsa-miR-320a | ZDHHC15 | - | - | - | + |
| hsa-miR-320a | ZDHHC16 | - | - | - | + |
| hsa-miR-320a | ZDHHC21 | - | + | - | + |
| hsa-miR-320a | ZDHHC23 | - | - | - | + |
| hsa-miR-320a | ZDHHC3 | - | - | - | + |
| hsa-miR-320a | ZDHHC7 | - | - | - | + |
| hsa-miR-320a | ZDHHC9 | - | - | - | + |
| hsa-miR-320a | ZEB2 | - | - | + | - |
| hsa-miR-320a | ZER1 | - | - | - | + |
| hsa-miR-320a | ZFAND1 | - | - | - | + |
| hsa-miR-320a | ZFAND5 | - | - | + | + |
| hsa-miR-320a | ZFAND6 | - | - | - | + |
| hsa-miR-320a | ZFHX3 | + | - | - | + |
| hsa-miR-320a | ZFHX4 | - | - | - | + |
| hsa-miR-320a | ZFP106 | - | - | - | + |
| hsa-miR-320a | ZFP36L2 | - | - | - | + |
| hsa-miR-320a | ZFP37 | - | - | + | - |
| hsa-miR-320a | ZFP42 | - | - | - | + |
| hsa-miR-320a | ZFP64 | - | - | + | - |
| hsa-miR-320a | ZFP91 | - | + | - | + |
| hsa-miR-320a | ZFPM2 | - | - | - | + |
| hsa-miR-320a | ZFR | - | - | - | + |
| hsa-miR-320a | ZFYVE16 | - | - | - | + |
| hsa-miR-320a | ZFYVE20 | - | - | - | + |
| hsa-miR-320a | ZFYVE9 | - | - | - | + |
| hsa-miR-320a | ZHX1 | - | - | - | + |
| hsa-miR-320a | ZHX2 | - | - | - | + |
| hsa-miR-320a | ZHX3 | - | - | - | + |
| hsa-miR-320a | ZIC1 | - | - | - | + |
| hsa-miR-320a | ZIC2 | - | - | + | + |
| hsa-miR-320a | ZIC3 | - | + | - | + |
| hsa-miR-320a | ZIM3 | - | - | - | + |
| hsa-miR-320a | ZKSCAN1 | - | - | - | + |
| hsa-miR-320a | ZMAT1 | - | - | - | + |
| hsa-miR-320a | ZMAT3 | - | - | - | + |
| hsa-miR-320a | ZMIZ1 | - | - | - | + |
| hsa-miR-320a | ZMYM1 | - | - | - | + |
| hsa-miR-320a | ZMYM4 | - | + | - | + |
| hsa-miR-320a | ZMYM5 | - | + | - | + |
| hsa-miR-320a | ZMYM6NB | + | - | - | - |
| hsa-miR-320a | ZMYND11 | - | - | - | + |
| hsa-miR-320a | ZMYND12 | - | - | - | + |
| hsa-miR-320a | ZMYND8 | - | - | - | + |
| hsa-miR-320a | ZNF107 | - | - | - | + |
| hsa-miR-320a | ZNF124 | - | - | - | + |
| hsa-miR-320a | ZNF136 | - | - | - | + |
| hsa-miR-320a | ZNF138 | - | - | - | + |
| hsa-miR-320a | ZNF146 | - | - | - | + |
| hsa-miR-320a | ZNF148 | - | - | + | + |
| hsa-miR-320a | ZNF154 | - | - | - | + |
| hsa-miR-320a | ZNF155 | - | - | + | + |
| hsa-miR-320a | ZNF182 | - | - | - | + |
| hsa-miR-320a | ZNF189 | - | - | - | + |
| hsa-miR-320a | ZNF192 | - | - | - | + |
| hsa-miR-320a | ZNF197 | + | - | - | + |
| hsa-miR-320a | ZNF20 | - | - | - | + |
| hsa-miR-320a | ZNF200 | - | - | - | + |
| hsa-miR-320a | ZNF214 | + | - | - | - |
| hsa-miR-320a | ZNF223 | - | - | - | + |
| hsa-miR-320a | ZNF23 | + | - | - | - |
| hsa-miR-320a | ZNF248 | - | - | - | + |
| hsa-miR-320a | ZNF25 | - | - | - | + |
| hsa-miR-320a | ZNF250 | - | - | - | + |
| hsa-miR-320a | ZNF254 | - | - | - | + |
| hsa-miR-320a | ZNF264 | - | - | - | + |
| hsa-miR-320a | ZNF267 | - | - | + | + |
| hsa-miR-320a | ZNF275 | - | - | + | - |
| hsa-miR-320a | ZNF280C | - | - | - | + |
| hsa-miR-320a | ZNF280D | - | - | - | + |
| hsa-miR-320a | ZNF282 | - | - | + | - |
| hsa-miR-320a | ZNF286A | - | + | - | - |
| hsa-miR-320a | ZNF292 | - | - | - | + |
| hsa-miR-320a | ZNF295 | - | - | - | + |
| hsa-miR-320a | ZNF3 | - | - | - | + |
| hsa-miR-320a | ZNF302 | - | - | - | + |
| hsa-miR-320a | ZNF318 | - | - | + | - |
| hsa-miR-320a | ZNF319 | - | - | - | + |
| hsa-miR-320a | ZNF320 | - | - | - | + |
| hsa-miR-320a | ZNF322 | - | - | - | + |
| hsa-miR-320a | ZNF333 | - | - | - | + |
| hsa-miR-320a | ZNF33A | - | - | - | + |
| hsa-miR-320a | ZNF33B | - | - | - | + |
| hsa-miR-320a | ZNF343 | - | - | - | + |
| hsa-miR-320a | ZNF345 | - | - | - | + |
| hsa-miR-320a | ZNF362 | - | - | - | + |
| hsa-miR-320a | ZNF365 | - | - | - | + |
| hsa-miR-320a | ZNF367 | - | - | - | + |
| hsa-miR-320a | ZNF385B | - | - | - | + |
| hsa-miR-320a | ZNF407 | - | - | - | + |
| hsa-miR-320a | ZNF420 | - | - | - | + |
| hsa-miR-320a | ZNF423 | - | - | - | + |
| hsa-miR-320a | ZNF425 | - | + | - | + |
| hsa-miR-320a | ZNF430 | - | - | - | + |
| hsa-miR-320a | ZNF432 | - | - | + | - |
| hsa-miR-320a | ZNF436 | - | + | + | + |
| hsa-miR-320a | ZNF439 | - | - | - | + |
| hsa-miR-320a | ZNF440 | - | - | - | + |
| hsa-miR-320a | ZNF449 | + | - | - | + |
| hsa-miR-320a | ZNF451 | - | - | + | + |
| hsa-miR-320a | ZNF460 | - | - | - | + |
| hsa-miR-320a | ZNF462 | - | - | + | + |
| hsa-miR-320a | ZNF473 | - | - | - | + |
| hsa-miR-320a | ZNF484 | - | - | - | + |
| hsa-miR-320a | ZNF493 | - | - | - | + |
| hsa-miR-320a | ZNF496 | - | - | + | - |
| hsa-miR-320a | ZNF498 | - | - | - | + |
| hsa-miR-320a | ZNF503-AS2 | - | - | - | + |
| hsa-miR-320a | ZNF507 | - | - | - | + |
| hsa-miR-320a | ZNF510 | - | - | - | + |
| hsa-miR-320a | ZNF512 | - | - | - | + |
| hsa-miR-320a | ZNF516 | - | - | - | + |
| hsa-miR-320a | ZNF518B | - | - | - | + |
| hsa-miR-320a | ZNF521 | - | - | - | + |
| hsa-miR-320a | ZNF525 | - | - | - | + |
| hsa-miR-320a | ZNF529 | - | - | - | + |
| hsa-miR-320a | ZNF536 | - | - | - | + |
| hsa-miR-320a | ZNF546 | - | - | - | + |
| hsa-miR-320a | ZNF551 | - | - | - | + |
| hsa-miR-320a | ZNF558 | - | - | - | + |
| hsa-miR-320a | ZNF559 | - | - | - | + |
| hsa-miR-320a | ZNF566 | - | - | - | + |
| hsa-miR-320a | ZNF568 | - | - | - | + |
| hsa-miR-320a | ZNF577 | - | - | - | + |
| hsa-miR-320a | ZNF587 | - | - | - | + |
| hsa-miR-320a | ZNF592 | - | - | - | + |
| hsa-miR-320a | ZNF597 | - | - | - | + |
| hsa-miR-320a | ZNF600 | - | - | + | - |
| hsa-miR-320a | ZNF607 | - | - | + | - |
| hsa-miR-320a | ZNF609 | - | - | - | + |
| hsa-miR-320a | ZNF614 | - | - | - | + |
| hsa-miR-320a | ZNF615 | - | - | - | + |
| hsa-miR-320a | ZNF618 | - | - | - | + |
| hsa-miR-320a | ZNF629 | - | - | - | + |
| hsa-miR-320a | ZNF646 | - | - | + | - |
| hsa-miR-320a | ZNF648 | - | - | - | + |
| hsa-miR-320a | ZNF652 | - | + | - | - |
| hsa-miR-320a | ZNF660 | - | - | - | + |
| hsa-miR-320a | ZNF668 | - | - | - | + |
| hsa-miR-320a | ZNF670 | - | + | - | - |
| hsa-miR-320a | ZNF671 | - | - | - | + |
| hsa-miR-320a | ZNF676 | - | - | - | + |
| hsa-miR-320a | ZNF680 | - | - | - | + |
| hsa-miR-320a | ZNF681 | - | - | - | + |
| hsa-miR-320a | ZNF682 | - | - | - | + |
| hsa-miR-320a | ZNF689 | - | - | - | + |
| hsa-miR-320a | ZNF695 | - | - | - | + |
| hsa-miR-320a | ZNF697 | - | - | - | + |
| hsa-miR-320a | ZNF699 | - | - | - | + |
| hsa-miR-320a | ZNF7 | - | - | - | + |
| hsa-miR-320a | ZNF704 | - | + | - | + |
| hsa-miR-320a | ZNF705A | - | - | - | + |
| hsa-miR-320a | ZNF705D | - | - | - | + |
| hsa-miR-320a | ZNF706 | - | - | - | + |
| hsa-miR-320a | ZNF708 | - | - | - | + |
| hsa-miR-320a | ZNF710 | - | - | + | - |
| hsa-miR-320a | ZNF716 | - | - | - | + |
| hsa-miR-320a | ZNF717 | + | - | - | - |
| hsa-miR-320a | ZNF732 | - | - | - | + |
| hsa-miR-320a | ZNF747 | - | - | - | + |
| hsa-miR-320a | ZNF765 | - | - | - | + |
| hsa-miR-320a | ZNF772 | - | - | - | + |
| hsa-miR-320a | ZNF780A | - | + | - | - |
| hsa-miR-320a | ZNF780B | - | + | - | + |
| hsa-miR-320a | ZNF785 | - | - | - | + |
| hsa-miR-320a | ZNF788 | - | - | - | + |
| hsa-miR-320a | ZNF792 | - | - | - | + |
| hsa-miR-320a | ZNF81 | - | - | - | + |
| hsa-miR-320a | ZNF827 | - | - | - | + |
| hsa-miR-320a | ZNF829 | - | - | - | + |
| hsa-miR-320a | ZNF83 | - | - | - | + |
| hsa-miR-320a | ZNF831 | - | - | - | + |
| hsa-miR-320a | ZNF84 | - | - | - | + |
| hsa-miR-320a | ZNF92 | - | - | - | + |
| hsa-miR-320a | ZNHIT6 | - | - | - | + |
| hsa-miR-320a | ZNRF3 | - | - | - | + |
| hsa-miR-320a | ZPLD1 | - | - | - | + |
| hsa-miR-320a | ZRANB1 | - | - | - | + |
| hsa-miR-320a | ZRANB2 | + | + | - | + |
| hsa-miR-320a | ZRANB3 | - | - | - | + |
| hsa-miR-320a | ZSCAN23 | - | - | - | + |
| hsa-miR-320a | ZSWIM4 | - | - | - | + |
| hsa-miR-320a | ZSWIM5 | - | - | - | + |
| hsa-miR-320a | ZSWIM6 | - | + | - | + |
| hsa-miR-320a | ZWILCH | + | - | - | + |
| hsa-miR-320a | ZXDB | - | - | - | + |

| mirna | gene_symbol | TargetScan | miRDB | miRTarbase | Tarbase |
| --- | --- | --- | --- | --- | --- |
| hsa-miR-22-3p | A4GNT | + | + | - | + |
| hsa-miR-22-3p | AADACL4 | + | - | - | - |
| hsa-miR-22-3p | AADAT | - | - | - | + |
| hsa-miR-22-3p | AAGAB | + | - | - | + |
| hsa-miR-22-3p | AAMP | - | - | - | + |
| hsa-miR-22-3p | AAR2 | + | - | - | + |
| hsa-miR-22-3p | AARS2 | - | - | - | + |
| hsa-miR-22-3p | AATK | + | - | - | - |
| hsa-miR-22-3p | ABAT | - | - | - | + |
| hsa-miR-22-3p | ABCA12 | - | - | - | + |
| hsa-miR-22-3p | ABCA4 | - | - | - | + |
| hsa-miR-22-3p | ABCB8 | - | - | - | + |
| hsa-miR-22-3p | ABCC10 | - | - | - | + |
| hsa-miR-22-3p | ABCC11 | + | - | - | - |
| hsa-miR-22-3p | ABCE1 | - | - | - | + |
| hsa-miR-22-3p | ABHD13 | - | - | - | + |
| hsa-miR-22-3p | ABHD2 | + | - | - | + |
| hsa-miR-22-3p | ABHD3 | + | - | - | - |
| hsa-miR-22-3p | ABI3 | + | - | - | - |
| hsa-miR-22-3p | ABL1 | - | - | - | + |
| hsa-miR-22-3p | ABL2 | - | - | - | + |
| hsa-miR-22-3p | ABLIM1 | - | - | - | + |
| hsa-miR-22-3p | ABR | - | - | - | + |
| hsa-miR-22-3p | AC004381.6 | + | - | - | - |
| hsa-miR-22-3p | AC005481.5 | - | - | - | + |
| hsa-miR-22-3p | AC005609.1 | + | - | - | - |
| hsa-miR-22-3p | AC006486.1 | + | - | - | - |
| hsa-miR-22-3p | AC007375.1 | + | - | - | - |
| hsa-miR-22-3p | AC008132.13 | + | - | - | - |
| hsa-miR-22-3p | AC008443.1 | + | - | - | - |
| hsa-miR-22-3p | AC010536.2 | - | - | - | + |
| hsa-miR-22-3p | AC015804.1 | - | - | - | + |
| hsa-miR-22-3p | AC016559.1 | + | - | - | - |
| hsa-miR-22-3p | AC016586.1 | + | - | - | - |
| hsa-miR-22-3p | AC026310.1 | + | - | - | - |
| hsa-miR-22-3p | AC069547.1 | + | - | - | - |
| hsa-miR-22-3p | AC079210.1 | + | - | - | - |
| hsa-miR-22-3p | AC104841.2 | + | - | - | - |
| hsa-miR-22-3p | AC112693.2 | + | - | - | - |
| hsa-miR-22-3p | AC119673.1 | + | - | - | - |
| hsa-miR-22-3p | AC124890.1 | + | - | - | - |
| hsa-miR-22-3p | AC133919.6 | - | - | - | + |
| hsa-miR-22-3p | AC139100.2 | - | - | - | + |
| hsa-miR-22-3p | AC174470.1 | + | - | - | - |
| hsa-miR-22-3p | ACACA | + | - | - | - |
| hsa-miR-22-3p | ACACB | - | - | - | + |
| hsa-miR-22-3p | ACADS | + | - | - | + |
| hsa-miR-22-3p | ACBD3 | - | - | - | + |
| hsa-miR-22-3p | ACER3 | + | + | - | + |
| hsa-miR-22-3p | ACLY | + | + | - | + |
| hsa-miR-22-3p | ACOT13 | - | - | - | + |
| hsa-miR-22-3p | ACOT4 | + | - | - | - |
| hsa-miR-22-3p | ACOX3 | - | - | - | + |
| hsa-miR-22-3p | ACPL2 | - | - | - | + |
| hsa-miR-22-3p | ACPP | - | - | - | + |
| hsa-miR-22-3p | ACSL4 | - | - | - | + |
| hsa-miR-22-3p | ACTR1A | - | - | - | + |
| hsa-miR-22-3p | ACTR2 | - | - | - | + |
| hsa-miR-22-3p | ACTR3 | - | - | - | + |
| hsa-miR-22-3p | ACVR1B | + | - | - | + |
| hsa-miR-22-3p | ACVR1C | - | - | + | - |
| hsa-miR-22-3p | ACVR2A | - | - | - | + |
| hsa-miR-22-3p | ACVRL1 | - | - | - | + |
| hsa-miR-22-3p | ADAM11 | + | - | - | - |
| hsa-miR-22-3p | ADAM12 | + | - | - | + |
| hsa-miR-22-3p | ADAM22 | - | - | - | + |
| hsa-miR-22-3p | ADAM28 | - | - | - | + |
| hsa-miR-22-3p | ADAM33 | + | - | - | + |
| hsa-miR-22-3p | ADAMTS1 | - | - | - | + |
| hsa-miR-22-3p | ADAMTS12 | - | - | - | + |
| hsa-miR-22-3p | ADAMTS14 | - | - | - | + |
| hsa-miR-22-3p | ADAMTS15 | - | - | - | + |
| hsa-miR-22-3p | ADAMTS3 | - | - | - | + |
| hsa-miR-22-3p | ADAMTS5 | - | - | - | + |
| hsa-miR-22-3p | ADAMTSL1 | - | - | - | + |
| hsa-miR-22-3p | ADAMTSL5 | + | - | - | - |
| hsa-miR-22-3p | ADAT2 | + | - | - | - |
| hsa-miR-22-3p | ADCK2 | + | + | - | + |
| hsa-miR-22-3p | ADCY1 | - | - | - | + |
| hsa-miR-22-3p | ADCY2 | - | - | - | + |
| hsa-miR-22-3p | ADCY5 | - | - | - | + |
| hsa-miR-22-3p | ADCY6 | + | - | - | - |
| hsa-miR-22-3p | ADCY7 | + | - | - | - |
| hsa-miR-22-3p | ADCYAP1 | - | - | - | + |
| hsa-miR-22-3p | ADD1 | - | - | - | + |
| hsa-miR-22-3p | ADGRG6 | - | - | - | + |
| hsa-miR-22-3p | ADH5 | - | - | - | + |
| hsa-miR-22-3p | ADH6 | - | - | - | + |
| hsa-miR-22-3p | ADIG | - | - | - | + |
| hsa-miR-22-3p | ADIPOR2 | - | - | - | + |
| hsa-miR-22-3p | ADNP | - | - | - | + |
| hsa-miR-22-3p | ADORA1 | + | - | - | - |
| hsa-miR-22-3p | ADORA2A | - | - | - | + |
| hsa-miR-22-3p | ADPRHL2 | - | - | - | + |
| hsa-miR-22-3p | ADSL | - | - | - | + |
| hsa-miR-22-3p | AEN | - | - | - | + |
| hsa-miR-22-3p | AES | - | - | - | + |
| hsa-miR-22-3p | AFAP1L1 | - | - | - | + |
| hsa-miR-22-3p | AFF4 | - | - | - | + |
| hsa-miR-22-3p | AGAP1 | + | - | - | + |
| hsa-miR-22-3p | AGBL5 | - | + | - | + |
| hsa-miR-22-3p | AGFG1 | - | - | - | + |
| hsa-miR-22-3p | AGFG2 | - | - | - | + |
| hsa-miR-22-3p | AGGF1 | - | - | - | + |
| hsa-miR-22-3p | AGMO | - | - | - | + |
| hsa-miR-22-3p | AGO1 | + | - | - | - |
| hsa-miR-22-3p | AGPAT6 | - | - | - | + |
| hsa-miR-22-3p | AGPAT9 | + | - | - | + |
| hsa-miR-22-3p | AGPS | - | - | - | + |
| hsa-miR-22-3p | AGTPBP1 | - | - | - | + |
| hsa-miR-22-3p | AGTR2 | - | - | - | + |
| hsa-miR-22-3p | AHCTF1 | - | - | - | + |
| hsa-miR-22-3p | AHCYL1 | - | - | - | + |
| hsa-miR-22-3p | AHCYL2 | - | - | - | + |
| hsa-miR-22-3p | AHI1 | - | - | - | + |
| hsa-miR-22-3p | AHRR | - | - | - | + |
| hsa-miR-22-3p | AHSG | - | - | - | + |
| hsa-miR-22-3p | AIM1 | - | - | - | + |
| hsa-miR-22-3p | AIMP1 | + | - | - | + |
| hsa-miR-22-3p | AK2 | + | - | - | + |
| hsa-miR-22-3p | AKAP10 | - | - | - | + |
| hsa-miR-22-3p | AKAP11 | - | - | - | + |
| hsa-miR-22-3p | AKAP13 | - | - | - | + |
| hsa-miR-22-3p | AKR1A1 | + | - | - | - |
| hsa-miR-22-3p | AKT3 | + | + | - | - |
| hsa-miR-22-3p | AL009178.1 | + | - | - | - |
| hsa-miR-22-3p | AL138847.1 | + | - | - | - |
| hsa-miR-22-3p | AL353791.1 | + | - | - | - |
| hsa-miR-22-3p | AL355390.1 | + | - | - | - |
| hsa-miR-22-3p | AL391421.1 | + | - | - | - |
| hsa-miR-22-3p | AL589765.1 | + | - | - | - |
| hsa-miR-22-3p | AL953854.2 | + | - | - | - |
| hsa-miR-22-3p | ALAD | - | - | - | + |
| hsa-miR-22-3p | ALCAM | + | - | - | + |
| hsa-miR-22-3p | ALDH18A1 | - | - | - | + |
| hsa-miR-22-3p | ALDH3B2 | - | - | - | + |
| hsa-miR-22-3p | ALDH6A1 | - | - | - | + |
| hsa-miR-22-3p | ALG1L | - | - | - | + |
| hsa-miR-22-3p | ALG9 | - | + | - | - |
| hsa-miR-22-3p | ALKBH1 | - | - | - | + |
| hsa-miR-22-3p | ALKBH5 | - | - | - | + |
| hsa-miR-22-3p | ALKBH8 | - | - | - | + |
| hsa-miR-22-3p | ALMS1 | - | - | + | - |
| hsa-miR-22-3p | ALOXE3 | + | - | - | - |
| hsa-miR-22-3p | ALPK3 | + | - | - | + |
| hsa-miR-22-3p | ALPPL2 | - | - | - | + |
| hsa-miR-22-3p | ALX1 | - | - | - | + |
| hsa-miR-22-3p | ALX4 | + | - | - | + |
| hsa-miR-22-3p | AMBN | - | - | - | + |
| hsa-miR-22-3p | AMBRA1 | - | - | - | + |
| hsa-miR-22-3p | AMER1 | - | - | - | + |
| hsa-miR-22-3p | AMFR | + | - | - | + |
| hsa-miR-22-3p | AMMECR1L | + | - | - | + |
| hsa-miR-22-3p | AMOT | - | + | - | + |
| hsa-miR-22-3p | AMOTL1 | - | - | - | + |
| hsa-miR-22-3p | AMPD2 | - | - | - | + |
| hsa-miR-22-3p | AMPH | + | - | - | + |
| hsa-miR-22-3p | AMZ1 | + | - | - | + |
| hsa-miR-22-3p | ANAPC1 | - | - | - | + |
| hsa-miR-22-3p | ANGEL2 | - | + | - | + |
| hsa-miR-22-3p | ANGPTL2 | - | - | - | + |
| hsa-miR-22-3p | ANKFY1 | - | - | - | + |
| hsa-miR-22-3p | ANKH | - | - | - | + |
| hsa-miR-22-3p | ANKHD1-EIF4EBP3 | + | - | - | - |
| hsa-miR-22-3p | ANKRD13A | + | + | - | + |
| hsa-miR-22-3p | ANKRD13B | - | - | - | + |
| hsa-miR-22-3p | ANKRD13C | - | - | - | + |
| hsa-miR-22-3p | ANKRD18A | + | - | - | - |
| hsa-miR-22-3p | ANKRD23 | - | - | - | + |
| hsa-miR-22-3p | ANKRD28 | - | - | - | + |
| hsa-miR-22-3p | ANKRD30BL | - | - | - | + |
| hsa-miR-22-3p | ANKRD34B | - | - | - | + |
| hsa-miR-22-3p | ANKRD35 | - | - | - | + |
| hsa-miR-22-3p | ANKRD36BP1 | - | - | - | + |
| hsa-miR-22-3p | ANKRD40 | - | - | - | + |
| hsa-miR-22-3p | ANKRD46 | - | - | - | + |
| hsa-miR-22-3p | ANKRD50 | - | - | - | + |
| hsa-miR-22-3p | ANKRD52 | - | - | - | + |
| hsa-miR-22-3p | ANKRD53 | + | - | - | + |
| hsa-miR-22-3p | ANKRD54 | - | - | - | + |
| hsa-miR-22-3p | ANKRD6 | - | + | - | + |
| hsa-miR-22-3p | ANKS1A | - | - | - | + |
| hsa-miR-22-3p | ANO4 | - | - | - | + |
| hsa-miR-22-3p | ANO6 | - | - | - | + |
| hsa-miR-22-3p | ANOS1 | - | - | - | + |
| hsa-miR-22-3p | ANP32E | + | - | - | - |
| hsa-miR-22-3p | ANTXR2 | - | - | - | + |
| hsa-miR-22-3p | ANXA13 | + | + | - | + |
| hsa-miR-22-3p | ANXA3 | - | - | - | + |
| hsa-miR-22-3p | AP000679.2 | - | - | - | + |
| hsa-miR-22-3p | AP1B1 | - | - | - | + |
| hsa-miR-22-3p | AP1G1 | - | - | - | + |
| hsa-miR-22-3p | AP1S1 | + | - | - | + |
| hsa-miR-22-3p | AP3M1 | - | - | - | + |
| hsa-miR-22-3p | AP3S2 | - | - | - | + |
| hsa-miR-22-3p | APBB2 | + | - | - | + |
| hsa-miR-22-3p | APCDD1L | + | + | - | + |
| hsa-miR-22-3p | APH1B | - | - | - | + |
| hsa-miR-22-3p | APOA5 | + | - | - | - |
| hsa-miR-22-3p | APOBEC3H | - | - | - | + |
| hsa-miR-22-3p | APOBEC4 | + | - | - | + |
| hsa-miR-22-3p | APPL1 | - | - | - | + |
| hsa-miR-22-3p | AQP1 | - | - | - | + |
| hsa-miR-22-3p | AQP9 | + | - | - | + |
| hsa-miR-22-3p | AR | - | - | - | + |
| hsa-miR-22-3p | ARC | - | - | - | + |
| hsa-miR-22-3p | ARF1 | - | - | - | + |
| hsa-miR-22-3p | ARF3 | - | - | - | + |
| hsa-miR-22-3p | ARFGAP2 | - | - | - | + |
| hsa-miR-22-3p | ARFGEF2 | - | - | - | + |
| hsa-miR-22-3p | ARFIP1 | - | - | - | + |
| hsa-miR-22-3p | ARFIP2 | + | + | - | - |
| hsa-miR-22-3p | ARHGAP17 | + | - | - | - |
| hsa-miR-22-3p | ARHGAP18 | - | - | - | + |
| hsa-miR-22-3p | ARHGAP19-SLIT1 | + | - | - | - |
| hsa-miR-22-3p | ARHGAP26 | - | - | - | + |
| hsa-miR-22-3p | ARHGAP27 | - | - | - | + |
| hsa-miR-22-3p | ARHGAP28 | - | - | - | + |
| hsa-miR-22-3p | ARHGAP35 | - | - | - | + |
| hsa-miR-22-3p | ARHGAP4 | + | - | - | - |
| hsa-miR-22-3p | ARHGAP9 | - | - | - | + |
| hsa-miR-22-3p | ARHGDIB | - | - | - | + |
| hsa-miR-22-3p | ARHGEF12 | + | + | - | + |
| hsa-miR-22-3p | ARHGEF2 | - | - | - | + |
| hsa-miR-22-3p | ARHGEF26 | + | + | - | + |
| hsa-miR-22-3p | ARHGEF6 | - | - | - | + |
| hsa-miR-22-3p | ARHGEF7 | - | - | - | + |
| hsa-miR-22-3p | ARHGEF9 | - | - | - | + |
| hsa-miR-22-3p | ARID3B | + | + | - | + |
| hsa-miR-22-3p | ARID5A | + | - | - | - |
| hsa-miR-22-3p | ARID5B | - | - | + | + |
| hsa-miR-22-3p | ARIH1 | - | + | - | - |
| hsa-miR-22-3p | ARL1 | - | - | - | + |
| hsa-miR-22-3p | ARL10 | + | - | - | - |
| hsa-miR-22-3p | ARL5B | - | - | - | + |
| hsa-miR-22-3p | ARL6IP5 | + | - | - | - |
| hsa-miR-22-3p | ARMC1 | - | - | - | + |
| hsa-miR-22-3p | ARMC3 | - | - | - | + |
| hsa-miR-22-3p | ARMC5 | - | - | - | + |
| hsa-miR-22-3p | ARMCX2 | - | - | - | + |
| hsa-miR-22-3p | ARMCX6 | - | - | - | + |
| hsa-miR-22-3p | ARNT | - | - | - | + |
| hsa-miR-22-3p | ARPC5 | + | + | + | - |
| hsa-miR-22-3p | ARPP19 | - | - | - | + |
| hsa-miR-22-3p | ARRB1 | + | + | - | - |
| hsa-miR-22-3p | ARRDC2 | + | - | - | + |
| hsa-miR-22-3p | ARRDC3 | - | - | - | + |
| hsa-miR-22-3p | ARSD | + | - | - | + |
| hsa-miR-22-3p | ARVCF | + | - | - | - |
| hsa-miR-22-3p | ASAP2 | - | - | - | + |
| hsa-miR-22-3p | ASB14 | - | - | - | + |
| hsa-miR-22-3p | ASB6 | + | + | - | - |
| hsa-miR-22-3p | ASB8 | - | - | - | + |
| hsa-miR-22-3p | ASCC1 | - | - | - | + |
| hsa-miR-22-3p | ASCL1 | + | - | - | + |
| hsa-miR-22-3p | ASPN | - | - | - | + |
| hsa-miR-22-3p | ASS1 | + | + | - | + |
| hsa-miR-22-3p | ASTN2 | - | - | - | + |
| hsa-miR-22-3p | ATAD2B | - | - | - | + |
| hsa-miR-22-3p | ATAD5 | - | - | - | + |
| hsa-miR-22-3p | ATF6 | - | - | - | + |
| hsa-miR-22-3p | ATG14 | - | - | - | + |
| hsa-miR-22-3p | ATG2B | - | + | - | + |
| hsa-miR-22-3p | ATG3 | - | - | - | + |
| hsa-miR-22-3p | ATG4B | - | - | - | + |
| hsa-miR-22-3p | ATG7 | - | - | - | + |
| hsa-miR-22-3p | ATHL1 | + | - | - | + |
| hsa-miR-22-3p | ATL3 | - | - | - | + |
| hsa-miR-22-3p | ATMIN | - | - | - | + |
| hsa-miR-22-3p | ATOH8 | + | - | - | - |
| hsa-miR-22-3p | ATP11A | - | - | - | + |
| hsa-miR-22-3p | ATP11B | - | - | - | + |
| hsa-miR-22-3p | ATP13A2 | - | - | - | + |
| hsa-miR-22-3p | ATP2A3 | - | - | - | + |
| hsa-miR-22-3p | ATP2B4 | - | - | - | + |
| hsa-miR-22-3p | ATP5G1 | - | - | - | + |
| hsa-miR-22-3p | ATP5SL | - | - | - | + |
| hsa-miR-22-3p | ATP6V1A | + | - | - | + |
| hsa-miR-22-3p | ATP6V1C1 | - | - | - | + |
| hsa-miR-22-3p | ATP7A | - | - | - | + |
| hsa-miR-22-3p | ATP7B | - | - | - | + |
| hsa-miR-22-3p | ATP8A1 | + | + | - | - |
| hsa-miR-22-3p | ATP9A | - | + | - | + |
| hsa-miR-22-3p | ATPIF1 | + | + | - | + |
| hsa-miR-22-3p | ATRX | - | - | - | + |
| hsa-miR-22-3p | ATXN1 | - | - | - | + |
| hsa-miR-22-3p | ATXN1L | - | - | - | + |
| hsa-miR-22-3p | ATXN3 | - | - | - | + |
| hsa-miR-22-3p | ATXN7 | - | + | - | + |
| hsa-miR-22-3p | ATXN7L1 | - | - | - | + |
| hsa-miR-22-3p | ATXN7L3 | + | - | - | - |
| hsa-miR-22-3p | AVEN | - | - | - | + |
| hsa-miR-22-3p | AXIN1 | - | - | - | + |
| hsa-miR-22-3p | AZIN1 | - | - | - | + |
| hsa-miR-22-3p | B3GAT1 | - | - | - | + |
| hsa-miR-22-3p | B3GNT5 | - | - | - | + |
| hsa-miR-22-3p | B3GNT6 | + | - | - | - |
| hsa-miR-22-3p | B3GNT7 | - | - | - | + |
| hsa-miR-22-3p | B4GALNT4 | + | - | - | - |
| hsa-miR-22-3p | B4GALT2 | + | - | - | - |
| hsa-miR-22-3p | B4GALT5 | + | + | - | + |
| hsa-miR-22-3p | BACE1 | - | - | - | + |
| hsa-miR-22-3p | BACH2 | - | - | - | + |
| hsa-miR-22-3p | BAG5 | + | + | - | + |
| hsa-miR-22-3p | BAIAP3 | - | - | - | + |
| hsa-miR-22-3p | BAP1 | - | - | - | + |
| hsa-miR-22-3p | BARX1 | - | - | - | + |
| hsa-miR-22-3p | BATF3 | + | + | - | + |
| hsa-miR-22-3p | BAX | + | - | - | - |
| hsa-miR-22-3p | BCL2L2 | - | - | - | + |
| hsa-miR-22-3p | BCL7B | - | - | - | + |
| hsa-miR-22-3p | BCL9 | + | + | - | + |
| hsa-miR-22-3p | BCL9L | + | - | - | - |
| hsa-miR-22-3p | BCR | + | - | - | + |
| hsa-miR-22-3p | BDH1 | - | - | - | + |
| hsa-miR-22-3p | BDNF | - | - | + | - |
| hsa-miR-22-3p | BDP1 | + | + | - | - |
| hsa-miR-22-3p | BEND4 | - | - | - | + |
| hsa-miR-22-3p | BEST4 | - | - | - | + |
| hsa-miR-22-3p | BEX1 | - | - | - | + |
| hsa-miR-22-3p | BEX2 | - | - | - | + |
| hsa-miR-22-3p | BFSP2 | - | - | - | + |
| hsa-miR-22-3p | BHLHA15 | + | - | - | - |
| hsa-miR-22-3p | BHLHA9 | + | - | - | - |
| hsa-miR-22-3p | BHLHE40 | - | - | - | + |
| hsa-miR-22-3p | BHLHE41 | - | - | - | + |
| hsa-miR-22-3p | BHMT | - | - | - | + |
| hsa-miR-22-3p | BIN1 | + | + | - | + |
| hsa-miR-22-3p | BLK | + | - | - | + |
| hsa-miR-22-3p | BLOC1S1 | + | - | - | - |
| hsa-miR-22-3p | BMF | + | - | - | - |
| hsa-miR-22-3p | BMI1 | - | - | - | + |
| hsa-miR-22-3p | BMP2 | - | - | - | + |
| hsa-miR-22-3p | BMP6 | - | - | + | - |
| hsa-miR-22-3p | BMP7 | - | - | + | - |
| hsa-miR-22-3p | BMPR1B | - | - | + | + |
| hsa-miR-22-3p | BOD1L2 | - | - | - | + |
| hsa-miR-22-3p | BOLL | - | - | - | + |
| hsa-miR-22-3p | BPY2 | + | - | - | - |
| hsa-miR-22-3p | BPY2B | + | - | - | + |
| hsa-miR-22-3p | BPY2C | + | - | - | - |
| hsa-miR-22-3p | BRAP | - | - | - | + |
| hsa-miR-22-3p | BRD3 | - | - | - | + |
| hsa-miR-22-3p | BRD4 | - | - | - | + |
| hsa-miR-22-3p | BRI3BP | + | - | - | - |
| hsa-miR-22-3p | BRMS1 | - | - | - | + |
| hsa-miR-22-3p | BRSK2 | + | + | - | - |
| hsa-miR-22-3p | BRWD3 | + | + | + | + |
| hsa-miR-22-3p | BSCL2 | + | - | - | - |
| hsa-miR-22-3p | BSG | - | - | + | - |
| hsa-miR-22-3p | BSN | - | - | - | + |
| hsa-miR-22-3p | BSPRY | + | + | - | + |
| hsa-miR-22-3p | BTBD10 | + | + | - | - |
| hsa-miR-22-3p | BTBD3 | + | - | - | - |
| hsa-miR-22-3p | BTBD9 | + | - | - | + |
| hsa-miR-22-3p | BTF3 | + | - | + | - |
| hsa-miR-22-3p | BTG1 | + | + | + | + |
| hsa-miR-22-3p | BTG2 | - | - | - | + |
| hsa-miR-22-3p | BTK | - | - | - | + |
| hsa-miR-22-3p | BTN1A1 | - | - | - | + |
| hsa-miR-22-3p | BTN3A3 | - | - | + | - |
| hsa-miR-22-3p | BTNL3 | - | - | - | + |
| hsa-miR-22-3p | BTRC | - | - | - | + |
| hsa-miR-22-3p | BUB1B | - | - | + | - |
| hsa-miR-22-3p | BVES | - | - | - | + |
| hsa-miR-22-3p | BZRAP1 | - | - | - | + |
| hsa-miR-22-3p | C10orf105 | + | - | - | - |
| hsa-miR-22-3p | C10orf11 | + | - | - | - |
| hsa-miR-22-3p | C10orf113 | + | - | - | + |
| hsa-miR-22-3p | C10orf12 | - | - | - | + |
| hsa-miR-22-3p | C10orf54 | - | - | - | + |
| hsa-miR-22-3p | C10orf67 | - | - | - | + |
| hsa-miR-22-3p | C10orf76 | + | - | - | + |
| hsa-miR-22-3p | C11orf34 | + | - | - | - |
| hsa-miR-22-3p | C11orf68 | - | - | - | + |
| hsa-miR-22-3p | C11orf9 | - | - | - | + |
| hsa-miR-22-3p | C12orf36 | - | - | - | + |
| hsa-miR-22-3p | C12orf4 | - | - | - | + |
| hsa-miR-22-3p | C12orf69 | - | - | - | + |
| hsa-miR-22-3p | C13orf33 | - | - | - | + |
| hsa-miR-22-3p | C14orf119 | + | - | - | - |
| hsa-miR-22-3p | C14orf129 | - | - | - | + |
| hsa-miR-22-3p | C14orf80 | - | - | - | + |
| hsa-miR-22-3p | C15orf39 | + | - | - | - |
| hsa-miR-22-3p | C15orf40 | - | - | + | - |
| hsa-miR-22-3p | C15orf62 | + | - | - | + |
| hsa-miR-22-3p | C16orf58 | + | - | - | - |
| hsa-miR-22-3p | C16orf71 | - | - | - | + |
| hsa-miR-22-3p | C16orf96 | - | - | - | + |
| hsa-miR-22-3p | C17orf101 | - | - | - | + |
| hsa-miR-22-3p | C17orf103 | + | - | - | - |
| hsa-miR-22-3p | C17orf47 | - | - | - | + |
| hsa-miR-22-3p | C17orf51 | + | - | - | + |
| hsa-miR-22-3p | C17orf53 | - | - | - | + |
| hsa-miR-22-3p | C17orf58 | + | - | - | - |
| hsa-miR-22-3p | C17orf72 | - | - | - | + |
| hsa-miR-22-3p | C17orf78 | + | - | - | - |
| hsa-miR-22-3p | C17orf80 | - | - | - | + |
| hsa-miR-22-3p | C17orf85 | + | - | - | - |
| hsa-miR-22-3p | C18orf26 | - | - | - | + |
| hsa-miR-22-3p | C18orf54 | - | - | - | + |
| hsa-miR-22-3p | C18orf62 | - | - | - | + |
| hsa-miR-22-3p | C19orf26 | + | - | - | - |
| hsa-miR-22-3p | C19orf44 | - | - | - | + |
| hsa-miR-22-3p | C19orf70 | + | - | - | - |
| hsa-miR-22-3p | C1QTNF2 | - | - | - | + |
| hsa-miR-22-3p | C1QTNF9 | - | - | - | + |
| hsa-miR-22-3p | C1orf110 | + | - | - | - |
| hsa-miR-22-3p | C1orf114 | - | - | - | + |
| hsa-miR-22-3p | C1orf115 | + | - | - | + |
| hsa-miR-22-3p | C1orf116 | - | - | - | + |
| hsa-miR-22-3p | C1orf122 | + | - | - | - |
| hsa-miR-22-3p | C1orf123 | + | - | - | + |
| hsa-miR-22-3p | C1orf145 | - | - | - | + |
| hsa-miR-22-3p | C1orf180 | + | - | - | + |
| hsa-miR-22-3p | C1orf21 | - | - | - | + |
| hsa-miR-22-3p | C1orf213 | - | - | - | + |
| hsa-miR-22-3p | C1orf220 | + | - | - | - |
| hsa-miR-22-3p | C1orf226 | - | - | - | + |
| hsa-miR-22-3p | C1orf43 | - | - | - | + |
| hsa-miR-22-3p | C1orf87 | - | - | + | - |
| hsa-miR-22-3p | C1orf95 | + | - | - | + |
| hsa-miR-22-3p | C20orf118 | - | - | - | + |
| hsa-miR-22-3p | C20orf151 | - | - | - | + |
| hsa-miR-22-3p | C20orf152 | - | - | - | + |
| hsa-miR-22-3p | C20orf160 | - | - | - | + |
| hsa-miR-22-3p | C20orf194 | - | - | - | + |
| hsa-miR-22-3p | C21orf88 | - | - | - | + |
| hsa-miR-22-3p | C21orf91 | - | - | - | + |
| hsa-miR-22-3p | C22orf26 | + | - | - | - |
| hsa-miR-22-3p | C2CD2 | - | - | - | + |
| hsa-miR-22-3p | C2CD2L | + | - | - | + |
| hsa-miR-22-3p | C2CD4C | - | - | - | + |
| hsa-miR-22-3p | C2orf44 | - | - | - | + |
| hsa-miR-22-3p | C2orf50 | + | - | - | - |
| hsa-miR-22-3p | C2orf65 | - | - | - | + |
| hsa-miR-22-3p | C2orf71 | - | - | - | + |
| hsa-miR-22-3p | C2orf72 | + | - | - | + |
| hsa-miR-22-3p | C2orf91 | - | - | - | + |
| hsa-miR-22-3p | C3orf14 | - | - | - | + |
| hsa-miR-22-3p | C3orf27 | + | - | - | - |
| hsa-miR-22-3p | C3orf52 | + | - | - | + |
| hsa-miR-22-3p | C5orf15 | - | - | - | + |
| hsa-miR-22-3p | C5orf24 | + | + | + | + |
| hsa-miR-22-3p | C5orf43 | - | - | - | + |
| hsa-miR-22-3p | C5orf44 | - | - | - | + |
| hsa-miR-22-3p | C5orf51 | - | - | - | + |
| hsa-miR-22-3p | C5orf62 | - | - | - | + |
| hsa-miR-22-3p | C5orf67 | - | + | - | - |
| hsa-miR-22-3p | C6orf108 | - | - | - | + |
| hsa-miR-22-3p | C6orf201 | + | - | - | - |
| hsa-miR-22-3p | C6orf222 | - | - | - | + |
| hsa-miR-22-3p | C6orf228 | - | - | - | + |
| hsa-miR-22-3p | C7 | - | - | - | + |
| hsa-miR-22-3p | C7orf23 | - | - | - | + |
| hsa-miR-22-3p | C7orf49 | + | + | - | - |
| hsa-miR-22-3p | C7orf60 | - | - | - | + |
| hsa-miR-22-3p | C7orf73 | - | - | - | + |
| hsa-miR-22-3p | C8orf42 | - | - | - | + |
| hsa-miR-22-3p | C8orf46 | + | + | - | - |
| hsa-miR-22-3p | C8orf49 | - | - | - | + |
| hsa-miR-22-3p | C8orf58 | - | - | - | + |
| hsa-miR-22-3p | C8orf86 | - | - | - | + |
| hsa-miR-22-3p | C9orf170 | + | - | - | - |
| hsa-miR-22-3p | C9orf69 | + | - | - | + |
| hsa-miR-22-3p | C9orf91 | - | - | - | + |
| hsa-miR-22-3p | CA13 | - | - | - | + |
| hsa-miR-22-3p | CA7 | + | - | - | + |
| hsa-miR-22-3p | CAB39 | - | - | - | + |
| hsa-miR-22-3p | CAB39L | - | - | - | + |
| hsa-miR-22-3p | CABLES1 | - | - | - | + |
| hsa-miR-22-3p | CABLES2 | - | - | - | + |
| hsa-miR-22-3p | CABP7 | - | - | - | + |
| hsa-miR-22-3p | CACNA1I | - | - | - | + |
| hsa-miR-22-3p | CACNA1S | - | - | - | + |
| hsa-miR-22-3p | CACNA2D2 | - | - | - | + |
| hsa-miR-22-3p | CACNB1 | - | - | - | + |
| hsa-miR-22-3p | CACNB2 | - | - | - | + |
| hsa-miR-22-3p | CACNB4 | - | - | - | + |
| hsa-miR-22-3p | CACNG3 | + | - | - | + |
| hsa-miR-22-3p | CACUL1 | + | - | - | - |
| hsa-miR-22-3p | CAD | - | - | - | + |
| hsa-miR-22-3p | CADM3 | + | - | - | + |
| hsa-miR-22-3p | CADPS2 | - | - | - | + |
| hsa-miR-22-3p | CALB1 | - | - | - | + |
| hsa-miR-22-3p | CALB2 | + | - | - | - |
| hsa-miR-22-3p | CALCR | + | + | - | + |
| hsa-miR-22-3p | CALD1 | - | - | - | + |
| hsa-miR-22-3p | CALM1 | - | - | - | + |
| hsa-miR-22-3p | CALM3 | - | - | - | + |
| hsa-miR-22-3p | CALN1 | + | - | - | + |
| hsa-miR-22-3p | CAMK2B | - | - | - | + |
| hsa-miR-22-3p | CAMK2D | - | - | - | + |
| hsa-miR-22-3p | CAMK2G | + | - | - | + |
| hsa-miR-22-3p | CAMK2N1 | + | - | + | - |
| hsa-miR-22-3p | CAMKK2 | - | - | - | + |
| hsa-miR-22-3p | CAMSAP2 | - | - | - | + |
| hsa-miR-22-3p | CAMTA2 | + | - | - | - |
| hsa-miR-22-3p | CANX | - | - | - | + |
| hsa-miR-22-3p | CAP1 | - | - | - | + |
| hsa-miR-22-3p | CAPN1 | + | - | - | + |
| hsa-miR-22-3p | CAPN6 | - | - | - | + |
| hsa-miR-22-3p | CAPNS1 | - | - | - | + |
| hsa-miR-22-3p | CARKD | - | - | - | + |
| hsa-miR-22-3p | CARNS1 | - | - | - | + |
| hsa-miR-22-3p | CASKIN2 | - | - | - | + |
| hsa-miR-22-3p | CASP10 | - | - | - | + |
| hsa-miR-22-3p | CASP14 | + | - | - | - |
| hsa-miR-22-3p | CATSPERG | + | - | - | - |
| hsa-miR-22-3p | CAV3 | + | + | - | + |
| hsa-miR-22-3p | CBFB | - | - | - | + |
| hsa-miR-22-3p | CBL | + | + | - | + |
| hsa-miR-22-3p | CBR1 | + | - | - | + |
| hsa-miR-22-3p | CBX2 | - | - | - | + |
| hsa-miR-22-3p | CBX5 | - | - | - | + |
| hsa-miR-22-3p | CBX6 | + | + | - | + |
| hsa-miR-22-3p | CBX7 | + | - | - | + |
| hsa-miR-22-3p | CC2D1B | - | - | - | + |
| hsa-miR-22-3p | CCBE1 | - | - | - | + |
| hsa-miR-22-3p | CCDC116 | - | - | - | + |
| hsa-miR-22-3p | CCDC136 | - | - | - | + |
| hsa-miR-22-3p | CCDC137 | + | - | - | + |
| hsa-miR-22-3p | CCDC149 | - | - | - | + |
| hsa-miR-22-3p | CCDC152 | - | - | - | + |
| hsa-miR-22-3p | CCDC181 | + | - | - | - |
| hsa-miR-22-3p | CCDC3 | + | - | - | - |
| hsa-miR-22-3p | CCDC40 | - | - | - | + |
| hsa-miR-22-3p | CCDC47 | - | + | - | + |
| hsa-miR-22-3p | CCDC50 | + | - | - | + |
| hsa-miR-22-3p | CCDC58 | - | - | - | + |
| hsa-miR-22-3p | CCDC64 | + | - | - | - |
| hsa-miR-22-3p | CCDC67 | + | + | - | - |
| hsa-miR-22-3p | CCDC69 | - | - | - | + |
| hsa-miR-22-3p | CCDC71L | - | - | - | + |
| hsa-miR-22-3p | CCDC74A | - | - | - | + |
| hsa-miR-22-3p | CCDC77 | - | - | - | + |
| hsa-miR-22-3p | CCDC85A | - | - | - | + |
| hsa-miR-22-3p | CCDC85C | + | - | - | - |
| hsa-miR-22-3p | CCDC87 | - | - | - | + |
| hsa-miR-22-3p | CCDC88B | - | - | - | + |
| hsa-miR-22-3p | CCDC93 | - | - | - | + |
| hsa-miR-22-3p | CCDC94 | - | - | - | + |
| hsa-miR-22-3p | CCDC97 | + | - | - | + |
| hsa-miR-22-3p | CCL2 | + | - | - | - |
| hsa-miR-22-3p | CCL4L1 | + | - | - | - |
| hsa-miR-22-3p | CCNA2 | - | + | - | + |
| hsa-miR-22-3p | CCND3 | - | - | - | + |
| hsa-miR-22-3p | CCNG2 | - | - | - | + |
| hsa-miR-22-3p | CCNI | + | - | - | + |
| hsa-miR-22-3p | CCNI2 | + | - | - | + |
| hsa-miR-22-3p | CCNJL | + | - | - | + |
| hsa-miR-22-3p | CCNL2 | + | - | - | - |
| hsa-miR-22-3p | CCNT1 | - | - | - | + |
| hsa-miR-22-3p | CCNT2 | + | - | + | + |
| hsa-miR-22-3p | CCPG1 | - | - | - | + |
| hsa-miR-22-3p | CCR4 | + | - | - | - |
| hsa-miR-22-3p | CCR7 | - | - | - | + |
| hsa-miR-22-3p | CCR9 | - | - | - | + |
| hsa-miR-22-3p | CD151 | + | - | + | + |
| hsa-miR-22-3p | CD1D | + | - | - | + |
| hsa-miR-22-3p | CD207 | + | - | - | + |
| hsa-miR-22-3p | CD244 | - | - | - | + |
| hsa-miR-22-3p | CD300A | - | - | - | + |
| hsa-miR-22-3p | CD300LG | - | - | - | + |
| hsa-miR-22-3p | CD302 | - | + | - | + |
| hsa-miR-22-3p | CD320 | + | - | - | - |
| hsa-miR-22-3p | CD3EAP | + | - | - | + |
| hsa-miR-22-3p | CD47 | - | - | - | + |
| hsa-miR-22-3p | CD5 | - | - | - | + |
| hsa-miR-22-3p | CD80 | + | + | - | + |
| hsa-miR-22-3p | CD84 | - | - | - | + |
| hsa-miR-22-3p | CD93 | - | - | - | + |
| hsa-miR-22-3p | CD97 | + | - | - | + |
| hsa-miR-22-3p | CD99L2 | - | - | - | + |
| hsa-miR-22-3p | CDADC1 | - | - | - | + |
| hsa-miR-22-3p | CDC14A | + | - | - | - |
| hsa-miR-22-3p | CDC25B | - | - | - | + |
| hsa-miR-22-3p | CDC42 | - | - | - | + |
| hsa-miR-22-3p | CDC42BPA | - | - | - | + |
| hsa-miR-22-3p | CDC42BPB | - | - | - | + |
| hsa-miR-22-3p | CDC42EP3 | + | - | - | + |
| hsa-miR-22-3p | CDC42SE2 | + | - | - | - |
| hsa-miR-22-3p | CDC5L | + | - | - | + |
| hsa-miR-22-3p | CDCA7 | - | - | - | + |
| hsa-miR-22-3p | CDCA7L | + | + | - | + |
| hsa-miR-22-3p | CDCP2 | + | - | - | - |
| hsa-miR-22-3p | CDH1 | + | + | - | - |
| hsa-miR-22-3p | CDH2 | - | - | - | + |
| hsa-miR-22-3p | CDH20 | - | - | - | + |
| hsa-miR-22-3p | CDH22 | - | - | - | + |
| hsa-miR-22-3p | CDK13 | - | - | - | + |
| hsa-miR-22-3p | CDK14 | + | + | - | + |
| hsa-miR-22-3p | CDK15 | + | - | - | + |
| hsa-miR-22-3p | CDK17 | + | - | - | - |
| hsa-miR-22-3p | CDK6 | - | - | + | + |
| hsa-miR-22-3p | CDKN1A | + | - | + | + |
| hsa-miR-22-3p | CDKN2AIP | + | - | - | - |
| hsa-miR-22-3p | CDKN2C | + | - | - | + |
| hsa-miR-22-3p | CDKN2D | - | - | - | + |
| hsa-miR-22-3p | CDON | - | - | - | + |
| hsa-miR-22-3p | CDPF1 | + | - | - | - |
| hsa-miR-22-3p | CDR2L | + | - | - | + |
| hsa-miR-22-3p | CDRT1 | + | - | - | - |
| hsa-miR-22-3p | CDT1 | - | - | - | + |
| hsa-miR-22-3p | CDX1 | - | - | - | + |
| hsa-miR-22-3p | CDX2 | + | + | - | - |
| hsa-miR-22-3p | CEACAM19 | - | - | - | + |
| hsa-miR-22-3p | CEBPD | + | - | - | - |
| hsa-miR-22-3p | CEBPG | - | - | - | + |
| hsa-miR-22-3p | CECR6 | - | - | - | + |
| hsa-miR-22-3p | CELF1 | - | - | - | + |
| hsa-miR-22-3p | CEMIP | - | - | - | + |
| hsa-miR-22-3p | CENPB | + | - | - | + |
| hsa-miR-22-3p | CENPBD1 | + | - | - | + |
| hsa-miR-22-3p | CENPE | - | - | - | + |
| hsa-miR-22-3p | CENPO | + | - | - | + |
| hsa-miR-22-3p | CENPV | + | + | - | - |
| hsa-miR-22-3p | CEP170 | - | - | - | + |
| hsa-miR-22-3p | CEP63 | - | - | - | + |
| hsa-miR-22-3p | CEP85 | - | - | - | + |
| hsa-miR-22-3p | CERK | + | - | - | + |
| hsa-miR-22-3p | CERS6 | - | - | - | + |
| hsa-miR-22-3p | CES3 | - | - | - | + |
| hsa-miR-22-3p | CFTR | + | - | - | - |
| hsa-miR-22-3p | CGA | - | - | - | + |
| hsa-miR-22-3p | CGGBP1 | - | - | - | + |
| hsa-miR-22-3p | CGNL1 | - | - | - | + |
| hsa-miR-22-3p | CHCHD3 | + | - | - | - |
| hsa-miR-22-3p | CHD2 | - | - | - | + |
| hsa-miR-22-3p | CHD4 | - | - | - | + |
| hsa-miR-22-3p | CHD5 | - | - | - | + |
| hsa-miR-22-3p | CHD7 | + | - | - | - |
| hsa-miR-22-3p | CHD8 | - | - | - | + |
| hsa-miR-22-3p | CHD9 | + | + | + | + |
| hsa-miR-22-3p | CHEK1 | - | - | - | + |
| hsa-miR-22-3p | CHGA | + | - | - | - |
| hsa-miR-22-3p | CHMP1A | + | - | - | + |
| hsa-miR-22-3p | CHMP3 | - | - | - | + |
| hsa-miR-22-3p | CHMP4C | - | - | - | + |
| hsa-miR-22-3p | CHMP6 | - | - | - | + |
| hsa-miR-22-3p | CHP1 | - | - | - | + |
| hsa-miR-22-3p | CHRM5 | + | - | - | - |
| hsa-miR-22-3p | CHRNA2 | + | - | - | + |
| hsa-miR-22-3p | CHRNB2 | - | - | - | + |
| hsa-miR-22-3p | CHST15 | + | - | - | - |
| hsa-miR-22-3p | CHST3 | - | - | - | + |
| hsa-miR-22-3p | CHST8 | - | - | - | + |
| hsa-miR-22-3p | CHSY3 | - | - | - | + |
| hsa-miR-22-3p | CHTF8 | - | - | - | + |
| hsa-miR-22-3p | CIAPIN1 | - | - | - | + |
| hsa-miR-22-3p | CIC | - | - | - | + |
| hsa-miR-22-3p | CIDEC | + | - | - | + |
| hsa-miR-22-3p | CIITA | + | + | - | - |
| hsa-miR-22-3p | CIPC | - | - | - | + |
| hsa-miR-22-3p | CISD1 | - | - | - | + |
| hsa-miR-22-3p | CISH | + | - | - | + |
| hsa-miR-22-3p | CKAP5 | - | - | - | + |
| hsa-miR-22-3p | CLASP1 | - | - | - | + |
| hsa-miR-22-3p | CLCA2 | - | - | - | + |
| hsa-miR-22-3p | CLCF1 | - | - | - | + |
| hsa-miR-22-3p | CLCN2 | + | - | - | - |
| hsa-miR-22-3p | CLCN5 | - | - | - | + |
| hsa-miR-22-3p | CLCN6 | - | - | - | + |
| hsa-miR-22-3p | CLCN7 | + | - | - | + |
| hsa-miR-22-3p | CLDN1 | - | - | - | + |
| hsa-miR-22-3p | CLDN18 | - | - | - | + |
| hsa-miR-22-3p | CLDN23 | - | - | - | + |
| hsa-miR-22-3p | CLDN8 | - | - | - | + |
| hsa-miR-22-3p | CLDND1 | - | + | - | + |
| hsa-miR-22-3p | CLEC3B | - | - | - | + |
| hsa-miR-22-3p | CLEC7A | + | - | - | - |
| hsa-miR-22-3p | CLGN | - | - | - | + |
| hsa-miR-22-3p | CLIC4 | - | + | - | + |
| hsa-miR-22-3p | CLIP2 | + | + | - | + |
| hsa-miR-22-3p | CLNK | + | - | - | - |
| hsa-miR-22-3p | CLPB | - | - | - | + |
| hsa-miR-22-3p | CLPTM1L | - | - | + | - |
| hsa-miR-22-3p | CLSTN3 | - | - | - | + |
| hsa-miR-22-3p | CLTC | - | - | - | + |
| hsa-miR-22-3p | CLVS1 | + | - | - | - |
| hsa-miR-22-3p | CMKLR1 | + | + | - | - |
| hsa-miR-22-3p | CMTM4 | - | - | - | + |
| hsa-miR-22-3p | CNGA2 | + | - | - | + |
| hsa-miR-22-3p | CNKSR2 | + | + | - | + |
| hsa-miR-22-3p | CNNM4 | - | - | - | + |
| hsa-miR-22-3p | CNOT6 | - | - | - | + |
| hsa-miR-22-3p | CNOT7 | - | - | - | + |
| hsa-miR-22-3p | CNPY4 | - | - | - | + |
| hsa-miR-22-3p | CNTLN | - | - | - | + |
| hsa-miR-22-3p | CNTN2 | - | - | - | + |
| hsa-miR-22-3p | CNTN3 | - | - | - | + |
| hsa-miR-22-3p | CNTN5 | - | - | - | + |
| hsa-miR-22-3p | CNTNAP3 | + | + | - | - |
| hsa-miR-22-3p | CNTNAP3B | - | + | - | - |
| hsa-miR-22-3p | CNTNAP5 | - | - | - | + |
| hsa-miR-22-3p | CNTRL | - | - | - | + |
| hsa-miR-22-3p | COA7 | - | + | - | - |
| hsa-miR-22-3p | COL12A1 | - | - | - | + |
| hsa-miR-22-3p | COL20A1 | + | - | - | + |
| hsa-miR-22-3p | COL4A4 | - | - | - | + |
| hsa-miR-22-3p | COL5A1 | - | - | - | + |
| hsa-miR-22-3p | COL8A2 | - | - | - | + |
| hsa-miR-22-3p | COMMD10 | - | - | - | + |
| hsa-miR-22-3p | COMMD5 | + | - | - | - |
| hsa-miR-22-3p | COPA | + | - | - | + |
| hsa-miR-22-3p | COPG2 | - | - | - | + |
| hsa-miR-22-3p | COPS7B | + | + | - | - |
| hsa-miR-22-3p | COPZ1 | - | - | - | + |
| hsa-miR-22-3p | COQ10B | - | - | - | + |
| hsa-miR-22-3p | CORO2B | - | - | - | + |
| hsa-miR-22-3p | COX10 | - | - | - | + |
| hsa-miR-22-3p | COX11 | - | - | - | + |
| hsa-miR-22-3p | CPA3 | - | - | - | + |
| hsa-miR-22-3p | CPD | - | - | - | + |
| hsa-miR-22-3p | CPEB1 | + | + | - | - |
| hsa-miR-22-3p | CPEB4 | - | - | - | + |
| hsa-miR-22-3p | CPLX3 | - | - | - | + |
| hsa-miR-22-3p | CPPED1 | - | - | - | + |
| hsa-miR-22-3p | CPSF3 | - | - | - | + |
| hsa-miR-22-3p | CPSF3L | + | - | - | - |
| hsa-miR-22-3p | CPSF4 | + | - | - | - |
| hsa-miR-22-3p | CPT1C | - | - | - | + |
| hsa-miR-22-3p | CREB1 | - | + | - | + |
| hsa-miR-22-3p | CREB3L2 | - | - | - | + |
| hsa-miR-22-3p | CREB5 | - | - | - | + |
| hsa-miR-22-3p | CREBBP | - | - | - | + |
| hsa-miR-22-3p | CREG1 | - | - | - | + |
| hsa-miR-22-3p | CRIM1 | - | - | - | + |
| hsa-miR-22-3p | CRKL | - | - | - | + |
| hsa-miR-22-3p | CRLS1 | - | - | - | + |
| hsa-miR-22-3p | CRNN | - | - | - | + |
| hsa-miR-22-3p | CROT | - | - | - | + |
| hsa-miR-22-3p | CRTC1 | - | + | - | - |
| hsa-miR-22-3p | CSDE1 | - | - | - | + |
| hsa-miR-22-3p | CSF1R | + | + | + | - |
| hsa-miR-22-3p | CSMD2 | + | + | - | - |
| hsa-miR-22-3p | CSNK1A1 | + | - | - | - |
| hsa-miR-22-3p | CSNK1D | - | - | - | + |
| hsa-miR-22-3p | CSNK1E | - | - | - | + |
| hsa-miR-22-3p | CSNK1G2 | - | - | - | + |
| hsa-miR-22-3p | CSNK2A1 | - | - | + | - |
| hsa-miR-22-3p | CSRNP2 | - | - | - | + |
| hsa-miR-22-3p | CSRNP3 | - | - | - | + |
| hsa-miR-22-3p | CST3 | + | - | - | - |
| hsa-miR-22-3p | CST7 | + | - | - | - |
| hsa-miR-22-3p | CSTF2T | - | - | - | + |
| hsa-miR-22-3p | CT45A4 | - | - | - | + |
| hsa-miR-22-3p | CTAG2 | - | - | - | + |
| hsa-miR-22-3p | CTC1 | - | - | + | - |
| hsa-miR-22-3p | CTD-2014B16.3 | + | - | - | - |
| hsa-miR-22-3p | CTD-2054N24.2 | + | - | - | - |
| hsa-miR-22-3p | CTDNEP1 | - | - | - | + |
| hsa-miR-22-3p | CTDP1 | - | - | - | + |
| hsa-miR-22-3p | CTDSP1 | - | + | - | + |
| hsa-miR-22-3p | CTDSPL | - | - | - | + |
| hsa-miR-22-3p | CTIF | + | + | - | + |
| hsa-miR-22-3p | CTNNA2 | - | - | - | + |
| hsa-miR-22-3p | CTNNB1 | - | - | - | + |
| hsa-miR-22-3p | CTNS | - | - | - | + |
| hsa-miR-22-3p | CTPS1 | - | - | - | + |
| hsa-miR-22-3p | CTRC | + | - | - | - |
| hsa-miR-22-3p | CTSC | - | - | - | + |
| hsa-miR-22-3p | CTTNBP2NL | - | - | - | + |
| hsa-miR-22-3p | CTXN3 | - | - | - | + |
| hsa-miR-22-3p | CUEDC1 | - | - | - | + |
| hsa-miR-22-3p | CUL3 | - | - | - | + |
| hsa-miR-22-3p | CUTC | - | - | - | + |
| hsa-miR-22-3p | CX3CL1 | + | - | - | + |
| hsa-miR-22-3p | CX3CR1 | - | - | - | + |
| hsa-miR-22-3p | CXCL9 | - | - | - | + |
| hsa-miR-22-3p | CXCR2 | + | - | - | + |
| hsa-miR-22-3p | CXXC4 | - | - | - | + |
| hsa-miR-22-3p | CXorf58 | + | + | - | + |
| hsa-miR-22-3p | CYB561D1 | - | - | - | + |
| hsa-miR-22-3p | CYB561D2 | + | - | - | + |
| hsa-miR-22-3p | CYB5D1 | + | - | - | + |
| hsa-miR-22-3p | CYCS | - | - | + | - |
| hsa-miR-22-3p | CYFIP1 | + | - | - | - |
| hsa-miR-22-3p | CYHR1 | - | + | - | + |
| hsa-miR-22-3p | CYP11B2 | - | - | - | + |
| hsa-miR-22-3p | CYP17A1 | - | - | - | + |
| hsa-miR-22-3p | CYP1A2 | - | - | - | + |
| hsa-miR-22-3p | CYP21A2 | - | - | - | + |
| hsa-miR-22-3p | CYP26A1 | - | - | - | + |
| hsa-miR-22-3p | CYP27B1 | - | - | - | + |
| hsa-miR-22-3p | CYP2A6 | - | - | - | + |
| hsa-miR-22-3p | CYP2A7 | - | - | - | + |
| hsa-miR-22-3p | CYR61 | + | - | + | + |
| hsa-miR-22-3p | CYS1 | + | - | - | + |
| hsa-miR-22-3p | CYTH1 | - | - | - | + |
| hsa-miR-22-3p | CYTH3 | + | + | - | + |
| hsa-miR-22-3p | CYTH4 | - | - | - | + |
| hsa-miR-22-3p | CYTIP | - | - | - | + |
| hsa-miR-22-3p | DACT2 | - | - | - | + |
| hsa-miR-22-3p | DAD1 | + | + | - | - |
| hsa-miR-22-3p | DAG1 | - | - | - | + |
| hsa-miR-22-3p | DAGLA | + | + | - | - |
| hsa-miR-22-3p | DAGLB | + | - | - | - |
| hsa-miR-22-3p | DAK | - | - | - | + |
| hsa-miR-22-3p | DAND5 | + | - | - | - |
| hsa-miR-22-3p | DAPL1 | - | - | - | + |
| hsa-miR-22-3p | DAW1 | + | - | - | - |
| hsa-miR-22-3p | DAZL | - | - | - | + |
| hsa-miR-22-3p | DBN1 | - | - | - | + |
| hsa-miR-22-3p | DBNDD1 | - | - | - | + |
| hsa-miR-22-3p | DCAF10 | - | - | - | + |
| hsa-miR-22-3p | DCAF12 | - | - | - | + |
| hsa-miR-22-3p | DCAF16 | + | - | + | + |
| hsa-miR-22-3p | DCAF17 | - | - | - | + |
| hsa-miR-22-3p | DCAF4L1 | + | - | - | + |
| hsa-miR-22-3p | DCAF4L2 | - | - | - | + |
| hsa-miR-22-3p | DCBLD2 | - | + | - | - |
| hsa-miR-22-3p | DCDC2B | - | - | - | + |
| hsa-miR-22-3p | DCHS1 | - | - | - | + |
| hsa-miR-22-3p | DCP2 | - | - | - | + |
| hsa-miR-22-3p | DCX | - | - | - | + |
| hsa-miR-22-3p | DDI1 | - | - | - | + |
| hsa-miR-22-3p | DDIT4 | + | + | + | + |
| hsa-miR-22-3p | DDN | - | - | - | + |
| hsa-miR-22-3p | DDR1 | - | - | - | + |
| hsa-miR-22-3p | DDX11 | + | - | - | - |
| hsa-miR-22-3p | DDX17 | - | - | - | + |
| hsa-miR-22-3p | DDX18 | - | - | - | + |
| hsa-miR-22-3p | DDX20 | - | - | - | + |
| hsa-miR-22-3p | DDX3X | - | - | - | + |
| hsa-miR-22-3p | DDX51 | - | - | - | + |
| hsa-miR-22-3p | DDX54 | - | - | - | + |
| hsa-miR-22-3p | DDX6 | - | - | + | - |
| hsa-miR-22-3p | DEFB118 | - | - | - | + |
| hsa-miR-22-3p | DENND1B | + | + | - | - |
| hsa-miR-22-3p | DENND4A | - | - | - | + |
| hsa-miR-22-3p | DERL1 | - | - | - | + |
| hsa-miR-22-3p | DERL3 | + | - | - | - |
| hsa-miR-22-3p | DFFB | + | - | - | - |
| hsa-miR-22-3p | DFNB31 | - | - | - | + |
| hsa-miR-22-3p | DGCR14 | + | + | - | + |
| hsa-miR-22-3p | DGCR6 | + | - | - | - |
| hsa-miR-22-3p | DGCR6L | + | - | - | - |
| hsa-miR-22-3p | DGCR8 | - | - | - | + |
| hsa-miR-22-3p | DGKE | + | + | - | + |
| hsa-miR-22-3p | DGKG | - | - | - | + |
| hsa-miR-22-3p | DGKI | - | + | - | - |
| hsa-miR-22-3p | DGKQ | + | + | - | + |
| hsa-miR-22-3p | DHCR24 | - | - | - | + |
| hsa-miR-22-3p | DHRS12 | - | - | - | + |
| hsa-miR-22-3p | DHX33 | - | - | - | + |
| hsa-miR-22-3p | DHX40 | - | - | - | + |
| hsa-miR-22-3p | DHX57 | + | - | - | + |
| hsa-miR-22-3p | DIAPH1 | - | - | - | + |
| hsa-miR-22-3p | DIAPH3 | - | - | - | + |
| hsa-miR-22-3p | DIP2B | - | - | - | + |
| hsa-miR-22-3p | DIP2C | - | - | - | + |
| hsa-miR-22-3p | DIRAS1 | + | - | - | + |
| hsa-miR-22-3p | DISC1 | - | - | - | + |
| hsa-miR-22-3p | DKFZP434H0512 | + | - | - | - |
| hsa-miR-22-3p | DKK4 | - | - | - | + |
| hsa-miR-22-3p | DLEC1 | - | - | - | + |
| hsa-miR-22-3p | DLG1 | - | - | - | + |
| hsa-miR-22-3p | DLG2 | - | - | - | + |
| hsa-miR-22-3p | DLGAP2 | + | - | - | + |
| hsa-miR-22-3p | DLGAP4 | - | - | - | + |
| hsa-miR-22-3p | DLL4 | + | - | - | + |
| hsa-miR-22-3p | DLX1 | - | - | - | + |
| hsa-miR-22-3p | DMD | - | - | - | + |
| hsa-miR-22-3p | DMKN | - | - | - | + |
| hsa-miR-22-3p | DMRT2 | - | - | - | + |
| hsa-miR-22-3p | DMXL1 | - | - | - | + |
| hsa-miR-22-3p | DNAH17-AS1 | + | - | - | - |
| hsa-miR-22-3p | DNAH7 | + | - | - | - |
| hsa-miR-22-3p | DNAI2 | + | - | - | - |
| hsa-miR-22-3p | DNAJA3 | - | - | - | + |
| hsa-miR-22-3p | DNAJB12 | + | - | - | + |
| hsa-miR-22-3p | DNAJB14 | - | - | - | + |
| hsa-miR-22-3p | DNAJB2 | - | - | - | + |
| hsa-miR-22-3p | DNAJB5 | + | + | - | - |
| hsa-miR-22-3p | DNAJC11 | - | - | - | + |
| hsa-miR-22-3p | DNAJC16 | + | - | - | - |
| hsa-miR-22-3p | DNAJC27 | + | + | - | + |
| hsa-miR-22-3p | DNAJC3 | - | + | - | - |
| hsa-miR-22-3p | DNAJC5 | + | + | - | - |
| hsa-miR-22-3p | DNAJC7 | + | - | - | - |
| hsa-miR-22-3p | DNAJC9-AS1 | - | - | - | + |
| hsa-miR-22-3p | DNAL4 | + | - | - | + |
| hsa-miR-22-3p | DND1 | + | - | - | + |
| hsa-miR-22-3p | DNER | - | - | - | + |
| hsa-miR-22-3p | DNHD1 | - | - | + | - |
| hsa-miR-22-3p | DNM3 | + | + | - | + |
| hsa-miR-22-3p | DNMT3A | - | - | - | + |
| hsa-miR-22-3p | DNPH1 | + | - | - | - |
| hsa-miR-22-3p | DOC2A | + | - | - | + |
| hsa-miR-22-3p | DOCK3 | - | - | - | + |
| hsa-miR-22-3p | DOK2 | + | - | - | - |
| hsa-miR-22-3p | DOK3 | - | - | - | + |
| hsa-miR-22-3p | DOK6 | - | - | - | + |
| hsa-miR-22-3p | DOK7 | - | - | - | + |
| hsa-miR-22-3p | DOT1L | - | - | - | + |
| hsa-miR-22-3p | DPF2 | + | + | - | + |
| hsa-miR-22-3p | DPM2 | + | + | - | + |
| hsa-miR-22-3p | DPP10 | + | + | - | + |
| hsa-miR-22-3p | DPP4 | + | - | - | - |
| hsa-miR-22-3p | DPPA4 | - | - | - | + |
| hsa-miR-22-3p | DPY19L2 | - | - | - | + |
| hsa-miR-22-3p | DPY30 | + | + | - | - |
| hsa-miR-22-3p | DPYSL3 | + | - | - | + |
| hsa-miR-22-3p | DRD1 | - | - | - | + |
| hsa-miR-22-3p | DRP2 | + | - | - | - |
| hsa-miR-22-3p | DSCAM | - | - | - | + |
| hsa-miR-22-3p | DSCR4 | + | - | - | + |
| hsa-miR-22-3p | DST | + | - | - | + |
| hsa-miR-22-3p | DSTYK | - | + | - | - |
| hsa-miR-22-3p | DTL | - | - | - | + |
| hsa-miR-22-3p | DTNA | - | - | - | + |
| hsa-miR-22-3p | DTX1 | - | - | - | + |
| hsa-miR-22-3p | DTX2 | + | - | - | + |
| hsa-miR-22-3p | DUSP10 | + | + | - | - |
| hsa-miR-22-3p | DUSP13 | + | - | - | + |
| hsa-miR-22-3p | DUSP18 | + | - | - | + |
| hsa-miR-22-3p | DUSP19 | - | - | - | + |
| hsa-miR-22-3p | DUSP26 | + | - | - | + |
| hsa-miR-22-3p | DYNC1H1 | - | - | - | + |
| hsa-miR-22-3p | DYNC1LI2 | - | - | - | + |
| hsa-miR-22-3p | DYRK3 | - | - | - | + |
| hsa-miR-22-3p | E2F2 | - | - | + | + |
| hsa-miR-22-3p | EBF1 | - | - | - | + |
| hsa-miR-22-3p | EBF2 | - | - | - | + |
| hsa-miR-22-3p | EDA | - | + | - | + |
| hsa-miR-22-3p | EDAR | - | - | - | + |
| hsa-miR-22-3p | EDARADD | + | + | - | + |
| hsa-miR-22-3p | EDC3 | - | + | + | + |
| hsa-miR-22-3p | EDEM1 | - | - | - | + |
| hsa-miR-22-3p | EDNRA | + | - | - | - |
| hsa-miR-22-3p | EEA1 | - | - | - | + |
| hsa-miR-22-3p | EEF2K | + | + | - | + |
| hsa-miR-22-3p | EEPD1 | - | - | - | + |
| hsa-miR-22-3p | EFCAB1 | + | - | - | + |
| hsa-miR-22-3p | EFCAB14 | - | + | - | + |
| hsa-miR-22-3p | EFCAB3 | - | - | - | + |
| hsa-miR-22-3p | EFHC1 | - | - | - | + |
| hsa-miR-22-3p | EFNA5 | + | - | - | + |
| hsa-miR-22-3p | EFNB2 | - | - | - | + |
| hsa-miR-22-3p | EFR3A | - | - | - | + |
| hsa-miR-22-3p | EFR3B | + | + | + | - |
| hsa-miR-22-3p | EFS | - | - | - | + |
| hsa-miR-22-3p | EGLN2 | - | - | - | + |
| hsa-miR-22-3p | EHBP1 | - | - | - | + |
| hsa-miR-22-3p | EHD1 | - | + | - | - |
| hsa-miR-22-3p | EI24 | + | - | - | - |
| hsa-miR-22-3p | EID1 | - | - | - | + |
| hsa-miR-22-3p | EID3 | - | - | - | + |
| hsa-miR-22-3p | EIF1B | - | - | - | + |
| hsa-miR-22-3p | EIF2AK1 | + | - | - | + |
| hsa-miR-22-3p | EIF2D | - | - | - | + |
| hsa-miR-22-3p | EIF4E2 | + | - | - | + |
| hsa-miR-22-3p | EIF4EBP2 | + | - | - | + |
| hsa-miR-22-3p | EIF4EBP3 | + | - | - | + |
| hsa-miR-22-3p | EIF4G3 | - | - | - | + |
| hsa-miR-22-3p | EIF4H | - | - | - | + |
| hsa-miR-22-3p | EIF5A2 | - | - | - | + |
| hsa-miR-22-3p | EIF5AL1 | - | - | - | + |
| hsa-miR-22-3p | ELAVL1 | - | + | - | + |
| hsa-miR-22-3p | ELAVL2 | - | - | - | + |
| hsa-miR-22-3p | ELAVL4 | - | - | - | + |
| hsa-miR-22-3p | ELF3 | - | - | - | + |
| hsa-miR-22-3p | ELF4 | - | - | - | + |
| hsa-miR-22-3p | ELF5 | + | + | - | - |
| hsa-miR-22-3p | ELK1 | - | - | - | + |
| hsa-miR-22-3p | ELL2 | - | - | - | + |
| hsa-miR-22-3p | ELL3 | + | - | - | + |
| hsa-miR-22-3p | ELMOD3 | + | - | - | - |
| hsa-miR-22-3p | ELMSAN1 | - | + | - | + |
| hsa-miR-22-3p | ELOVL1 | + | - | - | - |
| hsa-miR-22-3p | ELOVL2 | + | + | - | + |
| hsa-miR-22-3p | ELOVL6 | + | + | - | - |
| hsa-miR-22-3p | ELP5 | - | - | + | - |
| hsa-miR-22-3p | ELTD1 | - | - | - | + |
| hsa-miR-22-3p | EMB | - | - | - | + |
| hsa-miR-22-3p | EMC7 | - | - | - | + |
| hsa-miR-22-3p | EMCN | + | - | - | + |
| hsa-miR-22-3p | EMILIN3 | + | + | - | + |
| hsa-miR-22-3p | EMP1 | + | + | - | - |
| hsa-miR-22-3p | EMR1 | - | - | - | + |
| hsa-miR-22-3p | EMR2 | + | - | - | - |
| hsa-miR-22-3p | EMX2 | + | + | - | - |
| hsa-miR-22-3p | ENAH | - | - | - | + |
| hsa-miR-22-3p | ENDOD1 | - | - | - | + |
| hsa-miR-22-3p | ENO1 | + | + | - | - |
| hsa-miR-22-3p | ENPP1 | - | - | - | + |
| hsa-miR-22-3p | ENSA | - | - | - | + |
| hsa-miR-22-3p | ENTHD2 | + | - | - | + |
| hsa-miR-22-3p | ENTPD1 | - | - | - | + |
| hsa-miR-22-3p | ENTPD4 | - | - | - | + |
| hsa-miR-22-3p | ENTPD5 | - | - | - | + |
| hsa-miR-22-3p | EOMES | - | - | - | + |
| hsa-miR-22-3p | EP300 | + | + | - | + |
| hsa-miR-22-3p | EPAS1 | - | - | - | + |
| hsa-miR-22-3p | EPB41 | + | - | - | - |
| hsa-miR-22-3p | EPB41L1 | - | - | - | + |
| hsa-miR-22-3p | EPB41L2 | + | + | - | + |
| hsa-miR-22-3p | EPB41L4B | - | + | - | + |
| hsa-miR-22-3p | EPB41L5 | - | - | - | + |
| hsa-miR-22-3p | EPC1 | + | + | - | + |
| hsa-miR-22-3p | EPCAM | - | - | - | + |
| hsa-miR-22-3p | EPG5 | - | - | - | + |
| hsa-miR-22-3p | EPHA10 | + | - | - | + |
| hsa-miR-22-3p | EPHA3 | - | - | - | + |
| hsa-miR-22-3p | EPHA7 | - | - | - | + |
| hsa-miR-22-3p | EPM2A | + | - | - | + |
| hsa-miR-22-3p | EPN2 | - | - | - | + |
| hsa-miR-22-3p | EPO | - | - | - | + |
| hsa-miR-22-3p | EPPIN | - | - | - | + |
| hsa-miR-22-3p | EPX | - | - | - | + |
| hsa-miR-22-3p | ERAP2 | - | - | - | + |
| hsa-miR-22-3p | ERBB2IP | - | - | - | + |
| hsa-miR-22-3p | ERBB3 | + | + | + | - |
| hsa-miR-22-3p | ERBB4 | - | - | - | + |
| hsa-miR-22-3p | ERC2 | - | - | - | + |
| hsa-miR-22-3p | ERCC1 | - | - | - | + |
| hsa-miR-22-3p | EREG | - | - | - | + |
| hsa-miR-22-3p | ERF | - | - | - | + |
| hsa-miR-22-3p | ERGIC1 | + | - | - | - |
| hsa-miR-22-3p | ERI2 | + | + | - | - |
| hsa-miR-22-3p | ERLIN2 | + | - | - | - |
| hsa-miR-22-3p | ERO1LB | - | - | - | + |
| hsa-miR-22-3p | ERVFRD-1 | - | - | - | + |
| hsa-miR-22-3p | ESPNL | - | - | - | + |
| hsa-miR-22-3p | ESR1 | + | + | + | - |
| hsa-miR-22-3p | ESRP1 | - | - | - | + |
| hsa-miR-22-3p | ESRRB | + | - | - | + |
| hsa-miR-22-3p | ESRRG | - | - | - | + |
| hsa-miR-22-3p | ESYT1 | - | - | - | + |
| hsa-miR-22-3p | ETF1 | - | - | - | + |
| hsa-miR-22-3p | ETV1 | + | - | - | + |
| hsa-miR-22-3p | EVI5 | - | - | - | + |
| hsa-miR-22-3p | EVI5L | + | - | - | + |
| hsa-miR-22-3p | EVPLL | + | - | - | + |
| hsa-miR-22-3p | EVX2 | - | - | - | + |
| hsa-miR-22-3p | EXD1 | + | - | - | + |
| hsa-miR-22-3p | EXO1 | - | - | - | + |
| hsa-miR-22-3p | EXOC5 | + | - | - | - |
| hsa-miR-22-3p | EXOG | + | - | - | - |
| hsa-miR-22-3p | EXPH5 | - | - | - | + |
| hsa-miR-22-3p | EXT2 | - | - | - | + |
| hsa-miR-22-3p | EXTL1 | - | - | - | + |
| hsa-miR-22-3p | EYA3 | - | - | - | + |
| hsa-miR-22-3p | EYA4 | - | - | - | + |
| hsa-miR-22-3p | EZH1 | + | - | - | + |
| hsa-miR-22-3p | EZR | - | - | - | + |
| hsa-miR-22-3p | F8A2 | - | - | - | + |
| hsa-miR-22-3p | FA2H | - | - | - | + |
| hsa-miR-22-3p | FABP5 | + | - | - | - |
| hsa-miR-22-3p | FADD | - | - | - | + |
| hsa-miR-22-3p | FAF2 | + | - | - | + |
| hsa-miR-22-3p | FAIM2 | - | - | - | + |
| hsa-miR-22-3p | FAM102A | - | - | - | + |
| hsa-miR-22-3p | FAM104A | - | - | - | + |
| hsa-miR-22-3p | FAM105B | - | - | - | + |
| hsa-miR-22-3p | FAM106A | + | - | - | + |
| hsa-miR-22-3p | FAM107A | - | - | - | + |
| hsa-miR-22-3p | FAM108C1 | - | - | - | + |
| hsa-miR-22-3p | FAM110A | - | - | - | + |
| hsa-miR-22-3p | FAM110C | - | - | - | + |
| hsa-miR-22-3p | FAM114A1 | - | - | - | + |
| hsa-miR-22-3p | FAM115C | - | - | - | + |
| hsa-miR-22-3p | FAM117A | + | - | - | + |
| hsa-miR-22-3p | FAM117B | - | - | - | + |
| hsa-miR-22-3p | FAM122B | - | - | - | + |
| hsa-miR-22-3p | FAM123A | - | - | - | + |
| hsa-miR-22-3p | FAM129C | + | - | - | + |
| hsa-miR-22-3p | FAM131B | + | + | - | - |
| hsa-miR-22-3p | FAM134A | - | - | - | + |
| hsa-miR-22-3p | FAM13A | - | - | - | + |
| hsa-miR-22-3p | FAM155B | - | - | - | + |
| hsa-miR-22-3p | FAM160B1 | - | - | - | + |
| hsa-miR-22-3p | FAM163A | + | - | - | + |
| hsa-miR-22-3p | FAM168A | - | - | - | + |
| hsa-miR-22-3p | FAM168B | - | - | - | + |
| hsa-miR-22-3p | FAM172A | - | - | - | + |
| hsa-miR-22-3p | FAM175B | - | - | - | + |
| hsa-miR-22-3p | FAM178A | + | - | - | - |
| hsa-miR-22-3p | FAM189A1 | + | + | - | + |
| hsa-miR-22-3p | FAM190B | - | - | - | + |
| hsa-miR-22-3p | FAM192A | - | + | - | + |
| hsa-miR-22-3p | FAM199X | - | - | - | + |
| hsa-miR-22-3p | FAM210A | + | - | - | - |
| hsa-miR-22-3p | FAM213B | - | - | - | + |
| hsa-miR-22-3p | FAM216B | + | - | - | + |
| hsa-miR-22-3p | FAM217B | - | - | - | + |
| hsa-miR-22-3p | FAM219A | - | - | - | + |
| hsa-miR-22-3p | FAM21D | + | - | - | - |
| hsa-miR-22-3p | FAM22D | - | - | - | + |
| hsa-miR-22-3p | FAM3C | - | - | - | + |
| hsa-miR-22-3p | FAM48A | - | - | - | + |
| hsa-miR-22-3p | FAM49B | + | + | - | + |
| hsa-miR-22-3p | FAM50A | + | - | - | - |
| hsa-miR-22-3p | FAM53C | + | + | - | + |
| hsa-miR-22-3p | FAM54B | - | - | - | + |
| hsa-miR-22-3p | FAM63B | - | - | - | + |
| hsa-miR-22-3p | FAM71B | - | - | - | + |
| hsa-miR-22-3p | FAM81A | - | - | - | + |
| hsa-miR-22-3p | FAM83A | + | - | - | + |
| hsa-miR-22-3p | FAM83F | + | - | - | - |
| hsa-miR-22-3p | FAM83H | - | - | - | + |
| hsa-miR-22-3p | FAM84A | - | - | - | + |
| hsa-miR-22-3p | FAM84B | - | - | - | + |
| hsa-miR-22-3p | FAM89A | - | - | - | + |
| hsa-miR-22-3p | FAM89B | + | - | - | + |
| hsa-miR-22-3p | FAM8A1 | - | - | - | + |
| hsa-miR-22-3p | FAM96A | + | - | - | - |
| hsa-miR-22-3p | FANCC | - | - | - | + |
| hsa-miR-22-3p | FAS | - | + | - | + |
| hsa-miR-22-3p | FASTKD5 | - | - | - | + |
| hsa-miR-22-3p | FAT4 | - | - | - | + |
| hsa-miR-22-3p | FBLN5 | - | - | - | + |
| hsa-miR-22-3p | FBN2 | - | - | - | + |
| hsa-miR-22-3p | FBN3 | - | - | - | + |
| hsa-miR-22-3p | FBRS | + | + | - | - |
| hsa-miR-22-3p | FBXL16 | - | - | - | + |
| hsa-miR-22-3p | FBXL19 | + | + | - | + |
| hsa-miR-22-3p | FBXO11 | - | - | - | + |
| hsa-miR-22-3p | FBXO17 | - | - | - | + |
| hsa-miR-22-3p | FBXO28 | - | - | - | + |
| hsa-miR-22-3p | FBXO41 | + | + | - | + |
| hsa-miR-22-3p | FBXO45 | - | - | - | + |
| hsa-miR-22-3p | FBXO46 | + | - | - | + |
| hsa-miR-22-3p | FBXW12 | - | - | - | + |
| hsa-miR-22-3p | FBXW7 | + | + | - | + |
| hsa-miR-22-3p | FCER1A | - | - | - | + |
| hsa-miR-22-3p | FCER1G | - | - | - | + |
| hsa-miR-22-3p | FCER2 | + | - | - | + |
| hsa-miR-22-3p | FCGR2A | - | - | - | + |
| hsa-miR-22-3p | FCHO2 | - | - | - | + |
| hsa-miR-22-3p | FCHSD1 | - | - | - | + |
| hsa-miR-22-3p | FCN2 | - | - | - | + |
| hsa-miR-22-3p | FCN3 | - | - | - | + |
| hsa-miR-22-3p | FCRL4 | - | - | - | + |
| hsa-miR-22-3p | FDX1 | - | - | - | + |
| hsa-miR-22-3p | FEM1B | - | + | - | + |
| hsa-miR-22-3p | FFAR3 | - | - | - | + |
| hsa-miR-22-3p | FGD1 | - | - | - | + |
| hsa-miR-22-3p | FGD6 | - | + | - | + |
| hsa-miR-22-3p | FGF23 | - | - | - | + |
| hsa-miR-22-3p | FGF5 | - | - | - | + |
| hsa-miR-22-3p | FGFBP2 | + | - | - | + |
| hsa-miR-22-3p | FGFR1 | - | + | - | + |
| hsa-miR-22-3p | FGFR1OP2 | - | - | - | + |
| hsa-miR-22-3p | FGFR2 | - | + | - | + |
| hsa-miR-22-3p | FGFR3 | - | - | - | + |
| hsa-miR-22-3p | FGR | - | - | - | + |
| hsa-miR-22-3p | FHDC1 | - | - | - | + |
| hsa-miR-22-3p | FIBCD1 | + | + | - | + |
| hsa-miR-22-3p | FIGF | - | - | - | + |
| hsa-miR-22-3p | FITM1 | - | - | - | + |
| hsa-miR-22-3p | FJX1 | - | - | - | + |
| hsa-miR-22-3p | FKBP1A | - | - | - | + |
| hsa-miR-22-3p | FKBP5 | - | - | + | + |
| hsa-miR-22-3p | FKRP | + | - | - | + |
| hsa-miR-22-3p | FKTN | - | - | - | + |
| hsa-miR-22-3p | FLG | - | - | - | + |
| hsa-miR-22-3p | FLG2 | - | - | - | + |
| hsa-miR-22-3p | FLI1 | - | - | - | + |
| hsa-miR-22-3p | FLJ00418 | + | - | - | - |
| hsa-miR-22-3p | FMN1 | + | - | - | + |
| hsa-miR-22-3p | FMNL2 | + | + | - | - |
| hsa-miR-22-3p | FMNL3 | - | - | - | + |
| hsa-miR-22-3p | FMOD | + | - | - | + |
| hsa-miR-22-3p | FNBP1 | - | - | - | + |
| hsa-miR-22-3p | FNBP4 | + | + | - | + |
| hsa-miR-22-3p | FNDC5 | - | - | - | + |
| hsa-miR-22-3p | FNIP2 | - | - | - | + |
| hsa-miR-22-3p | FOCAD | - | - | - | + |
| hsa-miR-22-3p | FOSL1 | + | + | - | + |
| hsa-miR-22-3p | FOXC1 | - | - | - | + |
| hsa-miR-22-3p | FOXD4L1 | - | - | - | + |
| hsa-miR-22-3p | FOXE3 | - | - | - | + |
| hsa-miR-22-3p | FOXK1 | - | - | - | + |
| hsa-miR-22-3p | FOXL1 | + | - | - | + |
| hsa-miR-22-3p | FOXN3 | + | - | - | + |
| hsa-miR-22-3p | FOXO3 | - | - | - | + |
| hsa-miR-22-3p | FOXP1 | - | - | + | + |
| hsa-miR-22-3p | FOXP4 | - | - | - | + |
| hsa-miR-22-3p | FPR2 | - | - | - | + |
| hsa-miR-22-3p | FRAS1 | - | - | - | + |
| hsa-miR-22-3p | FRAT1 | - | - | - | + |
| hsa-miR-22-3p | FRAT2 | + | + | + | + |
| hsa-miR-22-3p | FREM2 | - | - | - | + |
| hsa-miR-22-3p | FRG2 | - | - | - | + |
| hsa-miR-22-3p | FRMD6 | + | - | - | + |
| hsa-miR-22-3p | FRMD8 | - | - | - | + |
| hsa-miR-22-3p | FRMPD4 | - | - | - | + |
| hsa-miR-22-3p | FRS2 | - | - | - | + |
| hsa-miR-22-3p | FSD1L | - | - | - | + |
| hsa-miR-22-3p | FSD2 | + | + | - | + |
| hsa-miR-22-3p | FSTL1 | + | - | - | - |
| hsa-miR-22-3p | FSTL3 | - | - | - | + |
| hsa-miR-22-3p | FTL | + | + | - | + |
| hsa-miR-22-3p | FUBP1 | - | - | + | - |
| hsa-miR-22-3p | FUOM | + | - | - | + |
| hsa-miR-22-3p | FURIN | + | + | - | + |
| hsa-miR-22-3p | FUT8 | - | - | - | + |
| hsa-miR-22-3p | FUT9 | + | + | - | - |
| hsa-miR-22-3p | FXR2 | - | - | - | + |
| hsa-miR-22-3p | FXYD5 | + | - | - | - |
| hsa-miR-22-3p | FYCO1 | - | - | - | + |
| hsa-miR-22-3p | FZD3 | - | - | - | + |
| hsa-miR-22-3p | FZD5 | - | - | - | + |
| hsa-miR-22-3p | FZD6 | + | - | - | - |
| hsa-miR-22-3p | FZD9 | - | - | - | + |
| hsa-miR-22-3p | G3BP2 | - | - | - | + |
| hsa-miR-22-3p | GAB1 | - | - | - | + |
| hsa-miR-22-3p | GAB4 | + | - | - | - |
| hsa-miR-22-3p | GABBR2 | + | + | - | + |
| hsa-miR-22-3p | GABPA | - | - | - | + |
| hsa-miR-22-3p | GABRA5 | - | - | - | + |
| hsa-miR-22-3p | GABRA6 | - | - | - | + |
| hsa-miR-22-3p | GABRG1 | - | - | - | + |
| hsa-miR-22-3p | GABRR2 | + | - | - | - |
| hsa-miR-22-3p | GALNT10 | + | - | - | + |
| hsa-miR-22-3p | GALNT2 | - | - | - | + |
| hsa-miR-22-3p | GALNT3 | + | - | - | + |
| hsa-miR-22-3p | GALNT4 | + | + | - | + |
| hsa-miR-22-3p | GALNT6 | - | - | - | + |
| hsa-miR-22-3p | GALNT8 | - | - | - | + |
| hsa-miR-22-3p | GALNTL2 | - | - | - | + |
| hsa-miR-22-3p | GAS7 | - | - | - | + |
| hsa-miR-22-3p | GATA5 | - | - | - | + |
| hsa-miR-22-3p | GATAD2B | - | - | - | + |
| hsa-miR-22-3p | GATM | + | + | - | + |
| hsa-miR-22-3p | GBA | + | - | - | - |
| hsa-miR-22-3p | GBE1 | - | - | - | + |
| hsa-miR-22-3p | GBX2 | + | - | - | - |
| hsa-miR-22-3p | GCG | - | - | - | + |
| hsa-miR-22-3p | GCHFR | + | - | - | - |
| hsa-miR-22-3p | GCK | - | - | - | + |
| hsa-miR-22-3p | GCSAM | - | - | - | + |
| hsa-miR-22-3p | GDAP1 | + | - | - | + |
| hsa-miR-22-3p | GDE1 | - | - | - | + |
| hsa-miR-22-3p | GDF11 | - | - | - | + |
| hsa-miR-22-3p | GEN1 | - | - | - | + |
| hsa-miR-22-3p | GFPT2 | - | - | - | + |
| hsa-miR-22-3p | GFRA1 | - | - | - | + |
| hsa-miR-22-3p | GGACT | + | - | - | - |
| hsa-miR-22-3p | GGT6 | - | - | - | + |
| hsa-miR-22-3p | GHDC | - | - | - | + |
| hsa-miR-22-3p | GHITM | - | - | - | + |
| hsa-miR-22-3p | GHRHR | + | - | - | - |
| hsa-miR-22-3p | GID4 | - | - | - | + |
| hsa-miR-22-3p | GIGYF1 | - | - | - | + |
| hsa-miR-22-3p | GIGYF2 | + | + | - | - |
| hsa-miR-22-3p | GIMAP1 | - | - | - | + |
| hsa-miR-22-3p | GINS2 | + | - | + | - |
| hsa-miR-22-3p | GIPC3 | - | - | - | + |
| hsa-miR-22-3p | GIT1 | - | - | - | + |
| hsa-miR-22-3p | GJA1 | - | - | - | + |
| hsa-miR-22-3p | GJA9 | + | - | - | - |
| hsa-miR-22-3p | GJC1 | - | - | - | + |
| hsa-miR-22-3p | GJC2 | - | - | - | + |
| hsa-miR-22-3p | GLB1 | - | - | - | + |
| hsa-miR-22-3p | GLDN | + | - | - | + |
| hsa-miR-22-3p | GLI3 | - | - | - | + |
| hsa-miR-22-3p | GLIPR2 | - | - | - | + |
| hsa-miR-22-3p | GLIS2 | + | - | + | + |
| hsa-miR-22-3p | GLIS3 | - | - | - | + |
| hsa-miR-22-3p | GLRA3 | + | - | - | - |
| hsa-miR-22-3p | GLUD1 | - | - | - | + |
| hsa-miR-22-3p | GLYAT | - | - | - | + |
| hsa-miR-22-3p | GM2A | + | + | - | + |
| hsa-miR-22-3p | GMEB2 | - | - | - | + |
| hsa-miR-22-3p | GNA11 | + | - | - | + |
| hsa-miR-22-3p | GNAI1 | - | - | - | + |
| hsa-miR-22-3p | GNAI3 | + | + | - | - |
| hsa-miR-22-3p | GNAQ | - | - | - | + |
| hsa-miR-22-3p | GNB4 | - | + | - | - |
| hsa-miR-22-3p | GNG12 | + | - | - | + |
| hsa-miR-22-3p | GNG2 | - | - | - | + |
| hsa-miR-22-3p | GNL1 | - | - | - | + |
| hsa-miR-22-3p | GNLY | - | - | - | + |
| hsa-miR-22-3p | GNPAT | - | - | - | + |
| hsa-miR-22-3p | GNPDA1 | + | - | - | + |
| hsa-miR-22-3p | GNRHR2 | - | - | - | + |
| hsa-miR-22-3p | GOLGA1 | + | + | - | + |
| hsa-miR-22-3p | GOLGA2 | - | - | - | + |
| hsa-miR-22-3p | GOLGA6C | + | - | - | - |
| hsa-miR-22-3p | GOLGA6D | + | - | - | - |
| hsa-miR-22-3p | GOLGA6L10 | + | - | - | - |
| hsa-miR-22-3p | GOLGA7B | - | - | - | + |
| hsa-miR-22-3p | GOLGA8F | + | - | - | - |
| hsa-miR-22-3p | GOLGA8G | + | - | - | - |
| hsa-miR-22-3p | GOLGA8H | + | - | - | - |
| hsa-miR-22-3p | GOLGA8I | + | - | - | - |
| hsa-miR-22-3p | GOLGA8J | + | - | - | - |
| hsa-miR-22-3p | GOLGA8K | + | - | - | - |
| hsa-miR-22-3p | GOLGA8M | + | - | - | - |
| hsa-miR-22-3p | GOLGA8O | + | - | - | - |
| hsa-miR-22-3p | GOLGA8R | + | - | - | - |
| hsa-miR-22-3p | GOLPH3 | - | - | - | + |
| hsa-miR-22-3p | GP9 | + | - | - | + |
| hsa-miR-22-3p | GPATCH2L | - | - | - | + |
| hsa-miR-22-3p | GPATCH4 | + | - | - | + |
| hsa-miR-22-3p | GPBP1 | - | + | - | - |
| hsa-miR-22-3p | GPBP1L1 | - | - | - | + |
| hsa-miR-22-3p | GPCPD1 | - | - | - | + |
| hsa-miR-22-3p | GPD1 | + | - | - | + |
| hsa-miR-22-3p | GPD1L | - | - | - | + |
| hsa-miR-22-3p | GPM6A | - | - | - | + |
| hsa-miR-22-3p | GPNMB | + | - | - | - |
| hsa-miR-22-3p | GPR107 | + | + | - | + |
| hsa-miR-22-3p | GPR132 | + | - | - | - |
| hsa-miR-22-3p | GPR161 | + | - | - | - |
| hsa-miR-22-3p | GPR162 | + | - | - | - |
| hsa-miR-22-3p | GPR171 | - | - | - | + |
| hsa-miR-22-3p | GPR173 | - | - | - | + |
| hsa-miR-22-3p | GPR180 | - | - | - | + |
| hsa-miR-22-3p | GPR31 | + | - | - | - |
| hsa-miR-22-3p | GPR55 | + | - | - | + |
| hsa-miR-22-3p | GPR61 | + | - | - | - |
| hsa-miR-22-3p | GPR62 | - | - | - | + |
| hsa-miR-22-3p | GPR88 | - | - | - | + |
| hsa-miR-22-3p | GPSM2 | + | + | - | - |
| hsa-miR-22-3p | GPX3 | - | - | - | + |
| hsa-miR-22-3p | GPX6 | - | - | - | + |
| hsa-miR-22-3p | GRAMD1B | - | - | - | + |
| hsa-miR-22-3p | GRAP2 | - | - | - | + |
| hsa-miR-22-3p | GREB1 | - | - | - | + |
| hsa-miR-22-3p | GRIA1 | - | - | - | + |
| hsa-miR-22-3p | GRID1 | - | - | - | + |
| hsa-miR-22-3p | GRIK3 | - | - | - | + |
| hsa-miR-22-3p | GRIN2A | - | - | - | + |
| hsa-miR-22-3p | GRIN2B | + | - | - | - |
| hsa-miR-22-3p | GRM1 | - | - | - | + |
| hsa-miR-22-3p | GRM5 | + | + | - | + |
| hsa-miR-22-3p | GRSF1 | - | - | - | + |
| hsa-miR-22-3p | GS1-211B7.1 | - | - | - | + |
| hsa-miR-22-3p | GSDMD | + | - | - | - |
| hsa-miR-22-3p | GSK3B | - | - | - | + |
| hsa-miR-22-3p | GSTM2 | - | + | - | - |
| hsa-miR-22-3p | GTF2A1 | - | - | - | + |
| hsa-miR-22-3p | GTF2B | - | - | - | + |
| hsa-miR-22-3p | GTF2E1 | - | - | - | + |
| hsa-miR-22-3p | GTF2IRD2 | + | - | - | - |
| hsa-miR-22-3p | GTF3C3 | + | - | - | + |
| hsa-miR-22-3p | GTPBP1 | - | - | - | + |
| hsa-miR-22-3p | GTPBP6 | + | - | - | + |
| hsa-miR-22-3p | GUCA1A | - | - | - | + |
| hsa-miR-22-3p | GUCD1 | + | + | - | + |
| hsa-miR-22-3p | GUCY1B3 | + | - | - | + |
| hsa-miR-22-3p | GUCY2C | - | - | - | + |
| hsa-miR-22-3p | GUK1 | + | - | - | + |
| hsa-miR-22-3p | GXYLT1 | - | - | - | + |
| hsa-miR-22-3p | GYS1 | - | - | - | + |
| hsa-miR-22-3p | GYS2 | - | - | - | + |
| hsa-miR-22-3p | H1FNT | - | - | - | + |
| hsa-miR-22-3p | H2AFV | - | - | - | + |
| hsa-miR-22-3p | H2AFX | + | - | - | - |
| hsa-miR-22-3p | H3F3B | + | + | + | + |
| hsa-miR-22-3p | H3F3C | + | + | - | + |
| hsa-miR-22-3p | H6PD | - | - | - | + |
| hsa-miR-22-3p | HABP4 | + | + | - | - |
| hsa-miR-22-3p | HAPLN1 | - | - | - | + |
| hsa-miR-22-3p | HAPLN4 | + | - | - | + |
| hsa-miR-22-3p | HAS3 | - | - | - | + |
| hsa-miR-22-3p | HAUS2 | - | - | - | + |
| hsa-miR-22-3p | HAUS5 | - | - | - | + |
| hsa-miR-22-3p | HAUS6 | - | - | - | + |
| hsa-miR-22-3p | HCFC1 | - | - | - | + |
| hsa-miR-22-3p | HCN4 | - | - | - | + |
| hsa-miR-22-3p | HDAC1 | - | - | - | + |
| hsa-miR-22-3p | HDAC4 | - | - | + | + |
| hsa-miR-22-3p | HDAC6 | - | - | + | + |
| hsa-miR-22-3p | HDAC8 | - | + | - | - |
| hsa-miR-22-3p | HDC | + | - | - | + |
| hsa-miR-22-3p | HDDC2 | + | - | - | - |
| hsa-miR-22-3p | HDX | - | - | - | + |
| hsa-miR-22-3p | HEBP1 | + | - | - | - |
| hsa-miR-22-3p | HECA | - | - | - | + |
| hsa-miR-22-3p | HECTD3 | - | - | - | + |
| hsa-miR-22-3p | HEG1 | - | - | - | + |
| hsa-miR-22-3p | HELZ | - | - | - | + |
| hsa-miR-22-3p | HEPACAM | - | - | - | + |
| hsa-miR-22-3p | HEPHL1 | - | - | - | + |
| hsa-miR-22-3p | HERC1 | - | - | - | + |
| hsa-miR-22-3p | HERC3 | - | - | - | + |
| hsa-miR-22-3p | HERPUD2 | + | + | - | - |
| hsa-miR-22-3p | HES2 | - | - | - | + |
| hsa-miR-22-3p | HEY1 | - | - | - | + |
| hsa-miR-22-3p | HEY2 | - | + | - | + |
| hsa-miR-22-3p | HFE2 | - | - | - | + |
| hsa-miR-22-3p | HGSNAT | + | + | - | + |
| hsa-miR-22-3p | HHIPL1 | - | - | - | + |
| hsa-miR-22-3p | HIF1A | - | - | + | - |
| hsa-miR-22-3p | HIF1AN | + | + | - | - |
| hsa-miR-22-3p | HIP1R | - | - | - | + |
| hsa-miR-22-3p | HIPK1 | - | + | - | + |
| hsa-miR-22-3p | HIPK2 | - | - | - | + |
| hsa-miR-22-3p | HIRIP3 | - | - | - | + |
| hsa-miR-22-3p | HIST1H1A | - | - | - | + |
| hsa-miR-22-3p | HIST1H1D | - | - | - | + |
| hsa-miR-22-3p | HIST1H2AG | + | - | - | - |
| hsa-miR-22-3p | HIST1H2AK | + | - | - | - |
| hsa-miR-22-3p | HIVEP3 | - | - | - | + |
| hsa-miR-22-3p | HLA-E | - | - | - | + |
| hsa-miR-22-3p | HLF | - | - | - | + |
| hsa-miR-22-3p | HLTF | - | - | - | + |
| hsa-miR-22-3p | HM13 | + | - | - | + |
| hsa-miR-22-3p | HMCN1 | + | - | - | - |
| hsa-miR-22-3p | HMG20A | - | - | - | + |
| hsa-miR-22-3p | HMGA2 | + | - | - | - |
| hsa-miR-22-3p | HMGB1 | - | - | + | - |
| hsa-miR-22-3p | HMGCR | - | - | - | + |
| hsa-miR-22-3p | HMMR | - | - | - | + |
| hsa-miR-22-3p | HMOX1 | + | - | - | + |
| hsa-miR-22-3p | HN1L | + | - | - | + |
| hsa-miR-22-3p | HNF1A | - | - | - | + |
| hsa-miR-22-3p | HNF4G | + | + | - | + |
| hsa-miR-22-3p | HNMT | - | - | - | + |
| hsa-miR-22-3p | HNRNPA2B1 | - | - | - | + |
| hsa-miR-22-3p | HNRNPA3 | + | - | + | + |
| hsa-miR-22-3p | HNRNPC | - | - | - | + |
| hsa-miR-22-3p | HNRNPF | - | - | - | + |
| hsa-miR-22-3p | HNRNPH2 | - | - | - | + |
| hsa-miR-22-3p | HNRNPUL1 | - | - | - | + |
| hsa-miR-22-3p | HNRNPUL2 | - | + | - | + |
| hsa-miR-22-3p | HNRPLL | - | - | - | + |
| hsa-miR-22-3p | HOMER1 | + | + | - | - |
| hsa-miR-22-3p | HOMER3 | + | + | - | + |
| hsa-miR-22-3p | HOOK1 | + | - | - | - |
| hsa-miR-22-3p | HORMAD2 | - | - | - | + |
| hsa-miR-22-3p | HOXA1 | - | - | - | + |
| hsa-miR-22-3p | HOXA3 | - | - | - | + |
| hsa-miR-22-3p | HOXA4 | + | + | - | - |
| hsa-miR-22-3p | HOXA5 | - | - | - | + |
| hsa-miR-22-3p | HOXC13 | - | - | - | + |
| hsa-miR-22-3p | HOXD1 | - | - | - | + |
| hsa-miR-22-3p | HOXD3 | - | - | - | + |
| hsa-miR-22-3p | HP1BP3 | - | - | - | + |
| hsa-miR-22-3p | HPCAL4 | - | - | - | + |
| hsa-miR-22-3p | HR | - | - | - | + |
| hsa-miR-22-3p | HRASLS2 | - | - | - | + |
| hsa-miR-22-3p | HRH1 | - | - | - | + |
| hsa-miR-22-3p | HRH2 | + | - | - | + |
| hsa-miR-22-3p | HS2ST1 | - | - | - | + |
| hsa-miR-22-3p | HS3ST3B1 | - | - | - | + |
| hsa-miR-22-3p | HS3ST4 | - | - | - | + |
| hsa-miR-22-3p | HS6ST1 | - | - | - | + |
| hsa-miR-22-3p | HS6ST3 | + | - | - | - |
| hsa-miR-22-3p | HSBP1 | + | - | - | - |
| hsa-miR-22-3p | HSD11B2 | - | - | - | + |
| hsa-miR-22-3p | HSD17B13 | - | - | - | + |
| hsa-miR-22-3p | HSF2BP | - | + | - | + |
| hsa-miR-22-3p | HSF5 | - | - | - | + |
| hsa-miR-22-3p | HSPA12A | - | - | - | + |
| hsa-miR-22-3p | HSPA12B | - | - | - | + |
| hsa-miR-22-3p | HSPA13 | - | - | - | + |
| hsa-miR-22-3p | HSPA1B | - | - | + | - |
| hsa-miR-22-3p | HTR2C | - | - | + | - |
| hsa-miR-22-3p | HTR3A | + | + | - | - |
| hsa-miR-22-3p | HTR7 | - | - | - | + |
| hsa-miR-22-3p | HTT | - | + | - | + |
| hsa-miR-22-3p | HUNK | - | + | - | + |
| hsa-miR-22-3p | HYDIN | + | - | - | - |
| hsa-miR-22-3p | IBA57 | - | - | - | + |
| hsa-miR-22-3p | ICAM4 | - | - | - | + |
| hsa-miR-22-3p | ICOSLG | + | - | - | - |
| hsa-miR-22-3p | ICT1 | + | - | - | - |
| hsa-miR-22-3p | IDE | - | - | - | + |
| hsa-miR-22-3p | IFFO2 | + | - | - | + |
| hsa-miR-22-3p | IFITM10 | + | - | - | - |
| hsa-miR-22-3p | IFT140 | + | - | + | - |
| hsa-miR-22-3p | IFT57 | - | - | - | + |
| hsa-miR-22-3p | IGF1 | - | - | - | + |
| hsa-miR-22-3p | IGF1R | - | - | - | + |
| hsa-miR-22-3p | IGF2BP1 | - | - | - | + |
| hsa-miR-22-3p | IGFBP4 | - | - | - | + |
| hsa-miR-22-3p | IGFL3 | - | - | - | + |
| hsa-miR-22-3p | IGLL1 | - | - | - | + |
| hsa-miR-22-3p | IGSF1 | - | - | - | + |
| hsa-miR-22-3p | IGSF3 | - | - | - | + |
| hsa-miR-22-3p | IHH | - | - | - | + |
| hsa-miR-22-3p | IKBKB | + | - | - | - |
| hsa-miR-22-3p | IKZF4 | + | + | - | - |
| hsa-miR-22-3p | IL13RA1 | + | + | - | + |
| hsa-miR-22-3p | IL16 | - | - | - | + |
| hsa-miR-22-3p | IL17D | - | - | - | + |
| hsa-miR-22-3p | IL17RA | - | - | - | + |
| hsa-miR-22-3p | IL17RD | + | + | - | + |
| hsa-miR-22-3p | IL17REL | - | - | - | + |
| hsa-miR-22-3p | IL1RL1 | + | - | - | + |
| hsa-miR-22-3p | IL27RA | + | + | - | + |
| hsa-miR-22-3p | IL2RB | + | - | - | + |
| hsa-miR-22-3p | IL31 | + | - | - | + |
| hsa-miR-22-3p | IL36G | + | - | - | - |
| hsa-miR-22-3p | IL36RN | - | - | - | + |
| hsa-miR-22-3p | IL6R | - | + | - | + |
| hsa-miR-22-3p | IL6ST | + | - | - | + |
| hsa-miR-22-3p | ILDR1 | - | - | - | + |
| hsa-miR-22-3p | IMPG2 | - | - | - | + |
| hsa-miR-22-3p | INA | - | - | - | + |
| hsa-miR-22-3p | INCENP | - | - | - | + |
| hsa-miR-22-3p | ING5 | - | - | - | + |
| hsa-miR-22-3p | INHBB | + | + | - | + |
| hsa-miR-22-3p | INO80 | + | - | - | - |
| hsa-miR-22-3p | INO80D | - | - | - | + |
| hsa-miR-22-3p | INO80E | + | - | - | - |
| hsa-miR-22-3p | INPP5B | + | + | - | - |
| hsa-miR-22-3p | INPP5D | - | - | - | + |
| hsa-miR-22-3p | INPP5E | - | - | - | + |
| hsa-miR-22-3p | INSIG1 | + | - | + | - |
| hsa-miR-22-3p | INSM2 | - | - | - | + |
| hsa-miR-22-3p | INSR | - | - | - | + |
| hsa-miR-22-3p | INTS12 | - | - | - | + |
| hsa-miR-22-3p | INTS2 | - | - | - | + |
| hsa-miR-22-3p | IP6K1 | - | - | - | + |
| hsa-miR-22-3p | IPO5 | - | - | - | + |
| hsa-miR-22-3p | IPO7 | + | + | - | + |
| hsa-miR-22-3p | IPO8 | - | - | - | + |
| hsa-miR-22-3p | IPO9 | - | - | - | + |
| hsa-miR-22-3p | IQCE | - | - | - | + |
| hsa-miR-22-3p | IQGAP3 | - | - | - | + |
| hsa-miR-22-3p | IRAK1 | - | - | - | + |
| hsa-miR-22-3p | IRAK2 | - | - | - | + |
| hsa-miR-22-3p | IRAK3 | - | - | - | + |
| hsa-miR-22-3p | IRF5 | + | - | + | - |
| hsa-miR-22-3p | IRF6 | - | - | - | + |
| hsa-miR-22-3p | IRGQ | - | - | - | + |
| hsa-miR-22-3p | IRS2 | - | - | - | + |
| hsa-miR-22-3p | ISY1 | - | - | - | + |
| hsa-miR-22-3p | ITCH | - | - | - | + |
| hsa-miR-22-3p | ITGA10 | + | + | - | + |
| hsa-miR-22-3p | ITGA5 | + | - | - | + |
| hsa-miR-22-3p | ITGAM | - | - | - | + |
| hsa-miR-22-3p | ITGAX | - | - | - | + |
| hsa-miR-22-3p | ITGB3 | + | - | - | - |
| hsa-miR-22-3p | ITGB3BP | + | - | - | - |
| hsa-miR-22-3p | ITGB5 | - | - | - | + |
| hsa-miR-22-3p | ITK | - | - | - | + |
| hsa-miR-22-3p | ITPA | - | - | - | + |
| hsa-miR-22-3p | ITPKA | - | - | - | + |
| hsa-miR-22-3p | ITPKB | - | - | - | + |
| hsa-miR-22-3p | IVL | - | - | - | + |
| hsa-miR-22-3p | JADE1 | - | - | - | + |
| hsa-miR-22-3p | JAGN1 | + | - | - | + |
| hsa-miR-22-3p | JARID2 | - | - | - | + |
| hsa-miR-22-3p | JMJD4 | + | - | - | + |
| hsa-miR-22-3p | JMY | - | - | - | + |
| hsa-miR-22-3p | JPH2 | + | - | - | + |
| hsa-miR-22-3p | KALRN | - | + | - | - |
| hsa-miR-22-3p | KANK2 | - | - | - | + |
| hsa-miR-22-3p | KAT5 | + | - | - | + |
| hsa-miR-22-3p | KAT6A | + | + | - | - |
| hsa-miR-22-3p | KAT6B | + | + | - | + |
| hsa-miR-22-3p | KAT7 | + | + | - | + |
| hsa-miR-22-3p | KATNA1 | - | - | - | + |
| hsa-miR-22-3p | KATNB1 | - | - | - | + |
| hsa-miR-22-3p | KATNBL1 | + | - | - | + |
| hsa-miR-22-3p | KBTBD11 | - | - | - | + |
| hsa-miR-22-3p | KBTBD12 | - | - | - | + |
| hsa-miR-22-3p | KBTBD5 | - | - | - | + |
| hsa-miR-22-3p | KCMF1 | - | - | - | + |
| hsa-miR-22-3p | KCNA1 | - | - | - | + |
| hsa-miR-22-3p | KCNA6 | - | - | - | + |
| hsa-miR-22-3p | KCNAB1 | + | + | - | + |
| hsa-miR-22-3p | KCNAB2 | - | - | - | + |
| hsa-miR-22-3p | KCNC1 | - | - | - | + |
| hsa-miR-22-3p | KCNC3 | - | - | - | + |
| hsa-miR-22-3p | KCNE1L | + | - | - | + |
| hsa-miR-22-3p | KCNIP3 | - | - | - | + |
| hsa-miR-22-3p | KCNIP4 | - | - | - | + |
| hsa-miR-22-3p | KCNJ12 | + | - | - | + |
| hsa-miR-22-3p | KCNJ13 | + | - | - | - |
| hsa-miR-22-3p | KCNJ16 | - | - | - | + |
| hsa-miR-22-3p | KCNK10 | + | + | - | + |
| hsa-miR-22-3p | KCNK12 | + | - | - | - |
| hsa-miR-22-3p | KCNK13 | - | - | - | + |
| hsa-miR-22-3p | KCNMB1 | + | - | - | - |
| hsa-miR-22-3p | KCNMB4 | - | - | - | + |
| hsa-miR-22-3p | KCNS3 | - | - | - | + |
| hsa-miR-22-3p | KCNU1 | - | - | - | + |
| hsa-miR-22-3p | KCTD10 | - | + | + | + |
| hsa-miR-22-3p | KCTD11 | - | - | - | + |
| hsa-miR-22-3p | KCTD12 | - | - | + | - |
| hsa-miR-22-3p | KCTD21 | - | - | - | + |
| hsa-miR-22-3p | KCTD5 | - | - | - | + |
| hsa-miR-22-3p | KDM3A | + | + | - | - |
| hsa-miR-22-3p | KDM5A | - | - | - | + |
| hsa-miR-22-3p | KDM5C | - | - | - | + |
| hsa-miR-22-3p | KDM6B | + | + | - | + |
| hsa-miR-22-3p | KDSR | - | - | - | + |
| hsa-miR-22-3p | KHDRBS2 | - | - | - | + |
| hsa-miR-22-3p | KHNYN | + | + | - | + |
| hsa-miR-22-3p | KIAA0040 | + | + | - | + |
| hsa-miR-22-3p | KIAA0182 | - | - | - | + |
| hsa-miR-22-3p | KIAA0196 | - | - | - | + |
| hsa-miR-22-3p | KIAA0226 | - | - | - | + |
| hsa-miR-22-3p | KIAA0226L | - | - | - | + |
| hsa-miR-22-3p | KIAA0232 | - | - | - | + |
| hsa-miR-22-3p | KIAA0247 | - | - | - | + |
| hsa-miR-22-3p | KIAA0319 | - | - | - | + |
| hsa-miR-22-3p | KIAA0319L | - | - | - | + |
| hsa-miR-22-3p | KIAA0355 | - | - | - | + |
| hsa-miR-22-3p | KIAA0430 | - | - | - | + |
| hsa-miR-22-3p | KIAA0556 | - | - | - | + |
| hsa-miR-22-3p | KIAA0754 | - | - | - | + |
| hsa-miR-22-3p | KIAA0895 | - | - | - | + |
| hsa-miR-22-3p | KIAA1147 | - | - | - | + |
| hsa-miR-22-3p | KIAA1211L | + | - | - | + |
| hsa-miR-22-3p | KIAA1244 | - | - | - | + |
| hsa-miR-22-3p | KIAA1456 | - | - | - | + |
| hsa-miR-22-3p | KIAA1467 | + | - | - | + |
| hsa-miR-22-3p | KIAA1522 | - | - | - | + |
| hsa-miR-22-3p | KIAA1549 | - | - | - | + |
| hsa-miR-22-3p | KIAA1598 | - | - | - | + |
| hsa-miR-22-3p | KIAA1715 | - | - | - | + |
| hsa-miR-22-3p | KIAA1755 | - | - | - | + |
| hsa-miR-22-3p | KIAA2018 | - | - | - | + |
| hsa-miR-22-3p | KIF11 | - | - | - | + |
| hsa-miR-22-3p | KIF12 | - | - | - | + |
| hsa-miR-22-3p | KIF13A | - | - | - | + |
| hsa-miR-22-3p | KIF13B | - | - | - | + |
| hsa-miR-22-3p | KIF14 | - | - | - | + |
| hsa-miR-22-3p | KIF18A | + | - | - | + |
| hsa-miR-22-3p | KIF18B | - | - | - | + |
| hsa-miR-22-3p | KIF1A | - | - | - | + |
| hsa-miR-22-3p | KIF1B | - | - | - | + |
| hsa-miR-22-3p | KIF1C | - | - | - | + |
| hsa-miR-22-3p | KIF24 | - | + | - | - |
| hsa-miR-22-3p | KIF26B | - | - | - | + |
| hsa-miR-22-3p | KIF2C | - | - | - | + |
| hsa-miR-22-3p | KIF3A | - | - | - | + |
| hsa-miR-22-3p | KIN | - | - | - | + |
| hsa-miR-22-3p | KIRREL3 | + | + | - | + |
| hsa-miR-22-3p | KLF11 | - | - | - | + |
| hsa-miR-22-3p | KLF12 | - | - | - | + |
| hsa-miR-22-3p | KLF13 | - | - | - | + |
| hsa-miR-22-3p | KLF16 | + | - | - | - |
| hsa-miR-22-3p | KLF3 | - | - | - | + |
| hsa-miR-22-3p | KLF5 | - | - | - | + |
| hsa-miR-22-3p | KLF6 | + | - | - | + |
| hsa-miR-22-3p | KLF7 | - | - | - | + |
| hsa-miR-22-3p | KLF8 | - | - | - | + |
| hsa-miR-22-3p | KLHDC5 | - | - | - | + |
| hsa-miR-22-3p | KLHDC7A | - | - | - | + |
| hsa-miR-22-3p | KLHDC8A | + | - | - | + |
| hsa-miR-22-3p | KLHDC8B | + | - | - | + |
| hsa-miR-22-3p | KLHDC9 | - | - | - | + |
| hsa-miR-22-3p | KLHL14 | - | - | - | + |
| hsa-miR-22-3p | KLHL15 | - | - | - | + |
| hsa-miR-22-3p | KLHL3 | - | - | - | + |
| hsa-miR-22-3p | KLHL4 | - | - | - | + |
| hsa-miR-22-3p | KLHL42 | + | - | - | - |
| hsa-miR-22-3p | KLRC2 | - | - | - | + |
| hsa-miR-22-3p | KMT2A | - | + | - | + |
| hsa-miR-22-3p | KMT2D | - | - | - | + |
| hsa-miR-22-3p | KNDC1 | + | - | - | - |
| hsa-miR-22-3p | KPNA6 | - | - | - | + |
| hsa-miR-22-3p | KPRP | + | + | - | - |
| hsa-miR-22-3p | KRAS | - | - | - | + |
| hsa-miR-22-3p | KREMEN1 | - | - | - | + |
| hsa-miR-22-3p | KRI1 | - | - | - | + |
| hsa-miR-22-3p | KRT10 | - | - | - | + |
| hsa-miR-22-3p | KRT6A | - | - | - | + |
| hsa-miR-22-3p | KRT6B | - | - | - | + |
| hsa-miR-22-3p | KRT6C | - | - | - | + |
| hsa-miR-22-3p | KRT75 | - | - | - | + |
| hsa-miR-22-3p | KRT76 | - | - | - | + |
| hsa-miR-22-3p | KRT77 | + | - | - | - |
| hsa-miR-22-3p | KRT78 | - | - | - | + |
| hsa-miR-22-3p | KRT80 | + | - | - | + |
| hsa-miR-22-3p | KRT85 | + | - | - | + |
| hsa-miR-22-3p | KRT9 | - | - | - | + |
| hsa-miR-22-3p | KRTAP1-5 | - | - | - | + |
| hsa-miR-22-3p | KRTAP12-2 | - | - | - | + |
| hsa-miR-22-3p | KRTAP17-1 | - | - | - | + |
| hsa-miR-22-3p | KRTAP2-3 | + | - | - | - |
| hsa-miR-22-3p | KRTAP9-9 | - | - | - | + |
| hsa-miR-22-3p | KSR1 | - | - | - | + |
| hsa-miR-22-3p | KSR2 | - | - | - | + |
| hsa-miR-22-3p | L3MBTL2 | + | - | - | - |
| hsa-miR-22-3p | LAIR1 | - | - | - | + |
| hsa-miR-22-3p | LAMA2 | - | - | - | + |
| hsa-miR-22-3p | LAMC1 | + | + | - | + |
| hsa-miR-22-3p | LAMP2 | - | - | - | + |
| hsa-miR-22-3p | LAMP3 | - | - | - | + |
| hsa-miR-22-3p | LANCL2 | - | - | - | + |
| hsa-miR-22-3p | LAPTM4A | - | - | - | + |
| hsa-miR-22-3p | LAPTM5 | - | - | - | + |
| hsa-miR-22-3p | LARGE | + | - | - | - |
| hsa-miR-22-3p | LARP1 | - | - | - | + |
| hsa-miR-22-3p | LARP4B | - | - | - | + |
| hsa-miR-22-3p | LARS | - | - | - | + |
| hsa-miR-22-3p | LAS1L | + | - | - | - |
| hsa-miR-22-3p | LASP1 | - | - | - | + |
| hsa-miR-22-3p | LAT | + | - | - | + |
| hsa-miR-22-3p | LATS1 | - | - | - | + |
| hsa-miR-22-3p | LAX1 | + | - | - | + |
| hsa-miR-22-3p | LBP | - | - | + | - |
| hsa-miR-22-3p | LCE1B | - | - | - | + |
| hsa-miR-22-3p | LCMT2 | + | - | - | - |
| hsa-miR-22-3p | LCN6 | + | - | - | - |
| hsa-miR-22-3p | LCNL1 | + | - | - | - |
| hsa-miR-22-3p | LCP1 | - | - | - | + |
| hsa-miR-22-3p | LDB1 | + | - | - | + |
| hsa-miR-22-3p | LDB3 | - | - | - | + |
| hsa-miR-22-3p | LDLRAD3 | - | - | - | + |
| hsa-miR-22-3p | LDLRAD4 | - | - | - | + |
| hsa-miR-22-3p | LDLRAP1 | - | - | - | + |
| hsa-miR-22-3p | LDOC1L | - | - | - | + |
| hsa-miR-22-3p | LEF1 | - | - | - | + |
| hsa-miR-22-3p | LEMD3 | - | - | + | + |
| hsa-miR-22-3p | LEP | - | - | - | + |
| hsa-miR-22-3p | LEPROT | + | - | - | + |
| hsa-miR-22-3p | LEPROTL1 | - | - | - | + |
| hsa-miR-22-3p | LGALS1 | + | - | + | - |
| hsa-miR-22-3p | LGALS9 | + | - | - | - |
| hsa-miR-22-3p | LGALS9B | + | - | - | - |
| hsa-miR-22-3p | LGALS9C | + | - | - | - |
| hsa-miR-22-3p | LGR5 | - | - | - | + |
| hsa-miR-22-3p | LHFPL4 | - | - | - | + |
| hsa-miR-22-3p | LHFPL5 | - | - | - | + |
| hsa-miR-22-3p | LHX5 | + | - | - | - |
| hsa-miR-22-3p | LHX6 | + | + | - | + |
| hsa-miR-22-3p | LIF | + | - | - | + |
| hsa-miR-22-3p | LIG3 | + | + | - | - |
| hsa-miR-22-3p | LILRA1 | - | - | - | + |
| hsa-miR-22-3p | LILRB4 | - | - | - | + |
| hsa-miR-22-3p | LIMCH1 | - | - | - | + |
| hsa-miR-22-3p | LIMD1 | + | - | - | - |
| hsa-miR-22-3p | LIMK1 | - | - | - | + |
| hsa-miR-22-3p | LIN28A | - | - | - | + |
| hsa-miR-22-3p | LIN28B | - | - | - | + |
| hsa-miR-22-3p | LIN7C | + | + | + | + |
| hsa-miR-22-3p | LINC01565 | - | - | - | + |
| hsa-miR-22-3p | LIX1L | + | - | - | - |
| hsa-miR-22-3p | LL22NC03-63E9.3 | + | - | - | + |
| hsa-miR-22-3p | LMAN2 | - | - | - | + |
| hsa-miR-22-3p | LMNB2 | - | - | - | + |
| hsa-miR-22-3p | LMO4 | - | - | - | + |
| hsa-miR-22-3p | LMX1B | - | - | - | + |
| hsa-miR-22-3p | LOC101930123 | - | + | - | - |
| hsa-miR-22-3p | LOC102723534 | - | + | - | - |
| hsa-miR-22-3p | LONP2 | - | - | + | - |
| hsa-miR-22-3p | LONRF2 | - | - | - | + |
| hsa-miR-22-3p | LOXL3 | - | - | - | + |
| hsa-miR-22-3p | LOXL4 | - | - | - | + |
| hsa-miR-22-3p | LPAR5 | - | - | - | + |
| hsa-miR-22-3p | LPCAT2 | - | - | - | + |
| hsa-miR-22-3p | LPHN2 | - | - | - | + |
| hsa-miR-22-3p | LPIN2 | + | - | - | + |
| hsa-miR-22-3p | LPIN3 | + | - | - | + |
| hsa-miR-22-3p | LPL | - | - | - | + |
| hsa-miR-22-3p | LPP | + | - | - | - |
| hsa-miR-22-3p | LRCH1 | + | + | - | - |
| hsa-miR-22-3p | LRFN2 | - | - | - | + |
| hsa-miR-22-3p | LRIT1 | - | - | - | + |
| hsa-miR-22-3p | LRIT2 | - | - | - | + |
| hsa-miR-22-3p | LRP11 | - | - | - | + |
| hsa-miR-22-3p | LRP12 | - | - | - | + |
| hsa-miR-22-3p | LRP1B | - | - | - | + |
| hsa-miR-22-3p | LRRC1 | - | + | + | + |
| hsa-miR-22-3p | LRRC14 | - | - | - | + |
| hsa-miR-22-3p | LRRC16A | + | + | - | + |
| hsa-miR-22-3p | LRRC20 | - | - | + | + |
| hsa-miR-22-3p | LRRC3 | + | - | - | - |
| hsa-miR-22-3p | LRRC38 | + | - | - | + |
| hsa-miR-22-3p | LRRC3DN | + | - | - | - |
| hsa-miR-22-3p | LRRC41 | - | - | - | + |
| hsa-miR-22-3p | LRRC55 | + | - | - | + |
| hsa-miR-22-3p | LRRC59 | + | + | - | + |
| hsa-miR-22-3p | LRRC73 | + | + | - | + |
| hsa-miR-22-3p | LRRC8E | + | - | - | + |
| hsa-miR-22-3p | LRRN2 | - | - | - | + |
| hsa-miR-22-3p | LRTM1 | - | - | - | + |
| hsa-miR-22-3p | LSM11 | - | - | - | + |
| hsa-miR-22-3p | LTB4R | + | - | - | + |
| hsa-miR-22-3p | LTBP1 | - | - | - | + |
| hsa-miR-22-3p | LTBP2 | - | - | - | + |
| hsa-miR-22-3p | LTK | - | - | - | + |
| hsa-miR-22-3p | LUZP1 | - | - | - | + |
| hsa-miR-22-3p | LUZP4 | + | - | - | - |
| hsa-miR-22-3p | LY6H | + | - | - | - |
| hsa-miR-22-3p | LY75-CD302 | - | + | - | - |
| hsa-miR-22-3p | LYPD3 | - | - | - | + |
| hsa-miR-22-3p | LYPLA2 | + | - | - | - |
| hsa-miR-22-3p | LZIC | + | - | - | + |
| hsa-miR-22-3p | LZTFL1 | - | - | - | + |
| hsa-miR-22-3p | MAB21L3 | - | - | - | + |
| hsa-miR-22-3p | MACC1 | - | - | - | + |
| hsa-miR-22-3p | MACROD1 | + | - | - | - |
| hsa-miR-22-3p | MACROD2 | + | - | - | - |
| hsa-miR-22-3p | MADD | - | - | - | + |
| hsa-miR-22-3p | MAFG | - | - | - | + |
| hsa-miR-22-3p | MAFK | + | - | - | + |
| hsa-miR-22-3p | MAGI2 | + | + | - | + |
| hsa-miR-22-3p | MAGI3 | - | - | - | + |
| hsa-miR-22-3p | MAK16 | - | - | - | + |
| hsa-miR-22-3p | MALL | + | - | - | + |
| hsa-miR-22-3p | MAML2 | - | - | - | + |
| hsa-miR-22-3p | MAN1A2 | - | - | - | + |
| hsa-miR-22-3p | MAN2A2 | - | - | - | + |
| hsa-miR-22-3p | MAOA | - | - | + | - |
| hsa-miR-22-3p | MAP1B | - | - | - | + |
| hsa-miR-22-3p | MAP2K4 | + | - | - | - |
| hsa-miR-22-3p | MAP3K1 | - | - | - | + |
| hsa-miR-22-3p | MAP3K12 | + | + | - | - |
| hsa-miR-22-3p | MAP3K13 | + | - | - | + |
| hsa-miR-22-3p | MAP3K3 | - | + | - | + |
| hsa-miR-22-3p | MAP3K5 | - | - | - | + |
| hsa-miR-22-3p | MAP3K6 | + | - | - | + |
| hsa-miR-22-3p | MAP3K7 | - | - | - | + |
| hsa-miR-22-3p | MAP3K9 | - | - | - | + |
| hsa-miR-22-3p | MAP4K5 | - | - | - | + |
| hsa-miR-22-3p | MAP6 | - | - | - | + |
| hsa-miR-22-3p | MAP7 | - | - | - | + |
| hsa-miR-22-3p | MAP9 | - | - | - | + |
| hsa-miR-22-3p | MAPK1 | + | - | - | - |
| hsa-miR-22-3p | MAPK10 | + | - | - | - |
| hsa-miR-22-3p | MAPK14 | + | + | - | + |
| hsa-miR-22-3p | MAPK1IP1L | + | - | - | + |
| hsa-miR-22-3p | MAPK4 | - | - | - | + |
| hsa-miR-22-3p | MAPK8IP3 | - | + | - | + |
| hsa-miR-22-3p | MAPK9 | - | - | - | + |
| hsa-miR-22-3p | MAPKAPK2 | - | - | - | + |
| hsa-miR-22-3p | MAPRE3 | - | - | - | + |
| hsa-miR-22-3p | MAPT | + | - | - | + |
| hsa-miR-22-3p | 2-Mar | - | - | - | + |
| hsa-miR-22-3p | 8-Mar | - | - | - | + |
| hsa-miR-22-3p | 9-Mar | + | - | - | + |
| hsa-miR-22-3p | MARK1 | - | - | - | + |
| hsa-miR-22-3p | MARK4 | - | - | - | + |
| hsa-miR-22-3p | MAST3 | - | - | - | + |
| hsa-miR-22-3p | MAT2A | + | + | - | + |
| hsa-miR-22-3p | MAVS | - | - | - | + |
| hsa-miR-22-3p | MAX | + | + | + | - |
| hsa-miR-22-3p | MAZ | - | - | - | + |
| hsa-miR-22-3p | MBD1 | - | - | - | + |
| hsa-miR-22-3p | MBD2 | - | - | - | + |
| hsa-miR-22-3p | MBD3 | + | - | - | - |
| hsa-miR-22-3p | MBL2 | - | - | - | + |
| hsa-miR-22-3p | MBNL3 | - | - | - | + |
| hsa-miR-22-3p | MC2R | - | - | - | + |
| hsa-miR-22-3p | MCAM | - | - | - | + |
| hsa-miR-22-3p | MCC | - | - | - | + |
| hsa-miR-22-3p | MCF2L | - | - | - | + |
| hsa-miR-22-3p | MCL1 | - | - | - | + |
| hsa-miR-22-3p | MCM6 | - | - | - | + |
| hsa-miR-22-3p | MCMDC2 | - | - | - | + |
| hsa-miR-22-3p | MDC1 | - | + | - | + |
| hsa-miR-22-3p | MDGA1 | + | - | - | + |
| hsa-miR-22-3p | MDH2 | - | - | - | + |
| hsa-miR-22-3p | MDM2 | - | - | - | + |
| hsa-miR-22-3p | MDN1 | - | - | - | + |
| hsa-miR-22-3p | MECOM | + | - | - | + |
| hsa-miR-22-3p | MECP2 | + | - | - | + |
| hsa-miR-22-3p | MED19 | - | - | - | + |
| hsa-miR-22-3p | MED8 | - | - | - | + |
| hsa-miR-22-3p | MED9 | - | - | - | + |
| hsa-miR-22-3p | MEGF11 | + | - | - | - |
| hsa-miR-22-3p | MEGF8 | - | - | - | + |
| hsa-miR-22-3p | MEGF9 | - | - | - | + |
| hsa-miR-22-3p | MEIS2 | + | + | - | - |
| hsa-miR-22-3p | MEOX1 | - | - | - | + |
| hsa-miR-22-3p | MESDC2 | - | - | - | + |
| hsa-miR-22-3p | MESP2 | + | - | - | - |
| hsa-miR-22-3p | METAP2 | - | - | - | + |
| hsa-miR-22-3p | METTL7A | - | - | - | + |
| hsa-miR-22-3p | METTL9 | - | - | - | + |
| hsa-miR-22-3p | MFAP5 | - | - | - | + |
| hsa-miR-22-3p | MFGE8 | + | + | - | - |
| hsa-miR-22-3p | MFN1 | + | + | - | - |
| hsa-miR-22-3p | MFN2 | - | - | - | + |
| hsa-miR-22-3p | MFSD10 | + | - | - | - |
| hsa-miR-22-3p | MFSD2A | - | - | - | + |
| hsa-miR-22-3p | MFSD5 | - | - | - | + |
| hsa-miR-22-3p | MFSD6 | - | - | - | + |
| hsa-miR-22-3p | MFSD6L | - | - | - | + |
| hsa-miR-22-3p | MFSD8 | - | - | - | + |
| hsa-miR-22-3p | MGAT3 | + | + | - | + |
| hsa-miR-22-3p | MGAT5 | - | - | - | + |
| hsa-miR-22-3p | MGRN1 | - | - | - | + |
| hsa-miR-22-3p | MICAL1 | - | - | - | + |
| hsa-miR-22-3p | MICALCL | + | + | - | + |
| hsa-miR-22-3p | MICALL1 | - | - | - | + |
| hsa-miR-22-3p | MICB | - | - | - | + |
| hsa-miR-22-3p | MID1 | - | - | - | + |
| hsa-miR-22-3p | MIEF2 | + | + | - | + |
| hsa-miR-22-3p | MINOS1 | - | - | - | + |
| hsa-miR-22-3p | MINPP1 | - | - | - | + |
| hsa-miR-22-3p | MIP | - | - | - | + |
| hsa-miR-22-3p | MIS18BP1 | - | - | + | + |
| hsa-miR-22-3p | MKI67 | - | - | - | + |
| hsa-miR-22-3p | MKLN1 | - | - | - | + |
| hsa-miR-22-3p | MKX | - | - | - | + |
| hsa-miR-22-3p | MLANA | + | - | - | - |
| hsa-miR-22-3p | MLC1 | - | - | - | + |
| hsa-miR-22-3p | MLEC | + | - | - | + |
| hsa-miR-22-3p | MLF1 | - | - | - | + |
| hsa-miR-22-3p | MLKL | + | + | - | + |
| hsa-miR-22-3p | MLL4 | - | - | - | + |
| hsa-miR-22-3p | MLLT10 | - | - | - | + |
| hsa-miR-22-3p | MLLT3 | - | - | - | + |
| hsa-miR-22-3p | MMAB | - | - | - | + |
| hsa-miR-22-3p | MMD | - | - | - | + |
| hsa-miR-22-3p | MMGT1 | - | - | - | + |
| hsa-miR-22-3p | MMP1 | - | - | - | + |
| hsa-miR-22-3p | MMP14 | + | - | - | - |
| hsa-miR-22-3p | MMP16 | - | - | - | + |
| hsa-miR-22-3p | MMP19 | + | - | - | - |
| hsa-miR-22-3p | MMP24 | - | - | - | + |
| hsa-miR-22-3p | MMRN2 | - | - | - | + |
| hsa-miR-22-3p | MMS22L | - | - | - | + |
| hsa-miR-22-3p | MN1 | - | - | - | + |
| hsa-miR-22-3p | MOAP1 | - | - | - | + |
| hsa-miR-22-3p | MOB1A | + | - | - | - |
| hsa-miR-22-3p | MOB1B | + | + | - | + |
| hsa-miR-22-3p | MOB3A | - | - | - | + |
| hsa-miR-22-3p | MOB3C | - | - | - | + |
| hsa-miR-22-3p | MOCS1 | + | - | - | + |
| hsa-miR-22-3p | MON2 | + | + | - | - |
| hsa-miR-22-3p | MORC3 | - | - | - | + |
| hsa-miR-22-3p | MORF4L1 | - | - | - | + |
| hsa-miR-22-3p | MPP1 | + | - | - | - |
| hsa-miR-22-3p | MPZL3 | + | + | - | + |
| hsa-miR-22-3p | MRAS | - | - | - | + |
| hsa-miR-22-3p | MRC1 | + | - | - | - |
| hsa-miR-22-3p | MRC1L1 | + | - | - | - |
| hsa-miR-22-3p | MRFAP1L1 | - | - | - | + |
| hsa-miR-22-3p | MRGPRG-AS1 | - | - | - | + |
| hsa-miR-22-3p | MRPL23 | + | - | - | - |
| hsa-miR-22-3p | MRPL35 | - | - | - | + |
| hsa-miR-22-3p | MRPL49 | - | - | - | + |
| hsa-miR-22-3p | MRPL52 | - | - | - | + |
| hsa-miR-22-3p | MRPS10 | - | - | - | + |
| hsa-miR-22-3p | MRPS25 | - | - | - | + |
| hsa-miR-22-3p | MRPS36 | - | - | - | + |
| hsa-miR-22-3p | MRTO4 | - | - | - | + |
| hsa-miR-22-3p | MSI2 | - | - | - | + |
| hsa-miR-22-3p | MSL2 | + | + | - | + |
| hsa-miR-22-3p | MSL3 | - | - | - | + |
| hsa-miR-22-3p | MSR1 | - | - | - | + |
| hsa-miR-22-3p | MSRB3 | - | - | - | + |
| hsa-miR-22-3p | MST1L | - | + | - | - |
| hsa-miR-22-3p | MST4 | + | - | - | - |
| hsa-miR-22-3p | MSX2 | - | - | - | + |
| hsa-miR-22-3p | MTA3 | - | - | - | + |
| hsa-miR-22-3p | MTAP | - | - | - | + |
| hsa-miR-22-3p | MTCP1 | - | - | - | + |
| hsa-miR-22-3p | MTDH | - | - | - | + |
| hsa-miR-22-3p | MTF1 | + | + | - | + |
| hsa-miR-22-3p | MTF2 | - | + | - | + |
| hsa-miR-22-3p | MTG1 | - | - | - | + |
| hsa-miR-22-3p | MTHFD2 | + | + | - | - |
| hsa-miR-22-3p | MTHFR | + | + | - | - |
| hsa-miR-22-3p | MTL5 | - | - | - | + |
| hsa-miR-22-3p | MTMR1 | + | - | - | + |
| hsa-miR-22-3p | MTMR12 | - | - | - | + |
| hsa-miR-22-3p | MTMR2 | + | + | - | + |
| hsa-miR-22-3p | MTMR3 | - | - | - | + |
| hsa-miR-22-3p | MTMR4 | - | - | - | + |
| hsa-miR-22-3p | MTMR6 | - | - | - | + |
| hsa-miR-22-3p | MTMR7 | - | - | - | + |
| hsa-miR-22-3p | MTPN | - | - | - | + |
| hsa-miR-22-3p | MTRF1L | - | - | - | + |
| hsa-miR-22-3p | MTX3 | + | - | - | - |
| hsa-miR-22-3p | MUL1 | - | - | - | + |
| hsa-miR-22-3p | MUM1L1 | + | - | - | + |
| hsa-miR-22-3p | MURC | + | + | - | + |
| hsa-miR-22-3p | MVB12B | + | + | - | + |
| hsa-miR-22-3p | MXD1 | - | - | - | + |
| hsa-miR-22-3p | MXD3 | + | - | - | - |
| hsa-miR-22-3p | MXD4 | - | - | - | + |
| hsa-miR-22-3p | MYADML2 | + | - | - | + |
| hsa-miR-22-3p | MYCBP | + | - | + | - |
| hsa-miR-22-3p | MYCL | - | - | - | + |
| hsa-miR-22-3p | MYH10 | - | - | - | + |
| hsa-miR-22-3p | MYH15 | - | - | - | + |
| hsa-miR-22-3p | MYL12A | - | - | - | + |
| hsa-miR-22-3p | MYLIP | - | - | - | + |
| hsa-miR-22-3p | MYO1B | - | - | - | + |
| hsa-miR-22-3p | MYO5A | - | - | - | + |
| hsa-miR-22-3p | MYO6 | - | - | + | + |
| hsa-miR-22-3p | MYO9A | - | - | - | + |
| hsa-miR-22-3p | MYOC | + | - | - | + |
| hsa-miR-22-3p | MYOM3 | + | - | - | - |
| hsa-miR-22-3p | MYOT | - | - | - | + |
| hsa-miR-22-3p | MYOZ2 | + | - | - | + |
| hsa-miR-22-3p | MYOZ3 | + | - | - | + |
| hsa-miR-22-3p | MYPN | - | - | - | + |
| hsa-miR-22-3p | MYSM1 | - | - | - | + |
| hsa-miR-22-3p | MYT1 | - | - | - | + |
| hsa-miR-22-3p | MYT1L | - | - | - | + |
| hsa-miR-22-3p | MZT1 | + | - | - | + |
| hsa-miR-22-3p | N4BP1 | - | - | - | + |
| hsa-miR-22-3p | N4BP2L1 | + | - | - | + |
| hsa-miR-22-3p | N4BP3 | - | + | - | + |
| hsa-miR-22-3p | NAA10 | + | - | - | - |
| hsa-miR-22-3p | NAA15 | - | - | - | + |
| hsa-miR-22-3p | NAA16 | - | - | - | + |
| hsa-miR-22-3p | NAA20 | + | + | - | + |
| hsa-miR-22-3p | NAA25 | - | - | - | + |
| hsa-miR-22-3p | NAA40 | - | - | - | + |
| hsa-miR-22-3p | NAALADL2 | + | - | - | + |
| hsa-miR-22-3p | NACA2 | - | - | - | + |
| hsa-miR-22-3p | NAGS | - | - | - | + |
| hsa-miR-22-3p | NALCN | - | - | - | + |
| hsa-miR-22-3p | NAP1L1 | - | - | - | + |
| hsa-miR-22-3p | NAP1L4 | - | - | - | + |
| hsa-miR-22-3p | NAP1L5 | - | - | - | + |
| hsa-miR-22-3p | NAPG | - | - | - | + |
| hsa-miR-22-3p | NAT6 | + | - | - | + |
| hsa-miR-22-3p | NAT9 | + | - | - | + |
| hsa-miR-22-3p | NBEA | - | - | - | + |
| hsa-miR-22-3p | NBR1 | + | - | - | + |
| hsa-miR-22-3p | NCALD | - | - | - | + |
| hsa-miR-22-3p | NCAM1 | - | - | - | + |
| hsa-miR-22-3p | NCAPD3 | + | - | - | - |
| hsa-miR-22-3p | NCBP1 | - | - | - | + |
| hsa-miR-22-3p | NCDN | - | - | - | + |
| hsa-miR-22-3p | NCLN | - | + | - | - |
| hsa-miR-22-3p | NCMAP | + | + | - | + |
| hsa-miR-22-3p | NCOA1 | + | - | + | + |
| hsa-miR-22-3p | NCR3 | + | - | - | + |
| hsa-miR-22-3p | NDEL1 | + | + | - | + |
| hsa-miR-22-3p | NDRG3 | - | - | - | + |
| hsa-miR-22-3p | NDUFA10 | - | - | - | + |
| hsa-miR-22-3p | NDUFA5 | - | - | - | + |
| hsa-miR-22-3p | NDUFB10 | + | - | - | - |
| hsa-miR-22-3p | NEB | - | - | - | + |
| hsa-miR-22-3p | NEBL | - | - | - | + |
| hsa-miR-22-3p | NECAB1 | - | - | - | + |
| hsa-miR-22-3p | NECAP1 | + | + | - | - |
| hsa-miR-22-3p | NECAP2 | + | - | - | + |
| hsa-miR-22-3p | NEFM | + | - | - | + |
| hsa-miR-22-3p | NEGR1 | + | + | - | - |
| hsa-miR-22-3p | NEK1 | - | - | - | + |
| hsa-miR-22-3p | NELL1 | - | - | - | + |
| hsa-miR-22-3p | NELL2 | + | - | - | + |
| hsa-miR-22-3p | NEMP2 | - | + | - | - |
| hsa-miR-22-3p | NEO1 | + | - | - | + |
| hsa-miR-22-3p | NET1 | + | + | + | + |
| hsa-miR-22-3p | NEU3 | + | - | - | - |
| hsa-miR-22-3p | NEURL | - | - | - | + |
| hsa-miR-22-3p | NEUROD1 | - | - | - | + |
| hsa-miR-22-3p | NFAM1 | - | - | - | + |
| hsa-miR-22-3p | NFATC2 | - | - | - | + |
| hsa-miR-22-3p | NFATC2IP | - | - | - | + |
| hsa-miR-22-3p | NFATC3 | - | - | - | + |
| hsa-miR-22-3p | NFIB | - | - | - | + |
| hsa-miR-22-3p | NFIC | - | - | - | + |
| hsa-miR-22-3p | NFKB1 | - | - | - | + |
| hsa-miR-22-3p | NFKBIZ | - | - | - | + |
| hsa-miR-22-3p | NFYA | + | + | - | - |
| hsa-miR-22-3p | NGB | + | - | - | + |
| hsa-miR-22-3p | NGFR | - | - | - | + |
| hsa-miR-22-3p | NHLRC4 | - | - | - | + |
| hsa-miR-22-3p | NHP2 | + | + | - | + |
| hsa-miR-22-3p | NHS | - | - | - | + |
| hsa-miR-22-3p | NINJ1 | + | - | - | + |
| hsa-miR-22-3p | NIP7 | - | - | - | + |
| hsa-miR-22-3p | NIPA1 | - | - | - | + |
| hsa-miR-22-3p | NIPSNAP3A | - | - | - | + |
| hsa-miR-22-3p | NIT1 | - | - | - | + |
| hsa-miR-22-3p | NKAIN1 | - | - | - | + |
| hsa-miR-22-3p | NKAPL | - | - | - | + |
| hsa-miR-22-3p | NKD1 | - | - | - | + |
| hsa-miR-22-3p | NKX2-2 | - | - | - | + |
| hsa-miR-22-3p | NKX2-3 | - | - | - | + |
| hsa-miR-22-3p | NKX6-3 | + | - | - | - |
| hsa-miR-22-3p | NLGN4X | - | - | - | + |
| hsa-miR-22-3p | NLN | - | - | - | + |
| hsa-miR-22-3p | NLRP14 | + | - | - | - |
| hsa-miR-22-3p | NLRP3 | + | + | - | + |
| hsa-miR-22-3p | NLRP8 | - | - | - | + |
| hsa-miR-22-3p | NOL4L | - | - | - | + |
| hsa-miR-22-3p | NOL9 | - | - | - | + |
| hsa-miR-22-3p | NOLC1 | - | - | - | + |
| hsa-miR-22-3p | NONO | - | - | - | + |
| hsa-miR-22-3p | NOP9 | - | - | - | + |
| hsa-miR-22-3p | NOS1 | - | - | - | + |
| hsa-miR-22-3p | NOTCH2 | - | - | - | + |
| hsa-miR-22-3p | NOTCH4 | - | - | - | + |
| hsa-miR-22-3p | NOVA1 | + | - | - | - |
| hsa-miR-22-3p | NOXRED1 | - | - | - | + |
| hsa-miR-22-3p | NPAS2 | - | - | - | + |
| hsa-miR-22-3p | NPAS3 | + | + | - | + |
| hsa-miR-22-3p | NPAT | - | - | - | + |
| hsa-miR-22-3p | NPFFR1 | + | - | - | - |
| hsa-miR-22-3p | NPHP3 | + | - | - | - |
| hsa-miR-22-3p | NPHS2 | - | - | - | + |
| hsa-miR-22-3p | NPNT | + | - | - | + |
| hsa-miR-22-3p | NPPA | - | - | - | + |
| hsa-miR-22-3p | NPTX2 | - | - | - | + |
| hsa-miR-22-3p | NR1H2 | - | - | - | + |
| hsa-miR-22-3p | NR3C1 | - | + | + | + |
| hsa-miR-22-3p | NR4A2 | - | - | - | + |
| hsa-miR-22-3p | NR5A1 | - | - | - | + |
| hsa-miR-22-3p | NRAS | - | + | - | + |
| hsa-miR-22-3p | NRBP2 | - | - | - | + |
| hsa-miR-22-3p | NRF1 | - | - | - | + |
| hsa-miR-22-3p | NRGN | + | - | - | - |
| hsa-miR-22-3p | NRK | - | - | - | + |
| hsa-miR-22-3p | NRL | - | - | - | + |
| hsa-miR-22-3p | NRM | - | - | - | + |
| hsa-miR-22-3p | NRP1 | - | - | - | + |
| hsa-miR-22-3p | NRSN1 | + | - | - | - |
| hsa-miR-22-3p | NSD1 | - | - | - | + |
| hsa-miR-22-3p | NSDHL | + | - | - | + |
| hsa-miR-22-3p | NT5C2 | - | - | - | + |
| hsa-miR-22-3p | NT5E | - | - | - | + |
| hsa-miR-22-3p | NTMT1 | + | - | - | + |
| hsa-miR-22-3p | NTN4 | - | - | - | + |
| hsa-miR-22-3p | NTRK2 | + | + | - | + |
| hsa-miR-22-3p | NTS | - | - | - | + |
| hsa-miR-22-3p | NTSR1 | + | - | - | + |
| hsa-miR-22-3p | NUCKS1 | - | - | - | + |
| hsa-miR-22-3p | NUDT10 | + | - | - | + |
| hsa-miR-22-3p | NUDT15 | - | - | - | + |
| hsa-miR-22-3p | NUDT16 | - | - | - | + |
| hsa-miR-22-3p | NUDT2 | - | - | - | + |
| hsa-miR-22-3p | NUDT4 | + | + | - | + |
| hsa-miR-22-3p | NUFIP2 | - | - | - | + |
| hsa-miR-22-3p | NUGGC | - | - | - | + |
| hsa-miR-22-3p | NUP210 | - | + | - | + |
| hsa-miR-22-3p | NUP214 | - | - | + | + |
| hsa-miR-22-3p | NUP50 | - | - | - | + |
| hsa-miR-22-3p | NUP98 | - | - | - | + |
| hsa-miR-22-3p | NUS1 | + | + | - | + |
| hsa-miR-22-3p | NUSAP1 | + | + | - | + |
| hsa-miR-22-3p | NXF1 | + | - | - | + |
| hsa-miR-22-3p | NXPH1 | - | - | - | + |
| hsa-miR-22-3p | NXT2 | + | - | - | + |
| hsa-miR-22-3p | NYAP2 | + | - | - | + |
| hsa-miR-22-3p | NYNRIN | - | - | - | + |
| hsa-miR-22-3p | O3FAR1 | - | - | - | + |
| hsa-miR-22-3p | OAS3 | - | - | - | + |
| hsa-miR-22-3p | OASL | - | - | - | + |
| hsa-miR-22-3p | OCIAD1 | - | - | - | + |
| hsa-miR-22-3p | OCLN | + | - | - | + |
| hsa-miR-22-3p | ODF1 | + | + | - | - |
| hsa-miR-22-3p | ODF2 | - | - | - | + |
| hsa-miR-22-3p | ODZ3 | - | - | - | + |
| hsa-miR-22-3p | ODZ4 | - | - | - | + |
| hsa-miR-22-3p | OGDH | + | - | - | + |
| hsa-miR-22-3p | OGN | + | + | - | + |
| hsa-miR-22-3p | OGT | - | - | - | + |
| hsa-miR-22-3p | OLA1 | - | - | - | + |
| hsa-miR-22-3p | OLFM3 | - | + | - | - |
| hsa-miR-22-3p | OLFM4 | + | - | - | + |
| hsa-miR-22-3p | OLFML1 | - | - | - | + |
| hsa-miR-22-3p | OLFML2A | - | + | - | + |
| hsa-miR-22-3p | OLFML3 | - | - | - | + |
| hsa-miR-22-3p | ONECUT2 | - | - | - | + |
| hsa-miR-22-3p | OPALIN | - | - | - | + |
| hsa-miR-22-3p | OPCML | - | - | - | + |
| hsa-miR-22-3p | OPHN1 | + | - | - | + |
| hsa-miR-22-3p | OPN5 | - | + | - | - |
| hsa-miR-22-3p | OPRK1 | - | - | - | + |
| hsa-miR-22-3p | OPRL1 | + | - | - | + |
| hsa-miR-22-3p | OR10S1 | - | - | - | + |
| hsa-miR-22-3p | OR12D3 | - | - | - | + |
| hsa-miR-22-3p | OR3A2 | - | - | - | + |
| hsa-miR-22-3p | OR4K15 | - | - | - | + |
| hsa-miR-22-3p | OR6C1 | - | - | - | + |
| hsa-miR-22-3p | OR6C4 | - | - | - | + |
| hsa-miR-22-3p | OR6C74 | - | - | - | + |
| hsa-miR-22-3p | OR8D1 | - | - | - | + |
| hsa-miR-22-3p | OR9A2 | - | - | - | + |
| hsa-miR-22-3p | OR9Q1 | - | - | - | + |
| hsa-miR-22-3p | ORAI2 | - | - | - | + |
| hsa-miR-22-3p | ORAI3 | - | - | - | + |
| hsa-miR-22-3p | ORC4 | - | - | - | + |
| hsa-miR-22-3p | ORMDL1 | - | - | - | + |
| hsa-miR-22-3p | ORMDL2 | - | + | - | - |
| hsa-miR-22-3p | ORMDL3 | + | + | - | + |
| hsa-miR-22-3p | OSGIN2 | - | - | - | + |
| hsa-miR-22-3p | OSM | + | - | - | + |
| hsa-miR-22-3p | OSMR | - | - | - | + |
| hsa-miR-22-3p | OSR1 | + | - | - | + |
| hsa-miR-22-3p | OSTC | - | - | - | + |
| hsa-miR-22-3p | OTOA | - | - | - | + |
| hsa-miR-22-3p | OTUB2 | - | - | - | + |
| hsa-miR-22-3p | OTUD3 | - | - | - | + |
| hsa-miR-22-3p | OTUD4 | - | - | - | + |
| hsa-miR-22-3p | OTUD6B | - | + | - | + |
| hsa-miR-22-3p | OXLD1 | + | - | - | - |
| hsa-miR-22-3p | OXNAD1 | - | - | - | + |
| hsa-miR-22-3p | OXR1 | - | - | - | + |
| hsa-miR-22-3p | OXSR1 | - | - | - | + |
| hsa-miR-22-3p | P2RY1 | + | - | - | - |
| hsa-miR-22-3p | P2RY11 | + | - | - | + |
| hsa-miR-22-3p | P2RY8 | - | - | - | + |
| hsa-miR-22-3p | PABPC1L2A | - | - | - | + |
| hsa-miR-22-3p | PABPC4L | + | - | - | - |
| hsa-miR-22-3p | PACRGL | - | - | - | + |
| hsa-miR-22-3p | PADI2 | - | - | - | + |
| hsa-miR-22-3p | PADI3 | - | - | - | + |
| hsa-miR-22-3p | PAEP | + | - | - | - |
| hsa-miR-22-3p | PAFAH1B1 | - | - | - | + |
| hsa-miR-22-3p | PAFAH1B2 | - | - | - | + |
| hsa-miR-22-3p | PAIP2B | - | - | - | + |
| hsa-miR-22-3p | PAK1 | - | - | - | + |
| hsa-miR-22-3p | PAK2 | - | - | - | + |
| hsa-miR-22-3p | PALD1 | + | + | - | + |
| hsa-miR-22-3p | PALLD | - | - | - | + |
| hsa-miR-22-3p | PALM | + | - | - | + |
| hsa-miR-22-3p | PAPD7 | - | - | - | + |
| hsa-miR-22-3p | PAPOLA | - | - | - | + |
| hsa-miR-22-3p | PAPPA | - | - | - | + |
| hsa-miR-22-3p | PAPPA2 | - | - | - | + |
| hsa-miR-22-3p | PAQR7 | - | - | - | + |
| hsa-miR-22-3p | PARD3 | - | - | - | + |
| hsa-miR-22-3p | PARK2 | - | - | - | + |
| hsa-miR-22-3p | PARM1 | + | - | - | - |
| hsa-miR-22-3p | PARP15 | - | - | - | + |
| hsa-miR-22-3p | PARP3 | + | - | - | - |
| hsa-miR-22-3p | PASK | - | - | - | + |
| hsa-miR-22-3p | PATE3 | - | - | - | + |
| hsa-miR-22-3p | PATZ1 | - | - | - | + |
| hsa-miR-22-3p | PAX2 | - | - | - | + |
| hsa-miR-22-3p | PAX5 | + | - | - | + |
| hsa-miR-22-3p | PAX6 | - | - | - | + |
| hsa-miR-22-3p | PAX8 | + | - | - | + |
| hsa-miR-22-3p | PBRM1 | - | - | - | + |
| hsa-miR-22-3p | PBX1 | + | + | - | + |
| hsa-miR-22-3p | PBX2 | - | - | - | + |
| hsa-miR-22-3p | PBX3 | - | - | - | + |
| hsa-miR-22-3p | PCDH12 | - | - | - | + |
| hsa-miR-22-3p | PCDH19 | - | - | - | + |
| hsa-miR-22-3p | PCDH20 | - | - | - | + |
| hsa-miR-22-3p | PCDHA1 | - | - | - | + |
| hsa-miR-22-3p | PCDHA11 | - | - | - | + |
| hsa-miR-22-3p | PCDHA2 | - | - | - | + |
| hsa-miR-22-3p | PCDHA3 | - | - | - | + |
| hsa-miR-22-3p | PCDHA4 | - | - | - | + |
| hsa-miR-22-3p | PCDHA5 | - | - | - | + |
| hsa-miR-22-3p | PCDHA6 | - | - | - | + |
| hsa-miR-22-3p | PCDHA7 | - | - | - | + |
| hsa-miR-22-3p | PCDHA8 | - | - | - | + |
| hsa-miR-22-3p | PCDHAC2 | - | - | - | + |
| hsa-miR-22-3p | PCDHB3 | - | - | - | + |
| hsa-miR-22-3p | PCGF3 | - | - | - | + |
| hsa-miR-22-3p | PCGF5 | + | - | - | + |
| hsa-miR-22-3p | PCMT1 | - | - | - | + |
| hsa-miR-22-3p | PCNP | + | - | - | - |
| hsa-miR-22-3p | PCNX | - | - | - | + |
| hsa-miR-22-3p | PCP4L1 | + | - | - | + |
| hsa-miR-22-3p | PCSK5 | - | - | - | + |
| hsa-miR-22-3p | PCSK7 | - | - | - | + |
| hsa-miR-22-3p | PCTP | + | - | - | - |
| hsa-miR-22-3p | PCYT1B | - | - | - | + |
| hsa-miR-22-3p | PCYT2 | + | - | - | - |
| hsa-miR-22-3p | PDAP1 | + | - | - | + |
| hsa-miR-22-3p | PDCD2 | - | - | - | + |
| hsa-miR-22-3p | PDCD7 | - | - | - | + |
| hsa-miR-22-3p | PDE10A | - | - | - | + |
| hsa-miR-22-3p | PDE1A | + | - | - | - |
| hsa-miR-22-3p | PDE3A | - | - | - | + |
| hsa-miR-22-3p | PDE3B | - | - | - | + |
| hsa-miR-22-3p | PDE4D | - | - | - | + |
| hsa-miR-22-3p | PDE5A | - | - | - | + |
| hsa-miR-22-3p | PDE6A | - | - | - | + |
| hsa-miR-22-3p | PDGFB | - | - | - | + |
| hsa-miR-22-3p | PDGFC | + | - | - | - |
| hsa-miR-22-3p | PDGFD | - | - | - | + |
| hsa-miR-22-3p | PDHA1 | - | - | + | + |
| hsa-miR-22-3p | PDHA2 | - | - | - | + |
| hsa-miR-22-3p | PDIA3 | - | - | - | + |
| hsa-miR-22-3p | PDIA6 | - | - | - | + |
| hsa-miR-22-3p | PDIK1L | + | + | + | + |
| hsa-miR-22-3p | PDLIM1 | - | - | - | + |
| hsa-miR-22-3p | PDLIM2 | - | - | - | + |
| hsa-miR-22-3p | PDLIM4 | - | - | - | + |
| hsa-miR-22-3p | PDPK1 | - | - | - | + |
| hsa-miR-22-3p | PDPN | - | - | - | + |
| hsa-miR-22-3p | PDS5A | + | - | - | + |
| hsa-miR-22-3p | PDSS1 | + | + | - | - |
| hsa-miR-22-3p | PDXDC1 | - | - | - | + |
| hsa-miR-22-3p | PDYN | - | - | - | + |
| hsa-miR-22-3p | PDZD4 | - | - | - | + |
| hsa-miR-22-3p | PEAK1 | - | - | - | + |
| hsa-miR-22-3p | PEG10 | + | - | - | + |
| hsa-miR-22-3p | PEG3 | - | - | - | + |
| hsa-miR-22-3p | PELI2 | - | - | - | + |
| hsa-miR-22-3p | PER3 | - | - | - | + |
| hsa-miR-22-3p | PEX5 | - | - | + | + |
| hsa-miR-22-3p | PFDN6 | + | - | - | - |
| hsa-miR-22-3p | PFKFB2 | - | + | - | + |
| hsa-miR-22-3p | PFKFB3 | - | - | - | + |
| hsa-miR-22-3p | PFN2 | - | - | - | + |
| hsa-miR-22-3p | PGM3 | - | - | - | + |
| hsa-miR-22-3p | PGM5 | + | - | - | + |
| hsa-miR-22-3p | PGP | - | - | - | + |
| hsa-miR-22-3p | PGPEP1 | - | - | - | + |
| hsa-miR-22-3p | PGR | - | - | - | + |
| hsa-miR-22-3p | PHACTR2 | + | + | - | + |
| hsa-miR-22-3p | PHACTR4 | - | + | - | - |
| hsa-miR-22-3p | PHB | - | - | - | + |
| hsa-miR-22-3p | PHC1 | - | + | - | - |
| hsa-miR-22-3p | PHEX | - | - | - | + |
| hsa-miR-22-3p | PHF1 | - | - | - | + |
| hsa-miR-22-3p | PHF13 | + | + | - | + |
| hsa-miR-22-3p | PHF15 | - | - | - | + |
| hsa-miR-22-3p | PHF19 | - | - | - | + |
| hsa-miR-22-3p | PHF2 | + | - | - | - |
| hsa-miR-22-3p | PHF21B | - | - | - | + |
| hsa-miR-22-3p | PHF23 | - | - | - | + |
| hsa-miR-22-3p | PHF5A | + | - | - | + |
| hsa-miR-22-3p | PHF6 | - | - | - | + |
| hsa-miR-22-3p | PHF8 | + | + | - | + |
| hsa-miR-22-3p | PHKB | - | - | - | + |
| hsa-miR-22-3p | PHKG2 | - | - | - | + |
| hsa-miR-22-3p | PHLDA3 | + | - | - | - |
| hsa-miR-22-3p | PHLPP1 | - | - | - | + |
| hsa-miR-22-3p | PHLPP2 | - | - | - | + |
| hsa-miR-22-3p | PHOX2B | - | - | - | + |
| hsa-miR-22-3p | PHYHIP | + | - | - | + |
| hsa-miR-22-3p | PI15 | - | - | - | + |
| hsa-miR-22-3p | PI4K2A | - | - | - | + |
| hsa-miR-22-3p | PIANP | + | - | - | - |
| hsa-miR-22-3p | PIAS4 | - | - | - | + |
| hsa-miR-22-3p | PIBF1 | - | - | - | + |
| hsa-miR-22-3p | PICALM | - | - | - | + |
| hsa-miR-22-3p | PICK1 | - | - | - | + |
| hsa-miR-22-3p | PIGN | + | - | - | + |
| hsa-miR-22-3p | PIGP | - | - | + | - |
| hsa-miR-22-3p | PIK3AP1 | - | - | - | + |
| hsa-miR-22-3p | PIK3C2A | - | - | + | + |
| hsa-miR-22-3p | PIK3CD | + | - | - | - |
| hsa-miR-22-3p | PIK3IP1 | + | - | - | + |
| hsa-miR-22-3p | PIK3R6 | + | + | - | - |
| hsa-miR-22-3p | PIKFYVE | - | - | - | + |
| hsa-miR-22-3p | PIP4K2B | - | + | - | - |
| hsa-miR-22-3p | PIP5K1A | + | - | - | + |
| hsa-miR-22-3p | PIP5K1C | - | - | - | + |
| hsa-miR-22-3p | PITPNA | - | - | - | + |
| hsa-miR-22-3p | PITPNB | - | - | - | + |
| hsa-miR-22-3p | PITPNM2 | - | - | - | + |
| hsa-miR-22-3p | PIWIL3 | + | - | - | - |
| hsa-miR-22-3p | PKHD1 | - | - | - | + |
| hsa-miR-22-3p | PKLR | - | - | - | + |
| hsa-miR-22-3p | PKN3 | - | - | - | + |
| hsa-miR-22-3p | PLA1A | - | - | - | + |
| hsa-miR-22-3p | PLA2G12A | - | - | - | + |
| hsa-miR-22-3p | PLA2G2C | + | - | - | - |
| hsa-miR-22-3p | PLAG1 | - | - | - | + |
| hsa-miR-22-3p | PLAGL2 | + | - | - | + |
| hsa-miR-22-3p | PLB1 | + | - | - | - |
| hsa-miR-22-3p | PLBD2 | + | + | - | - |
| hsa-miR-22-3p | PLCL1 | + | - | - | - |
| hsa-miR-22-3p | PLCXD3 | + | - | - | + |
| hsa-miR-22-3p | PLD1 | + | - | - | - |
| hsa-miR-22-3p | PLD6 | - | - | - | + |
| hsa-miR-22-3p | PLEC | - | - | - | + |
| hsa-miR-22-3p | PLEK | - | - | - | + |
| hsa-miR-22-3p | PLEKHA1 | - | - | - | + |
| hsa-miR-22-3p | PLEKHA4 | - | - | - | + |
| hsa-miR-22-3p | PLEKHA6 | - | + | - | - |
| hsa-miR-22-3p | PLEKHA7 | - | - | - | + |
| hsa-miR-22-3p | PLEKHF1 | - | - | - | + |
| hsa-miR-22-3p | PLEKHF2 | - | - | - | + |
| hsa-miR-22-3p | PLEKHH1 | - | - | - | + |
| hsa-miR-22-3p | PLEKHH2 | - | - | - | + |
| hsa-miR-22-3p | PLEKHO2 | - | - | - | + |
| hsa-miR-22-3p | PLIN4 | - | - | - | + |
| hsa-miR-22-3p | PLK2 | - | - | - | + |
| hsa-miR-22-3p | PLK3 | - | - | - | + |
| hsa-miR-22-3p | PLN | - | - | - | + |
| hsa-miR-22-3p | PLS1 | - | - | - | + |
| hsa-miR-22-3p | PLTP | + | - | - | - |
| hsa-miR-22-3p | PLXDC1 | - | - | - | + |
| hsa-miR-22-3p | PLXNA1 | - | - | - | + |
| hsa-miR-22-3p | PLXNA4 | - | - | - | + |
| hsa-miR-22-3p | PLXNC1 | - | - | - | + |
| hsa-miR-22-3p | PMAIP1 | + | + | - | + |
| hsa-miR-22-3p | PMEPA1 | - | - | - | + |
| hsa-miR-22-3p | PML | + | - | - | - |
| hsa-miR-22-3p | PNISR | - | - | - | + |
| hsa-miR-22-3p | PNKD | - | - | - | + |
| hsa-miR-22-3p | PNMA5 | + | - | - | + |
| hsa-miR-22-3p | PNMA6C | - | - | - | + |
| hsa-miR-22-3p | PNMAL1 | - | - | - | + |
| hsa-miR-22-3p | PNN | - | - | - | + |
| hsa-miR-22-3p | PNPLA4 | - | - | - | + |
| hsa-miR-22-3p | PNRC1 | - | - | - | + |
| hsa-miR-22-3p | PNRC2 | - | - | - | + |
| hsa-miR-22-3p | POC1B | + | - | - | - |
| hsa-miR-22-3p | POC1B-GALNT4 | + | - | - | + |
| hsa-miR-22-3p | POGK | + | + | - | - |
| hsa-miR-22-3p | POGLUT1 | - | - | - | + |
| hsa-miR-22-3p | POGZ | - | - | - | + |
| hsa-miR-22-3p | POLA1 | - | - | - | + |
| hsa-miR-22-3p | POLDIP3 | - | - | - | + |
| hsa-miR-22-3p | POLE3 | - | - | - | + |
| hsa-miR-22-3p | POLR1A | + | - | - | - |
| hsa-miR-22-3p | POLR1E | - | - | - | + |
| hsa-miR-22-3p | POLR3A | - | - | - | + |
| hsa-miR-22-3p | POLR3G | - | - | - | + |
| hsa-miR-22-3p | POLR3H | - | - | - | + |
| hsa-miR-22-3p | POM121 | - | - | - | + |
| hsa-miR-22-3p | POMT2 | - | - | - | + |
| hsa-miR-22-3p | POPDC2 | - | - | - | + |
| hsa-miR-22-3p | POU3F2 | - | - | - | + |
| hsa-miR-22-3p | POU6F2 | - | - | - | + |
| hsa-miR-22-3p | PP2CE | - | + | - | - |
| hsa-miR-22-3p | PPAN-P2RY11 | - | - | - | + |
| hsa-miR-22-3p | PPARA | + | - | + | + |
| hsa-miR-22-3p | PPARD | - | - | - | + |
| hsa-miR-22-3p | PPARGC1B | + | + | - | + |
| hsa-miR-22-3p | PPCDC | - | - | - | + |
| hsa-miR-22-3p | PPFIA4 | + | - | - | + |
| hsa-miR-22-3p | PPHLN1 | - | - | - | + |
| hsa-miR-22-3p | PPIF | + | - | - | + |
| hsa-miR-22-3p | PPIL6 | - | - | - | + |
| hsa-miR-22-3p | PPM1A | - | - | - | + |
| hsa-miR-22-3p | PPM1B | - | - | - | + |
| hsa-miR-22-3p | PPM1F | + | - | - | + |
| hsa-miR-22-3p | PPM1G | - | - | - | + |
| hsa-miR-22-3p | PPM1H | - | - | - | + |
| hsa-miR-22-3p | PPM1K | + | + | - | - |
| hsa-miR-22-3p | PPM1L | + | - | - | - |
| hsa-miR-22-3p | PPP1R10 | - | - | - | + |
| hsa-miR-22-3p | PPP1R12A | - | - | - | + |
| hsa-miR-22-3p | PPP1R12B | + | - | - | - |
| hsa-miR-22-3p | PPP1R15B | + | + | - | + |
| hsa-miR-22-3p | PPP1R16B | + | - | - | - |
| hsa-miR-22-3p | PPP1R3B | + | + | - | + |
| hsa-miR-22-3p | PPP1R9A | - | - | - | + |
| hsa-miR-22-3p | PPP2R1B | - | + | - | + |
| hsa-miR-22-3p | PPP2R2B | - | - | - | + |
| hsa-miR-22-3p | PPP2R2C | - | - | - | + |
| hsa-miR-22-3p | PPP2R5A | - | - | - | + |
| hsa-miR-22-3p | PPP3R1 | + | - | - | + |
| hsa-miR-22-3p | PPP6C | - | - | - | + |
| hsa-miR-22-3p | PPT1 | + | - | - | + |
| hsa-miR-22-3p | PPTC7 | - | - | - | + |
| hsa-miR-22-3p | PRB1 | - | - | - | + |
| hsa-miR-22-3p | PRB2 | - | - | - | + |
| hsa-miR-22-3p | PRCD | + | - | - | - |
| hsa-miR-22-3p | PRDM11 | + | - | - | - |
| hsa-miR-22-3p | PRDM16 | - | - | - | + |
| hsa-miR-22-3p | PRDM4 | + | + | - | + |
| hsa-miR-22-3p | PRELID1 | + | - | - | - |
| hsa-miR-22-3p | PRELID2 | + | - | + | - |
| hsa-miR-22-3p | PRELP | + | - | - | + |
| hsa-miR-22-3p | PRICKLE2 | - | - | - | + |
| hsa-miR-22-3p | PRIMA1 | - | - | - | + |
| hsa-miR-22-3p | PRKAA1 | - | - | - | + |
| hsa-miR-22-3p | PRKAB1 | - | - | - | + |
| hsa-miR-22-3p | PRKACA | - | - | + | + |
| hsa-miR-22-3p | PRKACB | - | - | - | + |
| hsa-miR-22-3p | PRKAG2 | - | - | - | + |
| hsa-miR-22-3p | PRKAR2A | + | - | - | + |
| hsa-miR-22-3p | PRKCA | - | - | - | + |
| hsa-miR-22-3p | PRKCB | + | - | - | + |
| hsa-miR-22-3p | PRKCE | - | - | - | + |
| hsa-miR-22-3p | PRKG1 | - | - | - | + |
| hsa-miR-22-3p | PRMT8 | - | - | - | + |
| hsa-miR-22-3p | PRNP | + | - | - | + |
| hsa-miR-22-3p | PRNT | + | - | - | - |
| hsa-miR-22-3p | PROK1 | - | - | - | + |
| hsa-miR-22-3p | PROK2 | - | - | - | + |
| hsa-miR-22-3p | PROS1 | - | + | - | + |
| hsa-miR-22-3p | PROX1 | + | + | - | + |
| hsa-miR-22-3p | PRPF38A | + | + | - | - |
| hsa-miR-22-3p | PRPF4B | - | - | - | + |
| hsa-miR-22-3p | PRPS1 | - | - | - | + |
| hsa-miR-22-3p | PRPS2 | - | - | - | + |
| hsa-miR-22-3p | PRR12 | - | - | - | + |
| hsa-miR-22-3p | PRR23C | - | - | - | + |
| hsa-miR-22-3p | PRR24 | + | - | - | - |
| hsa-miR-22-3p | PRR9 | - | - | - | + |
| hsa-miR-22-3p | PRRC1 | - | + | - | - |
| hsa-miR-22-3p | PRRC2C | + | + | - | + |
| hsa-miR-22-3p | PRRG4 | - | - | - | + |
| hsa-miR-22-3p | PRRT2 | - | - | - | + |
| hsa-miR-22-3p | PRRT3 | - | - | - | + |
| hsa-miR-22-3p | PRRX1 | - | - | - | + |
| hsa-miR-22-3p | PRSS12 | - | - | - | + |
| hsa-miR-22-3p | PRSS16 | - | - | - | + |
| hsa-miR-22-3p | PRTFDC1 | + | - | - | + |
| hsa-miR-22-3p | PRTG | - | - | - | + |
| hsa-miR-22-3p | PRX | + | - | - | + |
| hsa-miR-22-3p | PSD2 | + | - | - | + |
| hsa-miR-22-3p | PSD3 | - | - | - | + |
| hsa-miR-22-3p | PSD4 | + | + | - | - |
| hsa-miR-22-3p | PSEN1 | + | - | - | + |
| hsa-miR-22-3p | PSKH1 | - | - | - | + |
| hsa-miR-22-3p | PSMA5 | - | - | - | + |
| hsa-miR-22-3p | PSMB11 | - | - | - | + |
| hsa-miR-22-3p | PSMD9 | - | - | - | + |
| hsa-miR-22-3p | PSME3 | - | - | - | + |
| hsa-miR-22-3p | PSMF1 | - | - | - | + |
| hsa-miR-22-3p | PTBP3 | - | - | - | + |
| hsa-miR-22-3p | PTCD1 | - | - | - | + |
| hsa-miR-22-3p | PTCD3 | - | - | - | + |
| hsa-miR-22-3p | PTCHD2 | - | - | - | + |
| hsa-miR-22-3p | PTEN | + | + | + | + |
| hsa-miR-22-3p | PTGDR2 | + | - | - | + |
| hsa-miR-22-3p | PTGER3 | + | - | - | + |
| hsa-miR-22-3p | PTGER4 | - | - | - | + |
| hsa-miR-22-3p | PTGES3 | - | - | - | + |
| hsa-miR-22-3p | PTGES3L | + | - | - | - |
| hsa-miR-22-3p | PTGFR | - | - | - | + |
| hsa-miR-22-3p | PTGFRN | - | - | - | + |
| hsa-miR-22-3p | PTGS1 | + | + | - | + |
| hsa-miR-22-3p | PTMS | - | - | + | - |
| hsa-miR-22-3p | PTP4A3 | + | + | - | - |
| hsa-miR-22-3p | PTPLAD1 | - | - | - | + |
| hsa-miR-22-3p | PTPN1 | - | - | - | + |
| hsa-miR-22-3p | PTPN20 | - | - | - | + |
| hsa-miR-22-3p | PTPN4 | - | - | - | + |
| hsa-miR-22-3p | PTPN9 | - | - | - | + |
| hsa-miR-22-3p | PTPRD | - | - | - | + |
| hsa-miR-22-3p | PTPRN | - | - | - | + |
| hsa-miR-22-3p | PTPRN2 | - | - | - | + |
| hsa-miR-22-3p | PTPRS | - | - | - | + |
| hsa-miR-22-3p | PTPRT | - | - | - | + |
| hsa-miR-22-3p | PTRF | - | - | - | + |
| hsa-miR-22-3p | PURB | - | - | - | + |
| hsa-miR-22-3p | PVRL1 | - | - | - | + |
| hsa-miR-22-3p | PWWP2B | - | - | - | + |
| hsa-miR-22-3p | PXDN | - | - | - | + |
| hsa-miR-22-3p | PYGO2 | - | - | - | + |
| hsa-miR-22-3p | PYROXD2 | - | - | - | + |
| hsa-miR-22-3p | QKI | - | - | - | + |
| hsa-miR-22-3p | QSER1 | - | - | - | + |
| hsa-miR-22-3p | QSOX2 | - | - | - | + |
| hsa-miR-22-3p | RAB10 | - | - | - | + |
| hsa-miR-22-3p | RAB11A | - | - | - | + |
| hsa-miR-22-3p | RAB11FIP1 | - | - | - | + |
| hsa-miR-22-3p | RAB11FIP3 | - | - | - | + |
| hsa-miR-22-3p | RAB11FIP4 | + | - | - | - |
| hsa-miR-22-3p | RAB11FIP5 | - | - | - | + |
| hsa-miR-22-3p | RAB18 | - | - | - | + |
| hsa-miR-22-3p | RAB1A | - | - | - | + |
| hsa-miR-22-3p | RAB1B | - | - | - | + |
| hsa-miR-22-3p | RAB26 | + | - | - | + |
| hsa-miR-22-3p | RAB27A | - | - | - | + |
| hsa-miR-22-3p | RAB28 | + | + | - | - |
| hsa-miR-22-3p | RAB33B | - | - | - | + |
| hsa-miR-22-3p | RAB35 | + | + | - | + |
| hsa-miR-22-3p | RAB36 | - | - | - | + |
| hsa-miR-22-3p | RAB3B | - | - | - | + |
| hsa-miR-22-3p | RAB3C | + | + | - | + |
| hsa-miR-22-3p | RAB3D | - | - | - | + |
| hsa-miR-22-3p | RAB3GAP1 | - | - | - | + |
| hsa-miR-22-3p | RAB3GAP2 | - | - | - | + |
| hsa-miR-22-3p | RAB3IP | - | - | - | + |
| hsa-miR-22-3p | RAB40C | + | - | - | + |
| hsa-miR-22-3p | RAB42 | + | - | - | - |
| hsa-miR-22-3p | RAB43 | - | - | - | + |
| hsa-miR-22-3p | RAB44 | - | - | + | - |
| hsa-miR-22-3p | RAB4A | - | - | - | + |
| hsa-miR-22-3p | RAB5B | + | + | + | - |
| hsa-miR-22-3p | RAB5C | + | - | - | - |
| hsa-miR-22-3p | RABEP2 | + | - | - | + |
| hsa-miR-22-3p | RABGAP1L | - | - | - | + |
| hsa-miR-22-3p | RABGEF1 | + | + | - | + |
| hsa-miR-22-3p | RAC1 | - | - | - | + |
| hsa-miR-22-3p | RAD18 | - | - | - | + |
| hsa-miR-22-3p | RAD21 | - | - | - | + |
| hsa-miR-22-3p | RAD23B | - | - | - | + |
| hsa-miR-22-3p | RAD51 | - | - | - | + |
| hsa-miR-22-3p | RAD51B | - | + | - | + |
| hsa-miR-22-3p | RAD9A | - | - | - | + |
| hsa-miR-22-3p | RAET1E | - | - | - | + |
| hsa-miR-22-3p | RAI14 | - | - | - | + |
| hsa-miR-22-3p | RAI2 | - | - | - | + |
| hsa-miR-22-3p | RALA | - | - | - | + |
| hsa-miR-22-3p | RAMP1 | + | - | - | - |
| hsa-miR-22-3p | RAN | + | - | - | + |
| hsa-miR-22-3p | RANBP10 | - | - | - | + |
| hsa-miR-22-3p | RANBP2 | - | - | - | + |
| hsa-miR-22-3p | RANGAP1 | + | - | - | - |
| hsa-miR-22-3p | RAP1A | - | - | - | + |
| hsa-miR-22-3p | RAP1GAP2 | - | - | - | + |
| hsa-miR-22-3p | RAP2B | + | - | + | - |
| hsa-miR-22-3p | RAPGEF2 | - | - | - | + |
| hsa-miR-22-3p | RAPGEF3 | - | - | - | + |
| hsa-miR-22-3p | RAPGEFL1 | + | - | - | - |
| hsa-miR-22-3p | RAPH1 | - | - | - | + |
| hsa-miR-22-3p | RARG | - | - | - | + |
| hsa-miR-22-3p | RASA2 | + | - | - | + |
| hsa-miR-22-3p | RASAL1 | - | - | - | + |
| hsa-miR-22-3p | RASD2 | - | - | - | + |
| hsa-miR-22-3p | RASEF | - | - | - | + |
| hsa-miR-22-3p | RASGRF1 | - | - | - | + |
| hsa-miR-22-3p | RASGRF2 | - | - | - | + |
| hsa-miR-22-3p | RASL10B | + | - | - | - |
| hsa-miR-22-3p | RASL11A | + | - | - | - |
| hsa-miR-22-3p | RASSF3 | - | + | - | + |
| hsa-miR-22-3p | RASSF8 | - | - | - | + |
| hsa-miR-22-3p | RAVER1 | - | - | - | + |
| hsa-miR-22-3p | RBFOX1 | - | - | - | + |
| hsa-miR-22-3p | RBFOX2 | - | - | - | + |
| hsa-miR-22-3p | RBFOX3 | - | - | - | + |
| hsa-miR-22-3p | RBL1 | - | - | + | - |
| hsa-miR-22-3p | RBM15 | + | + | - | - |
| hsa-miR-22-3p | RBM17 | - | - | - | + |
| hsa-miR-22-3p | RBM19 | - | - | - | + |
| hsa-miR-22-3p | RBM24 | - | - | - | + |
| hsa-miR-22-3p | RBM27 | - | - | - | + |
| hsa-miR-22-3p | RBM39 | - | - | + | - |
| hsa-miR-22-3p | RBM46 | - | - | - | + |
| hsa-miR-22-3p | RBMS2 | - | - | - | + |
| hsa-miR-22-3p | RBMXL1 | + | + | - | + |
| hsa-miR-22-3p | RBP3 | + | - | - | - |
| hsa-miR-22-3p | RBP7 | - | - | - | + |
| hsa-miR-22-3p | RBPJL | - | - | - | + |
| hsa-miR-22-3p | RC3H1 | - | - | - | + |
| hsa-miR-22-3p | RCBTB1 | - | - | - | + |
| hsa-miR-22-3p | RCBTB2 | - | - | - | + |
| hsa-miR-22-3p | RCC2 | + | + | + | + |
| hsa-miR-22-3p | RCL1 | - | - | - | + |
| hsa-miR-22-3p | RCOR1 | + | + | + | - |
| hsa-miR-22-3p | RCOR2 | + | - | - | + |
| hsa-miR-22-3p | RCSD1 | - | - | - | + |
| hsa-miR-22-3p | RD3 | - | - | - | + |
| hsa-miR-22-3p | REEP1 | - | - | - | + |
| hsa-miR-22-3p | REEP2 | - | - | - | + |
| hsa-miR-22-3p | RELL2 | + | - | - | - |
| hsa-miR-22-3p | REPS2 | - | - | - | + |
| hsa-miR-22-3p | REST | - | - | - | + |
| hsa-miR-22-3p | RET | - | - | - | + |
| hsa-miR-22-3p | RFC3 | - | - | - | + |
| hsa-miR-22-3p | RFT1 | - | - | - | + |
| hsa-miR-22-3p | RFTN1 | - | - | - | + |
| hsa-miR-22-3p | RFXANK | + | + | - | - |
| hsa-miR-22-3p | RGL4 | + | - | - | - |
| hsa-miR-22-3p | RGP1 | + | + | - | - |
| hsa-miR-22-3p | RGS10 | + | - | - | - |
| hsa-miR-22-3p | RGS14 | + | + | - | + |
| hsa-miR-22-3p | RGS2 | + | + | + | + |
| hsa-miR-22-3p | RGS3 | - | - | - | + |
| hsa-miR-22-3p | RGS4 | - | - | - | + |
| hsa-miR-22-3p | RGS5 | - | - | - | + |
| hsa-miR-22-3p | RGS7BP | - | - | - | + |
| hsa-miR-22-3p | RGS9BP | + | - | - | + |
| hsa-miR-22-3p | RHAG | - | - | - | + |
| hsa-miR-22-3p | RHBDD1 | + | - | - | + |
| hsa-miR-22-3p | RHBDL3 | - | - | - | + |
| hsa-miR-22-3p | RHCG | + | - | - | - |
| hsa-miR-22-3p | RHNO1 | - | - | - | + |
| hsa-miR-22-3p | RHO | - | - | - | + |
| hsa-miR-22-3p | RHOBTB1 | - | - | - | + |
| hsa-miR-22-3p | RHOG | - | - | - | + |
| hsa-miR-22-3p | RHOU | - | - | - | + |
| hsa-miR-22-3p | RHOV | + | + | - | - |
| hsa-miR-22-3p | RIBC1 | - | - | - | + |
| hsa-miR-22-3p | RIC8B | + | - | - | + |
| hsa-miR-22-3p | RIMBP2 | - | - | - | + |
| hsa-miR-22-3p | RIMS1 | - | - | - | + |
| hsa-miR-22-3p | RIMS3 | - | - | - | + |
| hsa-miR-22-3p | RIMS4 | + | - | - | + |
| hsa-miR-22-3p | RING1 | - | - | - | + |
| hsa-miR-22-3p | RIOK2 | - | - | - | + |
| hsa-miR-22-3p | RIOK3 | - | - | - | + |
| hsa-miR-22-3p | RIPK1 | + | - | - | - |
| hsa-miR-22-3p | RIT1 | - | - | - | + |
| hsa-miR-22-3p | RMDN3 | + | + | - | - |
| hsa-miR-22-3p | RMND5A | + | - | + | + |
| hsa-miR-22-3p | RND1 | - | - | - | + |
| hsa-miR-22-3p | RND2 | + | - | - | - |
| hsa-miR-22-3p | RNF10 | - | - | - | + |
| hsa-miR-22-3p | RNF11 | - | - | - | + |
| hsa-miR-22-3p | RNF112 | - | - | - | + |
| hsa-miR-22-3p | RNF13 | - | - | - | + |
| hsa-miR-22-3p | RNF144A | - | - | - | + |
| hsa-miR-22-3p | RNF150 | + | - | - | - |
| hsa-miR-22-3p | RNF157 | + | - | - | + |
| hsa-miR-22-3p | RNF169 | + | - | - | + |
| hsa-miR-22-3p | RNF185 | + | + | - | + |
| hsa-miR-22-3p | RNF20 | - | - | - | + |
| hsa-miR-22-3p | RNF212 | - | - | - | + |
| hsa-miR-22-3p | RNF213 | - | - | - | + |
| hsa-miR-22-3p | RNF215 | + | + | - | + |
| hsa-miR-22-3p | RNF216 | + | - | - | + |
| hsa-miR-22-3p | RNF224 | - | - | - | + |
| hsa-miR-22-3p | RNF26 | - | - | - | + |
| hsa-miR-22-3p | RNF38 | + | - | - | + |
| hsa-miR-22-3p | RNF44 | + | - | - | + |
| hsa-miR-22-3p | RNF8 | - | - | - | + |
| hsa-miR-22-3p | ROBO2 | - | - | - | + |
| hsa-miR-22-3p | ROCK2 | - | - | - | + |
| hsa-miR-22-3p | ROR2 | + | - | - | + |
| hsa-miR-22-3p | RORA | - | - | - | + |
| hsa-miR-22-3p | RORC | - | - | - | + |
| hsa-miR-22-3p | RP1-37E16.12 | - | - | - | + |
| hsa-miR-22-3p | RP11-1026M7.2 | + | - | - | - |
| hsa-miR-22-3p | RP11-111H3.1 | - | - | - | + |
| hsa-miR-22-3p | RP11-131H24.4 | + | - | - | - |
| hsa-miR-22-3p | RP11-17M16.1 | + | - | - | - |
| hsa-miR-22-3p | RP11-321M21.3 | + | - | - | - |
| hsa-miR-22-3p | RP11-362K2.2 | + | - | - | - |
| hsa-miR-22-3p | RP11-422N16.3 | + | - | - | + |
| hsa-miR-22-3p | RP11-527L4.2 | + | - | - | - |
| hsa-miR-22-3p | RP11-579D7.1 | - | - | - | + |
| hsa-miR-22-3p | RP11-744N12.3 | - | - | - | + |
| hsa-miR-22-3p | RP11-796G6.2 | - | - | - | + |
| hsa-miR-22-3p | RP11-80A15.1 | + | - | - | - |
| hsa-miR-22-3p | RP11-826N14.2 | + | - | - | + |
| hsa-miR-22-3p | RP11-865B13.1 | + | - | - | - |
| hsa-miR-22-3p | RP2 | - | - | - | + |
| hsa-miR-22-3p | RP4-604K5.1 | - | - | - | + |
| hsa-miR-22-3p | RP4-758J18.2 | + | - | - | - |
| hsa-miR-22-3p | RP5-1187M17.10 | - | - | - | + |
| hsa-miR-22-3p | RP5-850E9.3 | + | - | - | - |
| hsa-miR-22-3p | RPL13 | - | + | - | - |
| hsa-miR-22-3p | RPL14 | + | - | - | - |
| hsa-miR-22-3p | RPL24 | - | - | + | - |
| hsa-miR-22-3p | RPL27A | - | - | - | + |
| hsa-miR-22-3p | RPL28 | - | - | - | + |
| hsa-miR-22-3p | RPL35A | - | - | + | - |
| hsa-miR-22-3p | RPL36A-HNRNPH2 | + | - | - | - |
| hsa-miR-22-3p | RPL3L | + | - | - | - |
| hsa-miR-22-3p | RPN2 | - | - | - | + |
| hsa-miR-22-3p | RPP14 | + | - | - | - |
| hsa-miR-22-3p | RPRD1B | - | - | - | + |
| hsa-miR-22-3p | RPRD2 | - | - | - | + |
| hsa-miR-22-3p | RPRML | + | + | - | + |
| hsa-miR-22-3p | RPS2 | - | - | + | - |
| hsa-miR-22-3p | RPS3 | + | - | - | - |
| hsa-miR-22-3p | RPS4X | - | - | + | - |
| hsa-miR-22-3p | RPS6KA3 | - | - | - | + |
| hsa-miR-22-3p | RPS6KC1 | + | + | - | - |
| hsa-miR-22-3p | RPS9 | + | - | - | - |
| hsa-miR-22-3p | RPSA | - | - | + | - |
| hsa-miR-22-3p | RPTOR | - | - | - | + |
| hsa-miR-22-3p | RRAGD | - | - | - | + |
| hsa-miR-22-3p | RRN3 | - | - | - | + |
| hsa-miR-22-3p | RRP1B | - | - | - | + |
| hsa-miR-22-3p | RRP36 | + | - | - | - |
| hsa-miR-22-3p | RS1 | - | - | - | + |
| hsa-miR-22-3p | RSBN1 | + | + | - | + |
| hsa-miR-22-3p | RSF1 | - | - | - | + |
| hsa-miR-22-3p | RSL1D1 | - | - | - | + |
| hsa-miR-22-3p | RSPH10B | + | - | - | - |
| hsa-miR-22-3p | RSPO1 | + | - | - | - |
| hsa-miR-22-3p | RSPO4 | - | - | - | + |
| hsa-miR-22-3p | RSPRY1 | - | - | - | + |
| hsa-miR-22-3p | RTF1 | - | - | - | + |
| hsa-miR-22-3p | RTKN | - | - | - | + |
| hsa-miR-22-3p | RTN3 | - | - | - | + |
| hsa-miR-22-3p | RTP1 | - | - | - | + |
| hsa-miR-22-3p | RTTN | + | - | - | - |
| hsa-miR-22-3p | RUFY2 | + | + | - | + |
| hsa-miR-22-3p | RUNDC1 | - | - | - | + |
| hsa-miR-22-3p | RUNX2 | + | + | - | + |
| hsa-miR-22-3p | RUNX3 | - | - | - | + |
| hsa-miR-22-3p | RUSC2 | - | - | - | + |
| hsa-miR-22-3p | RWDD2B | - | - | - | + |
| hsa-miR-22-3p | RWDD4 | - | - | - | + |
| hsa-miR-22-3p | RXFP2 | + | - | - | + |
| hsa-miR-22-3p | RYR1 | - | - | - | + |
| hsa-miR-22-3p | RYR3 | - | - | - | + |
| hsa-miR-22-3p | S100A10 | - | - | - | + |
| hsa-miR-22-3p | S100PBP | - | - | - | + |
| hsa-miR-22-3p | S100Z | - | - | - | + |
| hsa-miR-22-3p | SAMD10 | - | - | - | + |
| hsa-miR-22-3p | SAMD12 | + | - | - | + |
| hsa-miR-22-3p | SAMD14 | + | - | - | - |
| hsa-miR-22-3p | SAMD5 | + | - | - | + |
| hsa-miR-22-3p | SAPCD1 | - | - | - | + |
| hsa-miR-22-3p | SAPCD2 | + | - | - | + |
| hsa-miR-22-3p | SAR1B | - | - | - | + |
| hsa-miR-22-3p | SARM1 | + | - | - | - |
| hsa-miR-22-3p | SASH1 | - | - | - | + |
| hsa-miR-22-3p | SATB2 | + | + | - | + |
| hsa-miR-22-3p | SAYSD1 | + | - | - | + |
| hsa-miR-22-3p | SBDS | + | - | - | - |
| hsa-miR-22-3p | SBF2 | - | - | - | + |
| hsa-miR-22-3p | SBK1 | + | - | - | + |
| hsa-miR-22-3p | SCAF8 | - | - | - | + |
| hsa-miR-22-3p | SCAMP1 | + | - | - | - |
| hsa-miR-22-3p | SCAMP3 | - | - | - | + |
| hsa-miR-22-3p | SCAMP5 | - | - | - | + |
| hsa-miR-22-3p | SCARA3 | + | - | - | + |
| hsa-miR-22-3p | SCCPDH | - | - | - | + |
| hsa-miR-22-3p | SCD | + | - | + | + |
| hsa-miR-22-3p | SCGN | + | - | - | - |
| hsa-miR-22-3p | SCN2B | - | - | - | + |
| hsa-miR-22-3p | SCN3A | - | - | - | + |
| hsa-miR-22-3p | SCRN1 | - | - | - | + |
| hsa-miR-22-3p | SCUBE3 | - | - | - | + |
| hsa-miR-22-3p | SCYL2 | - | - | - | + |
| hsa-miR-22-3p | SCYL3 | + | + | - | + |
| hsa-miR-22-3p | SDC1 | - | - | - | + |
| hsa-miR-22-3p | SDC4 | - | - | - | + |
| hsa-miR-22-3p | SDHD | - | - | - | + |
| hsa-miR-22-3p | SDK2 | - | - | - | + |
| hsa-miR-22-3p | SEC14L1 | + | - | - | - |
| hsa-miR-22-3p | SEC14L4 | - | + | - | - |
| hsa-miR-22-3p | SEC14L5 | - | - | - | + |
| hsa-miR-22-3p | SEC23B | - | + | - | + |
| hsa-miR-22-3p | SEC61A1 | - | - | - | + |
| hsa-miR-22-3p | SEC62 | - | - | - | + |
| hsa-miR-22-3p | SEC63 | - | - | - | + |
| hsa-miR-22-3p | SECISBP2L | - | - | - | + |
| hsa-miR-22-3p | SEL1L | - | - | - | + |
| hsa-miR-22-3p | SELL | - | - | - | + |
| hsa-miR-22-3p | SELPLG | + | - | - | - |
| hsa-miR-22-3p | SELRC1 | + | - | - | - |
| hsa-miR-22-3p | SEMA3A | - | - | - | + |
| hsa-miR-22-3p | SEMA3D | - | - | - | + |
| hsa-miR-22-3p | SEMA4C | - | - | - | + |
| hsa-miR-22-3p | SEMA5A | - | - | - | + |
| hsa-miR-22-3p | SEMA6A | - | - | - | + |
| hsa-miR-22-3p | SEMA6D | - | - | - | + |
| hsa-miR-22-3p | SEMA7A | - | - | - | + |
| hsa-miR-22-3p | SENP5 | - | - | - | + |
| hsa-miR-22-3p | SENP7 | - | - | - | + |
| hsa-miR-22-3p | SEPHS1 | + | + | - | + |
| hsa-miR-22-3p | SEPP1 | - | - | - | + |
| hsa-miR-22-3p | 10-Sep | - | - | - | + |
| hsa-miR-22-3p | 8-Sep | - | - | - | + |
| hsa-miR-22-3p | SERBP1 | + | + | + | + |
| hsa-miR-22-3p | SERF1A | - | - | - | + |
| hsa-miR-22-3p | SERF1B | - | - | - | + |
| hsa-miR-22-3p | SERINC5 | - | + | - | - |
| hsa-miR-22-3p | SESN1 | + | + | - | + |
| hsa-miR-22-3p | SESN2 | + | - | - | + |
| hsa-miR-22-3p | SESN3 | - | - | - | + |
| hsa-miR-22-3p | SETD1A | - | - | - | + |
| hsa-miR-22-3p | SF3A3 | - | - | - | + |
| hsa-miR-22-3p | SF3B3 | - | - | - | + |
| hsa-miR-22-3p | SFMBT2 | - | - | - | + |
| hsa-miR-22-3p | SFRP1 | - | - | - | + |
| hsa-miR-22-3p | SFT2D1 | - | - | - | + |
| hsa-miR-22-3p | SFXN1 | - | - | + | + |
| hsa-miR-22-3p | SFXN2 | + | - | - | - |
| hsa-miR-22-3p | SFXN5 | - | - | - | + |
| hsa-miR-22-3p | SGCB | - | - | - | + |
| hsa-miR-22-3p | SGCG | - | - | - | + |
| hsa-miR-22-3p | SGMS1 | - | - | - | + |
| hsa-miR-22-3p | SGPL1 | - | - | - | + |
| hsa-miR-22-3p | SGSM1 | - | - | - | + |
| hsa-miR-22-3p | SGTA | - | - | - | + |
| hsa-miR-22-3p | SH2B3 | - | - | - | + |
| hsa-miR-22-3p | SH2D1A | - | - | - | + |
| hsa-miR-22-3p | SH2D1B | + | - | - | + |
| hsa-miR-22-3p | SH2D4B | - | - | - | + |
| hsa-miR-22-3p | SH3BP2 | - | - | - | + |
| hsa-miR-22-3p | SH3D19 | - | - | - | + |
| hsa-miR-22-3p | SH3GL1 | - | - | - | + |
| hsa-miR-22-3p | SH3PXD2A | + | - | - | + |
| hsa-miR-22-3p | SH3PXD2B | - | - | - | + |
| hsa-miR-22-3p | SH3RF1 | - | - | - | + |
| hsa-miR-22-3p | SHCBP1 | - | - | - | + |
| hsa-miR-22-3p | SHF | + | - | - | - |
| hsa-miR-22-3p | SHISA5 | + | - | - | - |
| hsa-miR-22-3p | SHISA6 | - | - | - | + |
| hsa-miR-22-3p | SHISA7 | - | - | - | + |
| hsa-miR-22-3p | SHISA9 | - | + | - | + |
| hsa-miR-22-3p | SHKBP1 | - | - | - | + |
| hsa-miR-22-3p | SHPK | - | - | - | + |
| hsa-miR-22-3p | SHROOM2 | - | - | - | + |
| hsa-miR-22-3p | SHROOM4 | - | - | - | + |
| hsa-miR-22-3p | SIDT1 | + | - | - | + |
| hsa-miR-22-3p | SIGMAR1 | + | - | - | - |
| hsa-miR-22-3p | SIPA1L1 | - | - | - | + |
| hsa-miR-22-3p | SIRT1 | + | + | - | + |
| hsa-miR-22-3p | SIRT5 | - | + | - | + |
| hsa-miR-22-3p | SIX3 | - | - | - | + |
| hsa-miR-22-3p | SIX5 | + | - | - | - |
| hsa-miR-22-3p | SKA1 | - | - | - | + |
| hsa-miR-22-3p | SKA2 | - | - | - | + |
| hsa-miR-22-3p | SKAP2 | - | - | - | + |
| hsa-miR-22-3p | SKI | - | - | - | + |
| hsa-miR-22-3p | SKIDA1 | - | - | - | + |
| hsa-miR-22-3p | SKIL | - | - | - | + |
| hsa-miR-22-3p | SLAMF8 | - | - | - | + |
| hsa-miR-22-3p | SLC10A3 | - | - | - | + |
| hsa-miR-22-3p | SLC10A7 | - | - | - | + |
| hsa-miR-22-3p | SLC12A1 | - | - | - | + |
| hsa-miR-22-3p | SLC12A4 | - | - | - | + |
| hsa-miR-22-3p | SLC14A1 | - | - | - | + |
| hsa-miR-22-3p | SLC15A2 | - | - | - | + |
| hsa-miR-22-3p | SLC16A10 | - | - | - | + |
| hsa-miR-22-3p | SLC16A14 | + | - | - | + |
| hsa-miR-22-3p | SLC16A2 | + | - | - | + |
| hsa-miR-22-3p | SLC17A5 | - | - | - | + |
| hsa-miR-22-3p | SLC17A6 | - | - | - | + |
| hsa-miR-22-3p | SLC17A7 | - | - | - | + |
| hsa-miR-22-3p | SLC18B1 | - | - | - | + |
| hsa-miR-22-3p | SLC1A3 | + | + | - | + |
| hsa-miR-22-3p | SLC20A1 | - | - | - | + |
| hsa-miR-22-3p | SLC20A2 | - | - | - | + |
| hsa-miR-22-3p | SLC22A11 | + | - | - | + |
| hsa-miR-22-3p | SLC22A12 | - | - | - | + |
| hsa-miR-22-3p | SLC22A14 | - | - | - | + |
| hsa-miR-22-3p | SLC22A15 | - | - | - | + |
| hsa-miR-22-3p | SLC22A23 | + | + | - | + |
| hsa-miR-22-3p | SLC24A2 | + | - | - | - |
| hsa-miR-22-3p | SLC24A4 | + | - | - | - |
| hsa-miR-22-3p | SLC25A10 | + | - | - | - |
| hsa-miR-22-3p | SLC25A11 | - | - | - | + |
| hsa-miR-22-3p | SLC25A13 | - | - | - | + |
| hsa-miR-22-3p | SLC25A14 | - | - | - | + |
| hsa-miR-22-3p | SLC25A18 | - | - | - | + |
| hsa-miR-22-3p | SLC25A22 | + | - | - | + |
| hsa-miR-22-3p | SLC25A30 | - | - | - | + |
| hsa-miR-22-3p | SLC25A36 | - | - | - | + |
| hsa-miR-22-3p | SLC25A42 | - | - | - | + |
| hsa-miR-22-3p | SLC25A53 | + | - | - | - |
| hsa-miR-22-3p | SLC26A4 | - | - | - | + |
| hsa-miR-22-3p | SLC26A9 | - | + | - | + |
| hsa-miR-22-3p | SLC28A1 | + | - | - | + |
| hsa-miR-22-3p | SLC28A3 | - | - | - | + |
| hsa-miR-22-3p | SLC2A1 | + | - | + | + |
| hsa-miR-22-3p | SLC2A13 | - | - | - | + |
| hsa-miR-22-3p | SLC2A6 | + | - | - | + |
| hsa-miR-22-3p | SLC30A4 | - | + | - | - |
| hsa-miR-22-3p | SLC30A7 | - | - | - | + |
| hsa-miR-22-3p | SLC30A8 | + | + | - | + |
| hsa-miR-22-3p | SLC31A1 | + | - | - | + |
| hsa-miR-22-3p | SLC35A2 | - | - | - | + |
| hsa-miR-22-3p | SLC35B4 | - | - | - | + |
| hsa-miR-22-3p | SLC35C2 | - | - | - | + |
| hsa-miR-22-3p | SLC35D1 | - | - | - | + |
| hsa-miR-22-3p | SLC35E4 | + | - | - | - |
| hsa-miR-22-3p | SLC35G3 | - | - | - | + |
| hsa-miR-22-3p | SLC37A2 | + | - | - | - |
| hsa-miR-22-3p | SLC38A11 | + | - | - | - |
| hsa-miR-22-3p | SLC38A7 | + | - | - | - |
| hsa-miR-22-3p | SLC39A11 | - | - | - | + |
| hsa-miR-22-3p | SLC39A12 | - | - | - | + |
| hsa-miR-22-3p | SLC39A14 | - | - | - | + |
| hsa-miR-22-3p | SLC39A2 | + | - | - | + |
| hsa-miR-22-3p | SLC39A6 | - | - | - | + |
| hsa-miR-22-3p | SLC45A3 | - | - | - | + |
| hsa-miR-22-3p | SLC45A4 | - | - | - | + |
| hsa-miR-22-3p | SLC46A2 | - | - | - | + |
| hsa-miR-22-3p | SLC48A1 | + | + | - | + |
| hsa-miR-22-3p | SLC4A11 | - | - | - | + |
| hsa-miR-22-3p | SLC4A7 | - | - | - | + |
| hsa-miR-22-3p | SLC4A9 | + | - | - | - |
| hsa-miR-22-3p | SLC5A3 | - | - | - | + |
| hsa-miR-22-3p | SLC5A7 | - | - | - | + |
| hsa-miR-22-3p | SLC6A1 | + | + | - | + |
| hsa-miR-22-3p | SLC6A12 | - | - | - | + |
| hsa-miR-22-3p | SLC6A17 | + | + | - | + |
| hsa-miR-22-3p | SLC6A2 | - | - | - | + |
| hsa-miR-22-3p | SLC6A3 | - | - | - | + |
| hsa-miR-22-3p | SLC6A8 | + | - | - | + |
| hsa-miR-22-3p | SLC7A1 | - | - | - | + |
| hsa-miR-22-3p | SLC7A14 | + | - | - | + |
| hsa-miR-22-3p | SLC7A2 | + | - | - | + |
| hsa-miR-22-3p | SLC7A6 | - | - | - | + |
| hsa-miR-22-3p | SLC7A7 | + | + | - | - |
| hsa-miR-22-3p | SLC9A1 | + | + | - | - |
| hsa-miR-22-3p | SLC9A2 | - | - | - | + |
| hsa-miR-22-3p | SLC9A5 | + | + | - | - |
| hsa-miR-22-3p | SLC9A6 | + | + | - | + |
| hsa-miR-22-3p | SLC9A8 | - | - | - | + |
| hsa-miR-22-3p | SLC9B2 | - | - | - | + |
| hsa-miR-22-3p | SLCO1B1 | - | - | - | + |
| hsa-miR-22-3p | SLCO2B1 | - | - | - | + |
| hsa-miR-22-3p | SLFN5 | + | + | - | - |
| hsa-miR-22-3p | SLIT1 | + | - | - | + |
| hsa-miR-22-3p | SLIT3 | - | - | - | + |
| hsa-miR-22-3p | SLITRK1 | - | - | - | + |
| hsa-miR-22-3p | SLITRK3 | - | - | - | + |
| hsa-miR-22-3p | SLK | - | - | - | + |
| hsa-miR-22-3p | SLMO2 | + | - | - | + |
| hsa-miR-22-3p | SLU7 | - | - | - | + |
| hsa-miR-22-3p | SMAD5 | - | - | - | + |
| hsa-miR-22-3p | SMAP1 | + | - | - | + |
| hsa-miR-22-3p | SMAP2 | - | - | - | + |
| hsa-miR-22-3p | SMARCD1 | - | - | - | + |
| hsa-miR-22-3p | SMARCD2 | - | - | - | + |
| hsa-miR-22-3p | SMC1A | - | - | - | + |
| hsa-miR-22-3p | SMC2 | - | - | - | + |
| hsa-miR-22-3p | SMCO3 | + | - | - | - |
| hsa-miR-22-3p | SMCR7L | - | - | - | + |
| hsa-miR-22-3p | SMG1 | - | - | - | + |
| hsa-miR-22-3p | SMG5 | - | - | - | + |
| hsa-miR-22-3p | SMG7 | + | + | - | + |
| hsa-miR-22-3p | SMIM21 | + | - | - | - |
| hsa-miR-22-3p | SMLR1 | + | + | - | + |
| hsa-miR-22-3p | SMOC1 | - | - | - | + |
| hsa-miR-22-3p | SMOX | - | - | - | + |
| hsa-miR-22-3p | SMTN | - | - | - | + |
| hsa-miR-22-3p | SMTNL2 | - | - | - | + |
| hsa-miR-22-3p | SMU1 | - | - | - | + |
| hsa-miR-22-3p | SMURF1 | - | - | - | + |
| hsa-miR-22-3p | SNAI1 | + | - | - | + |
| hsa-miR-22-3p | SNAP91 | + | - | - | - |
| hsa-miR-22-3p | SNED1 | - | - | - | + |
| hsa-miR-22-3p | SNRK | + | + | - | + |
| hsa-miR-22-3p | SNRNP200 | - | - | - | + |
| hsa-miR-22-3p | SNTA1 | - | - | - | + |
| hsa-miR-22-3p | SNTB1 | - | - | - | + |
| hsa-miR-22-3p | SNTG1 | - | - | - | + |
| hsa-miR-22-3p | SNX10 | - | - | - | + |
| hsa-miR-22-3p | SNX12 | - | - | - | + |
| hsa-miR-22-3p | SNX15 | - | - | - | + |
| hsa-miR-22-3p | SNX16 | - | - | - | + |
| hsa-miR-22-3p | SNX18 | - | - | - | + |
| hsa-miR-22-3p | SNX19 | - | - | - | + |
| hsa-miR-22-3p | SNX24 | - | - | - | + |
| hsa-miR-22-3p | SNX30 | + | - | - | + |
| hsa-miR-22-3p | SNX8 | + | - | - | + |
| hsa-miR-22-3p | SOAT1 | - | - | - | + |
| hsa-miR-22-3p | SOBP | - | - | - | + |
| hsa-miR-22-3p | SOCS1 | - | - | - | + |
| hsa-miR-22-3p | SOCS2 | + | - | - | - |
| hsa-miR-22-3p | SOCS3 | - | - | - | + |
| hsa-miR-22-3p | SOGA1 | - | - | + | - |
| hsa-miR-22-3p | SOGA3 | - | - | - | + |
| hsa-miR-22-3p | SOLH | - | - | - | + |
| hsa-miR-22-3p | SORBS2 | - | - | - | + |
| hsa-miR-22-3p | SORBS3 | - | - | - | + |
| hsa-miR-22-3p | SORL1 | - | - | - | + |
| hsa-miR-22-3p | SOS2 | - | - | - | + |
| hsa-miR-22-3p | SOSTDC1 | - | - | - | + |
| hsa-miR-22-3p | SOWAHA | - | - | - | + |
| hsa-miR-22-3p | SOWAHB | - | - | - | + |
| hsa-miR-22-3p | SOX11 | - | - | - | + |
| hsa-miR-22-3p | SOX13 | - | + | - | + |
| hsa-miR-22-3p | SOX3 | + | - | - | + |
| hsa-miR-22-3p | SOX4 | - | - | - | + |
| hsa-miR-22-3p | SOX5 | - | - | - | + |
| hsa-miR-22-3p | SOX6 | - | - | - | + |
| hsa-miR-22-3p | SP1 | - | + | + | + |
| hsa-miR-22-3p | SP100 | - | + | - | - |
| hsa-miR-22-3p | SP5 | + | - | - | - |
| hsa-miR-22-3p | SPAM1 | - | - | - | + |
| hsa-miR-22-3p | SPARC | - | - | - | + |
| hsa-miR-22-3p | SPATA12 | + | - | - | + |
| hsa-miR-22-3p | SPATA13 | - | - | - | + |
| hsa-miR-22-3p | SPATA18 | - | - | - | + |
| hsa-miR-22-3p | SPATA5 | - | - | - | + |
| hsa-miR-22-3p | SPATS2 | - | - | - | + |
| hsa-miR-22-3p | SPCS2 | - | - | - | + |
| hsa-miR-22-3p | SPG11 | - | - | + | - |
| hsa-miR-22-3p | SPHKAP | - | - | - | + |
| hsa-miR-22-3p | SPIN1 | - | - | - | + |
| hsa-miR-22-3p | SPIN3 | + | - | - | - |
| hsa-miR-22-3p | SPIRE1 | - | - | - | + |
| hsa-miR-22-3p | SPOCK1 | + | + | - | + |
| hsa-miR-22-3p | SPOCK2 | - | - | - | + |
| hsa-miR-22-3p | SPOP | - | - | - | + |
| hsa-miR-22-3p | SPOPL | - | - | - | + |
| hsa-miR-22-3p | SPRED2 | - | - | - | + |
| hsa-miR-22-3p | SPRN | + | - | - | + |
| hsa-miR-22-3p | SPRY3 | - | - | - | + |
| hsa-miR-22-3p | SPRY4 | - | - | - | + |
| hsa-miR-22-3p | SPRYD3 | - | - | - | + |
| hsa-miR-22-3p | SPSB1 | - | - | - | + |
| hsa-miR-22-3p | SPTA1 | + | - | - | - |
| hsa-miR-22-3p | SPTAN1 | - | - | - | + |
| hsa-miR-22-3p | SPTB | - | - | - | + |
| hsa-miR-22-3p | SPTBN5 | - | - | - | + |
| hsa-miR-22-3p | SPTLC2 | - | - | - | + |
| hsa-miR-22-3p | SPTSSA | - | - | - | + |
| hsa-miR-22-3p | SPTY2D1 | - | - | - | + |
| hsa-miR-22-3p | SPZ1 | - | - | - | + |
| hsa-miR-22-3p | SQLE | - | - | - | + |
| hsa-miR-22-3p | SREK1 | - | - | - | + |
| hsa-miR-22-3p | SRGAP3 | - | - | - | + |
| hsa-miR-22-3p | SRPK1 | - | - | + | + |
| hsa-miR-22-3p | SRPK2 | - | - | - | + |
| hsa-miR-22-3p | SRRM4 | - | - | - | + |
| hsa-miR-22-3p | SRSF10 | + | - | - | - |
| hsa-miR-22-3p | SRSF7 | - | - | + | - |
| hsa-miR-22-3p | SRXN1 | + | + | - | + |
| hsa-miR-22-3p | SS18 | - | - | - | + |
| hsa-miR-22-3p | SS18L1 | - | - | - | + |
| hsa-miR-22-3p | SSNA1 | + | - | - | - |
| hsa-miR-22-3p | SSR1 | - | - | - | + |
| hsa-miR-22-3p | ST3GAL1 | - | - | - | + |
| hsa-miR-22-3p | ST6GAL2 | + | - | - | + |
| hsa-miR-22-3p | ST8SIA3 | - | - | - | + |
| hsa-miR-22-3p | STAG2 | + | + | - | + |
| hsa-miR-22-3p | STAM | - | - | - | + |
| hsa-miR-22-3p | STAM2 | + | - | - | + |
| hsa-miR-22-3p | STARD4 | - | - | - | + |
| hsa-miR-22-3p | STAT3 | - | - | - | + |
| hsa-miR-22-3p | STAT5A | + | - | - | + |
| hsa-miR-22-3p | STC1 | - | - | - | + |
| hsa-miR-22-3p | STEAP2 | - | - | - | + |
| hsa-miR-22-3p | STIM1 | - | - | - | + |
| hsa-miR-22-3p | STK25 | + | - | - | - |
| hsa-miR-22-3p | STK26 | - | + | - | + |
| hsa-miR-22-3p | STK32A | - | - | - | + |
| hsa-miR-22-3p | STK33 | - | - | - | + |
| hsa-miR-22-3p | STK35 | + | - | - | + |
| hsa-miR-22-3p | STK38 | - | - | - | + |
| hsa-miR-22-3p | STK39 | + | + | - | + |
| hsa-miR-22-3p | STK4 | - | - | - | + |
| hsa-miR-22-3p | STMN3 | - | - | - | + |
| hsa-miR-22-3p | STMN4 | - | - | - | + |
| hsa-miR-22-3p | STOM | - | - | - | + |
| hsa-miR-22-3p | STOML1 | - | - | - | + |
| hsa-miR-22-3p | STOX2 | + | - | - | - |
| hsa-miR-22-3p | STPG1 | + | - | - | + |
| hsa-miR-22-3p | STRA6 | - | - | - | + |
| hsa-miR-22-3p | STRADB | - | - | - | + |
| hsa-miR-22-3p | STRBP | - | - | - | + |
| hsa-miR-22-3p | STS | - | - | - | + |
| hsa-miR-22-3p | STT3B | - | - | - | + |
| hsa-miR-22-3p | STX12 | - | - | - | + |
| hsa-miR-22-3p | STX16 | - | - | - | + |
| hsa-miR-22-3p | STX17 | + | - | - | - |
| hsa-miR-22-3p | STX4 | + | - | + | - |
| hsa-miR-22-3p | STX6 | + | - | - | + |
| hsa-miR-22-3p | STXBP1 | - | - | - | + |
| hsa-miR-22-3p | STXBP6 | - | - | - | + |
| hsa-miR-22-3p | STYK1 | - | - | - | + |
| hsa-miR-22-3p | STYX | - | + | - | + |
| hsa-miR-22-3p | SUFU | - | - | - | + |
| hsa-miR-22-3p | SULF1 | - | - | - | + |
| hsa-miR-22-3p | SUMO1 | - | - | - | + |
| hsa-miR-22-3p | SUPT16H | - | - | - | + |
| hsa-miR-22-3p | SUPT6H | - | - | - | + |
| hsa-miR-22-3p | SURF4 | - | - | - | + |
| hsa-miR-22-3p | SUSD1 | + | - | - | + |
| hsa-miR-22-3p | SUZ12 | - | - | - | + |
| hsa-miR-22-3p | SV2A | + | + | - | + |
| hsa-miR-22-3p | SV2B | - | - | - | + |
| hsa-miR-22-3p | SWAP70 | - | - | - | + |
| hsa-miR-22-3p | SWT1 | - | - | - | + |
| hsa-miR-22-3p | SYBU | - | - | - | + |
| hsa-miR-22-3p | SYN3 | - | - | - | + |
| hsa-miR-22-3p | SYNCRIP | - | - | - | + |
| hsa-miR-22-3p | SYNDIG1L | - | - | - | + |
| hsa-miR-22-3p | SYNE1 | + | + | - | - |
| hsa-miR-22-3p | SYNGR1 | + | - | - | + |
| hsa-miR-22-3p | SYNPO2 | - | - | - | + |
| hsa-miR-22-3p | SYNPO2L | - | - | - | + |
| hsa-miR-22-3p | SYPL2 | - | - | - | + |
| hsa-miR-22-3p | SYT11 | - | - | - | + |
| hsa-miR-22-3p | SYT13 | + | + | - | + |
| hsa-miR-22-3p | SYT14 | + | - | - | - |
| hsa-miR-22-3p | SZRD1 | - | + | - | - |
| hsa-miR-22-3p | TACR3 | + | + | - | - |
| hsa-miR-22-3p | TADA2B | - | + | - | - |
| hsa-miR-22-3p | TAF1C | + | + | - | - |
| hsa-miR-22-3p | TAF5 | - | - | - | + |
| hsa-miR-22-3p | TAF7 | - | - | - | + |
| hsa-miR-22-3p | TAGLN | + | + | - | - |
| hsa-miR-22-3p | TAGLN2 | - | - | - | + |
| hsa-miR-22-3p | TAL1 | - | - | - | + |
| hsa-miR-22-3p | TANC1 | - | - | - | + |
| hsa-miR-22-3p | TANC2 | - | - | - | + |
| hsa-miR-22-3p | TAOK2 | - | - | - | + |
| hsa-miR-22-3p | TAP1 | + | - | - | + |
| hsa-miR-22-3p | TAP2 | - | - | - | + |
| hsa-miR-22-3p | TAS2R13 | - | - | - | + |
| hsa-miR-22-3p | TAS2R38 | - | - | - | + |
| hsa-miR-22-3p | TAX1BP3 | + | - | - | + |
| hsa-miR-22-3p | TBC1D10B | - | - | - | + |
| hsa-miR-22-3p | TBC1D12 | - | - | + | - |
| hsa-miR-22-3p | TBC1D15 | - | - | - | + |
| hsa-miR-22-3p | TBC1D16 | - | - | - | + |
| hsa-miR-22-3p | TBC1D20 | - | - | - | + |
| hsa-miR-22-3p | TBC1D2B | - | - | - | + |
| hsa-miR-22-3p | TBC1D30 | - | - | - | + |
| hsa-miR-22-3p | TBC1D4 | - | - | - | + |
| hsa-miR-22-3p | TBL1XR1 | - | - | - | + |
| hsa-miR-22-3p | TBX15 | - | - | - | + |
| hsa-miR-22-3p | TBX19 | - | - | - | + |
| hsa-miR-22-3p | TBX3 | - | - | + | + |
| hsa-miR-22-3p | TBX4 | - | - | - | + |
| hsa-miR-22-3p | TCEA1 | - | - | - | + |
| hsa-miR-22-3p | TCEAL1 | - | - | + | + |
| hsa-miR-22-3p | TCEAL2 | - | - | - | + |
| hsa-miR-22-3p | TCEAL8 | - | - | - | + |
| hsa-miR-22-3p | TCEB1 | - | - | - | + |
| hsa-miR-22-3p | TCF20 | - | - | - | + |
| hsa-miR-22-3p | TCF7 | - | - | - | + |
| hsa-miR-22-3p | TCF7L1 | - | - | - | + |
| hsa-miR-22-3p | TCF7L2 | - | - | - | + |
| hsa-miR-22-3p | TCL1A | - | - | - | + |
| hsa-miR-22-3p | TCL1B | + | - | - | + |
| hsa-miR-22-3p | TDG | - | - | - | + |
| hsa-miR-22-3p | TDP1 | - | - | - | + |
| hsa-miR-22-3p | TDRD10 | + | - | - | + |
| hsa-miR-22-3p | TDRKH | + | - | - | - |
| hsa-miR-22-3p | TEAD3 | - | - | - | + |
| hsa-miR-22-3p | TEAD4 | - | - | - | + |
| hsa-miR-22-3p | TECPR2 | - | - | - | + |
| hsa-miR-22-3p | TECTA | + | - | - | - |
| hsa-miR-22-3p | TECTB | - | - | - | + |
| hsa-miR-22-3p | TEF | - | - | - | + |
| hsa-miR-22-3p | TESK2 | + | - | - | - |
| hsa-miR-22-3p | TET1 | - | - | - | + |
| hsa-miR-22-3p | TET2 | - | + | + | - |
| hsa-miR-22-3p | TEX2 | - | - | - | + |
| hsa-miR-22-3p | TEX261 | - | - | - | + |
| hsa-miR-22-3p | TFCP2L1 | - | - | - | + |
| hsa-miR-22-3p | TFG | - | - | - | + |
| hsa-miR-22-3p | TFRC | - | - | + | - |
| hsa-miR-22-3p | TGFBI | + | - | - | - |
| hsa-miR-22-3p | TGFBR1 | + | - | - | + |
| hsa-miR-22-3p | TGIF1 | - | - | - | + |
| hsa-miR-22-3p | TGIF2 | + | - | - | + |
| hsa-miR-22-3p | TGIF2LY | - | - | - | + |
| hsa-miR-22-3p | TGM1 | - | - | - | + |
| hsa-miR-22-3p | TGM6 | + | - | - | + |
| hsa-miR-22-3p | TGOLN2 | - | - | - | + |
| hsa-miR-22-3p | THAP1 | + | - | - | + |
| hsa-miR-22-3p | THAP11 | + | - | - | + |
| hsa-miR-22-3p | THAP2 | - | - | - | + |
| hsa-miR-22-3p | THAP5 | - | - | - | + |
| hsa-miR-22-3p | THEM6 | + | + | - | + |
| hsa-miR-22-3p | THEMIS2 | - | - | - | + |
| hsa-miR-22-3p | THNSL1 | + | - | - | + |
| hsa-miR-22-3p | THPO | - | - | - | + |
| hsa-miR-22-3p | THRAP3 | - | - | - | + |
| hsa-miR-22-3p | THSD4 | - | - | - | + |
| hsa-miR-22-3p | THSD7A | + | - | - | - |
| hsa-miR-22-3p | THUMPD1 | - | - | - | + |
| hsa-miR-22-3p | THUMPD3 | - | - | - | + |
| hsa-miR-22-3p | TIA1 | - | - | - | + |
| hsa-miR-22-3p | TIAM1 | + | + | + | + |
| hsa-miR-22-3p | TIMM10B | + | + | - | + |
| hsa-miR-22-3p | TIMM9 | - | - | - | + |
| hsa-miR-22-3p | TIMP2 | + | - | - | + |
| hsa-miR-22-3p | TIPARP | + | - | - | + |
| hsa-miR-22-3p | TJAP1 | + | + | - | - |
| hsa-miR-22-3p | TJP1 | - | - | - | + |
| hsa-miR-22-3p | TJP2 | - | - | - | + |
| hsa-miR-22-3p | TK2 | - | - | - | + |
| hsa-miR-22-3p | TLK2 | + | + | - | - |
| hsa-miR-22-3p | TLL2 | - | - | - | + |
| hsa-miR-22-3p | TLN1 | - | - | - | + |
| hsa-miR-22-3p | TLN2 | - | - | - | + |
| hsa-miR-22-3p | TLR4 | - | - | - | + |
| hsa-miR-22-3p | TLR8 | + | - | - | + |
| hsa-miR-22-3p | TM9SF3 | - | - | - | + |
| hsa-miR-22-3p | TMBIM1 | + | - | - | + |
| hsa-miR-22-3p | TMC1 | - | - | - | + |
| hsa-miR-22-3p | TMC8 | - | - | - | + |
| hsa-miR-22-3p | TMCC2 | - | - | - | + |
| hsa-miR-22-3p | TMCO4 | + | - | - | + |
| hsa-miR-22-3p | TMED2 | - | - | - | + |
| hsa-miR-22-3p | TMED3 | + | - | - | - |
| hsa-miR-22-3p | TMED4 | + | - | + | + |
| hsa-miR-22-3p | TMED7 | - | - | - | + |
| hsa-miR-22-3p | TMEM104 | - | - | - | + |
| hsa-miR-22-3p | TMEM109 | + | - | - | + |
| hsa-miR-22-3p | TMEM110 | - | - | - | + |
| hsa-miR-22-3p | TMEM120B | - | - | + | + |
| hsa-miR-22-3p | TMEM131 | - | - | - | + |
| hsa-miR-22-3p | TMEM132B | - | - | - | + |
| hsa-miR-22-3p | TMEM132C | - | - | - | + |
| hsa-miR-22-3p | TMEM132E | - | - | - | + |
| hsa-miR-22-3p | TMEM133 | - | - | - | + |
| hsa-miR-22-3p | TMEM136 | - | - | - | + |
| hsa-miR-22-3p | TMEM154 | + | - | - | - |
| hsa-miR-22-3p | TMEM155 | + | + | - | + |
| hsa-miR-22-3p | TMEM159 | + | - | - | - |
| hsa-miR-22-3p | TMEM164 | + | + | - | - |
| hsa-miR-22-3p | TMEM173 | - | - | - | + |
| hsa-miR-22-3p | TMEM178A | - | - | - | + |
| hsa-miR-22-3p | TMEM178B | - | - | + | + |
| hsa-miR-22-3p | TMEM179B | + | - | - | - |
| hsa-miR-22-3p | TMEM19 | - | - | - | + |
| hsa-miR-22-3p | TMEM194A | - | - | - | + |
| hsa-miR-22-3p | TMEM199 | + | + | - | + |
| hsa-miR-22-3p | TMEM200B | - | - | - | + |
| hsa-miR-22-3p | TMEM201 | + | - | - | + |
| hsa-miR-22-3p | TMEM207 | - | - | - | + |
| hsa-miR-22-3p | TMEM214 | - | - | - | + |
| hsa-miR-22-3p | TMEM223 | + | - | - | - |
| hsa-miR-22-3p | TMEM229B | + | - | - | + |
| hsa-miR-22-3p | TMEM230 | - | - | - | + |
| hsa-miR-22-3p | TMEM231 | - | - | - | + |
| hsa-miR-22-3p | TMEM237 | - | - | - | + |
| hsa-miR-22-3p | TMEM245 | - | - | - | + |
| hsa-miR-22-3p | TMEM246 | + | - | - | + |
| hsa-miR-22-3p | TMEM255A | + | - | - | + |
| hsa-miR-22-3p | TMEM26 | - | - | - | + |
| hsa-miR-22-3p | TMEM30A | - | - | - | + |
| hsa-miR-22-3p | TMEM30B | + | - | - | + |
| hsa-miR-22-3p | TMEM33 | + | + | - | + |
| hsa-miR-22-3p | TMEM35 | + | - | - | + |
| hsa-miR-22-3p | TMEM43 | + | - | - | + |
| hsa-miR-22-3p | TMEM47 | - | - | - | + |
| hsa-miR-22-3p | TMEM50A | - | - | - | + |
| hsa-miR-22-3p | TMEM50B | - | + | - | + |
| hsa-miR-22-3p | TMEM62 | - | - | - | + |
| hsa-miR-22-3p | TMEM86A | - | - | - | + |
| hsa-miR-22-3p | TMEM99 | - | - | - | + |
| hsa-miR-22-3p | TMEM9B | - | - | - | + |
| hsa-miR-22-3p | TMIE | - | - | - | + |
| hsa-miR-22-3p | TMLHE | + | - | - | + |
| hsa-miR-22-3p | TMOD2 | - | - | - | + |
| hsa-miR-22-3p | TMOD3 | + | - | - | - |
| hsa-miR-22-3p | TMPRSS11A | + | - | - | - |
| hsa-miR-22-3p | TMPRSS13 | - | - | - | + |
| hsa-miR-22-3p | TMPRSS15 | - | - | - | + |
| hsa-miR-22-3p | TMPRSS9 | - | - | - | + |
| hsa-miR-22-3p | TMSB15A | - | - | - | + |
| hsa-miR-22-3p | TMSB15B | + | + | - | - |
| hsa-miR-22-3p | TMTC1 | - | - | - | + |
| hsa-miR-22-3p | TMX4 | - | - | - | + |
| hsa-miR-22-3p | TNFRSF10D | + | + | + | + |
| hsa-miR-22-3p | TNFRSF13B | - | + | - | - |
| hsa-miR-22-3p | TNFRSF13C | + | - | - | - |
| hsa-miR-22-3p | TNFRSF18 | - | - | - | + |
| hsa-miR-22-3p | TNFRSF1B | + | - | - | + |
| hsa-miR-22-3p | TNFRSF21 | - | - | - | + |
| hsa-miR-22-3p | TNFSF11 | + | - | - | - |
| hsa-miR-22-3p | TNFSF15 | + | - | - | - |
| hsa-miR-22-3p | TNIK | - | - | - | + |
| hsa-miR-22-3p | TNKS2 | + | + | - | + |
| hsa-miR-22-3p | TNN | - | - | - | + |
| hsa-miR-22-3p | TNNI1 | + | - | - | + |
| hsa-miR-22-3p | TNNT2 | + | - | - | - |
| hsa-miR-22-3p | TNRC6B | - | - | - | + |
| hsa-miR-22-3p | TNRC6C | - | - | - | + |
| hsa-miR-22-3p | TNS1 | - | - | - | + |
| hsa-miR-22-3p | TNS4 | - | - | - | + |
| hsa-miR-22-3p | TOB1 | - | - | - | + |
| hsa-miR-22-3p | TOLLIP | - | - | - | + |
| hsa-miR-22-3p | TOM1L2 | - | - | - | + |
| hsa-miR-22-3p | TOMM20 | - | - | - | + |
| hsa-miR-22-3p | TOMM34 | - | - | - | + |
| hsa-miR-22-3p | TOMM6 | + | - | - | - |
| hsa-miR-22-3p | TOMM70A | - | - | - | + |
| hsa-miR-22-3p | TOPBP1 | - | - | - | + |
| hsa-miR-22-3p | TOPORS | - | - | - | + |
| hsa-miR-22-3p | TOR1AIP2 | - | - | - | + |
| hsa-miR-22-3p | TOX | - | - | - | + |
| hsa-miR-22-3p | TP53 | + | - | - | - |
| hsa-miR-22-3p | TP53I11 | - | - | - | + |
| hsa-miR-22-3p | TP53INP1 | + | + | - | + |
| hsa-miR-22-3p | TP63 | - | - | - | + |
| hsa-miR-22-3p | TP73 | - | - | - | + |
| hsa-miR-22-3p | TPD52L2 | + | + | + | + |
| hsa-miR-22-3p | TPI1 | - | + | - | - |
| hsa-miR-22-3p | TPM3 | - | + | - | + |
| hsa-miR-22-3p | TPM4 | - | - | - | + |
| hsa-miR-22-3p | TPP1 | - | - | - | + |
| hsa-miR-22-3p | TPP2 | - | - | - | + |
| hsa-miR-22-3p | TPRX1 | + | - | - | - |
| hsa-miR-22-3p | TRABD2B | + | - | - | - |
| hsa-miR-22-3p | TRAF1 | - | - | - | + |
| hsa-miR-22-3p | TRAF3IP1 | - | - | + | + |
| hsa-miR-22-3p | TRAF4 | - | - | - | + |
| hsa-miR-22-3p | TRAF7 | - | - | - | + |
| hsa-miR-22-3p | TRAK1 | - | - | - | + |
| hsa-miR-22-3p | TRAK2 | - | - | - | + |
| hsa-miR-22-3p | TRAM1L1 | - | - | - | + |
| hsa-miR-22-3p | TRAPPC2L | + | - | - | - |
| hsa-miR-22-3p | TRAT1 | - | - | - | + |
| hsa-miR-22-3p | TREML2 | + | - | - | + |
| hsa-miR-22-3p | TRERF1 | - | - | - | + |
| hsa-miR-22-3p | TRIAP1 | - | - | - | + |
| hsa-miR-22-3p | TRIB1 | - | - | - | + |
| hsa-miR-22-3p | TRIB2 | + | + | - | + |
| hsa-miR-22-3p | TRIM11 | + | - | - | - |
| hsa-miR-22-3p | TRIM13 | - | + | - | + |
| hsa-miR-22-3p | TRIM14 | - | - | - | + |
| hsa-miR-22-3p | TRIM29 | - | - | - | + |
| hsa-miR-22-3p | TRIM31 | - | - | - | + |
| hsa-miR-22-3p | TRIM33 | - | - | - | + |
| hsa-miR-22-3p | TRIM46 | + | + | - | + |
| hsa-miR-22-3p | TRIM66 | + | + | - | + |
| hsa-miR-22-3p | TRIM67 | - | - | - | + |
| hsa-miR-22-3p | TRIM74 | - | - | - | + |
| hsa-miR-22-3p | TRIP11 | + | + | - | + |
| hsa-miR-22-3p | TRMT10A | - | - | - | + |
| hsa-miR-22-3p | TRMT44 | - | - | - | + |
| hsa-miR-22-3p | TRMT61A | - | - | - | + |
| hsa-miR-22-3p | TRMT61B | - | - | - | + |
| hsa-miR-22-3p | TRNAU1AP | - | + | - | + |
| hsa-miR-22-3p | TRNP1 | - | - | - | + |
| hsa-miR-22-3p | TRPC5 | - | - | - | + |
| hsa-miR-22-3p | TRPM7 | + | + | - | + |
| hsa-miR-22-3p | TRPV2 | + | - | - | - |
| hsa-miR-22-3p | TRPV4 | + | - | - | - |
| hsa-miR-22-3p | TRUB1 | + | + | - | + |
| hsa-miR-22-3p | TSC1 | - | - | - | + |
| hsa-miR-22-3p | TSC22D3 | - | - | - | + |
| hsa-miR-22-3p | TSC22D4 | - | - | + | + |
| hsa-miR-22-3p | TSGA10 | - | - | - | + |
| hsa-miR-22-3p | TSHZ1 | - | - | - | + |
| hsa-miR-22-3p | TSHZ2 | - | - | - | + |
| hsa-miR-22-3p | TSHZ3 | - | - | - | + |
| hsa-miR-22-3p | TSLP | - | - | - | + |
| hsa-miR-22-3p | TSPAN11 | + | + | - | + |
| hsa-miR-22-3p | TSPAN14 | - | - | - | + |
| hsa-miR-22-3p | TSPAN15 | + | - | - | + |
| hsa-miR-22-3p | TSPAN18 | - | - | - | + |
| hsa-miR-22-3p | TSPAN33 | + | - | - | - |
| hsa-miR-22-3p | TSPAN5 | + | - | - | - |
| hsa-miR-22-3p | TSPYL5 | - | - | - | + |
| hsa-miR-22-3p | TSTD1 | + | - | - | + |
| hsa-miR-22-3p | TTBK1 | - | - | - | + |
| hsa-miR-22-3p | TTBK2 | - | - | - | + |
| hsa-miR-22-3p | TTC16 | + | - | - | - |
| hsa-miR-22-3p | TTC28 | - | - | - | + |
| hsa-miR-22-3p | TTC3 | - | - | - | + |
| hsa-miR-22-3p | TTC31 | + | - | - | - |
| hsa-miR-22-3p | TTC33 | - | - | + | - |
| hsa-miR-22-3p | TTC39B | - | - | - | + |
| hsa-miR-22-3p | TTC9 | + | - | - | + |
| hsa-miR-22-3p | TTL | + | - | - | - |
| hsa-miR-22-3p | TTLL12 | - | - | - | + |
| hsa-miR-22-3p | TTLL6 | + | - | - | - |
| hsa-miR-22-3p | TTLL9 | + | - | - | - |
| hsa-miR-22-3p | TTPA | - | - | - | + |
| hsa-miR-22-3p | TTYH3 | + | + | - | + |
| hsa-miR-22-3p | TUB | + | - | - | + |
| hsa-miR-22-3p | TUBA3C | - | - | - | + |
| hsa-miR-22-3p | TUBA3D | - | - | - | + |
| hsa-miR-22-3p | TUBA3E | - | - | - | + |
| hsa-miR-22-3p | TUBA8 | - | - | - | + |
| hsa-miR-22-3p | TUBD1 | + | - | - | - |
| hsa-miR-22-3p | TUFM | + | - | - | - |
| hsa-miR-22-3p | TULP1 | - | - | - | + |
| hsa-miR-22-3p | TULP3 | - | - | - | + |
| hsa-miR-22-3p | TXLNA | + | - | - | + |
| hsa-miR-22-3p | TXLNG | - | - | - | + |
| hsa-miR-22-3p | TXNDC5 | - | - | - | + |
| hsa-miR-22-3p | TXNL4B | - | - | - | + |
| hsa-miR-22-3p | TXNRD3NB | + | - | - | + |
| hsa-miR-22-3p | TYRO3 | + | + | - | - |
| hsa-miR-22-3p | UACA | - | - | - | + |
| hsa-miR-22-3p | UBA1 | - | - | - | + |
| hsa-miR-22-3p | UBA7 | - | - | - | + |
| hsa-miR-22-3p | UBALD1 | + | - | - | - |
| hsa-miR-22-3p | UBAP1 | + | - | - | - |
| hsa-miR-22-3p | UBASH3B | - | - | - | + |
| hsa-miR-22-3p | UBE2D1 | - | - | - | + |
| hsa-miR-22-3p | UBE2D3 | - | - | - | + |
| hsa-miR-22-3p | UBE2I | - | - | - | + |
| hsa-miR-22-3p | UBE2J1 | - | - | - | + |
| hsa-miR-22-3p | UBE2K | - | + | - | + |
| hsa-miR-22-3p | UBE2NL | + | - | - | - |
| hsa-miR-22-3p | UBE2Z | + | + | - | + |
| hsa-miR-22-3p | UBE3A | - | - | - | + |
| hsa-miR-22-3p | UBE4A | - | - | - | + |
| hsa-miR-22-3p | UBFD1 | - | - | - | + |
| hsa-miR-22-3p | UBL4A | - | - | - | + |
| hsa-miR-22-3p | UBL4B | + | - | - | + |
| hsa-miR-22-3p | UBOX5 | - | - | - | + |
| hsa-miR-22-3p | UBQLNL | - | - | - | + |
| hsa-miR-22-3p | UBR5 | - | - | - | + |
| hsa-miR-22-3p | UBTD1 | - | - | - | + |
| hsa-miR-22-3p | UBXN1 | - | - | - | + |
| hsa-miR-22-3p | UBXN4 | - | - | - | + |
| hsa-miR-22-3p | UGCG | + | - | - | + |
| hsa-miR-22-3p | UHRF1BP1 | - | - | - | + |
| hsa-miR-22-3p | UHRF2 | + | - | - | - |
| hsa-miR-22-3p | ULBP1 | + | - | - | + |
| hsa-miR-22-3p | ULBP3 | + | - | - | - |
| hsa-miR-22-3p | ULK1 | - | - | - | + |
| hsa-miR-22-3p | ULK2 | + | + | - | + |
| hsa-miR-22-3p | ULK4 | - | - | - | + |
| hsa-miR-22-3p | UNC119B | - | + | - | + |
| hsa-miR-22-3p | UNC5B | - | - | - | + |
| hsa-miR-22-3p | UNC5C | - | - | - | + |
| hsa-miR-22-3p | UNC5D | - | + | - | + |
| hsa-miR-22-3p | UNC79 | + | - | - | + |
| hsa-miR-22-3p | UNG | + | - | - | - |
| hsa-miR-22-3p | UNK | + | + | - | - |
| hsa-miR-22-3p | UPF3B | - | - | - | + |
| hsa-miR-22-3p | UPK1B | - | - | - | + |
| hsa-miR-22-3p | UQCR11 | - | - | - | + |
| hsa-miR-22-3p | URB1 | - | - | - | + |
| hsa-miR-22-3p | USP12 | - | - | - | + |
| hsa-miR-22-3p | USP13 | - | - | - | + |
| hsa-miR-22-3p | USP20 | + | - | - | + |
| hsa-miR-22-3p | USP25 | - | - | - | + |
| hsa-miR-22-3p | USP30 | - | - | - | + |
| hsa-miR-22-3p | USP37 | - | + | - | + |
| hsa-miR-22-3p | USP40 | - | - | - | + |
| hsa-miR-22-3p | USP43 | + | - | - | + |
| hsa-miR-22-3p | USP44 | + | - | - | + |
| hsa-miR-22-3p | USP46 | - | - | - | + |
| hsa-miR-22-3p | USP47 | - | - | - | + |
| hsa-miR-22-3p | USP8 | + | - | - | - |
| hsa-miR-22-3p | UST | - | - | - | + |
| hsa-miR-22-3p | UTP14C | - | - | - | + |
| hsa-miR-22-3p | UTP18 | + | + | - | - |
| hsa-miR-22-3p | UTS2D | - | - | - | + |
| hsa-miR-22-3p | VAMP1 | - | - | - | + |
| hsa-miR-22-3p | VAMP3 | - | - | - | + |
| hsa-miR-22-3p | VAPB | + | - | + | + |
| hsa-miR-22-3p | VASH1 | - | - | - | + |
| hsa-miR-22-3p | VASN | - | - | + | - |
| hsa-miR-22-3p | VASP | + | + | - | - |
| hsa-miR-22-3p | VAT1 | + | - | - | + |
| hsa-miR-22-3p | VAV3 | - | - | - | + |
| hsa-miR-22-3p | VAX1 | - | - | - | + |
| hsa-miR-22-3p | VCP | - | - | - | + |
| hsa-miR-22-3p | VCPIP1 | - | - | - | + |
| hsa-miR-22-3p | VDAC1 | - | - | - | + |
| hsa-miR-22-3p | VEZF1 | - | + | - | - |
| hsa-miR-22-3p | VGLL4 | - | - | - | + |
| hsa-miR-22-3p | VIPR1 | - | - | - | + |
| hsa-miR-22-3p | VIT | - | - | - | + |
| hsa-miR-22-3p | VN1R4 | - | - | - | + |
| hsa-miR-22-3p | VOPP1 | + | - | - | + |
| hsa-miR-22-3p | VPRBP | - | + | - | + |
| hsa-miR-22-3p | VPS37B | - | - | - | + |
| hsa-miR-22-3p | VPS41 | - | - | - | + |
| hsa-miR-22-3p | VPS52 | + | - | - | - |
| hsa-miR-22-3p | VPS53 | - | - | - | + |
| hsa-miR-22-3p | VRTN | - | - | - | + |
| hsa-miR-22-3p | VSIG10 | - | - | - | + |
| hsa-miR-22-3p | VSIG4 | - | - | - | + |
| hsa-miR-22-3p | VSNL1 | + | - | + | - |
| hsa-miR-22-3p | VSTM1 | + | - | - | - |
| hsa-miR-22-3p | VSTM2A | - | - | - | + |
| hsa-miR-22-3p | VSTM2L | - | - | - | + |
| hsa-miR-22-3p | VTI1A | - | - | - | + |
| hsa-miR-22-3p | VWA5A | - | - | - | + |
| hsa-miR-22-3p | WAC | - | - | - | + |
| hsa-miR-22-3p | WAPAL | + | - | - | + |
| hsa-miR-22-3p | WARS2 | + | + | - | + |
| hsa-miR-22-3p | WASF1 | + | + | - | + |
| hsa-miR-22-3p | WASF2 | + | - | - | - |
| hsa-miR-22-3p | WBP4 | - | - | - | + |
| hsa-miR-22-3p | WBSCR17 | + | - | - | + |
| hsa-miR-22-3p | WDFY1 | - | - | - | + |
| hsa-miR-22-3p | WDFY3 | - | + | - | - |
| hsa-miR-22-3p | WDR26 | - | - | - | + |
| hsa-miR-22-3p | WDR33 | - | + | - | - |
| hsa-miR-22-3p | WDR4 | - | - | - | + |
| hsa-miR-22-3p | WDR46 | - | - | - | + |
| hsa-miR-22-3p | WDR47 | - | - | - | + |
| hsa-miR-22-3p | WDR77 | - | - | - | + |
| hsa-miR-22-3p | WDR82 | + | + | - | + |
| hsa-miR-22-3p | WDR85 | - | - | - | + |
| hsa-miR-22-3p | WDR93 | + | - | - | - |
| hsa-miR-22-3p | WDTC1 | + | + | - | + |
| hsa-miR-22-3p | WEE2 | - | - | - | + |
| hsa-miR-22-3p | WFIKKN2 | - | - | - | + |
| hsa-miR-22-3p | WFS1 | - | - | - | + |
| hsa-miR-22-3p | WHSC1L1 | + | - | - | + |
| hsa-miR-22-3p | WIPF1 | - | - | - | + |
| hsa-miR-22-3p | WIPF2 | - | - | - | + |
| hsa-miR-22-3p | WLS | - | - | - | + |
| hsa-miR-22-3p | WNK1 | + | - | - | + |
| hsa-miR-22-3p | WNK3 | - | - | - | + |
| hsa-miR-22-3p | WNT1 | - | - | + | - |
| hsa-miR-22-3p | WNT10B | + | - | - | - |
| hsa-miR-22-3p | WNT3 | - | - | - | + |
| hsa-miR-22-3p | WNT4 | + | - | - | - |
| hsa-miR-22-3p | WNT8A | - | - | - | + |
| hsa-miR-22-3p | WNT8B | + | - | - | + |
| hsa-miR-22-3p | WRNIP1 | + | + | - | - |
| hsa-miR-22-3p | WSCD2 | - | - | - | + |
| hsa-miR-22-3p | WTAP | - | - | - | + |
| hsa-miR-22-3p | WWC1 | - | - | + | + |
| hsa-miR-22-3p | WWC2 | + | + | - | - |
| hsa-miR-22-3p | WWC3 | - | - | - | + |
| hsa-miR-22-3p | WWP2 | - | - | - | + |
| hsa-miR-22-3p | WWTR1 | - | - | - | + |
| hsa-miR-22-3p | XAGE3 | - | - | - | + |
| hsa-miR-22-3p | XCL1 | - | - | - | + |
| hsa-miR-22-3p | XG | + | - | - | - |
| hsa-miR-22-3p | XIAP | - | - | - | + |
| hsa-miR-22-3p | XIRP1 | - | - | - | + |
| hsa-miR-22-3p | XIRP2 | - | - | - | + |
| hsa-miR-22-3p | XPNPEP3 | - | - | - | + |
| hsa-miR-22-3p | XPO7 | - | - | - | + |
| hsa-miR-22-3p | XPR1 | + | - | - | + |
| hsa-miR-22-3p | XRN1 | - | - | - | + |
| hsa-miR-22-3p | XXbac-BPG32J3.20 | + | - | - | - |
| hsa-miR-22-3p | XYLT1 | - | - | - | + |
| hsa-miR-22-3p | YAF2 | - | - | - | + |
| hsa-miR-22-3p | YAP1 | - | - | - | + |
| hsa-miR-22-3p | YARS | + | + | - | + |
| hsa-miR-22-3p | YBX2 | + | - | - | + |
| hsa-miR-22-3p | YIPF6 | - | - | - | + |
| hsa-miR-22-3p | YOD1 | - | - | - | + |
| hsa-miR-22-3p | YPEL2 | - | - | - | + |
| hsa-miR-22-3p | YTHDC2 | - | - | - | + |
| hsa-miR-22-3p | YWHAE | - | - | - | + |
| hsa-miR-22-3p | YWHAG | - | - | - | + |
| hsa-miR-22-3p | YWHAQ | - | - | - | + |
| hsa-miR-22-3p | YWHAZ | + | + | + | - |
| hsa-miR-22-3p | ZBED1 | - | - | - | + |
| hsa-miR-22-3p | ZBTB1 | - | - | - | + |
| hsa-miR-22-3p | ZBTB18 | - | - | - | + |
| hsa-miR-22-3p | ZBTB3 | + | - | - | + |
| hsa-miR-22-3p | ZBTB33 | - | - | - | + |
| hsa-miR-22-3p | ZBTB39 | + | + | - | + |
| hsa-miR-22-3p | ZBTB40 | - | - | - | + |
| hsa-miR-22-3p | ZBTB42 | + | - | - | - |
| hsa-miR-22-3p | ZBTB44 | - | - | - | + |
| hsa-miR-22-3p | ZBTB46 | - | - | - | + |
| hsa-miR-22-3p | ZBTB47 | - | + | - | - |
| hsa-miR-22-3p | ZC3H12C | - | - | - | + |
| hsa-miR-22-3p | ZC3H13 | - | - | - | + |
| hsa-miR-22-3p | ZC3H7B | - | - | - | + |
| hsa-miR-22-3p | ZC3HAV1L | - | - | - | + |
| hsa-miR-22-3p | ZCCHC14 | + | + | - | + |
| hsa-miR-22-3p | ZCCHC24 | - | - | - | + |
| hsa-miR-22-3p | ZDBF2 | - | - | - | + |
| hsa-miR-22-3p | ZDHHC16 | + | + | - | + |
| hsa-miR-22-3p | ZDHHC3 | - | - | - | + |
| hsa-miR-22-3p | ZDHHC9 | - | - | - | + |
| hsa-miR-22-3p | ZER1 | - | - | - | + |
| hsa-miR-22-3p | ZFP36L2 | - | - | - | + |
| hsa-miR-22-3p | ZFP91 | - | - | - | + |
| hsa-miR-22-3p | ZFR2 | - | - | - | + |
| hsa-miR-22-3p | ZFYVE20 | - | - | + | - |
| hsa-miR-22-3p | ZFYVE9 | - | + | - | + |
| hsa-miR-22-3p | ZHX3 | - | - | - | + |
| hsa-miR-22-3p | ZIC3 | - | - | - | + |
| hsa-miR-22-3p | ZIC4 | - | - | - | + |
| hsa-miR-22-3p | ZKSCAN1 | - | - | - | + |
| hsa-miR-22-3p | ZMAT2 | - | - | - | + |
| hsa-miR-22-3p | ZMAT3 | - | - | - | + |
| hsa-miR-22-3p | ZMAT5 | + | - | + | - |
| hsa-miR-22-3p | ZMYM4 | + | + | - | + |
| hsa-miR-22-3p | ZMYM6NB | - | - | - | + |
| hsa-miR-22-3p | ZNF106 | + | - | - | + |
| hsa-miR-22-3p | ZNF107 | - | - | - | + |
| hsa-miR-22-3p | ZNF124 | - | - | - | + |
| hsa-miR-22-3p | ZNF197 | - | - | - | + |
| hsa-miR-22-3p | ZNF211 | + | - | - | - |
| hsa-miR-22-3p | ZNF217 | - | - | + | - |
| hsa-miR-22-3p | ZNF276 | - | - | - | + |
| hsa-miR-22-3p | ZNF280C | - | - | - | + |
| hsa-miR-22-3p | ZNF280D | - | - | - | + |
| hsa-miR-22-3p | ZNF292 | - | - | - | + |
| hsa-miR-22-3p | ZNF295 | - | - | - | + |
| hsa-miR-22-3p | ZNF3 | - | - | - | + |
| hsa-miR-22-3p | ZNF317 | + | - | - | - |
| hsa-miR-22-3p | ZNF323 | - | - | - | + |
| hsa-miR-22-3p | ZNF335 | - | - | - | + |
| hsa-miR-22-3p | ZNF362 | - | - | - | + |
| hsa-miR-22-3p | ZNF365 | - | - | - | + |
| hsa-miR-22-3p | ZNF394 | + | - | - | - |
| hsa-miR-22-3p | ZNF395 | - | - | - | + |
| hsa-miR-22-3p | ZNF423 | - | - | - | + |
| hsa-miR-22-3p | ZNF425 | - | - | - | + |
| hsa-miR-22-3p | ZNF436 | - | - | - | + |
| hsa-miR-22-3p | ZNF449 | - | - | - | + |
| hsa-miR-22-3p | ZNF45 | - | - | - | + |
| hsa-miR-22-3p | ZNF451 | + | - | - | + |
| hsa-miR-22-3p | ZNF460 | - | - | + | + |
| hsa-miR-22-3p | ZNF462 | - | - | - | + |
| hsa-miR-22-3p | ZNF498 | - | - | - | + |
| hsa-miR-22-3p | ZNF500 | + | - | - | - |
| hsa-miR-22-3p | ZNF503-AS2 | - | - | - | + |
| hsa-miR-22-3p | ZNF512 | - | - | - | + |
| hsa-miR-22-3p | ZNF512B | - | + | - | - |
| hsa-miR-22-3p | ZNF516 | - | - | - | + |
| hsa-miR-22-3p | ZNF517 | + | - | - | - |
| hsa-miR-22-3p | ZNF530 | - | - | - | + |
| hsa-miR-22-3p | ZNF536 | - | - | - | + |
| hsa-miR-22-3p | ZNF543 | - | - | - | + |
| hsa-miR-22-3p | ZNF546 | - | - | - | + |
| hsa-miR-22-3p | ZNF555 | - | - | - | + |
| hsa-miR-22-3p | ZNF584 | + | - | - | - |
| hsa-miR-22-3p | ZNF585A | + | - | - | + |
| hsa-miR-22-3p | ZNF594 | + | - | - | - |
| hsa-miR-22-3p | ZNF599 | + | - | - | - |
| hsa-miR-22-3p | ZNF605 | + | - | - | - |
| hsa-miR-22-3p | ZNF607 | - | - | - | + |
| hsa-miR-22-3p | ZNF609 | + | + | - | + |
| hsa-miR-22-3p | ZNF618 | + | - | - | + |
| hsa-miR-22-3p | ZNF629 | - | - | - | + |
| hsa-miR-22-3p | ZNF641 | - | - | - | + |
| hsa-miR-22-3p | ZNF646 | + | - | + | - |
| hsa-miR-22-3p | ZNF662 | + | - | + | - |
| hsa-miR-22-3p | ZNF671 | - | - | - | + |
| hsa-miR-22-3p | ZNF681 | - | - | - | + |
| hsa-miR-22-3p | ZNF689 | + | - | - | + |
| hsa-miR-22-3p | ZNF7 | - | - | - | + |
| hsa-miR-22-3p | ZNF704 | - | - | - | + |
| hsa-miR-22-3p | ZNF705A | + | - | - | + |
| hsa-miR-22-3p | ZNF705B | + | - | - | - |
| hsa-miR-22-3p | ZNF705D | + | - | - | + |
| hsa-miR-22-3p | ZNF705G | + | - | - | - |
| hsa-miR-22-3p | ZNF706 | - | + | - | + |
| hsa-miR-22-3p | ZNF707 | + | - | - | - |
| hsa-miR-22-3p | ZNF736 | + | - | - | - |
| hsa-miR-22-3p | ZNF740 | + | - | - | - |
| hsa-miR-22-3p | ZNF747 | - | - | - | + |
| hsa-miR-22-3p | ZNF792 | - | - | - | + |
| hsa-miR-22-3p | ZNF80 | - | - | - | + |
| hsa-miR-22-3p | ZNF821 | + | - | - | - |
| hsa-miR-22-3p | ZNF853 | - | - | - | + |
| hsa-miR-22-3p | ZNF862 | - | - | - | + |
| hsa-miR-22-3p | ZNFX1 | - | - | - | + |
| hsa-miR-22-3p | ZNHIT6 | + | - | - | + |
| hsa-miR-22-3p | ZNRF1 | - | - | - | + |
| hsa-miR-22-3p | ZNRF3 | - | - | - | + |
| hsa-miR-22-3p | ZSCAN1 | + | - | - | + |
| hsa-miR-22-3p | ZSCAN22 | - | - | - | + |
| hsa-miR-22-3p | ZSCAN23 | - | - | - | + |
| hsa-miR-22-3p | ZSWIM5 | - | - | - | + |
| hsa-miR-22-3p | ZSWIM6 | - | - | - | + |
| hsa-miR-22-3p | ZWINT | - | - | - | + |
| hsa-miR-22-3p | ZXDB | - | - | - | + |
| hsa-miR-22-3p | ZYG11B | - | - | - | + |
| hsa-miR-22-3p | ZZEF1 | - | - | - | + |
| hsa-miR-22-3p | hsa-mir-4763 | - | - | - | + |
